# Super-Armed Thiomannopyranosides in the Synthesis of a Mannose-Capped Trisaccharide of *Mycobacterium tuberculosis* Lipoarabinomannan

**DOI:** 10.3390/molecules31101598

**Published:** 2026-05-10

**Authors:** Polina Igorevna Abronina, Zinaida Vladimirovna Kuznetsova, Dmitry Sergeevich Novikov, Alexander Ivanovich Zinin, Natalya G. Georgievna Kolotyrkina, Leonid Olegovich Kononov

**Affiliations:** N.D. Zelinsky Institute of Organic Chemistry, Russian Academy of Sciences, Leninsky prosp., 47, Moscow 119991, Russia; 2kuznecova.z.v2@gmail.com (Z.V.K.); novikov-ds@ioc.ac.ru (D.S.N.); nkolotyr@ioc.ac.ru (N.G.G.K.)

**Keywords:** glycosylation, d-arabinofuranose, d-mannopyranose, silyl group, *Mycobacterium tuberculosis*, one pot synthesis

## Abstract

Silylation of phenyl 1-thio-α-d-mannopyranoside, ethyl 1-thio-α- and β-d-mannopyranosides under different conditions was studied. Low-temperature NMR analysis revealed that the silylated products typically exist as an equilibrium of two chair conformations with a predominance of the axially rich ^1^*C*_4_ conformation. The dependence of the ratio of conformers on the anomeric configuration, the type of silyl groups and the nature of the aglycone was established. The fully silylated phenyl 1-thio-α-d-mannopyranoside, ethyl 1-thio-α- and β-d-mannopyranosides were tested as glycosyl donors in the synthesis of di- and trisaccharides, including one-pot synthesis. In all cases, only α-linked oligosaccharides as mixtures of conformers were formed. The obtained deprotected Man*p*-(1→2)-α-d-Man*p-*(1→5)-α-d-Ara*f* trisaccharide 2-azidoethyl glycoside, related to the non-reducing terminal fragment of the mannose-capped lipoarabinomannan (ManLAM) of *Mycobacterium tuberculosis*, can be used for further preparation of conjugates with proteins to provide antigens, which are important for development of new tuberculosis screening assays.

## 1. Introduction

Mycobacterial infections have received significant attention due to the rising number of cases globally, while tuberculosis (TB), which is caused by *Mycobacterium tuberculosis*, continues to be one of the most dangerous infectious diseases [1].

A major mycobacterial cell wall glycan is lipoarabinomannan (LAM), which plays a critical role in mycobacteria–host interactions [2]. A significant number of immune processes related to mycobacterial infection are associated with LAM, which serves as the main non-protein antigen in mycobacteria [3]. The structure of LAM consists of a phosphatidyl-*myo*-inositol moiety, a core d-mannopyranose region and d-arabinofuranose domain with α-(1→5)-, α-(1→3)-, and β-(1→2)-glycosidic bonds. The terminal β-(1→2)-diarabinofuranoside fragments of the LAM of *M. tuberculosis* and others pathogenic strains, such as *M. bovis* and *M. leprae,* are often glycosylated with one to three α-1,2-linked mannopyranose residues (resulting in ManLAM) [4]. Endocytic receptors, including the macrophage mannose receptor and DC-SIGN, present on the surface of dendritic cells, thus playing an important role in host–cell recognition, interact predominantly with mannose-containing caps found on the ManLAM [5].

The LAM fragments are attractive targets for the development of TB diagnostic and therapeutic strategies [3,6,7,8,9]. It should be noted that despite the fact that many papers describe preparation of oligoarabinofuranosides [10,11,12,13,14,15,16,17,18,19,20,21,22], the synthesis of LAM fragments containing d-mannopyranose and d-arabinofuranose residues and, most importantly, neoglycoconjugates, thereof remains a challenge [23,24,25,26,27,28].

Generally, during the synthesis of oligoarabinofuranosides, capped with monosaccharide d-mannopyranose residues, the stereoselective formation of an α-glycosidic linkage is enabled by the presence of a participating acyl group at O-2 of the glycosyl donor [27]. Oligosaccharides with di- and trimannose caps can be synthesized by stepwise introduction of monosaccharide blocks. However, such a strategy increases the number of synthetic steps due to protective groups manipulations. Direct use of di- and trimannose building blocks is more challenging, since the effect of a neighboring participating group at O-2 cannot be applied to control the stereoselectivity. For example, when partially benzylated α-(1→2)-linked dimannopyranose thioglycosides were used for the glycosylation of the primary position of β-(1→2)-linked diarabinofuranosides, the formation of anomeric mixtures was observed [29].

A 1,2-*trans* glycosidic linkage can also be created by the use of polysilylated “super-armed” glycosyl donors. The conformational features of a number of silylated glycosyl donors were investigated previously (for a review, see [30]). In particular, it was shown that 6-*O*-benzyl-2,3,4-tris-*O*-(*tert*-butyldimethylsilyl (TBS))-1-thio-α-d-mannopyranoside exists as a mixture of ^4^*C*_1_ and ^1^*C*_4_ conformers in an 1:1 ratio (^1^H NMR, −35 °C) [31]. It was found that its reactions with “armed” phenyl tri-*O*-benzyl-β-d-thioglucosides led predominantly to α-linked glycosides (as was established after desilylation) [31]. Except the above data, no other information is currently available, to the best of our knowledge, regarding the synthesis and reactivity of variously protected polysilylated thiomannopyranosides, which could be valuable for synthesizing biologically important oligosaccharides.

**Figure 1:**
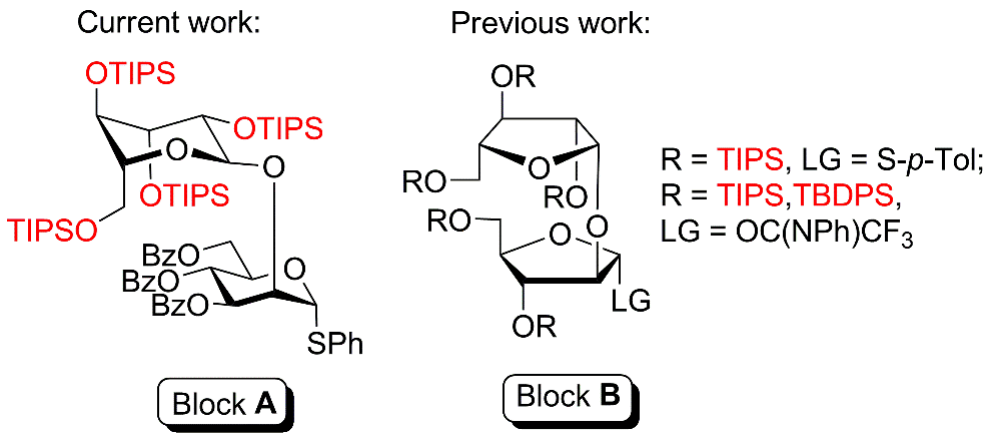
**Current work**: the use of α-(1→2)-linked dimannopyranose glycosyl donors with TIPS groups at the non-reducing end (Block (**A**)) for the preparation of mannose-capped trisaccharide of *M. tuberculosis* (ManLAM). **Previous work**: Diarabinofuranose Ara-β-(1→2)-Ara glycosyl donors for stereoselective 1,2-*trans*-glycosylation in the synthesis of the terminal fragments of arabinogalactan and LAM of *M. tuberculosis* (Block (**B**)), LG = S-*p*-Tol [32,33,34], LG = OC(NPh)CF_3_ [35,36].

In this work, we aimed at studying the silylation of phenyl 1-thio-α-d-mannopyranoside, ethyl 1-thio-α- and β-d-mannopyranosides under different conditions. Additionally, we focused on the investigation of conformational behavior and glycosylation ability of the obtained polysilylated thiomannopyranosides. We planned to perform one-pot synthesis of mannose-capped trisaccharide of the *M. tuberculosis* ManLAM and to compare our results with the use of α-(1→2)-linked dimannopyranoside (Block (**A**), Figure 1).

Recently, we successfully applied [32,33,34,35,36,37] silylated Ara-β-(1→2)-Ara diarabinofuranosides as glycosyl donors lacking 2-*O*-acyl participating groups for stereoselective 1,2-*trans*-glycosylation leading to the non-reducing terminal fragments of the arabinogalactan and LAM of *M. tuberculosis* (Block (**B**), Figure 1).

The current research may be regarded as an extension of super–armed concept based on the difference of the reactivity between glycosyl donors having silyl and benzyl-protecting groups [30]. We focused on the investigation of the reactivity of polysilylated mannopyranosyl donors with disarmed glycosyl acceptors, having benzoyl protection. It allows us to realize a benzyl-free strategy [34] and to expand the library of terminal oligoarabinofuranosides (with 2-azidoethyl, 4-(2-azidoethoxy)phenyl (AEP) and 4-(3-azidopropoxy)phenyl (APP) aglycones) related to the fragments of *M. tuberculosis* LAM [34]. These oligosaccharides can be used for further preparation of conjugates with proteins to provide antigens, which are important for development of new tuberculosis screening assays [38].

## 2. Results and Discussion

### 2.1. Silylation of Phenyl 1-thio-α-d-Mannopyranoside (***1***): The Analysis of Conformational Features of Super-Armed Silylated Thiomannopyranosides ***2***, ***5***, ***7*** According to NMR Data

**Scheme 1:**
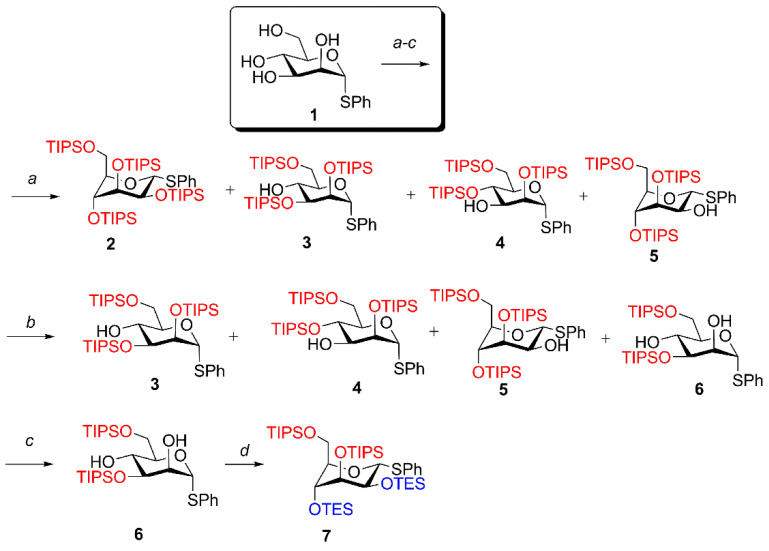
*Reagents and conditions*: *a*. TIPSOTf, 2,4,6-collidine, 90 °C, 45 h, **2** (63%), **3** (16%), **4** (10%), **5** (9%); *b*. TIPSCl (6 equiv.), imidazole, DMF, 90 °C, 52 h, **3** (21%), **4** (22%), **5** (21%), **6** (26%); *c*. TIPSCl (2 equiv.), imidazole, DMF, 70 °C, 72 h, **6** (85%); *d.* TESOTf, 2,4,6-collidine, 20 °C, 2 h, **7** (78%).

First, silylation of phenyl 1-thio-α-d-mannopyranoside (**1**) was investigated under various conditions (Scheme 1). According to Method A (TIPSOTf, 2,4,6-collidine, 90 °C, 45 h), fully silylated mannopyranoside (**2**) as the main product (63%) and a series of regioisomers (**3**–**5**) with one hydroxy group were obtained. As expected, the presence of several conformations is typical for compounds **2** and **5** due to the ring flip after the introduction of bulky silyl substituents at O-3 and O-4.

Silylation of phenyl 1-thio-α-d-mannopyranoside (**1**) under conditions of Method B (TIPSCl (6 equiv.), imidazole, DMF, 90 °C, 52 h) was incomplete and less selective. We obtained the following compounds: mannopyranoside **6** with two TIPS groups at O-3 and O-6, as well as mannopyranosides **3**–**5** with OH groups at O-4, O-3 and O-2, respectively. It should be noted that we did not observe the formation of fully silylated mannopyranoside (**2**) at all.

Besides, we directly obtained mannopyranoside **6** (82%) with two TIPS groups at O-3 and O-6 by silylation of phenyl 1-thio-α-d-mannopyranoside (**1**) under milder conditions of Method C (TIPSCl (2 equiv.), imidazole, DMF, 70 °C, 72 h). Subsequent introduction of TES groups under the conditions of Method D (TESOTf, 2,4,6-collidine, 20 °C, 2 h) resulted in formation of a fully silylated thioglycoside **7**.

We analyzed the ^1^*C*_4_ (A) to ^4^*C*_1_ (B) ratio for super-armed silylated thiomannopyranosides **2**, **5**, **7** according to NMR data (Table 1, Table 2 and Table 3). The A:B ratio for the mannosyl donor **2** could not be measured at 298 K and 323 K since the peaks in the NMR spectra were too broad for characterization. At the same time, we observed 10/1.6 ratio for two conformers of **2**, when the ^1^H NMR spectrum was recorded at 243 K.

The values *J*_H1–H2_ = 8.5 Hz (243 K), *J*_H1–H2_ = 7.6 Hz (298 K), *J*_H1–H2_ = 7.7 Hz (323 K) for silylated phenyl 1-thio-α-d-mannopyranoside (**2**) suggest a *trans*-diaxial relationship between H-1 and H-2, corresponding to ^1^*C*_4_ (A) conformer. Conversely, the value of *J*_H2–H1_ = 1.8 Hz (243 K) indicated *trans*-diequatorial relationship between H-1 and H-2 and the existence of ^4^*C*_1_ (B) conformer. At the same time, the compound **5** with OH group at O-2 exists exclusively as ^1^*C*_4_ conformer even at 298 K. We found the correlation of the signal of the anomeric proton of **5** at δ_H_ 5.06 (d, 1H, *J* 9.3 Hz, H-1) ppm in a ^1^H NMR spectrum with the carbon signal at δ_C_ 82.2 (C-1). For phenyl 1-thio-α-d-mannopyranoside (**7**), containing less bulky TES-groups at O-2 and O-4, the abundance of the ^4^*C*_1_ conformer increased (^1^*C*_4_ (A) to ^4^*C*_1_ (B) ratio = 1/1 (240 K)). On the contrary, ^4^*C*_1_ conformation is typical for compounds **3** (*J*_H1–H2_ = 1.8 Hz) and **4** (*J*_H1–H2_ = 1.6 Hz).

### 2.2. The Comparison of Silylation of Ethyl 1-thio-α- and β-d-Mannopyranosides (***8***, ***9***)

**Scheme 2:**
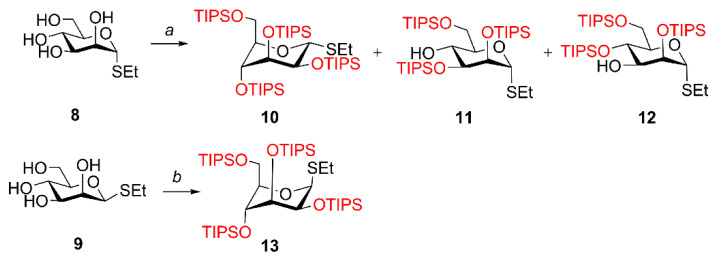
*Reagents and conditions*: *a*. TIPSOTf, 2,4,6-collidine, 90 °C, 0.5 h, **10** (51%), **11** (26%), **12** (6%); *b*. TIPSOTf, collidine, 90 °C, 0.5 h, **13** (84%).

The data obtained above were compared with the results of the silylation of ethyl 1-thio-α- and β-d-mannopyranosides (**8** and **9**), obtained from corresponding ethyl tetra-*O*-acetyl-1-thio-α- and β-d-mannopyranosides [39,40]. We used the same conditions as for phenyl 1-thio-α-d-mannopyranoside **1** (TIPSOTf (6 equiv.), 2,4,6-collidine, 90 °C, 45 h) (Scheme 2).

During the silylation of ethyl 1-thio-α-d- mannopyranoside (**8**), fully silylated mannopyranoside **10** in ^1^*C*_4_ conformation and partially silylated derivatives **11** and **12** in ^4^*C*_1_ conformation were obtained. On the contrary, silylation of ethyl 1-thio-β-d-mannopyranoside (**9**) led exclusively to the product of complete silylation **13** in 83% yield. We compared ^1^*C*_4_ (A) to ^4^*C*_1_ (B) ratio for ethyl 1-thio-α- and β-d-mannopyranosides (**10** and **13**) (Table 1, Table 2 and Table 3). We observed that silylated ethyl 1-thio-β-d-mannopyranoside **13** exists exclusively as a slightly distorted ^1^*C*_4_ conformer at 298 K and 240 K (*J*_H1–H2_ = 5.7 Hz), while for silylated ethyl 1-thio-α-d-mannopyranoside **10** the formation of the mixture of conformers is typical (^1^*C*_4_ (A) to ^4^*C*_1_ (B) ratio = 10/3.4 (240 K) with predominance of axially rich conformation ^1^*C*_4_.

### 2.3. A Model Reaction: Synthesis of 1,2-α-Linked Methyl Mannopyranoside ***16***

**Scheme 3:**
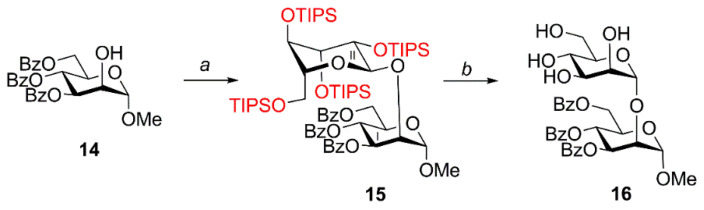
*Reagents and conditions*: *a*. **2**, TfOH, NIS, MS 4 Å, CH_2_Cl_2_, −60 → −30 °C, 2 h (58%); *b*. *n*-Bu_4_NF, AcOH, THF, 40 °C, 2 h (71%).

The reactivity of fully silylated phenyl 1-thio-α-d-mannopyranoside **2** was tested in a model reaction with methyl 3,4,6-tri-*O*-benzoyl-α-d-mannopyranoside (**14**) [41] under NIS/TfOH promotion (Scheme 3). After gel chromatography in toluene, dimannopyranoside **15** was obtained as a mixture of conformers. The ^1^*C*_4_ (A) to ^4^*C*_1_ (B) ratio of conformers for the monosaccharide residue at the non-reducing end of methyl dimannopyranoside **15** was 10/3 at 298 K and 10/7.6 at 240 K (Table 1). The anomeric protons for ^1^*C*_4_ (A) and ^4^*C*_1_ (B) conformers of the silylated residue of **15** (240 K) resonate at δ_H_ 4.98 (d, 1H, *J* 6.6 Hz, H-1^II^, A) and 4.68 (d, 1H, *J* 1.2 Hz, H-1^II^, B) ppm in ^1^H NMR spectra, correlating to the carbon signals at δ_C_ 99.7 (C-1^II^, A) and 102.2 (C-1^II^, B) ppm in ^13^C NMR spectra (Table 4 and Table 5). In addition to the characteristic high field position of C-1 (^1^*C*_4_ (A)) signals compared with low field position of C-1 (^4^C_1_ (B)) signals, the characteristic positions of C-3 for^1^*C*_4_ (A) and ^4^*C*_1_ (B) conformers in ^13^C NMR spectra could be noted. For example, we observed the carbon signals at δ_C_ 77.2 (C-3^II^, A) and 73.5 (C-3^II^, B) for **15** (240 K). The formation of an α-glycoside linkage in the dimannopyranoside **15** followed from ^1^H–^13^C HMBC NMR spectrum. We observed ^1^*J*_C1–H1_ = 171.2 Hz (240 K) for the major axially rich conformation ^1^*C*_4_ and ^1^*J*_C1–H1_ = 168.4 Hz for the minor ^4^*C*_1_ conformation. The TIPS groups in the resulting dimannopyranoside were removed (Bu_4_NF, AcOH, THF, 40 °C, 2 h) leading to the formation of disaccharide **16**, as expected, in the usual ^4^*C*_1_ conformation.

### 2.4. Synthesis of Thioglycoside of α-(1→2)-Linked Dimannopyranoside ***18*** with TIPS Protection at the Non-Reducing End

**Scheme 4:**
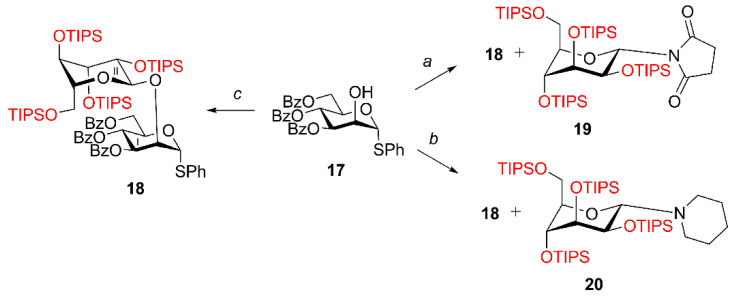
Synthesis of α-(1→2)-linked dimannopyranoside **18** with TIPS groups at the non-reducing end. *Reagents and conditions: a*. **2**, TfOH, NIS, CH_2_Cl_2_, MS 4 Å, −78 °C → −40 °C, **18**:**19** = 2.5:1 according to NMR data; *b*. **10**, TTBP, BSP, Tf_2_O, CH_2_Cl_2_, MS 4 Å, −78 → −50 °C, **18** (52%) containing 16 mol% of **19** (according to NMR data); *c*. **13**, TfOH, NIS, CH_2_Cl_2_, MS 4 Å, −78 → −50 °C, **18** (86%).

At the next step, glycosylation of phenyl 3,4,6-tri-*O*-benzoyl-1-thio-α-d-mannopyranoside (**17**) [42] with fully silylated α-configured thiomannopyranosides (**2**, **10**) and β-configured thiomannopyranoside **13**, was studied under various conditions (Scheme 4). The coupling of **17** with **2** with NIS/TfOH promotion resulted in the formation of a chromatographically inseparable mixture of the target α-(1→2)-linked dimannopyranoside **18** with silylated *N*-glycosylsuccinimide **19** in a ratio of **18**:**19** = 2.5:1 (NMR data). Glycosylation of thioglycoside **17** with silylated ethyl 1-thio-α-d-mannopyranoside **10** under preactivation conditions (benzenesulfenylpiperidine (BSP), 2,4,6-tri-*tert*-butylpyrimidine (TTBP), Tf_2_O) also gave a chromatographically inseparable mixture of α-(1→2)-linked dimannopyranoside **18** and piperidine-1-yl glycoside **20** (16 mol% according to ^1^H NMR data) (Scheme 4).

Glycosylation of phenyl 3,4,6-tri-*O*-benzoyl-1-thio-α-d-mannopyranoside (**17**) with silylated ethyl 1-thio-β-d-mannopyranoside **13** with NIS/TfOH promotion proved to be the best way to synthesize 1,2-α-linked dimannopyranoside **18**, obtained in 86% yield.

Based on these results, we can conclude that the optimal conditions for the preparation of the key 1,2-α-linked dimannopyranoside **18** were as follows: (1) an excess of the glycosyl acceptor; (2) temperature range from −78 to −50 °C; (3) NIS/TfOH promoting system. It should be noted that in all cases we observed the formation of only α-glycosydic linkage in **18**. Besides, the use of the silylated ethyl 1-thio-β-d-mannopyranoside **13** led to less side reactions and enabled simpler purification.

The dependence of the ^1^*C*_4_ (A) to ^4^*C*_1_ (B) ratio of conformers on nature of the aglycone in α-(1→2)-linked dimannopyranosides with *O*-methyl and *S*-phenyl aglycones (**15** and **18**) was also established. For methyl mannopyranoside **15** the portion of ^1^*C*_4_ (A) conformer is lower (10/3 (298 K); 10/7.6 (240 K)) than in the case of phenyl 1-thiomannopyranoside **18** (1/0 (303 K); 10/3.4 (240 K)) (Table 1). The anomeric protons for ^1^*C*_4_ (A) and ^4^*C*_1_ (B) conformers of silylated residues of **18** (240 K) resonated in the same region as for **15** at δ_H_ 4.96 (d, 1H, *J* 7.0 Hz, H-1^II^ A) and 4.72 (s, 1H, H-1^II^ B) ppm in ^1^H NMR spectra, correlating to the carbon signals at δ_C_ 99.7 (C-1^II^, A) and 102.9 (C-1^II^, B) ppm in ^13^C NMR spectra (Table 4 and Table 5). Besides, we observed the carbon signals δ_C_ 77.0 (2 C, C-3^II^, A; C-5^II^ B) and δ_C_ 73.5 (C-3^II^, B) for **18** (240 K) in the same region as for **15**.

### 2.5. Synthesis of Mannose-Capped Trisaccharide of the M. tuberculosis ManLAM

**Scheme 5:**
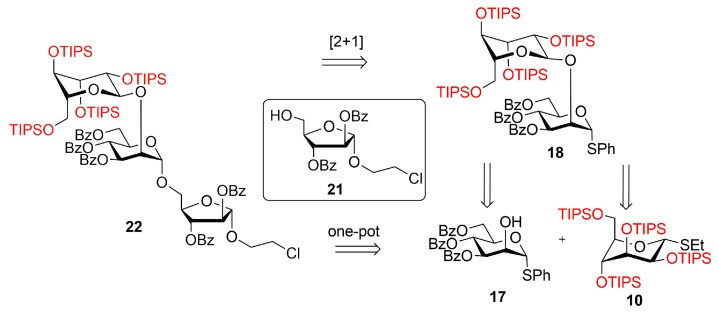
Retrosynthetic analysis of trisaccharide **22** bearing 2-chloroethyl aglycone.

**Scheme 6:**
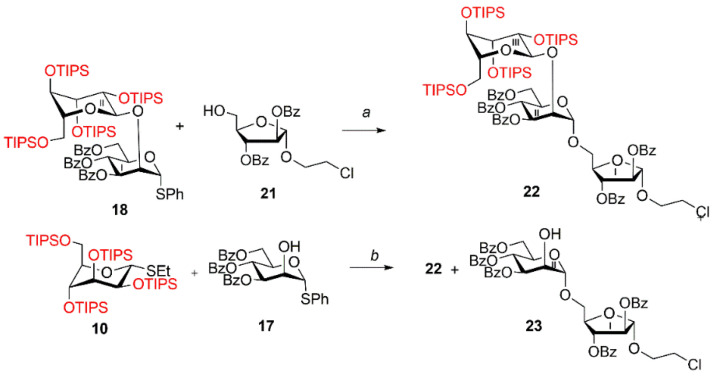
Synthesis of trisaccharide **22** bearing 2-chloroethyl aglycone. *Reagents and conditions*: *a*. TfOH, NIS, CH_2_Cl_2_, −10 °C → 0 °C (temperature raised during 1 h, kept at 0 °C for 16 h) (78%); *b.* (1) TfOH, NIS, CH_2_Cl_2_, −78 °C → −40 °C (temperature raised during 1 h) (2) **21**, TfOH, NIS, CH_2_Cl_2_, −40 °C → 0 °C (temperature raised during 1 h, kept at 0 °C for 23 h), **22** (49%), **23** (27%).

At the next step, we investigated the applicability of α-(1→2)-linked dimannopyranoside **18** with TIPS groups at the non-reducing end for the preparation of mannose-capped trisaccharide of the *M. tuberculosis* ManLAM. We studied two strategies to access protected trisaccharide **22** bearing 2-chloroethyl aglycone according to [2+1] convergent way and performing one-pot synthesis (Scheme 5 and Scheme 6).

To this end, the 2-chloroethyl arabinofuranoside **21** [43], synthesized by us earlier, was glycosylated with dimannothiopyranoside **18** under NIS/TfOH promotion to give trisaccharide **22** in 78% yield as a mixture of conformers according to NMR data (Scheme 6). The formation of α-glycoside linkage in the trisaccharide **22** followed from ^1^H–^13^C HMBC NMR spectrum. We observed ^1^*J*_C1–H1_ = 176.8 Hz (236 K) for the conformer A in the axially rich ^1^*C*_4_ conformation and ^1^*J*_C1–H1_ = 169.2 Hz for the minor conformer B in the ^4^*C*_1_ conformation.

Alternatively, based on the difference in reactivity of **10**, **17** and **21**, we performed one-pot synthesis of trisaccharide **22**. At the first step, we glycosylated phenyl 3,4,6-tri-*O*-benzoyl-1-thio-α-d-mannopyranoside **17** (1.2 equiv.) with fully silylated ethyl 1-thio-α-d-mannopyranoside **10** (1 equiv.) under NIS/TfOH promotion at −78 → −40 °C. Then 2-chloroethyl arabinofuranoside **21** was added followed by an additional portion of NIS/TfOH and the temperature was raised to 0 °C. Since the reaction was not complete after 20 h at 0 °C, an additional portion of arabinofuranoside **21** (2 equiv.) was added. The desired trisaccharide **22** was isolated in 49% yield (over two steps) along with partially protected trisaccharide with three TIPS groups (5%) and α-(1→5)-linked mannoarabinofuranoside **23** (27%). It is interesting to note that we observed the formation of only α-glycosidic linkages in the obtained mannoarabinofuranoside **23** despite the absence of a participating group at O-2 both in the monosaccharide mannosyl donor **17**.

**Scheme 7:**
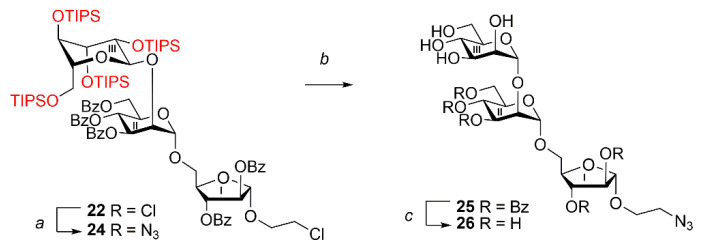
Synthesis of trisaccharide **26** bearing 2-azidoethoxy aglycone. *Reagents and conditions*: *a*. NaN_3_, 18-crown-6, DMF, 70 °C, 48 h (90%); *b*. *n*-Bu_4_NF, AcOH, THF, 40 °C, 3.5 h (60%); *c*. MeONa, MeOH, CH_2_Cl_2_, 20 °C, 18 h (78%).

Then we replaced the chlorine atom in the aglycone of the resulting trisaccharide **22** with azido group (NaN_3_, DMF, 18-crown-6) to give 2-azidoethyl glycoside **24** in 84% yield (Scheme 7). According to NMR data, the ^1^*C*_4_ (A) to ^4^*C*_1_ (B) ratios for trisaccharides **22** and **24** were 10/3 and 10/8.6 at 303 K and 236 (244) K, respectively (Table 1). We found the signals of the anomeric protons of **22** for conformer A at δ_H_ 5.06 (d, 1H, *J* 6.6 Hz, H-1^III^), 5.09 (d, 1H, *J* 1.8 Hz, H-1^II^), 5.14 (s, 1H, H-1^I^) in the ^1^H NMR spectrum, correlating with the carbon signals at δ_C_ 99.9 (C-1^III^), 100.11 (C-1^II^), 105.87 (C-1^I^) at 303 K. The signals of the anomeric protons of **22** for conformer B were found at δ_H_ 4.79 (s, 1H, H-1^III^), 5.18 (s, 1H, H-1^I^), 5.27 (s, 1H, H-1^II^), correlating with the carbon signals at δ_C_ 98.5 (C-1^II^), 102.5 (C-1^III^), 105.9 (C-1^I^) at 303 K (Table 4 and Table 5).

Then all TIPS groups in azide **24** were removed by treatment with TBAF in THF in the presence of AcOH at 40 °C to give partially protected trisaccharide **25** as expected, in the usual ^4^*C*_1_ conformation. Debenzoylation with MeONa in MeOH led the target deprotected 2-azidoethyl 5-*O*-[α-d-mannopyranosyl-2-*O*-(α-d-mannopyranosyl)-α-d-arabinofuranoside (**26**) in 78% yield (Scheme 7).

## 3. Materials and Methods

### 3.1. General Methods

All reactions sensitive to air and/or moisture were carried out under argon atmosphere. The reactions were performed with the use of commercial reagents (Aldrich (St. Louis, MO, USA), Fluka (Waltham, MA, USA), Acros Organics (Geel, Belgium)). Anhydrous solvents were purified and dried (where appropriate) according to standard procedures [44]. Dichloromethane was distilled over P_2_O_5_ and then over CaH_2_ and stored over 4 Å molecular sieves (MS 4 Å). Powdered MS 4 Å (Fluka, Seelze, Germany) were activated before glycosylation reactions by heating at 220 °C in high vacuum (0.2 mbar) for 6 h. Column chromatography was performed on silica gel 60 (40–63 μm, Merck, Darmstadt, Germany). Thin-layer chromatography was carried out on plates with silica gel 60 on aluminum foil (Merck). Spots of compounds were visualized under UV light (254 nm) and by heating the plates (at ca. 150 °C) after immersion in a 1:10 (*v*/*v*) mixture of 85% aqueous H_3_PO_4_ and 95% EtOH. Gel permeation chromatography was performed on a 400 × 20 mm column packed with Bio-Beads S-X3 (200–400 mesh, Bio-Rad, Hercules, CA, USA) or on a 450 × 30 mm column packed with Bio-Beads S-X1 (200–400 mesh). A procedure for “co-evaporation” with toluene involved addition of toluene and evaporation of volatiles on a rotary evaporator. ^1^H, ^13^C and ^29^Si NMR spectra were recorded on a Bruker AVANCE NEO 300 spectrometer (Billerica, MA, USA, 300.23, 75.50 and 59.65 MHz for ^1^H, ^13^C and ^29^Si, respectively) or on a Bruker AVANCE 600 spectrometer (Billerica, MA, USA, 600.13, 150.92 and 119 MHz for ^1^H, ^13^C and ^29^Si respectively).The ^1^H NMR chemical shifts are referred to the residual signal of CHCl_3_ (δ_H_ 7.27 ppm) for solutions in CDCl_3_, C_6_HD_5_ (δ_H_ 7.16 ppm) for solutions in C_6_D_6_ or CHD_2_OD (δ_H_ 3.31 ppm) for solutions in CD_3_OD, the ^13^C NMR shifts–to the central line of CDCl_3_ signal (δ_C_ 77.00 ppm), C_6_D_6_ (δ_C_ 128.06 ppm) or CD_3_OD signal (δ_C_ 49.00 ppm).The ^29^Si chemical shifts are given relative to the signal of external Me_4_Si (δ_Si_ 0.00 ppm). Assignments of the signals in the NMR spectra were performed using ^1^H–^1^H and ^1^H–^13^C 2D-spectroscopy (COSY, HSQC, HMBC). Position of silyl groups was determined from ^1^H–^29^Si HMBC experiments. High resolution mass spectra (HRMS, electrospray ionization (ESI)) were recorded in a positive ion mode on Bruker micrOTOF II or maXis mass spectrometers for 2 · 10^−5^ M solutions in MeCN. Optical rotations were measured using a JASCO P-2000 automatic digital polarimeter (Hachioji, Japan).

### 3.2. Silylation of Phenyl 1-thio-α-d-Mannopyranoside (***1***): Phenyl 2,3,4,6-Tetrakis-O-(Triisopropylsilyl)-1-thio-α-d-mannopyranoside (***2***), Phenyl 2, 3,6-tris-O-(Triisopropylsilyl)-1-thio-α-d-mannopyranoside (***3***), Phenyl 2,4,6-tris-O-(Triisopropylsilyl)-1-thio-α-d-mannopyranoside (***4***), Phenyl 3,4,6-tris-O-(Triisopropylsilyl)-1-thio-α-d-mannopyranoside (***5***), Phenyl 3,6-bis-O-(Triisopropylsilyl)-1-thio-α-d-mannopyranoside (***6***)

(1) *i*-Pr_3_SiOTf (0.630 mL, 2.34 mmol) was added to the solution of tetraol **1** (106 mg, 0.389 mmol) in 2,4,6-collidine (1.5 mL) at 20 °C. The reaction mixture was stirred at 90 °C for 45 h and then allowed to reach 20 °C, diluted with CH_2_Cl_2_ (40 mL), washed with H_2_O (40 mL), 1 M KHSO_4_ (3 × 40 mL), H_2_O (40 mL), and satd aq NaHCO_3_ (3 × 40 mL). Organic extracts were dried with Na_2_SO_4_, filtered, concentrated under reduced pressure, and purified by silica gel chromatography (gradient: 0 → 24% EtOAc in petroleum ether) to give silylated derivatives **2**–**5**.

Data for phenyl 2,3,4,6-tetrakis-*O*-(triisopropylsilyl)-1-thio-α-d-mannopyranoside (**2**):

219 mg (0.244 mmol, 63%), *R*_f_ = 0.73 (petroleum ether–CH_2_Cl_2_, 4:1), [α]_D_^21^ + 99.7 (c 0.90, CHCl_3_); ^1^H NMR (600 MHz, CDCl_3_, 323 K): δ 1.01–1.34 (m, 84H, 4 × ((CH_3_)_2_CH)_3_Si), 3.86 (dd, 1H, *J* 10.7 Hz, *J* 7.6 Hz, H-6a), 3.90 (s, 1H, H-4), 3.92–3.97 (m, 1H, H-6b), 3.96–4.01 (m, 1H, H-5), 4.15–4.19 (m, 1H, H-3), 4.20–4.24 (m, 1H, H-2), 5.40 (d, 1H, *J* 7.7 Hz, H-1), 7.16–7.21 (m, 1H, PhS (H-4)), 7.21–7.25 (m, 2H, PhS (H-3, H-5)), 7.55–7.59 (m, 2H, PhS (H-2, H-6)); ^13^C NMR (151 MHz, CDCl_3_): δ 12.1 (((CH_3_)_2_CH)_3_Si), 13.0 (((CH_3_)_2_CH)_3_Si), 13.2 (((CH_3_)_2_CH)_3_Si), 13.6 (((CH_3_)_2_CH)_3_Si), 17.9 (((CH_3_)_2_CH)_3_Si), 18.0 (((CH_3_)_2_CH)_3_Si), 18.2 (((CH_3_)_2_CH)_3_Si), 18.3 (((CH_3_)_2_CH)_3_Si), 18.4 (((CH_3_)_2_CH)_3_Si), 18.51 (((CH_3_)_2_CH)_3_Si), 18.53 (((CH_3_)_2_CH)_3_Si), 65.1 (C-6), 72.3 (C-2), 73.1 (C-4), 77.3 (C-3), 77.7 (C-5), 88.6 (C-1), 126.3 (PhS (C-4)), 128.4 (PhS (C-3, C-5)), 131.1 (PhS (C-2, C-6)).

^1^H NMR (600 MHz, CDCl_3_, 298 K): δ 0.89–1.38 (m, 84H, 4 × ((CH_3_)_2_CH)_3_Si), 3.85 (dd, 1H, *J* 10.7 Hz, *J* 7.6 Hz, H-6a), 3.84–3.90 (m, 1H, H-4), 3.90–3.94 (m, 1H, H-6b), 3.94–4.01 (m, 1H, H-5), 4.11–4.16 (m, 1H, H-3), 4.17–4.24 (m, 1H, H-2), 5.38 (d, 1H, *J* 7.6 Hz, H-1), 7.17–7.21 (m, 1H, PhS (H-4)), 7.21–7.26 (m, 2H, PhS (H-3, H-5)), 7.55–7.61 (m, 2H, PhS (H-2, H-6)); ^13^C NMR (151 MHz, CDCl_3_): δ 11.9 (((CH_3_)_2_CH)_3_Si), 12.7 (((CH_3_)_2_CH)_3_Si), 13.0 (((CH_3_)_2_CH)_3_Si), 13.3 (((CH_3_)_2_CH)_3_Si), 17.89 (((CH_3_)_2_CH)_3_Si), 17.94 (((CH_3_)_2_CH)_3_Si), 18.20 (((CH_3_)_2_CH)_3_Si), 18.24 (((CH_3_)_2_CH)_3_Si), 18.4 (((CH_3_)_2_CH)_3_Si), 18.45 (((CH_3_)_2_CH)_3_Si), 18.49 (((CH_3_)_2_CH)_3_Si), 64.7 (C-6), 71.8 (C-2), 72.9 (C-4), 77.1 (C-3), 77.6 (C-5), 88.3 (C-1), 126.3 (PhS (C-4)), 128.4 (PhS (C-3, C-5)), 131.1 (PhS (C-2, C-6));

^1^H NMR (600 MHz, CDCl_3_, 243 K, A:B = 10:1.6): δ 0.97–1.16 (m, 84H, ((CH_3_)_2_CH)_3_Si), 1.26 (p, 3H, *J* 7.5 Hz, ((CH_3_)_2_CH)_3_Si), 3.77 (dd, 1H, *J* 10.0 Hz, *J* 8.5 Hz, H-6a, B), 3.82–3.87 (m, 2H, H-4, A, H-6a, A), 3.88 (dd, 1H, *J* 10.8 Hz, *J* 7.4 Hz, H-6b, A), 3.94 (ddd, 1H, *J* 7.8 Hz, *J* 4.6 Hz, *J* 3.2 Hz, H-5, A), 3.94–3.97 (m, 1H, H-3, B), 4.04 (d, 1H, *J* 9.7 Hz, H-6b, B), 4.06–4.09 (m, 1H, H-4, B), 4.09 (d, 1H, *J* 2.2 Hz, H-3, A), 4.11–4.14 (m, 1H, H-5, B), 4.14 (dd, 1H, *J* 8.4 Hz, *J* 2.0 Hz, H-2, A), 4.33 (t, 1H, *J* 1.8 Hz, H-2, B), 5.34–5.35 (m, 1H, H-1, B), 5.35 (d, 1H, *J* 8.5 Hz, H-1, A), 7.18–7.24 (m, 1H, Ph (H-4)), 7.22–7.28 (m, 3H, Ph (H-3, H-5)), 7.54–7.59 (m, 1H, Ph (H-2, H-6), B), 7.57–7.62 (m, 2H, Ph (H-2, H-6), A); ^13^C NMR (151 MHz, CDCl_3_): δ 11.3 (((CH_3_)_2_CH)_3_Si, B), 11.5 (((CH_3_)_2_CH)_3_Si, A), 12.2 (((CH_3_)_2_CH)_3_Si, A), 12.6 (((CH_3_)_2_CH)_3_Si, A), 12.9 (((CH_3_)_2_CH)_3_Si, B), 13.1 (((CH_3_)_2_CH)_3_Si, A), 13.9 (((CH_3_)_2_CH)_3_Si, B), 14.2 (((CH_3_)_2_CH)_3_Si, B), 17.7 (((CH_3_)_2_CH)_3_Si, B), 17.79 (((CH_3_)_2_CH)_3_Si, A), 17.81 (((CH_3_)_2_CH)_3_Si, B), 17.9 (((CH_3_)_2_CH)_3_Si, A), 18.0 (((CH_3_)_2_CH)_3_Si, A), 18.08 (((CH_3_)_2_CH)_3_Si, A), 18.14 (((CH_3_)_2_CH)_3_Si, B), 18.2 (((CH_3_)_2_CH)_3_Si, A), 18.27 (((CH_3_)_2_CH)_3_Si, A), 18.31 (((CH_3_)_2_CH)_3_Si, A), 18.4 (((CH_3_)_2_CH)_3_Si, A), 18.6 (((CH_3_)_2_CH)_3_Si, B), 18.7 (((CH_3_)_2_CH)_3_Si, B), 63.7 (C-6, A), 63.8 (C-6, B), 69.9 (C-4, B), 71.1 (C-2, A), 72.3 (C-4, A), 74.9 (C-3, B), 75.6 (C-2, B), 76.5 (C-3, A), 76.6 (C-5, B), 77.9 (C-5, A), 87.4 (C-1, A), 88.9 (C-1, B), 126.2 (PhS (C-4), A), 126.7 (PhS (C-4), B), 128.3 (PhS (C-3, C-5), A), 128.6 (PhS (C-3, C-5), B), 130.7 (PhS (C-2, C-6), A), 131.7 (PhS (C-2, C-6), B), 135.2 (PhS (C-1), B), 136.4 (PhS (C-1), A);

^1^H NMR (300 MHz, C_6_D_6_): δ 0.93–1.54 (m, 84H, ((CH_3_)_2_CH)_3_Si), 4.03–4.25 (m, 4H, H-4, H-6), 4.28–4.39 (m, 1H, H-5), 4.41 (d, 1H, *J* 2.4 Hz, H-3), 4.49–4.57 (m, 1H, H-2), 5.77 (d, 1H, *J* 7.3 Hz, H-1), 6.91–7.04 (m, 1H, Ph (H-4)), 7.06–7.12 (m, 2H, Ph (H-3, H-5)), 7.61–8.09 (m, 2H, Ph (H-2, H-6));selected signals ^13^C NMR (76 MHz, C_6_D_6_): δ 12.4 (((CH_3_)_2_CH)_3_Si), 13.3 (((CH_3_)_2_CH)_3_Si), 13.6 (((CH_3_)_2_CH)_3_Si), 13.8 ((CH_3_)_2_CH)_3_Si)), 18.25 (((CH_3_)_2_CH)_3_Si), 18.29 (((CH_3_)_2_CH)_3_Si), 18.5 (((CH_3_)_2_CH)_3_Si), 18.6 (((CH_3_)_2_CH)_3_Si), 18.8 (((CH_3_)_2_CH)_3_Si), 18.8 (((CH_3_)_2_CH)_3_Si), 18.9 (((CH_3_)_2_CH)_3_Si), 65.6 (C-6), 77.70, 89.5 (C-1), 126.9 (PhS (C-4)), 129.0 (PhS (C-3, C-5)), 131.3 (PhS (C-2, C-6)), 136.6 (PhS (C-1));

Data for phenyl 2,3,6-tris-*O*-(triisopropylsilyl)-1-thio-α-d-mannopyranoside (**3**): 45.4 mg (0.061 mmol, 16%), *R_f_* = 0.37 (petroleum ether–CH_2_Cl_2_, 4:1). [α]_D_^22^ +58.7 (c 1.13, CHCl_3_); ^1^H NMR (300 MHz, C_6_D_6_): δ 1.01–1.39 (m, 63H, ((CH_3_)_2_CH)_3_Si), 2.77 (d, 1H, *J* 2.2 Hz, OH), 4.11 (dd, 1H, *J* 10.2 Hz, *J* 5.7 Hz, H-6a), 4.17 (dd, 1H, *J* 10.2 Hz, *J* 5.1 Hz, H-6b), 4.29 (td, 1H, *J* 9.0 Hz, *J* 2.2 Hz, H-4), 4.37 (dd, 1H, *J* 9.0 Hz, *J* 2.5 Hz, H-3), 4.43–4.52 (m, 1H, H-5), 4.66 (dd, 1H, *J* 2.5 Hz, *J* 1.8 Hz, H-2), 5.75 (dd, 1H, *J* 1.8 Hz, *J* 0.7 Hz, H-1), 6.89–6.97 (m, 1H, Ph (H-4)), 7.00–7.09 (m, 2H, Ph (H-3, H-5)), 7.59–7.68 (m, 2H, Ph (H-2, H-6)); ^13^C NMR (76 MHz, C_6_D_6_): δ 12.2 (((CH_3_)_2_CH)_3_Si), 13.3 (((CH_3_)_2_CH)_3_Si), 13.4 (((CH_3_)_2_CH)_3_Si), 18.2 (((CH_3_)_2_CH)_3_Si), 18.45 (((CH_3_)_2_CH)_3_Si), 18.50 (((CH_3_)_2_CH)_3_Si), 18.6 (((CH_3_)_2_CH)_3_Si), 18.7 (((CH_3_)_2_CH)_3_Si), 66.0 (C-6), 70.6 (C-4), 74.5 (C-5), 74.9 (C-3), 75.7 (C-2), 90.6 (C-1), 127.6 (PhS (C-4)), 129.4 (PhS (C-3, C-5)), 131.7 (PhS (C-2, C-6)), 135.7 (PhS (C-1)); ^29^Si INEPT NMR (60 MHz, C_6_D_6_): δ 12.6 (2-*O*-TIPS), 14.1 (3-*O*-TIPS), 15.0 (6-*O*-TIPS); HRMS (ESI): *m*/*z* [M+NH_4_]^+^ Calcd for C_48_H_100_NO_5_SSi_4_ 914.6394; Found: 914.6393.

Data for phenyl 2,4,6-tris-*O*-(triisopropylsilyl)-1-thio-α-d-mannopyranoside (**4**): 28.1 mg (0.038 mmol, 10%) *R*_f_ = 0.26 (petroleum ether–CH_2_Cl_2_, 4:1). [α]_D_^22^ +78.4 (c 1.32, CHCl_3_); ^1^H NMR (300 MHz, C_6_D_6_): δ 0.89–1.47 (m, 63H, 3 × ((CH_3_)_2_CH)_3_Si), 2.25 (d, 1H, *J* 9.8 Hz, 3-HO), 4.00 (ddd, 1H, *J* 9.8 Hz, *J* 8.2 Hz, *J* 3.4 Hz, H-3), 4.11 (dd, 1H, *J* 10.7 Hz, *J* 2.5 Hz, H-6a), 4.16 (dd, 1H, *J* 10.8 Hz, *J* 4.2 Hz, H-6b), 4.27 (dd, 1H, *J* 9.3 Hz, *J* 8.1 Hz, H-4), 4.34 (ddd, 1H, *J* 9.1 Hz, *J* 4.3 Hz, *J* 2.5 Hz, H-5), 4.47 (dd, 1H, *J* 3.4 Hz, *J* 1.6 Hz, H-2), 5.74 (d, 1H, *J* 1.6 Hz, H-1), 6.92–7.02 (m, 1H, PhS (H-4)), 7.05–7.14 (m, 2H, PhS (H-3, H-5)), 7.59–7.67 (m, 2H, PhS (H-2, H-6)); ^13^C NMR (76 MHz, C_6_D_6_): δ 12.4 (((CH_3_)_2_CH)_3_Si), 12.8 (((CH_3_)_2_CH)_3_Si), 13.6 (((CH_3_)_2_CH)_3_Si), 18.16 (((CH_3_)_2_CH)_3_Si), 18.18 (((CH_3_)_2_CH)_3_Si), 18.31 (((CH_3_)_2_CH)_3_Si), 18.34 (((CH_3_)_2_CH)_3_Si), 18.82 (((CH_3_)_2_CH)_3_Si), 18.85 (((CH_3_)_2_CH)_3_Si), 63.6 (C-6), 71.0 (C-4), 73.9 (C-3), 74.8 (C-2), 75.5 (C-5), 88.7 (C-1), 127.4 (PhS (C-4)), 129.3 (PhS (C-3, C-5)), 131.4 (PhS (C-2, C-6)), 135.7 (PhS (C-1)); ^29^Si INEPT NMR (60 MHz, C_6_D_6_): δ 12.2 (4-*O*-TIPS), 13.3 (6-*O*-TIPS), 15.5 (2-*O*-TIPS); HRMS (ESI): *m*/*z* [M+NH_4_]^+^ Calcd for C_39_H_80_NO_5_SSi_3_ 758.5060; Found: 758.5052.

Data for phenyl 3,4,6-tris-*O*-(triisopropylsilyl)-1-thio-α-d-mannopyranoside (**5**): 25 mg (0.034 mmol, 9%), *R_f_* = 0.17 (petroleum ether–CH_2_Cl_2_, 4:1). [α]_D_^22^ +30.3 (c 1.22, CHCl_3_); ^1^H NMR (600 MHz, CDCl_3_): δ 0.99–1.21 (m, 63H, 3 × ((CH_3_)_2_CH)_3_Si), 2.16 (d, 1H, *J* 4.9 Hz, OH), 3.83 (ddd, 1H, *J* 9.3 Hz, *J* 4.9 Hz, *J* 2.9 Hz, H-2), 3.99 (dd, 1H, *J* 12.4 Hz, *J* 5.8 Hz, H-6a), 3.97–4.01 (m, 1H, H-5), 4.08 (dd, 1H, *J* 3.8 Hz, *J* 1.6 Hz, H-4), 4.08 (ddd, 1H, *J* 12.4 Hz, *J* 8.8 Hz, H-6b), 4.23 (t, 1H, *J* 3.3 Hz, H-3), 5.06 (d, 1H, *J* 9.3 Hz, H-1), 7.20–7.28 (m, 3H, PhS (H-3, H-4, H-5)), 7.58–7.63 (m, 2H, PhS (H-2, H-6)); ^13^C NMR (151 MHz, CDCl_3_): δ 12.0 (((CH_3_)_2_CH)_3_Si), 12.4 (((CH_3_)_2_CH)_3_Si), 12.6 (((CH_3_)_2_CH)_3_Si), 18.0 (2 × ((CH_3_)_2_CH)_3_Si), 18.09 (((CH_3_)_2_CH)_3_Si), 18.11 (((CH_3_)_2_CH)_3_Si), 18.16 (((CH_3_)_2_CH)_3_Si), 18.24 (((CH_3_)_2_CH)_3_Si), 61.9 (C-6), 67.0 (C-2), 71.1 (C-4), 73.5 (C-3), 82.0 (C-5), 82.2 (C-1), 127.4 (PhS (C-4)), 128.7 (PhS (C-3, C-5)), 132.6 (PhS (C-1)), 132.5 (PhS (C-2, C-6)); ^29^Si INEPT NMR (119 MHz, CDCl_3_): δ 13.7 (4-*O*-TIPS), 14.0 (6-*O*-TIPS), 15.2 (3-*O*-TIPS); HRMS (ESI): *m*/*z* [M+NH_4_]^+^ Calcd for C_39_H_80_NO_5_SSi_3_ 758.5060; Found: 758.5058.

(2) To the solution of tetraol **1** (100 mg, 0.367 mmol) in DMF (2 mL) imidazole (300 mg, 4.41 mmol) and TIPSCl (0.471 mL, 2.2 mmol) were added. The reaction mixture was stirred at 90 °C for 52 h, then co-evaporated with toluene (3 × 10 mL). The residue was diluted with CH_2_Cl_2_ (50 mL), washed with H_2_O (50 mL), 1 M KHSO_4_ (3 × 30 mL), H_2_O (30 mL), satd aq NaHCO_3_ (3 × 30 mL), concentrated and dried in vacuo. After purification by silica gel chromatography (gradient: 0 → 24% CH_2_Cl_2_ in petroleum ether) silylated derivatives **3** (60.0 mg, 0.081 mmol, 21%), **4** (70.0 mg, 0.094 mmol, 22%), **5** (56 mg, 0.076 mmol, 21%), **6** (55 mg, 0.094 mmol, 26%) were obtained.

(3) To the solution of tetraol **1** (136 mg, 0.5 mmol) in DMF (2 mL) imidazole (170 mg, 2.5 mmol) and TIPSCl (0.234 mL, 1.1 mmol) were added. The reaction mixture was stirred at 20 °C for 96 h, then co-evaporated with toluene (3 × 10 mL). The residue was diluted with CH_2_Cl_2_ (50 mL), washed with H_2_O (50 mL), 1 M KHSO_4_ (3 × 30 mL), H_2_O (30 mL), satd aq NaHCO_3_ (3 × 30 mL), concentrated and dried in vacuo. After purification by silica gel chromatography (gradient: 2 → 10% EtOAc in petroleum ether) silylated derivative **6** (250 mg, 0.427 mmol, 85%) was obtained; *R_f_* = 0.75 (petroleum ether–EtOAc, 4:1). [α]_D_^26^ +77.3 (c 1.15, CHCl_3_); ^1^H NMR (300 MHz, CDCl_3_): δ 1.01–1.28 (m, 42H, 2 × ((CH_3_)_2_CH)_3_Si), 2.81 (d, 1H, *J* 1.4 Hz, OH-2), 2.99 (d, 1H, *J* 1.9 Hz, OH-4), 3.87 (td, 1H, *J* 9.0 Hz, *J* 1.8 Hz, H-4), 3.93–3.97 (m, 2H, H-6a, H-6b), 4.05 (dd, 1H, *J* 8.6 Hz, *J* 3.4 Hz, H-3), 4.10–4.19 (m, 2H, H-2, H-5), 5.59 (d, 1H, *J* 1.4 Hz, H-1), 7.24–7.33 (m, 3H, SPh (H-3, H-4, H-5)), 7.46–7.51 (m, 3H, SPh (H-2, H-6)); ^13^C NMR (75 MHz, CDCl_3_): δ 11.8 (((CH_3_)_2_CH)_3_Si), 12.5 (((CH_3_)_2_CH)_3_Si), 17.88 (4 C, ((CH_3_)_2_CH)_3_Si), 17.93–18.18 (4 C, ((CH_3_)_2_CH)_3_Si), 65.6 (C-6), 71.5 (C-4), 71.7 (C-5), 72.6 (C-2), 73.4 (C-3), 87.1 (C-1), 127.3 (SPh (C-4)), 129.0 (SPh (C-3, C-5)), 131.5 (SPh (C-2, C-6)); HRMS (ESI): *m*/*z* [M+Na]^+^Calcd for C_30_H_56_NaO_5_SSi_2_^+^ 607.3279; Found: 607.3282;

### 3.3. Phenyl 3,6-bis-O-(Triisopropylsilyl)-2,4-bis-O-(Triethylsilyl)-1-thio-α-d-mannopyranoside (***7***)

TESOTf (0.135 μL, 0.6 mmol) was added to the solution of diol **6** (116.6 mg, 0.2 mmol) in 2,4,6-collidine (2 mL) at 20 °C. The reaction mixture was stirred at 20 °C for 2 h, then diluted with CH_2_Cl_2_ (50 mL), washed with H_2_O (50 mL), satd aq NaHCO_3_ (50 mL), concentrated and dried in vacuo. After purification by silica gel chromatography (gradient: 1 → 6% EtOAc in petroleum ether) silylated derivative **7** (126 mg, 0.155 mmol, 78%) was obtained. *R*_f_ = 0.50 (petroleum ether–CH_2_Cl_2_, 2:1); [α]_D_^20^ +9.4 (c 0.99, CHCl_3_); ^1^H NMR (600 MHz, CDCl_3_, 303 K, A:B = 10:0.14) Signals were too broad for full assignment. Selected signals: δ 5.23 (br. s, 1H, H-1, B), 5.26 (d, 1H, *J* 6.3 Hz, H-1, A);^1^H NMR (600 MHz, CDCl_3_, 240 K, A:B = 1:1): δ 0.52–0.62 (m, 12H, 2 × (CH_3_CH_2_)_3_Si), 0.62–0.76 (m, 12H, 2 × (CH_3_CH_2_)_3_Si), 0.88–0.98 (m, 36H, 2 × (CH_3_CH_2_)_3_Si A, 2 × (CH_3_CH_2_)_3_Si B), 0.99–1.17 (m, 84H, 2 × ((CH_3_)_2_CH)_3_Si A, 2 × ((CH_3_)_2_CH)_3_Si B), 3.77–3.86 (m, 5H, H-6a B, H-4 A, H-5 A, H-6a A, H-6b A), 3.91–3.94 (m, 2H, H-3 B, H-3 A), 3.94–3.96 (m, 1H, H-6b B), 3.98 (dd, 1H, *J* 8.7 Hz, *J* 2.2 Hz, H-2 A), 3.99–4.03 (m, 1H, H-5 B), 4.05 (dd, 1H, *J* 9.3 Hz, *J* 8.6 Hz, H-4 B), 4.21 (t, 1H, *J* 1.9 Hz, H-2 B), 5.31 (d, 1H, *J* 8.8 Hz, H-1 A), 5.32 (d, 1H, *J* 1.4 Hz, H-1 B), 7.18–7.33 (m, 6H, PhS (H-4) A, PhS (H-4) B, PhS (H-3, H-5) A, PhS (H-3, H-5) B), 7.51–7.55 (m, 2H, PhS (H-2, H-6) B), 7.55–7.60 (m, 2H, PhS (H-2, H-6) A); ^13^C NMR (151 MHz, CDCl_3_): δ 4.4 ((CH_3_CH_2_)_3_Si), 4.6 ((CH_3_CH_2_)_3_Si), 4.8 ((CH_3_CH_2_)_3_Si), 5.1 ((CH_3_CH_2_)_3_Si), 6.90 ((CH_3_CH_2_)_3_Si), 6.92 ((CH_3_CH_2_)_3_Si), 6.7 ((CH_3_CH_2_)_3_Si), 7.1 ((CH_3_CH_2_)_3_Si), 11.49 (((CH_3_)_2_CH)_3_Si), 11.51 (((CH_3_)_2_CH)_3_Si), 12.4 (((CH_3_)_2_CH)_3_Si), 13.4 (((CH_3_)_2_CH)_3_Si), 17.8 (2 × ((CH_3_)_2_CH)_3_Si), 17.7 (2 × ((CH_3_)_2_CH)_3_Si), 18.18 (((CH_3_)_2_CH)_3_Si), 18.23 (((CH_3_)_2_CH)_3_Si), 18.33 (((CH_3_)_2_CH)_3_Si), 18.34 (((CH_3_)_2_CH)_3_Si), 62.9 (C-6 A, C-6 B), 63.0 (C-6 A, C-6 B), 68.7 (C-4 B), 70.2 (C-2 A), 71.2 (C-4 A), 74.6 (C-3 B), 75.0 (C-2 B), 75.9 (C-5 B), 76.2 (C-3 A), 78.3 (C-5 A), 86.0 (C-1 A), 88.8 (C-1 B), 126.2 (PhS (C-4) A), 126.6 (PhS (C-4) B), 128.5 (PhS (C-3, C-5) A, PhS (C-3, C-5) B), 128.6 (PhS (C-3, C-5) A, PhS (C-3, C-5) B), 130.4 (PhS (C-2, C-6) A), 130.7 (PhS (C-2, C-6) B), 135.6 (PhS (C-1) B), 136.1 (PhS (C-1) A); HRMS (ESI): *m*/*z* [M+Na]^+^Calcd for C_42_H_84_NaO_5_SSi_4_^+^ 835.5009; Found: 835.5009.

### 3.4. Silylation of Ethyl 1-thio-α-and β-d-Mannopyranosides (***8*** and ***9***): Ethyl 2,3,4,6-Tetrakis-O-(Triisopropylsilyl)-1-thio-α-d-mannopyranoside (***10***), Ethyl 2,3,6-tris-O-(Triisopropylsilyl)-1-thio-α-d-mannopyranoside (***11***) and Ethyl 2,4,6-tris-O-(Triisopropylsilyl)-1-thio-α-d-mannopyranoside (***12***)

*i*-Pr_3_SiOTf (0.720 mL, 2.68 mmol) was added to the solution of ethyl 1-thio-α-d-mannopyranoside **8** (108 mg, 0.483 mmol) in 2,4,6-collidine (1.5 mL) at 20 °C. The reaction mixture was stirred at 90 °C for 45 h and then allowed to reach 20 °C, diluted with CH_2_Cl_2_ (40 mL), washed with H_2_O (40 mL), 1 M KHSO_4_ (3 × 40 mL), H_2_O (40 mL) and satd aq NaHCO_3_ (3 × 40 mL). Organic extracts were dried with Na_2_SO_4_, filtered, concentrated under reduced pressure and purified by silica gel chromatography (gradient: 10 → 22% CH_2_Cl_2_ in petroleum ether) to give silylated derivatives **10**–**12**.

Data for ethyl 2,3,4,6-tetrakis-*O*-(triisopropylsilyl)-1-thio-α-d-mannopyranoside (**10**): 208 mg (0.254 mmol, 51%) *R*_f_ = 0.69 (petroleum ether–CH_2_Cl_2_, 2:1). [α]_D_^20^ +58.7 (c 1.02, CHCl_3_); ^1^H NMR (600 MHz, CDCl_3_, 240 K, A:B = 10:3.4): δ 0.98–1.13 (m, 168H, 4 × ((CH_3_)_2_CH)_3_Si, A&, B), 1.19 (q, 3H, *J* 7.3 Hz, ((CH_3_)_2_CH)_3_Si, A), 1.23 (t, 3H, *J* 7.4 Hz, CH_3_CH_2_S, B), 1.25 (t, 3H, *J* 7.4 Hz, CH_3_CH_2_S, A), 2.51 (dq, 1H, *J* 13.3 Hz, *J* 7.6 Hz, CH_3_CHHS, B), 2.57 (dq, 1H, *J* 13.0 Hz, *J* 7.5 Hz, CH_3_CHHS, A), 2.71 (dq, 1H, *J* 13.0 Hz, *J* 7.2 Hz, CH_3_CHHS, B), 2.76 (dq, 1H, *J* 13.0 Hz, *J* 7.3 Hz, CH_3_CHHS, A), 3.70 (dd, 1H, *J* 10.1 Hz, *J* 8.7 Hz, H-6a, B), 3.79–3.85 (m, 3H, H-4, A, H-5, A, H-6a, A), 3.87 (dd, 1H, *J* 11.2 Hz, *J* 1.9 Hz, H-6b, A), 3.93 (dp, 1H, *J* 10.6 Hz, *J* 5.8 Hz, H-5, B), 4.00 (dd, 1H, *J* 10.1 Hz, *J* 1.8 Hz, H-6b, B), 4.01–4.05 (m, 2H, H-3, B, H-4, B), 4.07 (t, 1H, *J* 2.3 Hz, H-3, A), 4.08 (dd, 1H, *J* 8.5 Hz, *J* 1.9 Hz, H-2, A), 4.14 (t, 1H, *J* 1.5 Hz, H-2, B), 5.12 (d, 1H, *J* 8.5 Hz, H-1, A), 5.19 (d, 1H, *J* 1.4 Hz, H-1, B); ^13^C NMR (151 MHz, CDCl_3_): δ 11.4 (((CH_3_)_2_CH)_3_Si, B), 11.5 (((CH_3_)_2_CH)_3_Si, A), 12.1 (((CH_3_)_2_CH)_3_Si, A), 12.6 (((CH_3_)_2_CH)_3_Si, A), 13.0 (((CH_3_)_2_CH)_3_Si, B), 13.1 (((CH_3_)_2_CH)_3_Si, A), 13.7 (((CH_3_)_2_CH)_3_Si, B), 14.1 (((CH_3_)_2_CH)_3_Si, B), 14.4 (CH_3_CH_2_S, B), 14.8 (CH_3_CH_2_S, A), 17.66 (((CH_3_)_2_CH)_3_Si, B), 17.74 (((CH_3_)_2_CH)_3_Si, B), 17.78 (((CH_3_)_2_CH)_3_Si, A), 17.83 (((CH_3_)_2_CH)_3_Si, A), 18.01 (((CH_3_)_2_CH)_3_Si, A), 18.05 (((CH_3_)_2_CH)_3_Si, A), 18.17 (((CH_3_)_2_CH)_3_Si, B), 18.23 (((CH_3_)_2_CH)_3_Si, A), 18.25 (((CH_3_)_2_CH)_3_Si, B), 18.30 (((CH_3_)_2_CH)_3_Si, A), 18.32 (((CH_3_)_2_CH)_3_Si, A&, B), 18.4 (((CH_3_)_2_CH)_3_Si, A&, B), 18.5 (((CH_3_)_2_CH)_3_Si, B), 18.6 (((CH_3_)_2_CH)_3_Si, B), 23.8 (CH_3_CH_2_S, B), 24.3 (CH_3_CH_2_S, A), 63.1 (C-6, A), 63.7 (C-6, B), 70.0 (C-4, B), 71.2 (C-2, A), 72.4 (C-4, A), 75.0 (C-3, B), 75.3 (C-2, B), 76.0 (C-5, B), 76.50 (C-3, A), 78.54 (C-5, A), 80.8 (C-1, A), 82.9 (C-1, B); HRMS (ESI): *m*/*z* [M+NH_4_]^+^ Calcd for C_44_H_100_NO_5_SSi_4_^+^ 866.6394; Found: 866.6389.

Data for ethyl 2,3,6-tris-*O*-(triisopropylsilyl)-1-thio-α-d-mannopyranoside (**11**): 87 mg (0.125 mmol, 26%), *R*_f_ = 0.44 (petroleum ether–CH_2_Cl_2_, 2:1) [α]_D_^20^ +48.6 (c 1.00, CHCl_3_); ^1^H NMR (600 MHz, CDCl_3_): δ 1.05–1.18 (m, 63H, 3 × ((CH_3_)_2_CH)_3_Si), 1.28 (t, 4H, *J* 7.4 Hz, SCH_2_CH_3_), 2.49 (d, 1H, *J* 2.0 Hz, OH-4), 2.57 (dq, 1H, *J* 13.0 Hz, *J* 7.5 Hz, SCH_2_CH_3_), 2.63–2.71 (m, 1H, SCH_2_CH_3_), 3.90–3.99 (m, 5H, H-3, H-4, H-5, H-6a, H-6b), 4.17 (dd, 1H, *J* 2.6 Hz, *J* 1.7 Hz, H-2), 5.17 (d, 1H, *J* 1.7 Hz, H-1); ^13^C NMR (151 MHz, CDCl_3_): δ 11.7 (((CH_3_)_2_CH)_3_Si), 12.9 ((CH_3_)_2_CH)_3_Si), 13.0 ((CH_3_)_2_CH)_3_Si), 15.0 (SCH_2_CH_3_), 17.90 ((CH_3_)_2_CH)_3_Si), 17.92 ((CH_3_)_2_CH)_3_Si), 18.17 ((CH_3_)_2_CH)_3_Si), 18.21 ((CH_3_)_2_CH)_3_Si), 18.3 ((CH_3_)_2_CH)_3_Si), 25.0 (SCH_2_CH_3_), 65.1 (C-6), 67.0 (C-4), 73.2 (C-5), 74.3 (C-3), 74.6 (C-2), 85.4 (C-1); HRMS (ESI): *m*/*z* [M+Na]^+^ Calcd for C_35_H_76_NaO_5_SSi_3_^+^ 715.4613; Found: 715.4611.

Data for ethyl 2,4,6-tris-*O*-(triisopropylsilyl)-1-thio-α-d-mannopyranoside (**12**): 18 mg (0.026 mmol, 6%), *R*_f_ = 0.33 (petroleum ether–CH_2_Cl_2_, 2:1). [α]_D_^20^ + 80.73 (c 1.01, CHCl_3_); ^1^H NMR (600 MHz, CDCl_3_): δ 1.03–1.22 (m, 63H, 3 × ((CH_3_)_2_CH)_3_Si), 1.27 (t, 4H, *J* 7.4 Hz, SCH_2_CH_3_), 2.21 (d, 1H, *J* 10.5 Hz, OH-3), 2.54 (dq, 1H, *J* 12.9 Hz, *J* 7.5 Hz, SCH_2_CH_3_), 2.69 (dq, 1H, *J* 12.9 Hz, *J* 7.3 Hz, -SCH_2_CH_3_), 3.68 (ddd, 1H, *J* 10.5 Hz, *J* 8.5 Hz, *J* 3.5 Hz, H-3), 3.81–3.86 (m, 2H, H-4, H-6b), 3.91 (ddd, 1H, *J* 8.7 Hz, *J* 6.4 Hz, *J* 1.9 Hz, H-5), 4.05 (dd, 1H, *J* 10.5 Hz, *J* 1.9 Hz, H-6a), 4.15 (dd, 1H, *J* 3.5 Hz, *J* 1.5 Hz, H-2), 5.27 (d, 1H, *J* 1.5 Hz, H-1); ^13^C NMR (151 MHz, CDCl_3_): δ 11.9 ((CH_3_)_2_CH)_3_Si), 12.6 ((CH_3_)_2_CH)_3_Si), 13.1 (((CH_3_)_2_CH)_3_Si), 14.5 (-SCH_2_CH_3_), 17.92 ((CH_3_)_2_CH)_3_Si), 17.97 ((CH_3_)_2_CH)_3_Si), 18.04 ((CH_3_)_2_CH)_3_Si), 18.4 ((CH_3_)_2_CH)_3_Si), 24.0 (-SCH_2_CH_3_), 63.4 (C-6), 70.9 (C-4), 73.4 (C-3), 74.1 (C-2), 74.5 (C-5), 83.1 (C-1); HRMS (ESI): *m*/*z* [M+NH_4_]^+^ Calcd for C_35_H_81_NO_5_SSi_3_^+^ 710.5060; Found: 710.5060.

### 3.5. Ethyl 2,3,4,6-Tetrakis-O-(Triisopropylsilyl)-1-thio-β-d-mannopyranoside (***13***)

*i*-Pr_3_SiOTf (0.720 mL, 2.68 mmol) was added to the solution of ethyl 1-thio-β-d-mannopyranoside (**9**) (100 mg, 0.445 mmol) in 2,4,6-collidine (1.5 mL) at 20 °C. The reaction mixture was stirred at 90 °C for 45 h and then allowed to reach 20 °C, diluted with CH_2_Cl_2_ (40 mL), washed with H_2_O (40 mL), 1 M KHSO_4_ (3 × 40 mL), H_2_O (40 mL), and satd aq NaHCO_3_ (3 × 40 mL). Organic extracts were dried with Na_2_SO_4_, filtered, concentrated under reduced pressure and purified by silica gel chromatography (gradient: 0 → 5% CH_2_Cl_2_ in petroleum ether) to give silylated derivative **13** (317.5 mg, 0.374 mmol, 84%), *R*_f_ = 0.49 (petroleum ether–CH_2_Cl_2_, 4:1). [α]_D_^20^ −55.2 (c 1.01, CHCl_3_); ^1^H NMR (600 MHz, CDCl_3_, 298 K): δ 1.03–1.21 (m, 84H, 4 × ((CH_3_)_2_CH)_3_Si), 1.26 (t, 3H, *J* 7.4 Hz, CH_3_CH_2_S), 2.59 (dq, 1H, *J* 12.5 Hz, *J* 7.4 Hz, CH_3_CHHS), 2.63 (dq, 1H, *J* 12.6 Hz, *J* 7.5 Hz, CH_3_CHHS), 3.83 (dd, 1H, *J* 8.6 Hz, *J* 4.8 Hz, H-5), 4.16 (t, 1H, *J* 3.2 Hz, H-3), 4.18 (dd, 1H, *J* 10.4 Hz, *J* 4.8 Hz, H-6a), 4.26 (d, 1H, *J* 3.7 Hz, H-4), 4.59 (dd, 1H, *J* 5.7 Hz, *J* 2.6 Hz, H-2), 4.61 (dd, 1H, *J* 10.4 Hz, *J* 8.6 Hz, H-6b), 5.14 (d, 1H, *J* 5.7 Hz, H-1); ^13^C NMR (151 MHz, CDCl_3_): δ 12.1 (((CH_3_)_2_CH)_3_Si), 12.5 (((CH_3_)_2_CH)_3_Si), 12.80 (((CH_3_)_2_CH)_3_Si), 12.81 (((CH_3_)_2_CH)_3_Si), 15.1 (CH_3_CH_2_S), 18.01 (((CH_3_)_2_CH)_3_Si), 18.02 (((CH_3_)_2_CH)_3_Si), 18.13 (((CH_3_)_2_CH)_3_Si), 18.15 (((CH_3_)_2_CH)_3_Si), 18.23 (((CH_3_)_2_CH)_3_Si), 18.3 (((CH_3_)_2_CH)_3_Si), 18.5 (((CH_3_)_2_CH)_3_Si), 18.6 (((CH_3_)_2_CH)_3_Si), 26.7 (CH_3_CH_2_S), 65.0 (C-6), 68.0 (C-2), 72.1 (C-4), 74.3 (C-3), 81.1 (C-5), 84.7 (C-1);

^1^H NMR (600 MHz, CDCl_3_, 240 K): δ 0.99–1.13 (m, 84H, 4 × ((CH_3_)_2_CH)_3_Si), 1.24 (t, 3H, *J* 7.4 Hz, CH_3_CH_2_S), 2.56 (dq, 1H, *J* 12.7 Hz, *J* 7.4 Hz, CH_3_CHHS), 2.59 (dq, 1H, *J* 12.7 Hz, *J* 7.5 Hz, CH_3_CHHS), 3.79 (dd, 1H, *J* 9.1 Hz, *J* 4.6 Hz, H-5), 4.05 (dd, 1H, *J* 10.3 Hz, *J* 4.6 Hz, H-6a), 4.10 (t, 1H, *J* 3.0 Hz, H-3), 4.22 (d, 1H, *J* 3.8 Hz, H-4), 4.54 (dd, 1H, *J* 5.7 Hz, *J* 2.4 Hz, H-2), 4.63 (dd, 1H, *J* 10.3 Hz, *J* 9.2 Hz, H-6b), 5.11 (d, 1H, *J* 5.7 Hz, H-1); ^13^C NMR (151 MHz, CDCl_3_): δ 11.6 (((CH_3_)_2_CH)_3_Si), 12.0 (((CH_3_)_2_CH)_3_Si), 12.4 (((CH_3_)_2_CH)_3_Si), 12.5 (((CH_3_)_2_CH)_3_Si), 15.3 (CH_3_CH_2_S), 17.87 (2 × ((CH_3_)_2_CH)_3_Si), 17.94 (((CH_3_)_2_CH)_3_Si), 18.00 (((CH_3_)_2_CH)_3_Si), 18.1 (((CH_3_)_2_CH)_3_Si), 18.2 (((CH_3_)_2_CH)_3_Si), 18.3 (((CH_3_)_2_CH)_3_Si), 18.4 (((CH_3_)_2_CH)_3_Si), 26.7 (CH_3_CH_2_S), 64.4 (C-6), 67.4 (C-2), 71.3 (C-4), 73.8 (C-3), 80.7 (C-5), 84.3 (C-1); HRMS (ESI): *m*/*z* [M+Na]^+^ Calcd for C_44_H_96_NaO_5_SSi_4_^+^ 871.5948; Found: 871.5949.

### 3.6. Synthesis of 1,2-α-Linked Methyl and Phenyl Mannopyranosides

#### 3.6.1. *General Procedure* for the Synthesis of 1,2-α-Linked Methyl and Phenyl Mannopyranosides **15**, **18** with NIS/TfOH

A mixture of silylated glycosyl donor (**2** or **13**) and glycosyl acceptor (**14** or **17**) was dried in vacuo for 2 h, then anhydrous CH_2_Cl_2_ (2 mL) was added under argon. Freshly activated powdered MS 4 Å (200 mg) were added under argon to the resulting solution. The suspension was stirred under argon at ~22 °C for 1 h, then cooled to −60 °C. Solid NIS (17–22 mg, 1 eq. of glycosyl donor’s moles) was added followed by TfOH (1 μL, 0.012 mmol). Then the temperature was allowed to rise slowly until appearance of persistent characteristic iodine color at −50 °C (for **2** + **14** or **13** + **14**) or −30 °C (for **13** + **17**) and was kept at that temperature for 2 h. Then the reaction was quenched by addition of Py (20 μL). The reaction mixture was diluted with CH_2_Cl_2_ (20 mL) and filtered through a Celite pad. The solids were washed with CH_2_Cl_2_ (20 mL) and the filtrate was successively washed with a mixture of satd aq Na_2_S_2_O_3_ (40 mL) and satd aq NaHCO_3_ (40 mL). The aqueous layer was extracted with CH_2_Cl_2_ (2×5 mL). Combined organic extracts were filtered through a cotton wool plug, concentrated and dried in vacuo. The residue was then dissolved in toluene (2 mL) and subjected to gel chromatography on Bio-Beads S-X3 in toluene. The fractions eluted just after the void volume were collected, concentrated under reduced pressure.

#### 3.6.2. Methyl 2-O-[2,3,4,6-Tetrakis-O-(Triisopropylsilyl)-*α*-d-mannopyranosyl]-3,4,6-tri-O-Benzoyl-*α*-d-mannopyranoside (**15**)

A mixture of silylated α-d-mannopyranoside **2** (88 mg, 0.1 mmol) and methyl mannopyranoside **14** (40 mg, 0.08 mmol) was treated according to *General procedure.* Concentrated fractions were additionally purified by silica gel chromatography in petroleum ether–CH_2_Cl_2_ (gradient: 0 → 35% CH_2_Cl_2_ in petroleum ether) to give α-linked methyl dimannopyranoside **15** as a mixture of conformers (59 mg, 0.046 mmol, 58%); *R*_f_ = 0.66 (petroleum ether–EtOAc, 6:1). [α]_D_^20^ +7.3 (c 1.14, CHCl_3_);^1^H NMR (600 MHz, CDCl_3,_ 298 K, A:B = 10:3): δ 0.80–1.34 (m, 84H, 4 × ((CH_3_)_2_CH)_3_Si), 3.45 (s, 3H, OCH_3_ A), 3.48 (s, 3H, OCH_3_ B), 3.67–3.70 (m, 1H, H-5^II^ B), 3.70 (ddd, 1H, *J* 7.8 Hz, *J* 4.6 Hz, *J* 3.2 Hz, H-5^II^ A), 3.72–3.76 (m, 1H, H-6^II^a B), 3.76 (dd, 1H, *J* 4.6 Hz, *J* 2.3 Hz, H-4^II^ A), 3.80 (dd, 1H, *J* 10.7 Hz, *J* 3.3 Hz, H-6^II^a A), 3.85 (dd, 1H, *J* 10.7 Hz, *J* 8.0 Hz, H-6^II^b A), 4.06 (d, 1H, *J* 10.1 Hz, H-6^II^b B), 4.06 (t, 1H, *J* 2.2 Hz, H-3^II^ A), 4.09 (t, 1H, *J* 9.3 Hz, H-4^II^ B), 4.16 (dd, 1H, *J* 6.5 Hz, *J* 2.0 Hz, H-2^II^ A), 4.21 (t, 1H, *J* 1.9 Hz, H-2^II^ B), 4.30 (dt, 1H, *J* 9.8 Hz, *J* 5.6 Hz, H-5^I^ A), 4.27–4.34 (m, 2H, H-5^I^ B, H-3^II^ B), 4.35 (dd, 1H, *J* 3.2 Hz, *J* 1.9 Hz, H-2^I^ B), 4.43 (dd, 1H, *J* 11.6 Hz, *J* 7.5 Hz, H-6^I^a B), 4.51 (d, 2H, *J* 5.6 Hz, H-6^I^b A, H-6^I^b A), 4.55 (dd, 1H, *J* 11.7 Hz, *J* 3.4 Hz, H-6^I^b B), 4.66 (dd, 1H, *J* 3.3 Hz, *J* 1.9 Hz, H-2^I^ A), 4.76 (d, 1H, *J* 1.8 Hz, H-1^II^ B), 4.88 (d, 1H, *J* 1.8 Hz, H-1^I^ A), 5.05 (d, 1H, *J* 6.5 Hz, H-1^II^ A), 5.07 (d, 1H, *J* 2.0 Hz, H-1^I^ B), 5.63 (dd, 1H, *J* 10.0 Hz, *J* 3.3 Hz, H-3^I^ B), 5.71 (t, 1H, *J* 9.9 Hz, H-4^I^ B), 5.75 (t, 1H, *J* 9.9 Hz, H-4^I^ A), 5.81 (dd, 1H, *J* 10.1 Hz, *J* 3.4 Hz, H-3^I^ A), 7.24–7.38 (m, 6H, C3 × PhCO (H-3, H-5)), 7.41–7.54 (m, 3H, 3 × PhCO (H-4)), 7.86–7.99 (m, 6H, 3 × PhCO (H-2, H-6)); ^13^C NMR (151 MHz, CDCl_3_): δ 12.0 (((CH_3_)_2_CH)_3_Si A&,B), 12.6 (((CH_3_)_2_CH)_3_Si A), 12.81 (((CH_3_)_2_CH)_3_Si A), 12.84 (((CH_3_)_2_CH)_3_Si B), 12.9 (((CH_3_)_2_CH)_3_Si A), 14.1 (((CH_3_)_2_CH)_3_Si B), 14.3 (((CH_3_)_2_CH)_3_Si, B), 17.90 (((CH_3_)_2_CH)_3_Si B), 17.92 (((CH_3_)_2_CH)_3_Si A&,B), 17.94 (((CH_3_)_2_CH)_3_Si A), 18.0 (((CH_3_)_2_CH)_3_Si B), 18.10 (((CH_3_)_2_CH)_3_Si, A), 18.13 (((CH_3_)_2_CH)_3_Si, B), 18.16 (((CH_3_)_2_CH)_3_Si A), 18.19 (((CH_3_)_2_CH)_3_Si A), 18.22 (((CH_3_)_2_CH)_3_Si A), 18.4 (2 × ((CH_3_)_2_CH)_3_Si A), 18.48 (((CH_3_)_2_CH)_3_Si, B), 18.50 (((CH_3_)_2_CH)_3_Si B), 18.7 (((CH_3_)_2_CH)_3_Si B), 18.9 (((CH_3_)_2_CH)_3_Si B), 54.75 (OCH_3_ B), 54.81 (OCH_3_, A), 64.0 (C-6^II^ A), 64.7 (C-6^II^ B), 65.0 (C-6^I^ B), 65.2 (C-6^I^ A), 68.1 (C-4^I^ B), 68.5 (C-5^I^ B), 68.8 (C-4^I^ A), 68.9 (C-5^I^ A), 70.1 (C-4^II^ B), 71.1 (C-2^I^ A), 72.0 (C-3^I^ A), 72.3 (C-3^I^ B), 72.6 (C-2^II^ A), 73.2 (C-4^II^ A), 73.8 (C-3^II^ B), 74.4 (C-2^II^ B), 75.4 (C-2^I^ B), 77.5 (C-3^II^ A), 77.7 (C-5^II^ B), 78.4 (C-5^II^ A), 99.6 (C-1^I^ B), 99.9 (C-1^II^ A), 101.4 (C-1^I^ A), 102.4 (C-1^II^ B), 128.1 (PhCO (C-3, C-5), A), 128.18 (PhCO (C-3, C-5), A), 128.22 (PhCO (C-3, C-5) A&,B), 128.3 (2 × PhCO (C-3, C-5) B), 129.4 (PhCO (C-1)), 129.5 (PhCO), 129.6 (PhCO (C-2, C-6) A&B), 129.7 (PhCO (C-2, C-6) B), 129.77 (PhCO (C-2, C-6)A&B), 129.84 (PhCO (C-2, C-6) A), 132.7 (PhCO (C-4) A&B), 132.8 (PhCO (C-4) A), 132.9 (PhCO (C-4) B), 133.0 (PhCO (C-4) A), 133.1 (PhCO (C-4) B), 165.4 (4^I^-*O*-PhCO A), 166.0 (3^I^-*O*-PhCO A), 166.1 (6^I^-*O*-PhCO A); ^29^Si INEPT NMR (60 MHz, CDCl_3_): δ 11.3 (TIPS A), 12.8 (TIPS A), 13.6 (TIPS A), 15.5 (TIPS A);

^1^H NMR (600 MHz, CDCl_3_, 240 K, A:B = 10:7.6): δ 0.62–1.35 (m, 168H, 3 × ((CH_3_)_2_CH)_3_Si, A, 3 × ((CH_3_)_2_CH)_3_Si, B), 3.46 (s, 3H, OCH_3_, A), 3.49 (s, 3H, OCH_3_, B), 3.65–3.70 (m, 3H, H-6^II^a, B, H-5^II^, A, H-5^II^, B), 3.71 (dd, 1H, *J* 4.8 Hz, *J* 2.2 Hz, H-4^II^, A), 3.74 (dd, 1H, *J* 10.9 Hz, *J* 3.6 Hz, H-6^II^a, A), 3.81 (dd, 1H, *J* 10.7 Hz, *J* 8.1 Hz, H-6^II^b, A), 3.95–4.05 (m, 3H, H-6^II^b,B, H-3^II^, A, H-4^II^, B), 4.12 (dd, 1H, *J* 6.8 Hz, *J* 1.8 Hz, H-2^II^, A), 4.14 (d, 1H, *J* 2.3 Hz, H-2^II^, B), 4.23 (dd, 1H, *J* 9.3 Hz, *J* 1.8 Hz, H-3^II^, B), 4.27–4.40 (m, 4H, H-5^I^, A, H-5^I^, B, H-2^I^, B, H-6^I^a,B), 4.46 (dd, 2H, *J* 11.7 Hz, *J* 7.8 Hz, H-6^I^a, A), 4.48 (dd, 1H, *J* 11.9 Hz, *J* 3.7 Hz, H-6^I^b, A), 4.54 (dd, 1H, *J* 11.2 Hz, *J* 2.3 Hz, H-6^I^b, B), 4.58 (dd, 1H, *J* 3.2 Hz, *J* 1.9 Hz, H-2^I^, A), 4.68 (d, 1H, *J* 1.2 Hz, H-1^II^, B), 4.92 (s, 1H, H-1^I^, A), 4.98 (d, 1H, *J* 6.6 Hz, H-1^II^, A), 5.08 (s, 1H, H-1^I^, B), 5.59 (dd, 1H, *J* 10.1 Hz, *J* 3.4 Hz, H-3^I^, B), 5.69 (t, 1H, *J* 9.7 Hz, H-4^I^, B), 5.72 (t, 1H, *J* 10.1 Hz, H-4^I^, A), 5.83 (dd, 1H, *J* 10.1 Hz, *J* 3.5 Hz, H-3^I^, A), 7.28–7.42 (m, 14H, 3 × PhCO (H-3, H-5), A, 3 × PhCO (H-3, H-5) B), 7.44–7.58 (m, 6H, 3 × PhCO (H-4) A, 3 × PhCO (H-4) B), 7.88–8.02 (m, 12H, 3 × PhCO (H-2, H-6) A, 3 × PhCO (H-2, H-6) B); ^13^C NMR (151 MHz, CDCl_3_): δ 11.48 (((CH_3_)_2_CH)_3_Si, B), 11.52 (((CH_3_)_2_CH)_3_Si, A), 12.2 (((CH_3_)_2_CH)_3_Si, A), 12.37 (((CH_3_)_2_CH)_3_Si, B), 12.41 (((CH_3_)_2_CH)_3_Si, A), 12.6 (((CH_3_)_2_CH)_3_Si, A), 13.95 (((CH_3_)_2_CH)_3_Si, B), 14.04 (((CH_3_)_2_CH)_3_Si, B), 17.7 (((CH_3_)_2_CH)_3_Si, B), 17.6 (((CH_3_)_2_CH)_3_Si,B), 17.78 (((CH_3_)_2_CH)_3_Si, A, ((CH_3_)_2_CH)_3_Si, B), 17.81 (((CH_3_)_2_CH)_3_Si, A), 17.91 (((CH_3_)_2_CH)_3_Si, B), 17.98 (((CH_3_)_2_CH)_3_Si, A), 18.02 (2 × ((CH_3_)_2_CH)_3_Si, A), 18.1 (((CH_3_)_2_CH)_3_Si, A), 18.3 (2 × ((CH_3_)_2_CH)_3_Si, A), 18.36 (2 × ((CH_3_)_2_CH)_3_Si, B), 18.44 (((CH_3_)_2_CH)_3_Si, B), 18.7 (((CH_3_)_2_CH)_3_Si,B), 54.7 (OCH_3_, B), 54.8 (OCH_3_, A), 63.2 (C-6^II^, A), 64.2 (C-6^II^, B), 64.8 (C-6^I^, B), 64.9 (C-6^I^, A), 67.1 (C-4^I^, B), 68.0 (2C, C-4^I^, A, C-5^I^, B), 68.5 (C-5^I^, A), 69.5 (C-4^II^, B), 71.2 (C-2^I^, A), 71.3 (C-3^I^, A), 72.0 (C-2^II^, A), 72.1 (C-3^I^, B), 72.6 (C-4^II^, A), 73.5 (C-3^II^, B), 73.7 (C-2^II^, B), 74.8 (C-2^I^, B), 77.1 (C-5^II^, B), 77.2 (C-3^II^, A), 78.0 (C-5^II^, A), 99.2 (C-1^I^, B), 99.7 (C-1^II^, A), 100.9 (C-1^I^, A), 102.2 (C-1^II^, B), 128.1 (PhCO (C-3, C-5)), 128.2 (PhCO (C-3, C-5)), 128.25 (2 × PhCO (C-3, C-5)), 128.30 (PhCO (C-3, C-5)), 128.33 (PhCO (C-3, C-5)), 128.7 (PhCO (C-1)), 128.8 (2 × PhCO (C-1)), 128.9 (PhCO (C-1)), 129.07 (PhCO (C-1)), 129.14 (PhCO (C-1)), 129.5 (2 × PhCO (C-2, C-6)), 129.56 (PhCO (C-2, C-6)), 129.58 (PhCO (C-2, C-6)), 129.65 (PhCO (C-2, C-6)), 129.73 (PhCO (C-2, C-6)), 132.9 (PhCO (C-4)), 133.0 (PhCO (C-4)), 133.1 (2 × PhCO (C-4)), 133.3 (2 × PhCO (C-4)), 165.2 (PhCO), 165.3 (PhCO), 165.9 (2 × PhCO), 166.18 (PhCO), 166.24 (PhCO); ^29^Si INEPT NMR (119 MHz, CDCl_3_): δ 11.6 (TIPS A), 11.7 (TIPS B), 12.3 (TIPS B), 12.8 (TIPS B), 13.38 (TIPS A), 13.44 (TIPS B), 14.0 (TIPS A), 15.6 (TIPS A); HRMS (ESI): *m*/*z* [M+Na]^+^ Calcd for C_70_H_116_NaO_14_Si_4_^+^ 1315.7334; Found: 1315.7330.

#### 3.6.3. Methyl 2-O-(*α*-d-Mannopyranosyl)-3,4,6-tri-O-benzoyl-*α*-d-mannopyranoside (**16**)

Protected methyl dimannopyranoside **15** (57 mg, 0.04 mmol) was dissolved in THF (2 mL), then AcOH (20 µL, 0.35 mmol) and 1 M TBAF in THF (700 µL, 0.7 mmol) were added. The reaction mixture was stirred at 40 °C for 2.5 h, then concentrated under reduced pressure, co-evaporated with toluene (5 × 5 mL) and dried in vacuo. The residue was purified by silica gel chromatography CH_2_Cl_2_–MeOH (gradient: 0 → 10% MeOH in CH_2_Cl_2_) to give tetraol isolated in a mixture with Bu_4_N. The obtained mixture was subjected to the gel chromatography on Bio-Beads S-X3 in toluene. The fractions eluted just after the void volume were collected, concentrated under reduced pressure to give α-linked methyl dimannopyranoside **16** (21 mg, 0.031 mmol, 71%). *R*_f_ = 0.55 (CH_2_Cl_2_–MeOH, 10:1). [α]_D_^25^ +18.9 (c 1.00, CHCl_3_); ^1^H NMR (600 MHz, CDCl_3_): δ 3.45 (s, 3H, CH_3_O), 3.65–3.74 (m, 2H, H-6^II^a, H-5^II^), 3.87 (d, 1H, *J* 11.3 Hz, H-6^II^b), 3.92–3.97 (m, 2H, H-3^II^, H-4^II^), 3.97–4.02 (m, 1H, HO), 4.04 (s, 1H, H-2^II^), 4.24 (dd, 1H, *J* 3.2 Hz, *J* 1.8 Hz, H-2^I^), 4.29 (ddd, 1H, *J* 9.8 Hz, *J* 5.1 Hz, *J* 3.5 Hz, H-5^I^), 4.35 (s, 1H, HO), 4.42 (s, 1H, HO), 4.50 (dd, 1H, *J* 12.0 Hz, *J* 5.2 Hz, H-6^I^a), 4.52 (s, 1H, 2^II^-HO), 4.53 (dd, 1H, *J* 12.1 Hz, *J* 3.4 Hz, H-6^I^b), 4.91 (d, 1H, *J* 1.9 Hz, H-1^I^), 4.97 (d, 1H, *J* 1.5 Hz, H-1^II^), 5.72 (dd, 1H, *J* 10.0 Hz, *J* 3.1 Hz, H-3^I^), 5.81 (t, 1H, *J* 10.0 Hz, H-4^I^), 7.28–7.35 (m, 4H, 3^I^-*O*-PhCO (H-3, H-5), 4^I^-*O*-PhCO (H-3, H-5)), 7.35–7.40 (m, 2H, 6^I^-*O*-PhCO (H-3, H-5)), 7.40–7.45 (m, 1H, 3^I^- or 4^I^-*O*-PhCO (H-4)), 7.45–7.52 (m, 2H, 4^I^- or 3^I^-*O*-PhCO (H-4), 6^I^-*O*-PhCO (H-4)), 7.90 (s, 4H, 3^I^-*O*-PhCO (H-2, H-6), 4^I^-*O*-PhCO (H-2, H-6)), 7.96–8.03 (m, 2H, 6^I^-*O*-PhCO (H-2, H-6)); ^13^C NMR (151 MHz, CDCl_3_): δ 55.3 (CH_3_O), 61.0 (C-6^II^), 63.5 (C-6^I^), 66.4 (C-4^II^), 67.5 (C-4^I^), 68.7 (C-5^I^), 70.9 (C-2^II^), 71.4 (C-3^I^), 71.5 (C-3^II^), 73.0 (C-5^II^), 76.5 (C-2^I^), 99.7 (C-1^I^), 102.1 (C-1^II^), 128.35 (PhCO (C-3, C-5)), 128.43 (PhCO (C-3, C-5)), 128.5 (PhCO (C-3, C-5)), 129.0 (PhCO (C-1)), 129.1 (PhCO (C-1)), 129.6 (PhCO (C-2, C-6)), 129.67 (PhCO (C-2, C-6)), 129.69 (PhCO (C-1)), 129.8 (PhCO (C-2, C-6)), 133.1 (6^I^-*O*-PhCO (C-4))), 133.29 (3^I^-*O*-PhCO (C-4)), 4^I^-*O*-PhCO (C-4))), 133.34 (3^I^-*O*-PhCO (C-4)), 4^I^-*O*-PhCO (C-4))), 165.55 (3^I^-*O*-PhCO, 4^I^-*O*-PhCO), 165.61 (3^I^-*O*-PhCO, 4^I^-*O*-PhCO), 166.2 (6^I^-*O*-PhCO); HRMS (ESI): *m*/*z* [M+NH_4_]^+^ Calcd for C_34_H_40_NO_14_^+^ 686.2443; Found: 686.2438.

#### 3.6.4. Phenyl 2-O-[2,3,4,6-Tetrakis-O-(Triisopropylsilyl)-*α*-d-mannopyranosyl]-3,4,6-tri-O-benzoyl-1-thio-*α*-d-mannopyranoside (**18**)

(*a*) A mixture of silylated phenyl 1-thio-α-d-mannopyranoside **2** (77 mg, 0.09 mmol) and alcohol **17** [42] (39 mg, 0.07 mmol) was treated according to *General procedure* to give unseparable mixture (98 mg) of disaccharide **18** (85% purity according to NMR data) with *N*-glycoside **19** in 2.5:1 ratio according to NMR data (HRMS (ESI): *m*/*z* [M+Na]^+^ Calcd for C_46_H_95_NNaO_7_Si_4_^+^ 908.6078; Found: 908.6068)).

*(b)* A mixture of ethyl 1-thio-α-d-mannopyranoside **10** (49 mg, 0.06 mmol), TTBP (31 mg, 0.13 mmol), BSP (14 mg, 0.07 mmol) was dried in vacuo for 2 h, then anhydrous CH_2_Cl_2_ (1 mL) was added under argon and freshly activated powdered MS 4 Å (200 mg) were added under argon to the resulting solution. The suspension was stirred under argon at ~22 °C for 1 h, then cooled to −78 °C. After 10 min Tf_2_O (15 μL) was added. Then the temperature was allowed to rise slowly until −60 °C and the solution of phenyl mannopyranoside **17** [42] (40 mg, 0.07 mmol) (previously dried in vacuo for 2 h) in anhydrous CH_2_Cl_2_ (1 mL) was added. Then the temperature was allowed to rise slowly to −50 °C during 0.5 h and was kept at −50 °C for 1 h. Then the reaction was quenched by addition of satd aq NaHCO_3_ (20 µL). The reaction mixture was diluted with CH_2_Cl_2_ (15 mL) and filtered through a Celite pad. The solids were washed with CH_2_Cl_2_ (20 mL) and the filtrate was successively washed with satd aq NaHCO_3_ (50 mL). The aqueous layer was extracted with CH_2_Cl_2_ (2 × 5 mL). Combined organic extracts were filtered through a cotton wool plug, concentrated and dried in vacuo. The residue was dried in vacuo, then dissolved in toluene (2 mL) and subjected to gel chromatography on Bio-Beads S-X3 in toluene. The fractions eluted just after the void volume were collected, concentrated under reduced pressure and additionally purified by silica gel chromatography in petroleum ether–CH_2_Cl_2_ (gradient: 0 → 11% CH_2_Cl_2_ in petroleum ether) to give of α-linked phenyl dimannopyranoside **18** (41 mg, 52% according to NMR) isolated as an inseparable mixture with piperidin-1-yl glycoside **20** (16 mol% according to NMR); HRMS (ESI): *m*/*z* [M+H]^+^ Calcd for C_47_H_111_NO_5_Si_4_^+^ 872.6830; Found: 872.6827)).

(*c*) A mixture of phenyl 1-thio-β-d-mannopyranoside **13** (49 mg, 0.08 mmol) and alcohol **17** [42] (63 mg, 0.07mmol) was treated according to *General procedure*. Residue obtained after gel chromatography was additionally purified by silica gel chromatography in petroleum ether–EtOAc (gradient: 0 → 8% EtOAc in petroleum ether) to give α-linked phenyl dimannopyranoside **18** (79.3 mg, 0.058 mmol, 86%); *R*_f_ = 0.66 (petroleum ether–EtOAc, 10:1); [α]_D_^20^ +56.3 (c 1.01, CHCl_3_); ^1^H NMR (600 MHz, CDCl_3_, 303 K): δ 0.88–1.35 (m, 84H, 3 × ((CH_3_)_2_CH)_3_Si), 3.77 (dt, 1H, *J* 6.9 Hz, *J* 3.6 Hz, H-5^II^), 3.80 (dd, 1H, *J* 10.7 Hz, *J* 4.0 Hz, H-6^II^b), 3.92 (dd, 1H, *J* 10.6 Hz, *J* 6.8 Hz, H-6^II^a), 3.94 (t, 1H, *J* 3.1 Hz, H-4^II^), 4.11 (s, 1H, H-3^II^), 4.21 (dd, 1H, *J* 6.8 Hz, *J* 1.9 Hz, H-2^II^), 4.48 (dd, 1H, *J* 11.8 Hz, *J* 8.2 Hz, H-6^I^a), 4.54 (dd, 1H, *J* 11.8 Hz, *J* 3.1 Hz, H-6^I^b), 4.86 (d, 1H, *J* 3.4 Hz, H-2^I^), 4.86–4.93 (m, 1H, H-5^I^), 5.05 (d, 1H, *J* 6.8 Hz, H-1^II^), 5.73 (dd, 1H, *J* 10.1 Hz, *J* 3.4 Hz, H-3^I^), 5.76 (s, 1H, H-1^I^), 5.80 (t, 1H, *J* 9.9 Hz, H-4^I^), 7.18 (dd, 3H, *J* 12.6 Hz, *J* 7.1 Hz, PhS (H-3, H-4, H-5)), 7.28–7.38 (m, 6H, 3 × PhCO (H-3, H-5)), 7.44–7.57 (m, 5H, 3 × PhCO (H-4), PhS (H-2, H-6)), 7.86–7.90 (m, 2H, PhCO (H-2, H-6)), 7.90–7.94 (m, 3H, PhCO (H-2, H-6)), 7.96–8.01 (m, 2H, PhCO (H-2, H-6)); ^13^C NMR (151 MHz, CDCl_3_): δ 12.0 (((CH_3_)_2_CH)_3_Si), 12.6 (((CH_3_)_2_CH)_3_Si), 12.9 (((CH_3_)_2_CH)_3_Si), 13.0 (((CH_3_)_2_CH)_3_Si), 18.02 (((CH_3_)_2_CH)_3_Si), 18.03 (((CH_3_)_2_CH)_3_Si), 18.1 (((CH_3_)_2_CH)_3_Si), 18.15 (((CH_3_)_2_CH)_3_Si), 18.23 (((CH_3_)_2_CH)_3_Si), 18.3 (((CH_3_)_2_CH)_3_Si), 18.5 (2 × ((CH_3_)_2_CH)_3_Si), 63.2 (C-6^II^), 64.7 (C-6^I^), 68.6 (C-4^I^), 70.0 (C-5^I^), 72.1 (C-3^I^), 72.3 (C-2^II^), 72.8 (C-4^II^), 73.6 (C-2^I^), 77.3 (C-3^II^), 79.5 (C-5^II^), 88.5 (C-1^I^), 99.7 (C-1^II^), 127.0 (PhS (C-4)), 128.1 (PhCO (C-3, C-5)), 128.2 (PhCO (C-3, C-5)), 128.3 (PhCO (C-3, C-5)), 128.9 (PhS (C-3, C-5)), 129.3 (PhCO (C-1)), 129.4 (PhCO (C-1)), 129.6 (PhCO (C-2, C-6)), 129.8 (PhCO (C-2, C-6)), 129.9 (PhCO (C-2, C-6)), 131.3 (PhS (C-2, C-6)), 132.8 (PhCO (C-4)), 132.9 (PhCO (C-4)), 133.1 (PhCO (C-4)), 134.3 (PhS (C-1)), 165.4 (PhCO), 166.0 (PhCO), 166.1 (PhCO); ^29^Si INEPT NMR (119 MHz, CDCl_3_): δ 12.0 (TIPS), 12.9 (TIPS), 13.9 (TIPS), 15.7 (2^II^-*O*-TIPS); ^1^H NMR (600 MHz, CDCl_3_, 240 K, A:B = 10:3.4): δ 0.78–1.28 (m, 126H, 3 × ((CH_3_)_2_CH)_3_Si), 3.63 (t, 1H, *J* 8.3 Hz, H-5^II^ B), 3.69–3.74 (m, 2H, H-6^II^a B, H-5^II^ A), 3.76 (dd, 1H, *J* 10.6 Hz, *J* 4.6 Hz, H-6^II^a A), 3.81–3.85 (m, 1H, H-6^II^b B), 3.86 (dd, 1H, *J* 10.8 Hz, *J* 6.5 Hz, H-6^II^b A), 3.93 (t, 1H, *J* 3.0 Hz, H-4^II^ A), 4.04 (t, 1H, *J* 2.2 Hz, H-3^II^ A), 4.07 (t, 1H, *J* 9.3 Hz, H-4^II^ B), 4.12–4.13 (m, 1H, H-2^II^ B), 4.15 (dd, 1H, *J* 7.1 Hz, *J* 1.9 Hz, H-2^II^ A), 4.23 (dd, 1H, *J* 9.4 Hz, *J* 1.7 Hz, H-3^II^ B), 4.33 (dd, 1H, *J* 11.7 Hz, *J* 8.8 Hz, H-6^I^a B), 4.43 (dd, 1H, *J* 11.8 Hz, *J* 8.9 Hz, H-6^I^a A), 4.51 (dd, 1H, *J* 11.9 Hz, *J* 2.5 Hz, H-6^I^b A), 4.55 (dd, 1H, *J* 11.9 Hz, *J* 2.3 Hz, H-6^I^b B), 4.61 (d, 1H, *J* 3.6 Hz, H-2^I^ B), 4.72 (s, 1H, H-1^II^ B), 4.73 (d, 1H, *J* 3.5 Hz, H-2^I^ A), 4.89–4.99 (m, 2H, H-5^I^ A, H-5^I^ B), 4.96 (d, 1H, *J* 7.0 Hz, H-1^II^ A), 5.55 (dd, 1H, *J* 10.1 Hz, *J* 3.3 Hz, H-3^I^ B), 5.70 (dd, 1H, *J* 10.3 Hz, *J* 3.5 Hz, 3^I^ A), 5.72–5.80 (m, 2H, 4^I^ B, 4^I^ A), 5.80 (s, 1H, H-1^I^ A), 5.91 (s, 1H, H-1^I^ B), 7.16 (t, 2H, *J* 7.6 Hz, PhS (H-3, H-5) A), 7.19–7.26 (m, 4H, PhS (H-3, H-5) B, PhS (H-4) A, PhS (H-4) B), 7.30–7.42 (m, 12H, 3 × PhCO (H-3, H-5) A, 3 × PhCO (H-3, H-5) B), 7.47–7.60 (m, 10H, 3 × PhCO (H-4) A, 3 × PhCO (H-4) B, PhS (H-2, H-6) A, PhS (H-2, H-6) B), 7.86–7.90 (m, 4H, PhCO (H-2, H-6) A, PhCO (H-2, H-6) B), 7.92 (d, 4H, *J* 7.8 Hz, PhCO (H-2, H-6) A, PhCO (H-2, H-6) B), 7.97–8.02 (m, 4H, PhCO (H-2, H-6) A, PhCO (H-2, H-6) B); ^13^C NMR (151 MHz, CDCl_3_): δ 11.4 (((CH_3_)_2_CH)_3_Si B), 11.5 (((CH_3_)_2_CH)_3_Si A), 12.1 (((CH_3_)_2_CH)_3_Si A), 12.4 (((CH_3_)_2_CH)_3_Si B), 12.5 (((CH_3_)_2_CH)_3_Si A), 12.7 (((CH_3_)_2_CH)_3_Si A), 13.9 (((CH_3_)_2_CH)_3_Si B), 14.1 (((CH_3_)_2_CH)_3_Si B), 17.7 (((CH_3_)_2_CH)_3_Si), 17.79 (((CH_3_)_2_CH)_3_Si), 17.84 (((CH_3_)_2_CH)_3_Si), 17.88 (((CH_3_)_2_CH)_3_Si), 17.92 (((CH_3_)_2_CH)_3_Si), 18.01 (((CH_3_)_2_CH)_3_Si), 18.03 (((CH_3_)_2_CH)_3_Si), 18.2 (((CH_3_)_2_CH)_3_Si), 18.3 (((CH_3_)_2_CH)_3_Si), 18.40 (((CH_3_)_2_CH)_3_Si), 18.45 (((CH_3_)_2_CH)_3_Si), 18.48 (((CH_3_)_2_CH)_3_Si), 18.7 (((CH_3_)_2_CH)_3_Si), 62.2 (C-6^II^ A), 63.5 (C-6^II^ B), 64.3 (C-6^I^ B), 64.4 (C-6^I^ B), 66.8 (C-4^I^ A), 67.7 (C-4^I^ A), 69.1 (2 C, C-4^II^ B, C-5^I^ B), 69.4 (C-5^I^ A), 71.4 (C-2^II^ A), 71.5 (C-3^I^ A), 71.8 (C-4^II^ A), 72.6 (C-3^I^ B), 73.5 (C-3^II^ B), 73.7 (C-2^II^ B), 74.2 (C-2^I^ A), 76.5 (C-2^I^ B), 77.0 (2 C, C-3^II^ A, C-5^II^ B), 79.1 (C-5^II^ A), 85.7 (C-1^I^ A), 88.2 (C-1^I^ A), 99.7 (C-1^II^ A), 102.9 (C-1^II^ B), 127.0 (PhS (C-4) B), 127.1 (PhS (C-4) A), 128.17 (PhCO (C-3, C-5)), 128.21 (PhCO (C-3, C-5)), 128.3 (PhCO), 128.36 (PhCO), 128.41 (PhCO), 128.5 (PhCO), 128.6 (PhCO), 128.9 (PhS (C-3, C-5) A), 129.0 (PhS (C-3, C-5) B), 129.1 (PhCO (C-1)), 129.58 (PhCO (C-2, C-6)), 129.66 (PhCO (C-2, C-6)), 129.71 (PhCO (C-2, C-6)), 129.8 (PhCO (C-2, C-6)), 130.3 (PhS (C-2, C-6) B), 131.2 (PhS (C-2, C-6) A), 133.06 (PhCO (C-4)), 133.11 (PhCO (C-4)), 133.3 (PhCO (C-4)), 133.38 (PhCO (C-4)), 133.43 (PhCO (C-4)), 133.5 (PhCO (C-4)), 133.7 (PhS (C-1) A), 165.1 (PhCO), 165.2 (PhCO), 165.9 (PhCO), 166.0 (PhCO), 166.2 (PhCO); ^29^Si INEPT NMR (119 MHz, CDCl_3_): δ 12.0, 12.2, 12.6, 12.9, 13.5, 14.0, 14.3, 15.8; HRMS (ESI): *m*/*z* [M+Na]^+^ Calcd for C_75_H_118_NaO_13_SSi_4_^+^ 1393.7262; Found: 1393.7262.

### 3.7. Synthesis of Mannose-Capped Trisaccharide of M. tuberculosis: 2-Chloroethyl 2,3-di-O-benzoyl-5-O-[3,4,6-tri-O-benzoyl-α-d-mannopyranosyl-2-O-{2,3,4,6-tetrakis-O-(Triisopropylsilyl)-α-d-mannopyranosyl}]-α-d-arabinofuranoside (***22***) and 2-Chloroethyl 2,3-di-O-benzoyl-5-O-(3,4,6-tri-O-benzoyl-α-d-mannopyranosyl)-α-d-arabinofuranoside (***23***)

(a) A mixture of 2-chloroethyl α-d-arabinofuranoside **21** [43] (14 mg, 0.033 mmol) and dimannothiopyranoside **18** (36 mg, 0.026 mmol) was dried in vacuo for 2 h, then dissolved in anhydrous CH_2_Cl_2_ (1.5 mL) under argon. Freshly activated powdered MS 4 Å (150 mg) were added under argon the resulting solution. The suspension was stirred under argon at ~22 °C for 1 h, then cooled to −10 °C. Solid NIS (6 mg, 0.03 mmol) was added followed by TfOH (1.4 µL, 0.017 mmol). Then the temperature was allowed to rise to 0 °C during 1 h and was kept at 0 °C for 16 h. Then the reaction was quenched by addition of Et_3_N (15 μL). The reaction mixture was diluted with CH_2_Cl_2_ (20 mL) and filtered through a Celite pad. The solids were washed with CH_2_Cl_2_ (20 mL) and the filtrate was successively washed with a mixture of satd aq Na_2_S_2_O_3_ (40 mL) and satd aq NaHCO_3_ (40 mL). The aqueous layer was extracted with CH_2_Cl_2_ (2 × 5 mL). Combined organic extracts were filtered through a cotton wool plug, concentrated and dried in vacuo. The residue was dried in vacuo, then purified by silica gel chromatography in petroleum ether–EtOAc (gradient: 0 → 12% EtOAc in petroleum ether) to give α-linked trisaccharide **22** as a mixture of conformers (34 mg, 0.020 mmol, 78%). *R*_f_ = 0.45 (petroleum ether–EtOAc, 5:1), [α]_D_^24^ −1.3 (c 1.07, CHCl_3_); HRMS (ESI): *m*/*z* [M+NH_4_]^+^ Calcd for C_90_H_137_NClO_20_Si_4_^+^ 1698.8494; Found: 1698.8512.

(b) A mixture of ethyl 1-thio-α-d-mannopyranoside **10** (47 mg, 0.055 mmol) and phenyl mannothiopyranoside **17** (39 mg, 0.067 mmol) was dried in vacuo for 2 h, then anhydrous CH_2_Cl_2_ (2 mL) was added under argon. Freshly activated powdered MS 4 Å (100 mg) were added under argon to the resulting solution and the suspension was stirred under argon at ~22 °C for 1 h, then cooled to −78 °C. Solid NIS (15 mg, 0.067 mmol) was added followed by TfOH (0.75 µL, 0.009 mmol). Then the temperature was allowed to rise to −40 °C during 1 h. After that, 2-chloroethyl α-d-arabinofuranoside **21** [43] (31 mg, 0.073 mmol) in anhydrous CH_2_Cl_2_ (0.6 mL), solid NIS (15.5 mg, 0.07 mmol) and TfOH (0.9 μL, 0.011 mmol) were added in this order to the reaction mixture. Then the temperature was allowed to rise to 0 °C during 1 h and was kept at 0 °C for 20 h. Since the reaction was not complete (according to TLC), an additional portion of arabinofuranoside **21** (16 mg, 0.038 mmol) in anhydrous CH_2_Cl_2_ (0.2 mL), solid NIS (9 mg, 0.04 mmol) and TfOH (0.75 µL, 0.009 mmol). were added. After additional 3 h at 0 °C the reaction was quenched by addition of Py (15 μL). The reaction mixture was diluted with CH_2_Cl_2_ (15 mL) and filtered through a Celite pad. The solids were washed with CH_2_Cl_2_ (20 mL) and the filtrate was successively washed with a mixture of satd aq Na_2_S_2_O_3_ (20 mL) and satd aq NaHCO_3_ (20 mL). The aqueous layer was extracted with CH_2_Cl_2_ (2 × 5 mL). Combined organic extracts were filtered through a cotton wool plug, concentrated and dried in vacuo. The residue was dried in vacuo, then dissolved in toluene (2 mL) and subjected to gel chromatography on Bio-Beads S-X3 in toluene. The fractions eluted just after the void volume were collected, concentrated under reduced pressure. The fist fraction was additionally purified by silica gel chromatography in petroleum ether–EtOAc (gradient: 5 → 15% EtOAc in petroleum ether) to give an α-linked trisaccharide **22** as a mixture of conformers (46 mg, 0.027 mmol, 49%) and a partially protected trisaccharide with three TIPS groups (4 mg, 0.0026 mmol, 5%, HRMS (ESI): *m*/*z* [M+Na]^+^ Calcd for C_81_H_113_ClNaO_20_Si_3_^+^ 1547.6714; Found: 1547.6706)). The second fraction (after gel chromatography on Bio-Beads S-X3) was also subjected to the silica gel chromatography in petroleum ether–EtOAc (gradient: 8 → 32% EtOAc in petroleum ether) to give α-linked benzoylated disacharide **23** (16 mg, 0.018 mmol, 27%).

Data for conformer A:

^1^H NMR (600 MHz, CDCl_3_, 303 K, A:B = 10:3): δ 0.80–1.35 (m, 84H, 4 × ((CH_3_)_2_CH)_3_Si), 3.70–3.77 (m, 3H, H-5^III^, CH_2_Cl), 3.80 (dd, 1H, *J* 10.8 Hz, *J* 3.5 Hz, H-6^III^a), 3.82–3.89 (m, 3H, H-6^III^b, H-4^III^, CHHO), 3.93 (dd, 1H, *J* 10.6 Hz, *J* 2.4 Hz, H-5^I^a), 4.03–4.08 (m, 1H, CHHO), 4.08 (t, 1H, *J* 2.2 Hz, H-3^III^), 4.16 (dd, 1H, *J* 10.6 Hz, *J* 7.0 Hz, H-5^I^b), 4.20 (dd, 1H, *J* 6.6 Hz, *J* 2.0 Hz, H-2^III^), 4.47 (dd, 1H, *J* 12.4 Hz, *J* 8.3 Hz, H-6^II^a), 4.52–4.61 (m, 3H, H-5^II^, H-6^II^b, H-4^I^), 4.70 (dd, 1H, *J* 3.5 Hz, *J* 1.8 Hz, H-2^II^), 5.06 (d, 1H, *J* 6.6 Hz, H-1^III^), 5.09 (d, 1H, *J* 1.8 Hz, H-1^II^), 5.14 (s, 1H, H-1^I^), 5.51 (dd, 1H, *J* 6.4 Hz, *J* 2.4 Hz, H-3^I^), 5.54 (d, 1H, *J* 2.5 Hz, H-2^I^), 5.78 (t, 1H, *J* 9.8 Hz, H-4^II^), 5.87 (dd, 1H, *J* 10.1 Hz, *J* 3.4 Hz, H-3^II^), 7.16–7.23 (m, 2H, PhCO (H-3, H-5)), 7.27–7.33 (m, 5H, 2 × PhCO (H-3, H-5), PhCO (H-4)), 7.36–7.41 (m, 4H, 2 × PhCO (H-3, H-5)), 7.42–7.56 (m, 4H, 4 × PhCO (H-4)), 7.86–7.92 (m, 4H, 2 × PhCO (H-2, H-6)), 7.93–7.98 (m, 2H, PhCO (H-2, H-6)), 8.01–8.06 (m, 4H, 2 × PhCO (H-2, H-6)); ^13^C NMR (151 MHz, CDCl_3_): δ 11.9 (((CH_3_)_2_CH)_3_Si), 12.7 (((CH_3_)_2_CH)_3_Si), 12.9 (((CH_3_)_2_CH)_3_Si), 13.0 (((CH_3_)_2_CH)_3_Si), 17.91 (((CH_3_)_2_CH)_3_Si), 17.93 (((CH_3_)_2_CH)_3_Si), 18.1 (((CH_3_)_2_CH)_3_Si), 18.17 (((CH_3_)_2_CH)_3_Si), 18.23 (((CH_3_)_2_CH)_3_Si), 18.3 (((CH_3_)_2_CH)_3_Si), 18.45 (((CH_3_)_2_CH)_3_Si), 18.46 (((CH_3_)_2_CH)_3_Si), 42.7 (CH_2_Cl), 63.7 (C-6^III^), 65.1 (C-6^II^), 67.2 (C-5^I^), 67.8 (CH_2_O), 68.8 (C-4^II^), 69.1 (C-5^II^), 71.8 (C-2^II^), 72.1 (C-3^II^), 72.5 (C-2^III^), 73.1 (C-4^III^), 77.2 (C-3^I^), 77.5 (C-3^III^), 78.7 (C-5^III^), 81.0 (C-4^I^), 82.5 (C-2^I^), 99.9 (C-1^III^), 100.1 (C-1^II^), 105.9 (C-1^I^), 128.09 (PhCO (C-3, C-5)), 128.11 (PhCO (C-3, C-5)), 128.2 (PhCO (C-3, C-5)), 128.36 (PhCO (C-3, C-5)), 128.41 (PhCO (C-3, C-5)), 129.1 (PhCO (C-1)), 129.2 (PhCO (C-1)), 129.5 (PhCO (C-2, C-6)), 129.8 (PhCO (C-2, C-6)), 129.87 (PhCO (C-2, C-6)), 129.94 (PhCO (C-2, C-6)), 130.0 (PhCO (C-2, C-6)), 132.66 (PhCO (C-4)), 132.69 (PhCO (C-4)), 132.9 (PhCO (C-4)), 133.4 (2 × PhCO (C-4)), 165.47 (CO), 165.55 (CO), 165.58 (CO), 165.7 (CO), 166.0 (CO); ^29^Si INEPT NMR (119 MHz, CDCl_3_): δ 11.5, 12.8, 13.7, 15.6;

Data for conformer B (selected signals):

^1^H NMR (600 MHz, CDCl_3_, 303 K, A:B = 10:3): δ 3.96 (dd, 1H, *J* 10.5 Hz, *J* 2.3 Hz, H-5^I^a), 4.10 (t, 1H, *J* 9.2 Hz, H-4^III^), 4.22 (t, 1H, *J* 1.9 Hz, H-2^III^), 4.32 (dd, 1H, *J* 9.1 Hz, *J* 1.9 Hz, H-3^III^), 4.42 (dd, 1H, *J* 12.3 Hz, *J* 8.2 Hz, H-6^II^a), 4.79 (s, 1H, H-1^III^), 5.18 (s, 1H, H-1^I^), 5.27 (s, 1H, H-1^II^), 5.56 (dd, 1H, *J* 6.2 Hz, *J* 2.5 Hz, H-3^I^), 5.71 (dd, 1H, *J* 9.9 Hz, *J* 3.0 Hz, H-3^II^), 5.75 (t, 1H, *J* 9.7 Hz, H-4^II^); ^13^C NMR (151 MHz, CDCl_3_): δ 11.9 (((CH_3_)_2_CH)_3_Si), 12.9 (((CH_3_)_2_CH)_3_Si), 14.1 (((CH_3_)_2_CH)_3_Si), 14.3 (((CH_3_)_2_CH)_3_Si), 18.1 (((CH_3_)_2_CH)_3_Si), 18.2 (((CH_3_)_2_CH)_3_Si), 18.50 (((CH_3_)_2_CH)_3_Si), 18.52 (((CH_3_)_2_CH)_3_Si), 18.7 (((CH_3_)_2_CH)_3_Si), 18.9 (((CH_3_)_2_CH)_3_Si), 42.7 (CH_2_Cl), 64.7 (C-6^III^), 65.0 (C-6^II^), 66.9 (C-5^I^), 67.8 (CH_2_O), 68.1 (C-4^II^), 68.8 (C-5^II^), 70.2 (C-4^III^), 72.4 (C-3^II^), 73.8 (C-3^III^), 74.5 (C-2^III^), 75.6 (C-2^II^), 77.0 (C-3^I^), 77.7 (C-5^III^), 80.8 (C-4^I^), 82.5 (C-2^I^), 98.5 (C-1^II^), 102.5 (C-1^III^), 105.9 (C-1^I^);

^1^H NMR (600 MHz, CDCl_3_, 236 K, A:B = 10:8.6): δ 0.62–1.37 (m, 168H, 4 × ((CH_3_)_2_CH)_3_Si A, 4 × ((CH_3_)_2_CH)_3_Si B), 3.63–3.71 (m, 3H, H-6^III^a B, H-5^III^ B, H-5^III^ A), 3.74 (dd, 1H, *J* 10.7 Hz, *J* 3.7 Hz, H-6^III^a A), 3.77–3.98 (m, 11H, CH_2_Cl A, CH_2_Cl B, H-4^III^ A, H-6^III^b A, CHHO A, CHHO B, H-5^I^a A, H-5^I^a B, H-6^III^b B), 3.98–4.04 (m, 2H, H-4^III^ B, H-3^III^ A), 4.08 (dt, 2H, *J* 11.1 Hz, *J* 5.5 Hz, CHHO A, CHHO B), 4.12–4.21 (m, 4H, H-2^III^ B, H-2^III^ A, H-5^I^b A, H-5^I^b B), 4.23 (d, 1H, *J* 9.2 Hz, H-3^III^ B), 4.31–4.37 (m, 1H, H-6^II^a A or B), 4.38–4.46 (m, 2H, H-6^II^a A or B, H-2^II^ B), 4.51–4.62 (m, 7H, H-6^II^b A, H-6^II^b B, H-5^II^ A, H-5^II^ B, H-4^I^ A, H-4^I^ B, H-2^II^ A), 4.71 (s, 1H, H-1^III^ B), 5.00 (d, 1H, *J* 6.9 Hz, H-1^III^ A), 5.08 (s, 1H, H-1^I^ A), 5.12 (s, 1H, H-1^II^ A), 5.13 (s, 1H, H-1^I^ B), 5.25 (s, 1H, H-1^II^ B), 5.48–5.52 (m, 2H, H-3^I^ A, H-2^I^ B), 5.52 (d, 1H, *J* 2.2 Hz, H-2^I^ A), 5.54 (dd, 1H, *J* 6.6 Hz, *J* 2.5 Hz, H-3^I^ B), 5.66–5.77 (m, 3H, H-3^II^ B, H-4^II^ B, H-4^II^ A), 5.91 (dd, 1H, *J* 10.1 Hz, *J* 3.4 Hz, H-3^II^ A), 7.16–7.65 (m, 30H, 5 × PhCO (H-3, H-4, H-5) A, 5 × PhCO (H-3, H-4, H-5) B), 7.86–8.07 (m, 20H, 5 × PhCO (H-2, H-6) A, 5 × PhCO (H-2, H-6) B); ^13^C NMR (151 MHz, CDCl_3_): δ 11.4 (((CH_3_)_2_CH)_3_Si), 11.4 (((CH_3_)_2_CH)_3_Si), 12.2 (((CH_3_)_2_CH)_3_Si), 12.4 (((CH_3_)_2_CH)_3_Si), 12.5 (((CH_3_)_2_CH)_3_Si), 12.7 (((CH_3_)_2_CH)_3_Si), 14.0 (((CH_3_)_2_CH)_3_Si), 14.1 (((CH_3_)_2_CH)_3_Si), 17.7 (2 × ((CH_3_)_2_CH)_3_Si), 17.76 (2 × ((CH_3_)_2_CH)_3_Si), 17.81 (((CH_3_)_2_CH)_3_Si), 17.9 (((CH_3_)_2_CH)_3_Si), 17.99 (((CH_3_)_2_CH)_3_Si), 18.03 (((CH_3_)_2_CH)_3_Si), 18.06 (((CH_3_)_2_CH)_3_Si), 18.13 (((CH_3_)_2_CH)_3_Si), 18.26 (((CH_3_)_2_CH)_3_Si), 18.27 (((CH_3_)_2_CH)_3_Si), 18.36 (((CH_3_)_2_CH)_3_Si), 18.38 (((CH_3_)_2_CH)_3_Si), 18.5 (((CH_3_)_2_CH)_3_Si), 18.7 (((CH_3_)_2_CH)_3_Si), 43.0 (CH_2_Cl), 62.8 (C-6^III^ A), 64.1 (C-6^III^ B), 64.6 (C-6^II^ A, C-6^II^ B), 64.7 (C-6^II^ A, C-6^II^ B), 66.4 (C-5^I^ B), 66.7 (C-5^I^ A), 67.1 (C-4^II^ B), 67.35 (CH_2_O A or B), 67.40 (CH_2_O A or B), 68.0 (C-4^II^ A), 68.2 (C-5^II^ B), 68.8 (C-5^II^ A), 69.4 (C-4^III^ B), 71.1 (C-3^II^ A), 71.7 (C-2^III^ A), 71.96 (C-2^II^ A, C-3^II^ B), 71.99 (C-2^II^ A, C-3^II^ B), 72.29 (C-4^III^ A), 73.38 (C-3^III^ B), 73.8 (C-2^III^ B), 75.1 (C-2^II^ B), 76.5 (C-3^I^ B), 76.7 (C-3^I^ A), 77.0 (C-5^III^ B), 77.1 (C-3^III^ A), 78.5 (C-5^III^ A), 80.0 (C-4^I^ B), 80.5 (C-4^I^ A), 82.1 (C-2^I^ A), 82.3 (C-2^I^ B), 98.2 (C-1^II^ B), 99.6 (C-1^III^ A), 99.7 (C-1^II^ A), 102.3 (C-1^III^ B), 105.4 (2 C, C-1^I^ A, C-1^I^ B), 128.05 (PhCO (C-3, C-5)), 128.10 (PhCO (C-3, C-5)), 128.11 (PhCO (C-3, C-5)), 128.2 (PhCO (C-3, C-5)), 128.26 (PhCO (C-3, C-5)), 128.28 (PhCO (C-3, C-5)), 128.33 (4 × PhCO (C-3, C-5)), 128.40 (PhCO (C-1)), 128.44 (3 × PhCO (C-1)), 128.8 (PhCO (C-1)), 128.86 (PhCO (C-1)), 128.88 (PhCO (C-1)), 128.92 (PhCO (C-1)), 129.2 (PhCO (C-1)), 129.3 (PhCO (C-1)), 129.4 (2 × PhCO (C-2, C-6)), 129.5 (PhCO (C-2, C-6)), 129.66 (PhCO (C-2, C-6)), 129.70 (PhCO (C-2, C-6)), 129.72 (PhCO (C-2, C-6)), 129.77 (PhCO (C-2, C-6)), 129.79 (PhCO (C-2, C-6)), 129.80 (PhCO (C-2, C-6)), 129.9 (PhCO (C-2, C-6)), 132.8 (PhCO (C-4)), 132.87 (PhCO (C-4)), 132.92 (PhCO (C-4)), 133.1 (PhCO (C-4)), 133.2 (PhCO (C-4)), 133.3 (PhCO (C-4)), 133.5 (4 × PhCO (C-4)), 165.3 (CO), 165.4 (CO), 165.46 (CO), 165.52 (CO), 165.53 (CO), 165.58 (CO), 165.62 (CO), 165.7 (CO), 166.06 (CO), 166.09 (CO); ^29^Si INEPT NMR (119 MHz, CDCl_3_): δ 11.9 (2 × TIPS), 12.3 (TIPS), 12.9 (TIPS), 13.4 (TIPS), 13.5 (TIPS), 14.0 (TIPS), 15.7 (TIPS).

Data for benzoylated disaccharide (**23**):

*R*_f_ = 0.33 (petroleum ether–EtOAc, 2:1); [α]_D_^25^ +5.0 (c 1.00, CHCl_3_); ^1^H NMR (600 MHz, CDCl_3_): δ 2.30 (d, 1H, J 4.5 Hz, OH-2^II^), 3.72–3.78 (m, 1H, CH_2_Cl), 3.85–3.92 (m, 1H, CH_2_aO), 4.01–4.09 (m, 2H, CH_2_bCl, H-5a^I^), 4.22 (dd, 1H, J 11.3 Hz, J 5.1 Hz, H-5b^I^), 4.37–4.43 (m, 1H, H-2^II^), 4.50 (dd, 1H, J 11.9 Hz, J 5.0 Hz, H-6a^II^), 4.52–4.58 (m, 2H, H-4^I^, H-5^II^), 4.63 (dd, 1H, J 11.9 Hz, J 2.9 Hz, H-6b^II^), 5.16 (d, 1H, J 1.8 Hz, H-1^II^), 5.30 (s, 1H, H-1^I^), 5.57 (d, 1H, J 1.6 Hz, H-2^I^), 5.61 (dd, 1H, J 5.5 Hz, J 1.6 Hz, H-3^I^), 5.74 (dd, 1H, J 9.9 Hz, J 3.2 Hz, H-3^II^), 5.97 (t, 1H, J 10.0 Hz, H-4^II^), 7.30–7.54 (m, 7H, Ph(H-3, H-4, H-5)), 7.57–7.62 (m, 1H, Ph(H-4)), 7.91–7.99 (m, 2H, Ph(H-2, H-6)), 7.99–8.04 (m, 1H, Ph(H-2, H-6)), 8.06–8.13 (m, 2H, Ph(H-2, H-6)); ^13^C NMR (151 MHz, CDCl_3_): δ 42.7 (CH_2_Cl), 63.4 (C-6^II^), 66.8 (C-5^I^), 67.0 (C-4^II^), 67.7 (CH_2_O), 69.0 (C-5^II^), 69.3 (C-2^II^), 72.6 (C-3^II^), 77.2 (C-3^I^), 81.9 (C-4^I^), 82.3 (C-2^I^), 99.9 (C-1^II^), 105.8 (C-1^I^), 128.30 (Ph(C-3, C-5)), 128.34 (Ph(C-3, C-5)), 128.4 (Ph(C-3, C-5)), 128.48 (Ph(C-3, C-5)), 128.53 (Ph(C-3, C-5)), 129.0 (Ph(C-1)), 129.1 (Ph(C-1)), 129.2 (Ph(C-1)), 129.3 (Ph(C-1)), 129.7 (Ph(C-2, C-6)), 129.78 (Ph(C-2, C-6)), 129.82 (Ph(C-2, C-6)), 129.9 (Ph(C-2, C-6)), 130.0 (Ph(C-2, C-6)), 133.0 (Ph(C-4)), 133.2 (Ph(C-4)), 133.3 (Ph(C-4)), 133.5 (2 × Ph(C-4)), 165.3 (PhCO), 165.5 (PhCO), 165.6 (PhCO), 165.8 (PhCO), 166.2 (PhCO); HRMS (ESI): *m*/*z* [M+H]^+^ Calcd for C_48_H_43_ClNaO_15_^+^ 917.2183; Found: 872.6827.

### 3.8. 2-Azidoethyl 2,3-di-O-Benzoyl-5-O-[3,4,6-tri-O-benzoyl-α-d-mannopyranosyl-2-O-{2,3,4,6-tetrakis-O-(Triisopropylsilyl)-α-d-mannopyranosyl}]-α-d-arabinofuranoside (***24***)

Trisaccharide **23** (32 mg, 0.019 mmol), NaN_3_ (7.4 mg, 0.11 mmol) and 18-crown-6 (3 mg, 0.016 mmol) in DMF (0.6 mL), was stirred at 80 °C for 22 h. Then the reaction mixture was concentrated, the residue was co-evaporated with toluene (4 × 5 mL), concentrated under reduced pressure (bath temperature ~35 °C) and purified by silica gel column chromatography (gradient: 0 → 15% EtOAc in petroleum ether) to give azide **24** (29 mg, 0.017 mmol, 90%). *R*_f_ = 0.45 (petroleum ether–EtOAc, 5:1); [α]_D_^22^ −8.9 (c 0.96; CHCl_3_);

Data for conformer A:

^1^H NMR (600 MHz, CDCl_3_, 303 K, A:B =10:3): δ 0.82–1.33 (m, 84H, 4 × ((CH_3_)_2_CH)_3_Si), 3.49 (dd, 3H, *J* 5.8 Hz, *J* 4.6 Hz, CH_2_N_3_), 3.69–3.77 (m, 2H, H-5^III^, CHHO), 3.80 (dd, 1H, *J* 10.8 Hz, *J* 3.5 Hz, H-6^III^a), 3.83 (dd, 1H, *J* 4.3 Hz, *J* 2.4 Hz, H-4^III^), 3.86 (dd, 1H, *J* 10.8 Hz, *J* 7.6 Hz, H-6^III^b), 3.93 (dd, 1H, *J* 10.6 Hz, *J* 2.3 Hz, H-5^I^a), 4.02 (dt, 1H, *J* 10.3 Hz, *J* 5.0 Hz, CHHO), 4.08 (t, 1H, *J* 2.2 Hz, H-3^III^), 4.16 (dd, 1H, *J* 10.6 Hz, *J* 6.9 Hz, H-5^I^b), 4.20 (dd, 1H, *J* 6.6 Hz, *J* 2.0 Hz, H-2^III^), 4.46 (dd, 1H, *J* 12.4 Hz, *J* 8.3 Hz, H-6^II^a), 4.53–4.60 (m, 3H, H-6^II^b, H-5^II^, H-4^I^), 4.70 (dd, 1H, *J* 3.4 Hz, *J* 1.9 Hz, H-2^II^), 5.06 (d, 1H, *J* 6.6 Hz, H-1^III^), 5.09 (d, 1H, *J* 2.0 Hz, H-1^II^), 5.14 (s, 1H, H-1^I^), 5.53 (dd, 1H, *J* 6.3 Hz, *J* 2.4 Hz, H-3^I^), 5.54 (d, 1H, *J* 2.6 Hz, H-2^I^), 5.78 (t, 1H, *J* 9.8 Hz, H-4^II^), 5.88 (dd, 1H, *J* 10.1 Hz, *J* 3.4 Hz, H-3^II^), 7.16–7.21 (m, 2H, PhCO (H-3, H-5)), 7.26–7.33 (m, 5H, 2 × PhCO (H-3, H-5), PhCO (H-4)), 7.36–7.40 (m, 4H, 2 × PhCO (H-3, H-5)), 7.41–7.48 (m, 2H, 2 × PhCO (H-4)), 7.46–7.52 (m, 1H, PhCO (H-4)), 7.50–7.56 (m, 2H, PhCO (H-4)), 7.87–7.93 (m, 4H, 2 × PhCO (H-2, H-6)), 7.92–7.97 (m, 3H, PhCO (H-2, H-6)), 8.00–8.06 (m, 4H, 2 × PhCO (H-2, H-6)); ^13^C NMR (151 MHz, CDCl_3_): δ 11.9 (((CH_3_)_2_CH)_3_Si), 12.7 (((CH_3_)_2_CH)_3_Si), 12.9 (((CH_3_)_2_CH)_3_Si), 13.0 (((CH_3_)_2_CH)_3_Si), 17.91 (((CH_3_)_2_CH)_3_Si), 17.92 (((CH_3_)_2_CH)_3_Si), 18.1 (((CH_3_)_2_CH)_3_Si), 18.5 (d, *J* 2.3 Hz, ((CH_3_)_2_CH)_3_Si), 18.2 (((CH_3_)_2_CH)_3_Si), 18.3 (((CH_3_)_2_CH)_3_Si), 18.45 (((CH_3_)_2_CH)_3_Si), 18.46 (((CH_3_)_2_CH)_3_Si), 50.7 (CH_2_N_3_), 63.7 (C-6^III^), 65.1 (C-6^II^), 66.6 (CH_2_O), 67.1 (C-5^I^), 68.8 (C-4^II^), 69.2 (C-5^II^), 71.8 (C-2^II^), 72.0 (C-3^II^), 72.5 (C-2^III^), 73.1 (C-4^III^), 77.2 (C-3^I^), 77.5 (C-3^III^), 78.7 (C-5^III^), 80.9 (C-4^I^), 82.6 (C-2^I^), 99.9 (C-1^III^), 100.1 (C-1^II^), 105.9 (C-1^I^), 128.10 (PhCO (C-3, C-5)), 128.12 (PhCO (C-3, C-5)), 128.2 (PhCO (C-3, C-5)), 128.6 (PhCO (C-3, C-5)), 128.4 (PhCO (C-3, C-5)), 129.10 (PhCO (C-1)), 129.13 (PhCO (C-1)), 129.5 (PhCO (C-2, C-6)), 129.8 (PhCO (C-2, C-6)), 129.9 (PhCO (C-2, C-6)), 129.95 (PhCO (C-2, C-6)), 130.00 (PhCO (C-2, C-6)), 132.67 (PhCO (C-4)), 132.71 (PhCO (C-4)), 132.9 (PhCO (C-4)), 133.3 (2 × PhCO (C-4)), 165.5 (CO), 165.58 (CO), 165.60 (CO), 165.9 (CO), 166.0 (CO); ^29^Si INEPT NMR (119 MHz, CDCl_3_): δ 11.5 (TIPS), 12.8 (TIPS), 13.7 (TIPS), 15.6 (TIPS);

Data for conformer B (selected signals):

^1^H NMR (600 MHz, CDCl_3_, 303 K, A:B = 10:3): δ 3.96 (dd, 1H, *J* 10.6 Hz, *J* 2.4 Hz, H-5^I^a), 4.04 (t, 1H, *J* 9.2 Hz, H-6^III^b), 4.10 (d, 1H, *J* 9.2 Hz, H-4^III^), 4.22 (t, 1H, *J* 1.5 Hz, H-2^III^), 4.31 (dd, 1H, *J* 9.3 Hz, *J* 1.5 Hz, H-3^III^), 4.41 (dd, 1H, *J* 12.1 Hz, *J* 8.0 Hz, H-6^II^a), 4.44 (d, 1H, *J* 2.4 Hz, H-2^II^), 4.79 (d, 1H, *J* 1.1 Hz, H-1^III^), 5.17 (s, 1H, H-1^I^), 5.27 (s, 1H, H-1^II^), 5.58 (dd, 1H, *J* 6.5 Hz, *J* 2.5 Hz, H-3^I^), 5.69–5.76 (m, 2H, H-3^II^, H-4^II^); ^13^C NMR (151 MHz, CDCl_3_): δ 11.91 (((CH_3_)_2_CH)_3_Si), 12.90 (((CH_3_)_2_CH)_3_Si), 14.08 (((CH_3_)_2_CH)_3_Si), 14.33 (((CH_3_)_2_CH)_3_Si), 18.05 (((CH_3_)_2_CH)_3_Si), 18.16 (((CH_3_)_2_CH)_3_Si), 18.49 (((CH_3_)_2_CH)_3_Si), 18.52 (((CH_3_)_2_CH)_3_Si), 18.68 (((CH_3_)_2_CH)_3_Si), 18.88 (((CH_3_)_2_CH)_3_Si), 50.69 (CH_2_N_3_), 64.75 (C-6^III^), 64.96 (C-6^II^), 66.62 (CH_2_O), 66.77 (C-5^I^), 68.05 (C-4^II^), 68.79 (C-5^II^), 70.16 (C-4^III^), 72.36 (C-3^II^), 73.78 (C-3^III^), 74.47 (C-2^III^), 75.68 (C-2^II^), 77.03 (C-3^I^), 77.66 (C-5^III^), 80.76 (C-4^I^), 82.70 (C-2^I^), 98.51 (C-1^II^), 102.55 (C-1^III^), 105.93 (C-1^I^);

^1^H NMR (600 MHz, CDCl_3_, 244 K, A:B =10:8.6): δ 0.72–1.32 (m, 168H, 4 × ((CH_3_)_2_CH)_3_Si A, 4 × ((CH_3_)_2_CH)_3_Si B), 3.43–3.57 (m, 3H, CH_2_N_3_ A, CH_2_N_3_ B), 3.65–3.78 (m, 6H, H-6^III^a B, H-5^III^ B, H-5^III^ A, CHHO A, CHHO B, H-6^III^a A), 3.79–3.84 (m, 2H, H-4^III^ A, H-6^III^b A), 3.91 (dd, 1H, *J* 10.9 Hz, *J* 1.8 Hz, H-5^I^a A), 3.93 (dd, 1H, *J* 10.5 Hz, *J* 1.9 Hz, H-5^I^a B), 3.97 (d, 1H, *J* 7.7 Hz, H-6^III^b B), 4.00–4.09 (m, 4H, H-4^III^ B, H-3^III^ A, CHHO A, CHHO B), 4.13–4.17 (m, 2H, H-2^III^ B, H-2^III^ A), 4.17–4.22 (m, 2H, H-5^I^b A, H-5^I^b B), 4.24 (dd, 1H, *J* 9.3 Hz, *J* 2.1 Hz, H-3^III^ B), 4.35 (dd, 1H, *J* 12.1 Hz, *J* 8.6 Hz, H-6^II^a B), 4.42 (dd, 1H, *J* 11.5 Hz, *J* 8.3 Hz, H-6^II^a A), 4.43–4.45 (m, 1H, H-2^II^ B), 4.51–4.61 (m, 6H, H-6^II^b A, H-4^I^ A, H-4^I^ B, H-5^II^ A, H-5^II^ B, H-6^II^b B), 4.61 (dd, 1H, *J* 3.3 Hz, *J* 1.8 Hz, H-2^II^ A), 4.72 (d, 1H, *J* 1.4 Hz, H-1^III^ B), 5.01 (d, 1H, *J* 6.7 Hz, H-1^III^ A), 5.10 (s, 1H, H-1^I^ A), 5.12 (d, 1H, *J* 1.7 Hz, H-1^II^ A), 5.14 (s, 1H, H-1^I^ B), 5.26 (d, 1H, *J* 1.7 Hz, H-1^II^ B), 5.52 (d, 1H, *J* 2.3 Hz, H-2^I^ B), 5.53 (d, 1H, *J* 2.1 Hz, H-2^I^ A), 5.54 (dd, 1H, *J* 6.5 Hz, *J* 2.2 Hz, H-3^I^ A), 5.58 (dd, 1H, *J* 6.6 Hz, *J* 2.0 Hz, H-3^I^ B), 5.69–5.77 (m, 3H, H-3^II^ B, H-4^II^ B, H-4^II^ A), 5.92 (dd, 1H, *J* 10.3 Hz, *J* 3.3 Hz, H-3^II^ A), 7.16–7.59 (m, 30H, 5 × PhCO (H-3, H-4, H-5) A, 5 × PhCO (H-3, H-4, H-5) B), 7.85–8.07 (m, 20H, 5 × PhCO (H-2, H-6) A, 5 × PhCO (H-2, H-6) B); ^13^C NMR (151 MHz, CDCl_3_): δ 11.41 (((CH_3_)_2_CH)_3_Si B), 11.43 (((CH_3_)_2_CH)_3_Si A), 12.2 (((CH_3_)_2_CH)_3_Si A), 12.46 (((CH_3_)_2_CH)_3_Si B), 12.51 (((CH_3_)_2_CH)_3_Si A), 12.7 (((CH_3_)_2_CH)_3_Si A), 14.0 (((CH_3_)_2_CH)_3_Si B), 14.1 (((CH_3_)_2_CH)_3_Si B), 17.7 (((CH_3_)_2_CH)_3_Si B), 17.75 (((CH_3_)_2_CH)_3_Si B), 17.77 (2 × ((CH_3_)_2_CH)_3_Si A), 17.83 (((CH_3_)_2_CH)_3_Si Bi), 17.96 (((CH_3_)_2_CH)_3_Si B), 18.00 (((CH_3_)_2_CH)_3_Si A), 18.04 (((CH_3_)_2_CH)_3_Si A), 18.1 (((CH_3_)_2_CH)_3_Si A), 18.2 (((CH_3_)_2_CH)_3_Si A), 18.27 (((CH_3_)_2_CH)_3_Si A), 18.28 (((CH_3_)_2_CH)_3_Si A), 18.37 (((CH_3_)_2_CH)_3_Si B), 18.40 (((CH_3_)_2_CH)_3_Si B), 18.5 (((CH_3_)_2_CH)_3_Si B), 18.7 (((CH_3_)_2_CH)_3_Si B), 50.28 (CH_2_N_3_ B), 50.32 (CH_2_N_3_ A), 62.9 (C-6^III^ A), 64.2 (C-6^III^ B), 64.63 (C-6^II^ B), 64.7 (C-6^II^ A), 66.3 (C-5^I^ B), 66.6 (C-5^I^ A, CH_2_O A, CH_2_O B), 67.2 (C-4^II^ B), 68.1 (C-4^II^ A), 68.3 (C-5^II^ B), 68.8 (C-5^II^ A), 69.5 (C-4^III^ B), 71.2 (C-3^II^ A), 71.8 (C-2^III^ A), 71.97 (C-2^II^ A), 72.02 (C-3^II^ B), 72.3 (C-4^III^ A), 73.4 (C-3^III^ B), 73.9 (C-2^III^ B), 75.3 (C-2^II^ B), 76.5 (C-3^I^ B), 76.7 (C-3^I^ A), 77.0 (C-5^III^ B), 77.2 (C-3^III^ A), 78.5 (C-5^III^ A), 80.1 (C-4^I^ B), 80.6 (C-4^I^ A), 82.4 (C-2^I^ A), 82.6 (C-2^I^ B), 98.2 (C-1^II^ B), 99.66 (C-1^III^ A), 99.72 (C-1^II^ A), 102.4 (C-1^III^ B), 105.47 (C-1^I^ A), 105.49 (C-1^I^ B), 128.07 (PhCO (C-3, C-5)), 128.12 (2 × PhCO (C-3, C-5)), 128.2 (PhCO (C-3, C-5)), 128.25 (PhCO (C-3, C-5)), 128.30 (3 × PhCO (C-3, C-5)), 128.34 (2 × PhCO (C-3, C-5)), 128.4 (PhCO (C-1)), 128.46 (PhCO (C-1)), 128.48 (PhCO (C-1)), 128.87 (PhCO (C-1)), 128.91 (PhCO (C-1)), 128.93 (PhCO (C-1)), 129.1 (PhCO (C-1)), 129.2 (PhCO (C-1)), 129.3 (PhCO (C-1)), 129.4 (2 × PhCO (C-2, C-6)), 129.5 (PhCO (C-2, C-6)), 129.66 (PhCO (C-2, C-6)), 129.71 (PhCO (C-2, C-6)), 129.73 (PhCO (C-2, C-6)), 129.80 (PhCO (C-2, C-6)), 129.81 (PhCO (C-2, C-6)), 129.84 (PhCO (C-2, C-6)), 129.9 (PhCO (C-2, C-6)), 132.8 (PhCO (C-4)), 132.87 (PhCO (C-4)), 132.91 (PhCO (C-4)), 133.1 (PhCO (C-4)), 133.2 (PhCO (C-4)), 133.3 (PhCO (C-4)), 133.5 × PhCO (C-4)), 165.3 (CO), 165.4 (CO), 165.48 (CO), 165.54 (CO), 165.58 (CO), 165.64 (2 × CO), 165.7 (CO), 166.07 (CO), 166.10 (CO); ^29^Si INEPT NMR (119 MHz, CDCl_3_): δ 11.8 (TIPS A), 11.9 (TIPS B), 12.3 (TIPS B), 12.9 (TIPS B), 13.4 (TIPS A), 13.5 (TIPS B), 14.0 (TIPS A), 15.7 (TIPS A); HRMS (ESI): *m/z* [M+NH_4_]^+^ Calcd for C_90_H_137_N_4_O_20_Si_4_^+^ 1705.8898; Found: 1705.8909.

### 3.9. 2-Azidoethyl 2,3-di-O-Benzoyl-5-O-[3,4,6-tri-O-benzoyl-α-d-mannopyranosyl-2-O-[α-d-mannopyranosyl]-α-d-arabinofuranoside (***25***)

Trisaccharide **24** (30 mg, 0.019 mmol) was dissolved in THF (1 mL) followed by the addition of AcOH (7.5 μL, 0.13 mmol) and 1 M TBAF in THF (275 µL, 0.275 mmol). After stirring at 40 °C for 3.5 h the reaction mixture was concentrated under reduced pressure, co-evaporated with toluene (5 × 5 mL) and dried in vacuo. The residue was purified by silica gel chromatography CH_2_Cl_2_–MeOH (gradient: 0 → 8% MeOH in CH_2_Cl_2_) to give trisaccharide **25** (11 mg, 0.010 mmol, 60%). *R*_f_ = 0.21 (CH_2_Cl_2_–MeOH, 20:1); [α]_D_^26^ +1.7 (c 0.96; CHCl_3_); ^1^H NMR (600 MHz, CD3OD): δ 3.46–3.55 (m, 2H, CH_2_N_3_), 3.65 (t, 1H, *J* 9.6 Hz, H-4^III^), 3.71 (dd, 1H, *J* 11.7 Hz, *J* 6.2 Hz, H-6^III^a), 3.77–3.83 (m, 2H, H-5^III^, CHHO), 3.86 (dd, 1H, *J* 9.5 Hz, *J* 3.4 Hz, H-3^III^), 3.88 (dd, 1H, *J* 11.8 Hz, *J* 2.3 Hz, H-6^III^b), 3.99 (dd, 1H, *J* 3.4 Hz, *J* 1.7 Hz, H-2^III^), 3.98–4.05 (m, 1H, CHHO), 4.05 (dd, 1H, *J* 11.3 Hz, *J* 2.8 Hz, H-5^I^a), 4.24 (dd, 1H, *J* 11.3 Hz, *J* 4.1 Hz, H-5^I^b), 4.39 (dd, 1H, *J* 3.3 Hz, *J* 1.8 Hz, H-2^II^), 4.46 (dd, 1H, *J* 12.0 Hz, *J* 3.5 Hz, H-6^II^a), 4.49 (dd, 1H, *J* 12.0 Hz, *J* 4.5 Hz, H-6^II^b), 4.53–4.59 (m, 2H, H-4^I^, H-5^II^), 4.90 (d, 1H, *J* 1.8 Hz, H-1^III^), 5.37 (d, 2H, *J* 1.9 Hz, H-1^II^), 5.39 (s, 1H, H-1^I^), 5.51 (d, 1H, *J* 1.8 Hz, H-2^I^), 5.72 (dd, 1H, *J* 5.6 Hz, *J* 1.8 Hz, H-3^I^), 5.74 (dd, 1H, *J* 10.1 Hz, *J* 3.2 Hz, H-3^II^), 5.84 (t, 1H, *J* 10.0 Hz, H-4^II^), 7.28–7.46 (m, 11H, 5 × PhCO (H-3, H-5), PhCO (H-4)), 7.46–7.62 (m, 4H, 4 × PhCO (H-4)), 7.82–7.87 (m, 4H, 2 × PhCO (H-2, H-6)), 7.92–7.97 (m, 2H, PhCO (H-2, H-6)), 8.03–8.10 (m, 4H, 2 × PhCO (H-2, H-6)); ^13^C NMR (151 MHz, CD3OD): δ 51.9 (CH_2_N_3_), 63.0 (C-6^III^), 64.6 (C-6^II^), 67.5 (C-5^I^), 67.8 (CH_2_O), 68.6 (C-4^III^), 68.9 (C-4^II^), 70.2 (C-5^II^), 72.1 (C-2^III^), 72.6 (C-3^III^), 73.0 (C-3^II^), 75.6 (C-5^III^), 77.7 (C-2^II^), 78.7 (C-3^I^), 83.1 (C-4^I^), 84.1 (C-2^I^), 100.4 (C-1^II^), 104.2 (C-1^III^), 107.1 (C-1^I^), 129.5 (PhCO (C-3, C-5)), 129.6 (3 × PhCO (C-3, C-5)), 129.7 (PhCO (C-3, C-5)), 130.4 (PhCO (C-1)), 130.48 (PhCO (C-1)), 130.50 (2 × PhCO (C-1)), 130.6 (PhCO (C-2, C-6)), 130.6 (PhCO (C-2, C-6)), 130.7 (PhCO (C-2, C-6)), 130.9 (2 × PhCO (C-2, C-6)), 134.3 (PhCO (C-4)), 134.55 (PhCO (C-4)), 134.60 (2 × PhCO (C-4)), 134.7 (PhCO (C-4)), 166.7 (PhCO), 167.1 (2 × PhCO), 167.6 (2 × PhCO); HRMS (ESI): *m*/*z* [M+NH_4_]^+^ Calcd for C_54_H_57_N_4_O_20_ 1081.3561; Found: 1081.3576.

### 3.10. 2-Azidoethyl 5-O-[α-d-Mannopyranosyl-2-O-(α-d-mannopyranosyl)]-α-d-arabinofuranoside (***26***)

Partially protected trisaccharide (**25**) (11 mg, 0.011 mmol) was dissolved in anhydrous CH_2_Cl_2_ (0.5 mL) and MeOH (1 mL) followed by an addition of 1 M methanolic MeONa (45 μL). The reaction mixture was kept at ~22 °C for 18 h, then neutralized with Dowex 50W × 8 (H+) ion-exchange resin (the resin was washed with MeOH before addition) and then filtered. The filtrate was concentrated under reduced pressure, and the residue was dried in vacuo and purified by reversed phase chromatography on a Sep-Pak C18 cartridge (particle size: 55–105 μm, pore size: 125 Å, sorbent substrate: silica, sorbent weight: 360 mg), gradient: 0 → 100% MeCN in H_2_O). The collected fraction was lyophilized to give deprotected trisaccharide **26** (4.3 mg, 0.0082 mmol, 78%). *R*_f_ = 0.49 (CH_2_Cl_2_–MeOH–H_2_O 40:10:3); [α]_D_^25^ +72.68 (c 1.0, CHCl_3_); ^1^H NMR (600 MHz, CD_3_OD): δ 3.38 (ddd, 2H, *J* 13.3 Hz, J 6.0 Hz, J 3.6 Hz, CH_2_N_3_a), 3.46 (ddd, 3H, *J* 13.3 Hz, J 7.1 Hz, J 3.5 Hz, CH_2_N_3_b), 3.56–3.66 (m, 4H, OCH_2_a, H-5^III^, H-4^II^, H-4^III^), 3.67–3.73 (m, 5H, H-5a^I^, H-6a^II^, H-6a^III^, H-3^III^, H-5^II^), 3.81–3.89 (m, 5H, OCH_2_b, H-5b^I^, H-6b^II^, H-6b^III^, H-3^II^), 3.89–3.92 (m, 2H, H-2^II^, H-3^I^), 3.98 (dd, 1H, *J* 3.3 Hz, J 1.8 Hz, H-2^III^), 4.00 (dd, 1H, *J* 4.1 Hz, J 1.9 Hz, H-2^I^), 4.04 (ddd, 1H, *J* 6.9 Hz, J 5.1 Hz, J 3.2 Hz, H-4^I^), 4.91 (d, 1H, *J* 1.9 Hz, H-1^I^), 4.98 (d, 1H, *J* 1.8 Hz, H-1^III^), 5.14 (d, 1H, *J* 1.8 Hz, H-1^II^); ^13^C NMR (151 MHz, CD_3_OD): δ 51.9 (CH_2_N_3_), 63.0 (C-6^II^), 63.1 (C-6^III^), 67.7 (C-5^I^), 67.9 (OCH_2_), 68.8 (C-4^II^), 69.0 (C-4^III^), 71.9 (C-2^III^), 72.1 (C-3^II^), 72.4 (C-3^III^), 74.7 (C-5^III^), 75.0 (C-5^II^), 78.8 (C-3^I^), 80.3 (C-2^II^), 83.5 (C-4^I^), 83.8 (C-2^I^), 100.3 (C-1^II^), 104.2 (C-1^III^), 109.7 (C-1^I^); HRMS (ESI): *m*/*z* [M+Na]^+^ Calcd for C_19_H_33_N_3_NaO_15_ 566.1804; Found: 566.1806.

## 4. Conclusions

In summary, silylation of phenyl 1-thio-α-d-mannopyranoside, ethyl 1-thio-α- and β-d-mannopyranosides under different conditions was studied. Low-temperature NMR analysis revealed that the silylated products **2**, **5**, **7**, **10**, **13** typically exist as an equilibrium of two chair conformations with a predominance of the axially rich ^1^*C*_4_ conformation. The ring flip was induced by simultaneous introduction of bulky silyl substituents at O-3 and O-4 in mannopyranosides. The dependence of the ratio of conformers on the anomeric configuration, the type of silyl groups and the nature of the aglycone for silylated mannopyranosides was established. The fully silylated phenyl 1-thio-α-d-mannopyranoside (**2**), ethyl 1-thio-α- and β-d-mannopyranosides (**10**, **13**) were involved in the synthesis of α-1,2-linked methyl- and phenyl mannopyranosides (**15**, **18**) and trisaccharide (**22**) with 2-chloroethyl aglycone. In particular, based on the difference in reactivity of (**10**), (**17**) and (**21**), we performed one-pot synthesis of trisaccharide (**22**). In all cases, regardless of conformational preference of silylated thiomannopyranosides (^1^*C*_4_ or ^4^*C*_1_), the formation of only α-linked oligosaccharides also existing as mixtures of conformers was observed. Thus, the use of polysilylated thiomannopyranosides led to the same α-stereoselectivity as in case of the use of mannopyranosyl donors with 2-O-acyl participating group. The exploring of the one-pot methodology could significantly reduce the number of stages.

The obtained deprotected Man*p*-(1→2)-α-d-Man*p-*(1→5)-α-d-Ara*f* trisaccharide 2-azidoethyl glycoside **26**, related to the non-reducing terminal fragment of the *M. tuberculosis* ManLAM, can be used for further preparation of conjugates with proteins to provide antigens, which are important for the development of new tuberculosis screening assays.

## Figures and Tables

**Table 1:** The ^1^*C*_4_ (A) to ^4^*C*_1_ (B) ratio for 1-thio-d-mannopyranosides **2**, **5**, **7**, **10**, **13** and for di- and trisaccharides **15**, **18**, **22**, **24** containing TIPS groups at the mannopyranosyl residues according to ^1^H NMR (600 MHz, CDCl_3_).

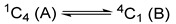	^1^*C*_4_ (A):^4^*C*_1_(B), *T* (K)
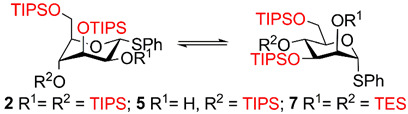	For **2**:10:1.6; 243; For **5**:1:0; 298; For **7**:10:1.4; 303; 1:1, 240
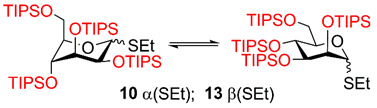	For **10**:10:3.4; 240; For **13**:1:0; 298; 1:0, 240
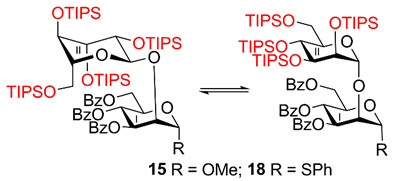	For **15**:10:3, 298; 10:7.6; 240; For **18**:1:0; 303; 10:3.4; 240
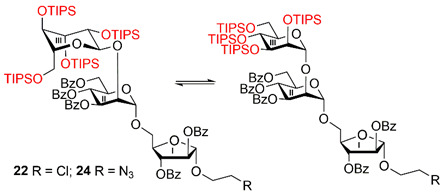	For **22**:10:3, 303; 10:8.6, 236; For **24**:10:3, 303; 10:8.6, 244

**Table 2:** ^1^H NMR data for super-armed thiomannopyranosides **2**, **5**, **7**, **10**, **13** (600 MHz, CDCl_3_, δ_H_ (ppm)/*J* (Hz)).

Donor (*T* (K))	H-1 δ_H_/*J*_1,2_	H-2 δ_H_/*J*_2,3_	H-3 δ_H_/*J*_3,4_	H-4 δ_H_/*J*_4,5_	H-5 δ_H_/*J*_5,6_ or *J*_5,4_	H-6a δ_H_/*J*_6a,6b_ or *J*_6a,5_	H-6b δ_H_/*J*_6b,6a_ or *J*_6b,5_
**2** (323)	5.40, 7.7	4.20–4.24	4.15–4.19	3.90/<1	3.96–4.01	3.86/10.7, 7.6	3.92–3.97
**2** (298)	5.38/7.6	4.17–4.24	4.11–4.16	3.84–3.90	3.94–4.01	3.85/10.7, 7.6	3.90–3.94
**2A** (243)	5.35/8.5	4.14/2.0	4.09/2.2	3.82–3.87	3.94/4.6, 3.2	3.82–3.87	3.88/10.8, 7.4
**2B** (243)	5.34–5.35	4.33/1.8 ^a^	3.94–3.97	4.06–4.09	4.11–4.14	3.77/10.0, 8.5	4.04
**5** (298)	5.06/9.3	3.83/2.9, 4.9 (OH)	4.23/3.3 ^a^	4.08/1.6	3.97–4.01	3.99/12.4, 5.8	4.08/8.8
**7A** (240)	5.31/8.8	3.98/2.2	3.91–3.94	3.77–3.86	3.77–3.86	3.77–3.86	3.77–3.86
**7B** (240)	5.32/1.4	4.21/1.9 ^a^	3.91–3.94	4.05/9.3, 8.6	3.99–4.03	3.77–3.86	3.94–3.96
**10A** (240)	5.12/8.5	4.08/1.9	4.07/2.3	3.79–3.85	3.79–3.85	3.79–3.85	3.87/11.2, 1.9
**10B** (240)	5.19/1.4	4.14/1.5	4.01–4.05	4.01–4.05	3.93/10.6, 5.8	3.70/10.1, 8.7	4.00/1.8
**13** (298)	5.14/5.7	4.59/2.6	4.16/3.7	4.26/<1	3.83	4.18/10.4, 4.8	4.61/8.6
**13** (240)	5.11/5.7	4.54/2.4	4.10/3.8	4.22/<1	3.79	4.05/10.3, 4.6	4.63/9.2

^a^ An apparent coupling constant (*J*_app_) is shown.

**Table 3:** ^13^C NMR data for super-armed thiomannopyranosides **2**, **5**, **7**, **10**, **13** (151 MHz, CDCl_3_, δ_C_ (ppm).

Donor (*T* (K))	C-1	C-2	C-3	C-4	C-5	C-6
**2** (323)	88.6	72.4	77.3	73.1	77.7	65.1
**2** (298)	88.3	71.8	77.1	73.0	77.6	64.7
**2A** (243)	87.4	71.1	76.5	72.3	77.9	63.7
**2B** (243)	88.9	75.6	74.9	69.9	76.6	63.8
**5** (298)	82.2	67.0	73.5	71.1	82.0	61.9
**7A** (240)	86.0	70.2	76.2	71.2	78.3	62.9 or 63.0
**7B** (240)	88.9	75.0	74.4	68.7	75.9	62.9 or 63.0
**10A** (240)	80.8	71.2	76.5	72.4	78.5	63.1
**10B** (240)	82.9	75.3	75.0	70.0	76.0	63.7
**13** (298)	84.7	68.0	74.3	72.1	81.1	65.0
**13** (240)	84.3	67.4	73.8	71.3	80.7	64.4

**Table 4:** ^1^H NMR data for di- and trisaccharides **15**, **18**, **22**, **24** containing mannopyranosyl residues with TIPS groups at the non-reducing end (600 MHz, CDCl_3_, δ_H_ (ppm).

Residue (*T* (K))	H-1 δ_H_/*J*_1,2_	H-2 δ_H_/*J*_2,3_	H-3 δ_H_/*J*_3,4_	H-4 δ_H_/*J*_4,5_	H-5 δ_H_/*J*_5,6_ or *J*_5,4_	H-6a δ_H_/*J*_6a,6b_ or *J*_6a,5_	H-6b δ_H_/*J*_6b,6a_ or *J*_6b,5_
**15A**^II^ (298)	5.05/6.5	4.16/6.5, 2.0	4.06/2.2 ^a^	3.76/4.6, 2.3	3.70/7.8, 4.6, 3.2	3.80/10.7, 3.3	3.85/10.7, 8.0
**15B**^II^ (298)	4.76/1.8	4.21/1.9 ^a^	4.27–4.34	4.09/9.3 ^a^	3.67–3.70	3.72–3.76	4.06/10.1
**15A**^II^ (240)	4.98/6.6	4.12/1.8	3.95–4.05	3.71/4.8, 2.2	3.65–3.70	3.74/10.9, 3.6	3.81/10.7, 8.1
**15B**^II^ (240)	4.68/1.2	4.14/2.3	4.23/9.3, 1.8	3.95–4.05	3.65–3.70	3.65–3.70	3.95–4.05
**18A**^II^ (303)	5.05/6.8	4.21/6.8, 1.9	4.11/<1	3.94/3.1	3.77/6.9, 3.6	3.92/10.6, 6.8	3.80/10.7, 4.0
**18A**^II^ (240)	4.96/7.0	4.15/7.1, 1.9	4.04/2.2 ^a^	3.93/3.0 ^a^	3.69–3.74	3.76/10.6, 4.6	3.86/10.8, 6.5
**18B**^II^ (240)	4.72/<1	4.12–4.13	4.23/9.4, 1.7	4.07/9.3 ^a^	3.63/8.3 ^a^	3.69–3.74	3.81–3.85
**22A**^III^ (303)	5.06/6.6	4.20/6.6, 2.0	4.08/2.2	3.82–3.89	3.70–3.77	3.80/10.8, 3.5	3.82–3.89
**22B**^III^ (303)	4.79/<1	4.22/1.9 ^a^	4.32/9.1, 1.9	4.10/9.2 ^a^	n.d	n.d	n.d
**22A**^III^ (236)	5.00/6.9	4.12–4.21	3.98–4.04	3.77–3.98	3.63–3.71	3.74/10.7, 3.7	3.77–3.98
**22B**^III^ (236)	4.71/<1	4.12–4.21	4.23/9.2	3.98–4.04	3.63–3.71	3.63–3.71	3.77–3.98
**24A**^III^ (303)	5.06/6.6	4.20/6.6, 2.0	4.08/2.2	3.83/4.3, 2.4	3.69–3.77	3.80/10.8, 3.5	3.86/10.8, 7.6
**24B**^III^ (303)	4.79/1.1	4.22/1.5	4.31/9.3, 1.5	4.10/9.2	n.d	n.d	4.04/9.2
**24A**^III^ (244)	5.01/6.7	4.13–4.17	4.00–4.09	3.79–3.84	3.65–3.78	3.65–3.78	3.79–3.84
**24B**^III^ (244)	4.72/1.4	4.13–4.17	4.24/9.3, 2.1	4.00–4.09	3.65–3.78	3.65–3.78	3.97/7.7

^a^ An apparent coupling constant (*J*_app_) is shown.

**Table 5:** ^13^C NMR data for di- and trisaccharides **15**, **18**, **22**, **24** containing mannopyranosyl residues with TIPS groups at the non-reducing end (151 MHz, CDCl_3_, δ_C_ (ppm).

Residue (*T* (K))	C-1	C-2	C-3	C-4	C-5	C-6
**15A**^II^ (298)	99.9	72.6	77.5	73.2	78.4	64.0
**15B**^II^ (298)	102.4	74.4	73.8	70.1	77.7	64.7
**15A**^II^ (240)	99.7	72.0	77.2	72.6	78.0	63.2
**15B**^II^ (240)	102.2	73.7	73.5	69.5	77.1	64.2
**18A**^II^ (303)	99.69	72.3	77.3	72.8	79.5	63.2
**18A**^II^ (240)	99.67	71.4	77.0	71.8	79.1	62.2
**18**^II^ (240)	102.9	73.7	73.5	69.1	77.0	63.5
**22A**^III^ (303)	99.9	72.5	77.5	73.1	77.5	63.7
**22B**^III^ (303)	102.5	74.5	73.8	70.2	77.7	64.8
**22A**^III^ (236)	99.6	71.7	77.1	72.3	78.5	62.8
**22B**^III^ (236)	102.3	73.8	73.4	69.4	77.0	64.1
**24A**^III^ (303)	99.9	72.5	77.5	73.1	78.7	63.7
**24B**^III^ (303)	102.6	74.5	73.8	70.2	77.7	64.8
**24A**^III^ (244)	99.7	71.8	77.2	72.3	78.5	62.9
**24B**^III^ (244)	102.4	73.9	73.4	69.5	77.0	64.2

## Data Availability

The original contributions presented in this study are included in the article/Supplementary Materials. Further inquiries can be directed to the corresponding author.

## Supplementary Materials

The following supporting information can be downloaded at: https://www.mdpi.com/article/10.3390/molecules31101598/s1: Copies of NMR spectra.

## References

[1] World Health Organization Tuberculosis. https://www.who.int/en/news-room/fact-sheets/detail/tuberculosis.

[2] Ravesloot-Chávez M.M., Van Dis E., Stanley S.A. (2021). The Innate Immune Response to *Mycobacterium tuberculosis* Infection. Annu. Rev. Immunol..

[3] Banoo S., Yadav Y., Tyagi R., Manna A., Sagar R. (2024). Recent efforts in the development of glycoconjugate vaccine and available treatment for tuberculosis. Bioorg. Chem..

[4] Angala S.K., Palcekova Z., Belardinelli J.M., Jackson M. (2018). Covalent modifications of polysaccharides in mycobacteria. Nat. Chem. Biol..

[5] Kang P.B., Azad A.K., Torrelles J.B., Kaufman T.M., Beharka A., Tibesar E., DesJardin L.E., Schlesinger L.S. (2005). The human macrophage mannose receptor directs *Mycobacterium tuberculosis* lipoarabinomannan-mediated phagosome biogenesis. J. Exp. Med..

[6] Zheng R.B., Jegouzo S.A.F., Joe M., Bai Y., Tran H.A., Shen K., Saupe J., Xia L., Ahmed M.F., Liu Y.H. (2017). Insights into Interactions of Mycobacteria with the Host Innate Immune System from a Novel Array of Synthetic Mycobacterial Glycans. ACS Chem. Biol..

[7] Brock M., Hanlon D., Zhao M., Pollock N.R. (2020). Detection of mycobacterial lipoarabinomannan in serum for diagnosis of active tuberculosis. Diagn. Microbiol. Infect. Dis..

[8] Bernardini R., Tengattini S., Li Z., Piubelli L., Bavaro T., Modolea A.B., Mattei M., Conti P., Marini S., Zhang Y. (2024). Effect of glycosylation on the affinity of the MTB protein Ag85B for specific antibodies: Towards the design of a dual-acting vaccine against tuberculosis. Biol. Direct.

[9] Liu Y., Brown C.M., Erramilli S., Su Y.C., Guu S.Y., Tseng P.S., Wang Y.J., Duong N.H., Tokarz P., Kloss B. (2025). Structural insights into terminal arabinosylation of mycobacterial cell wall arabinan. Nat. Commun..

[10] Mereyala H.B., Hotha S., Gurjar M.K. (1998). Synthesis of pentaarabinofuranosyl structure motif A of *Mycobacterium tuberculosis*. Chem. Commun..

[11] Ishiwata A., Akao H., Ito Y. (2006). Stereoselective synthesis of a fragment of mycobacterial arabinan. Org. Lett..

[12] Joe M., Bai Y., Nacario R.C., Lowary T.L. (2007). Synthesis of the docosanasaccharide arabinan domain of mycobacterial arabinogalactan and a proposed octadecasaccharide biosynthetic precursor. J. Am. Chem. Soc..

[13] Sahloul K., Lowary T.L. (2015). Development of an Orthogonal Protection Strategy for the Synthesis of Mycobacterial Arabinomannan Fragments. J. Org. Chem..

[14] Islam M., Gayatri G., Hotha S. (2015). Influence of Steric Crowding on Diastereoselective Arabinofuranosylations. J. Org. Chem..

[15] Lowary T.L. (2016). Twenty years of mycobacterial glycans: Furanosides and beyond. Acc. Chem. Res..

[16] Mishra B., Manmode S., Panda R.R.A., Hotha S. (2017). Expedient Synthesis of a Linear Nonadecaarabinofuranoside of the *Mycobacterium tuberculosis* Cellular Envelope. Eur. J. Org. Chem..

[17] Wu Y., Xiong D.C., Chen S.C., Wang Y.S., Ye X.S. (2017). Total synthesis of mycobacterial arabinogalactan containing 92 monosaccharide units. Nat. Commun..

[18] Pasari S., Manmode S., Walke G., Hotha S. (2018). A Versatile Synthesis of Pentacosafuranoside Subunit Reminiscent of Mycobacterial Arabinogalactan Employing One Strategic Glycosidation Protocol. Chem. Eur. J..

[19] Liu K., Wang L., Guo Z. (2019). An extensive review of studies on mycobacterium cell wall polysaccharide-related oligosaccharides—Part III: Synthetic studies and biological applications of arabinofuranosyl oligosaccharides and their analogs, derivatives and conjugates. J. Carbohydr. Chem..

[20] Wang L., Guo Z. (2019). An extensive review of studies on mycobacterium cell wall polysaccharide-related oligosaccharides—Part I: Synthetic studies on arabinofuranosyl oligosaccharides. J. Carbohydr. Chem..

[21] Sweeney R.P., Lowary T.L., Barchi J.J., Vidal S. (2021). 2.08—Glycosylation with Furanosides. Comprehensive Glycoscience.

[22] Yao W., Xiong D.-C., Yang Y., Geng C., Cong Z., Li F., Li B.-H., Qin X., Wang L.-N., Xue W.-Y. (2022). Automated Solution-Phase Multiplicative Synthesis of Complex Glycans Up to a 1,080-mer. Nat. Synth..

[23] Gao J., Liao G.C., Wang L.Z., Guo Z.W. (2014). Synthesis of a Miniature Lipoarabinomannan. Org. Lett..

[24] Islam M., Shinde G.P., Hotha S. (2017). Expedient synthesis of the heneicosasaccharyl mannose capped arabinomannan of the *Mycobacterium tuberculosis* cellular envelope by glycosyl carbonate donors. Chem. Sci..

[25] Wang L., Feng S., Wang S., Li H., Guo Z., Gu G. (2017). Synthesis and immunological comparison of differently linked lipoarabinomannan oligosaccharide–monophosphoryl Lipid A conjugates as antituberculosis vaccines. J. Org. Chem..

[26] Pardo-Vargas A., Bharate P., Delbianco M., Seeberger P.H. (2019). Automated glycan assembly of arabinomannan oligosaccharides from *Mycobacterium tuberculosis*. Beilstein J. Org. Chem..

[27] Han L., Wang L., Guo Z. (2019). An extensive review of studies on mycobacterium cell wall polysaccharide-related oligosaccharides—Part II: Synthetic studies on complex arabinofuranosyl oligosaccharides carrying other functional motifs and related derivatives and analogs. J. Carbohydr. Chem..

[28] Li Z.H., Bavaro T., Tengattini S., Bernardini R., Mattei M., Annunziata F., Cole R.B., Zheng C.P., Sollogoub M., Tamborini L. (2020). Chemoenzymatic synthesis of arabinomannan (AM) glycoconjugates as potential vaccines for tuberculosis. Eur. J. Med. Chem..

[29] Wang L., Feng S., An L., Gu G., Guo Z. (2015). Synthetic and immunological studies of mycobacterial lipoarabinomannan oligosaccharides and their protein conjugates. J. Org. Chem..

[30] Bols M., Frihed T.G., Pedersen M.J., Pedersen C.M. (2022). Silylated Sugars—Synthesis and Properties. Synlett.

[31] Pedersen C.M., Nordstrøm L.U., Bols M. (2007). “Super armed” glycosyl donors: Conformational arming of thioglycosides by silylation. J. Am. Chem. Soc..

[32] Abronina P.I., Malysheva N.N., Stepanova E.V., Shvyrkina J.S., Zinin A.I., Kononov L.O. (2022). Five triisopropylsilyl substituents in Ara-β-(1→2)-Ara disaccharide glycosyl donor make unselective glycosylation reaction stereoselective. Eur. J. Org. Chem..

[33] Abronina P.I., Malysheva N.N., Zinin A.I., Kolotyrkina N.G., Kononov L.O. (2024). Synthesis of hexaarabinofuranoside bearing the 4-(3-azidopropoxy)phenyl aglycone related to the terminal fragment of polysaccharides of mycobacteria. Russ. Chem. Bull..

[34] Abronina P.I., Malysheva N.N., Karpenko M.Y., Novikov D.S., Zinin A.I., Kolotyrkina N.G., Kononov L.O. (2025). A Rational Synthesis of a Branched Decaarabinofuranoside Related to the Fragments of Mycobacterial Polysaccharides. Molecules.

[35] Abronina P.I., Novikov D.S., Malysheva N.N., Zinin A.I., Chizhov A.O., Kononov L.O. (2024). Stereocontrolled 1,2-*trans*-arabinofuranosylation in the absence of 2-*O*-acyl group in glycosyl donor. Carbohydr. Res..

[36] Abronina P.I., Novikov D.S., Malysheva N.N., Zinin A.I., Kolotyrkina N.G., Kononov L.O. (2025). Application of Ara-β-(1→2)-Ara diarabinofuranosides with O-TBDPS protective groups in the synthesis of the tetra- and dodecaarabinofuranosides—Fragments of mycobacterial polysaccharides. Carbohydr. Res..

[37] Abronina P.I., Novikov D.S., Zinin A.I., Kononov L.O. (2026). Synthesis of the linear octaarabinofuranoside related to the terminal fragment of mycobacterial polysaccharides. Mendeleev Commun..

[38] Korolyova-Ushakova A.G., Baranova E.V., Ignatov S.G., Soloviev P.V., Kondakov N.N., Mel’nikova T.M., Abronina P.I., Podval’nyi N.M., Kononov L.O., Biketov S.F. (2019). Comparative Characteristics of the Diagnostic Potential of Mycobacterial Synthetic Antigens for the Seroriagnosis of Lepra and Tuberculosis. Appl. Biochem. Microbiol..

[39] Contour M.-O., Defaye J., Little M., Wong E. (1989). Zirconium(IV) chloride-catalyzed synthesis of 1,2-*trans*-1-thioglycopyranosides. Carbohydr. Res..

[40] Yu H.N., Ling C.-C., Bundle D.R. (2002). Synthesis of three *Salmonella* epitopes for biosensor studies of carbohydrate–antibody interactions. Can. J. Chem..

[41] Reina J.J., Di Maio A., Ramos-Soriano J., Figueiredo R.C., Rojo J. (2016). Rapid and efficient synthesis of α(1–2)mannobiosides. Org. Biomol. Chem..

[42] Amin M.N., Huang W., Mizanur R.M., Wang L.-X. (2011). Convergent Synthesis of Homogeneous Glc1Man9GlcNAc2-Protein and Derivatives as Ligands of Molecular Chaperones in Protein Quality Control. J. Am. Chem. Soc..

[43] Podvalnyy N.M., Sedinkin S.L., Abronina P.I., Zinin A.I., Fedina K.G., Torgov V.I., Kononov L.O. (2011). Ring opening of acylated β-D-arabinofuranose 1,2,5-orthobenzoates with nucleophiles allows access to novel selectively-protected arabinofuranose building blocks. Carbohydr. Res..

[44] Armarego W.L.F. (2017). Purification of Laboratory Chemicals.

