# Supplementary material for: Super-Armed Thiomannopyranosides in the Synthesis of a Mannose-Capped Trisaccharide of Mycobacterium tuberculosis Lipoarabinomannan
Source: Molecules. 2026 May 10;31(10):1598. doi: 10.3390/molecules31101598 (PMC13209574; doi:10.3390/molecules31101598)

Supplementary data

Super-armed thiomannopyranosides in the synthesis of a mannose-capped trisaccharide of *Mycobacterium tuberculosis* lipoarabinomannan

Polina Igorevna Abronina *, Zinaida Vladimirovna Kuznetsova, Dmitry Sergeevich Novikov, Alexander Ivanovich Zinin, Natalya G. Georgievna Kolotyrkina and Leonid Olegovich Kononov *

N.D. Zelinsky Institute of Organic Chemistry, Russian Academy of Sciences, Leninsky prosp., 47, Moscow 119991, Russia

* Correspondence: polina-abronina@yandex.ru (P.I.A.); leonid.kononov@gmail.com (L.O.K.)

Table of Contents

Phenyl 2,3,4,6-tetrakis-*O*-(triisopropylsilyl)-1-thio-α-D-mannopyranoside (2, $CDCl_3$, 243K)

^{1}H NMR spectrum of compound **2** S7

^{13}C NMR spectrum of compound **2** S8

COSY ^{1}H-^{1}H spectrum of compound **2** S9

HSQC ^{1}H-^{13}C spectrum of compound **2** S10

2, 3, 6-tri-*O*-(triisopropylsilyl)-1-thio-α-D-mannopyranoside (3, C_6D_6)

^{1}H NMR spectrum of compound **3** S11

^{13}C NMR spectrum of compound **3** S12

COSY ^{1}H-^{1}H spectrum of compound **3** S13

HSQC ^{1}H-^{13}C spectrum of compound **3** S14

HMBC ^{1}H-^{13}C spectrum of compound **3** S15

HMBC ^{1}H-^{29}Si spectrum of compound **3** S16

^{29}Si INEPT NMR spectrum of compound **3** S17

Phenyl 2, 4, 6-tri-*O*-(triisopropylsilyl)-1-thio-α-D-mannopyranoside (4, C_6D_6)

^{1}H NMR spectrum of compound **4** S18

^{13}C NMR spectrum of compound **4** S19

COSY ^{1}H-^{1}H spectrum of compound **4** S20

HSQC ^{1}H-^{13}C spectrum of compound **4** S21

HMBC ^{1}H-^{13}C spectrum of compound **4** S22

HMBC ^{1}H-^{29}Si spectrum of compound **4** S23

29Si INEPT NMR spectrum of compound **4** S24

Phenyl 3,4,6-tri-*O*-(triisopropylsilyl)-1-thio-α-D-mannopyranoside (5)

^{1}H NMR spectrum of compound **5** S25

^{13}C NMR spectrum of compound **5** S26

COSY ^{1}H-^{1}H spectrum of compound **5** S27

HSQC ^{1}H-^{13}C spectrum of compound **5** S28

HMBC ^{1}H-^{13}C spectrum of compound **5** S29

HMBC ^{1}H-^{29}Si spectrum of compound **5** S30

^{29}Si INEPT NMR spectrum of compound **5** S31

Phenyl 3,6-bis-*O*-(triisopropylsilyl)-1-thio-α-D-mannopyranoside (6)

^{1}H NMR spectrum of compound **6** S32

^{13}C NMR spectrum of compound **6** S33

COSY ^{1}H-^{1}H spectrum of compound **6** S34

HSQC ^{1}H-^{13}C spectrum of compound **6** S35

HMBC ^{1}H-^{13}C spectrum of compound **6** S36

Phenyl 3,6-bis-*O*-(triisopropylsilyl)-2,4-bis-*O*-(triethylsilyl)-1-thio-α-D-mannopyranoside (7, $CDCl_3$, 240K)

^{1}H NMR spectrum of compound **7** S37

^{13}C NMR spectrum of compound **7** S38

COSY ^{1}H-^{1}H spectrum of compound **7** S39

HSQC ^{1}H-^{13}C spectrum of compound **7** S40

HMBC ^{1}H-^{13}C spectrum of compound **7** S41

HMBC ^{1}H-^{29}Si spectrum of compound **7** S42

Ethyl 2, 3, 4, 6-tetrakis-*O*-(triisopropylsilyl)-1-thio-α-D-mannopyranoside (10)

^{1}H NMR spectrum of compound **10** S43

^{13}C NMR spectrum of compound **10** S44

COSY ^{1}H-^{1}H spectrum of compound **10** S45

HSQC ^{1}H-^{13}C spectrum of compound **10** S46

HMBC ^{1}H-^{13}C spectrum of compound **10** S47

HMBC ^{1}H-^{29}Si spectrum of compound **10** S48

^{29}Si INEPT NMR spectrum of compound **10** S49

Ethyl 2, 3 ,6-tri-*O*-(triisopropylsilyl)-1-thio-α-D-mannopyranoside (11)

^{1}H NMR spectrum of compound **11** S50

^{13}C NMR spectrum of compound **11** S51

COSY ^{1}H-^{1}H spectrum of compound **11** S52

HSQC ^{1}H-^{13}C spectrum of compound **11** S53

HMBC ^{1}H-^{13}C spectrum of compound **11** S54

Ethyl 2,4,6-tri-*O*-(triisopropylsilyl)-1-thio-α-D-mannopyranoside (12)

^{1}H NMR spectrum of compound **12** S55

^{13}C NMR spectrum of compound **12** S56

COSY ^{1}H-^{1}H spectrum of compound **12** S57

HSQC ^{1}H-^{13}C spectrum of compound **12** S58

HMBC ^{1}H-^{13}C spectrum of compound **12** S59

Ethyl 2, 3, 4, 6-tetrakis-*O*-(triisopropylsilyl)-1-thio-β-D-mannopyranoside (13)

^{1}H NMR spectrum of compound **13** S60

^{13}C NMR spectrum of compound **13** S61

COSY ^{1}H-^{1}H spectrum of compound **13** S62

HSQC ^{1}H-^{13}C spectrum of compound **13** S63

HMBC ^{1}H-^{13}C spectrum of compound **13** S64

Methyl 2-*O*-[2,3,4,6-tetrakis-*O*-(triisopropylsilyl)-α-D-mannopyranosyl]-3,4,6-tri-*O*-benzoyl-1-thio-α-D-mannopyranoside (15, $CDCl_3$, 298K)

^{1}H NMR spectrum of compound **15** S65

^{13}C NMR spectrum of compound **15** S66

COSY ^{1}H-^{1}H spectrum of compound **15** S67

HSQC ^{1}H-^{13}C spectrum of compound **15** S68

HMBC ^{1}H-^{13}C spectrum of compound **15** S69

HMBC ^{1}H-^{29}Si spectrum of compound **15** S70

^{29}Si INEPT NMR spectrum of compound **15** S71

Methyl 2-*O*-[2,3,4,6-tetrakis-*O*-(triisopropylsilyl)-α-D-mannopyranosyl]-3,4,6-tri-*O*-benzoyl-1-thio-α-D-mannopyranoside (15, $CDCl_3$, 240K)

^{1}H NMR spectrum of compound **15** S72

^{13}C NMR spectrum of compound **15** S73

COSY ^{1}H-^{1}H spectrum of compound **15** S74

HSQC ^{1}H-^{13}C spectrum of compound **15** S75

HMBC ^{1}H-^{13}C spectrum of compound **15** S76

HMBC ^{1}H-^{29}Si spectrum of compound **15** S77

^{29}Si INEPT NMR spectrum of compound **15** S78

Methyl 2-*O*-(α-D-mannopyranosyl)-3,4,6-tri-*O*-benzoyl-1-thio-α-D-mannopyranoside (16)

^{1}H NMR spectrum of compound **16** S79

^{13}C NMR spectrum of compound **16** S80

COSY ^{1}H-^{1}H spectrum of compound **16** S81

HSQC ^{1}H-^{13}C spectrum of compound **16** S82

HMBC ^{1}H-^{13}C spectrum of compound **16** S83

Phenyl 2-*O*-[2,3,4,6-tetrakis-*O*-(triisopropylsilyl)- α-D-mannopyranosyl]-3,4,6-tri-*O*-benzoyl-1-thio-α-D-mannopyranoside (18, $CDCl_3$, 303K)

^{1}H NMR spectrum of compound **18** S84

^{13}C NMR spectrum of compound **18** S85
COSY ^{1}H-^{1}H spectrum of compound **18** S86
HSQC ^{1}H-^{13}C spectrum of compound **18** S87
HMBC ^{1}H-^{13}C spectrum of compound **18** S88
^{29}Si INEPT NMR spectrum of compound **18** S89

Phenyl 2-*O*-[2,3,4,6-tetrakis-*O*-(triisopropylsilyl)- α-D-mannopyranosyl]-3,4,6-tri-*O*-benzoyl-1-thio-α-D-mannopyranoside (18, $CDCl_3$, 240K)

^{1}H NMR spectrum of compound **18** S90
^{13}C NMR spectrum of compound **18** S91
COSY ^{1}H-^{1}H spectrum of compound **18** S92
HSQC ^{1}H-^{13}C spectrum of compound **18** S93
HMBC ^{1}H-^{13}C spectrum of compound **18** S94
^{29}Si INEPT NMR spectrum of compound **18** S95

2-Chloroethyl 2,3-di-*O*-benzoyl-5-*O*-[3,4,6-tri-*O*-benzoyl- α-D-mannopyranosyl-2-O-{2,3,4,6-tetrakis-*O*-(triisopropylsilyl)-α-D-mannopyranosyl}]-α-D-arabinofuranoside (24, $CDCl_3$, 303K)

^{1}H NMR spectrum of compound **22** S96
^{13}C NMR spectrum of compound **22** S97
COSY ^{1}H-^{1}H spectrum of compound **22** S98
HSQC ^{1}H-^{13}C spectrum of compound **22** S99
HMBC ^{1}H-^{13}C spectrum of compound **22** S100
^{29}Si INEPT NMR spectrum of compound **22** S101

2-Chloroethyl 2,3-di-*O*-benzoyl-5-*O*-[3,4,6-tri-*O*-benzoyl- α-D-mannopyranosyl-2-O-{2,3,4,6-tetrakis-*O*-(triisopropylsilyl)-α-D-mannopyranosyl}]-α-D-arabinofuranoside (24, $CDCl_3$, 236K)

^{1}H NMR spectrum of compound **22** S102
^{13}C NMR spectrum of compound **22** S103
COSY ^{1}H-^{1}H spectrum of compound **22** S104
HSQC ^{1}H-^{13}C spectrum of compound **22** S105
HMBC ^{1}H-^{13}C spectrum of compound **22** S106
^{29}Si INEPT NMR spectrum of compound **22** S107

2-chloroethyl 2,3-di-*O*-benzoyl-5-*O*-(3,4,6-tri-*O*-benzoyl-α-D-mannopyranosyl)-α-D-arabinofuranoside (23)

^{1}H NMR spectrum of compound **23** S108
^{13}C NMR spectrum of compound **23** S109
COSY ^{1}H-^{1}H spectrum of compound **23** S110
HSQC ^{1}H-^{13}C spectrum of compound **23** S111
HMBC ^{1}H-^{13}C spectrum of compound **23** S112

2-Azidoethyl 2,3-di-*O*-benzoyl-5-*O*-[3,4,6-tri-*O*-benzoyl-α-D-mannopyranosyl-2-*O*-{2,3,4,6-tetrakis-*O*-(triisopropylsilyl)- α-D-mannopyranosyl}]-α-D-arabinofuranoside (24, $CDCl_3$, 303K)

^{1}H NMR spectrum of compound **24** S113

^{13}C NMR spectrum of compound **24** S114

COSY ^{1}H-^{1}H spectrum of compound **24** S115

HSQC ^{1}H-^{13}C spectrum of compound **24** S116

HMBC ^{1}H-^{13}C spectrum of compound **24** S117

^{29}Si INEPT NMR spectrum of compound **24** S118

2-Azidoethyl 2,3-di-*O*-benzoyl-5-*O*-[3,4,6-tri-*O*-benzoyl-α-D-mannopyranosyl-2-*O*-{2,3,4,6-tetrakis-*O*-(triisopropylsilyl)- α-D-mannopyranosyl}]-α-D-arabinofuranoside (24, $CDCl_3$, 244K)

^{1}H NMR spectrum of compound **24** S119

^{13}C NMR spectrum of compound **24** S120

COSY ^{1}H-^{1}H spectrum of compound **24** S121

HSQC ^{1}H-^{13}C spectrum of compound **24** S122

HMBC ^{1}H-^{13}C spectrum of compound **24** S123

^{29}Si INEPT NMR spectrum of compound **24** S124

2-Azidoethyl 2,3-di-*O*-benzoyl-5-*O*-[3,4,6-tri-*O*-benzoyl-α-D-mannopyranosyl-2-*O*-[-D-mannopyranosyl]-α-D-arabinofuranoside (25)

^{1}H NMR spectrum of compound **25** S125

^{13}C NMR spectrum of compound **25** S126

COSY ^{1}H-^{1}H spectrum of compound **25** S127

HSQC ^{1}H-^{13}C spectrum of compound **25α** S128

HMBC ^{1}H-^{13}C spectrum of compound **25** S129

2-Azidoethyl 5-*O*-[α-D-mannopyranosyl-2-*O*-(α-D-mannopyranosyl)]-α-D-arabinofuranoside

^{1}H NMR spectrum of compound **25** S130

^{13}C NMR spectrum of compound **25** S131

COSY ^{1}H-^{1}H spectrum of compound **25** S132

HSQC ^{1}H-^{13}C spectrum of compound **25** S133

HMBC ^{1}H-^{13}C spectrum of compound **25** S134

^{1}H NMR (600 MHz) spectrum of compound 2 in $CDCl_3$ (243K)

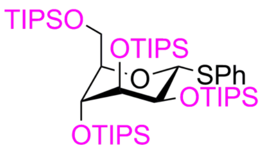

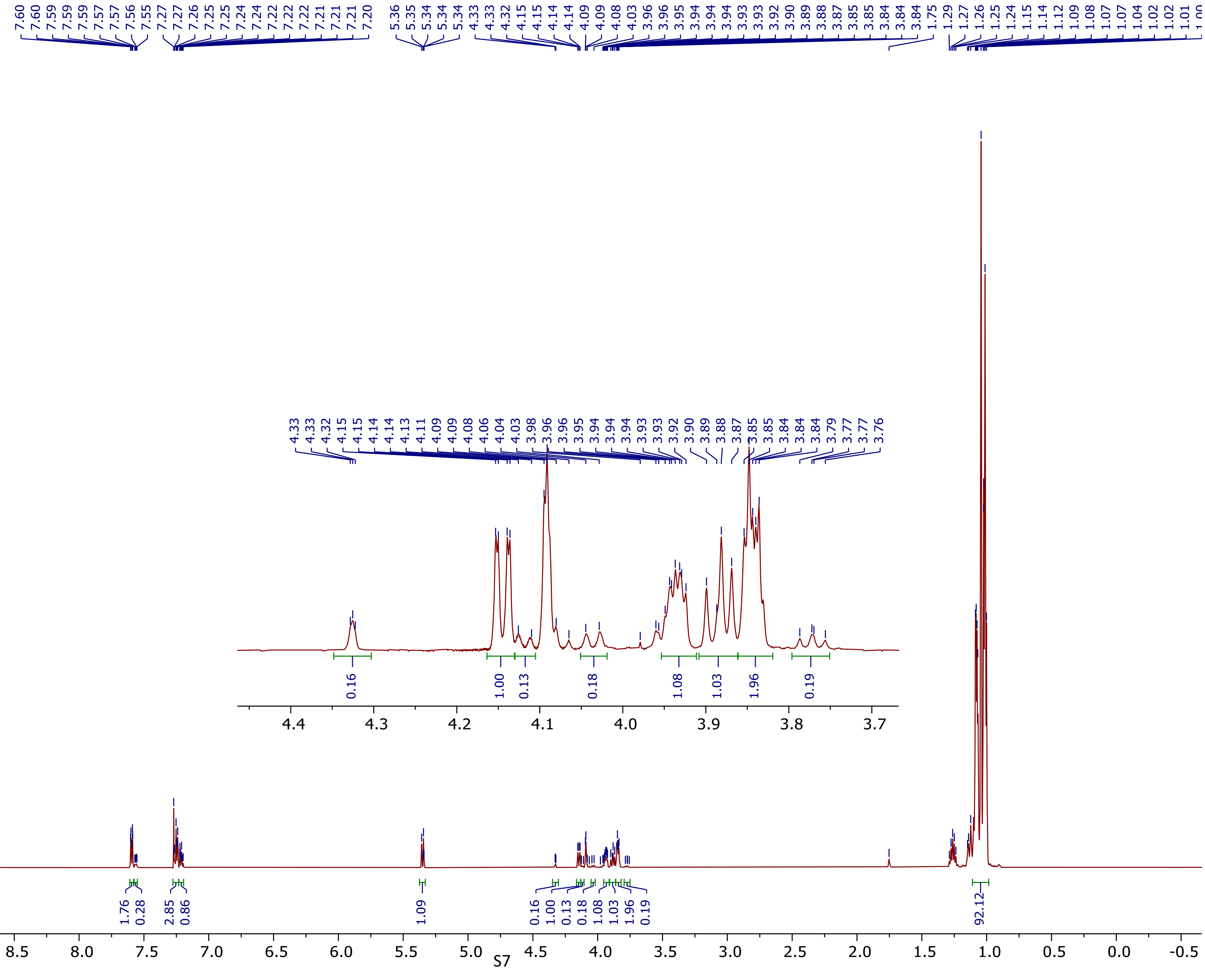

^{13}C NMR (151 MHz) spectrum of compound 2 in $CDCl_3$ (243K)

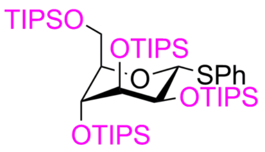

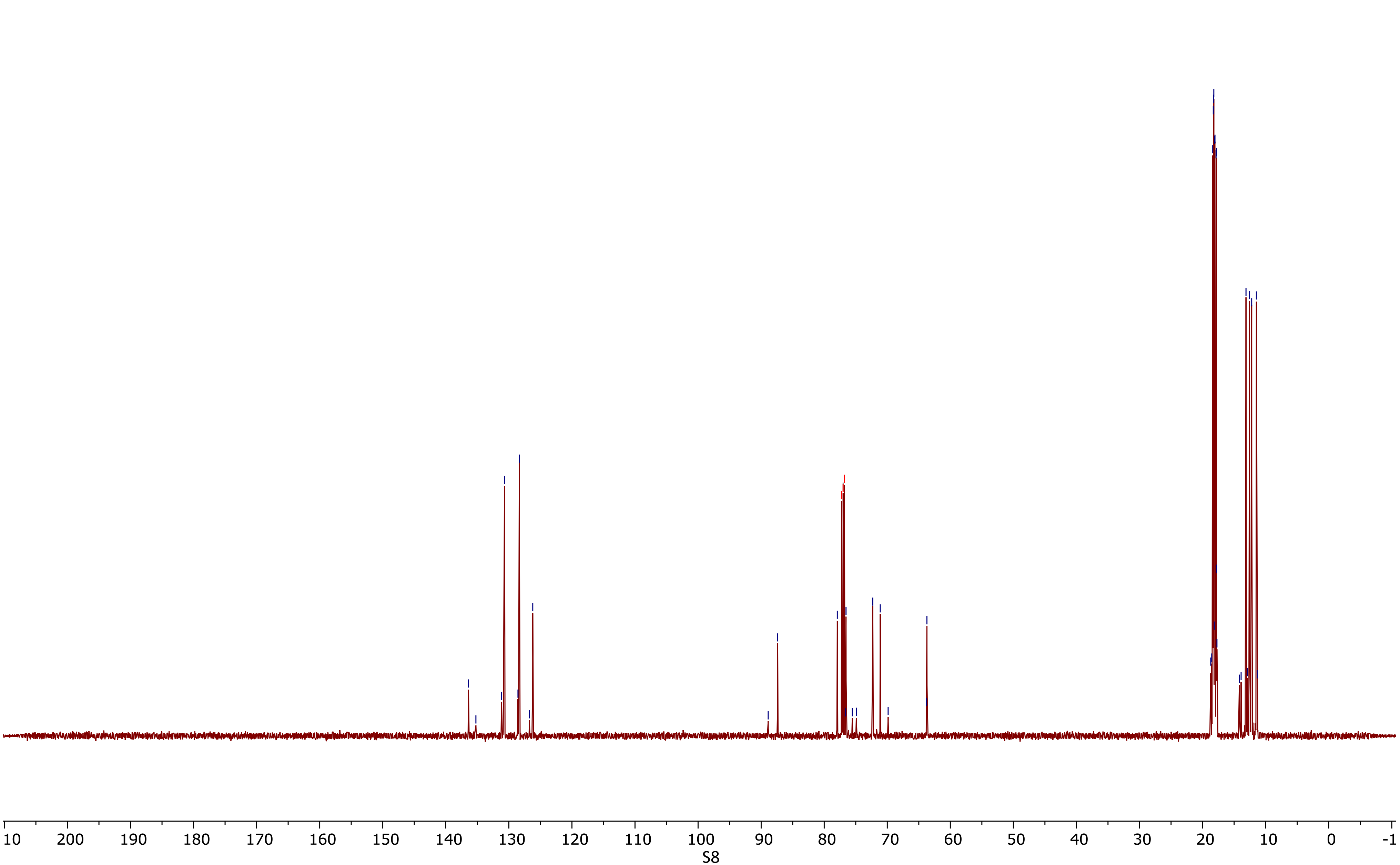

COSY (600 MHz) spectrum of compound 2 in $CDCl_3$ (243K)

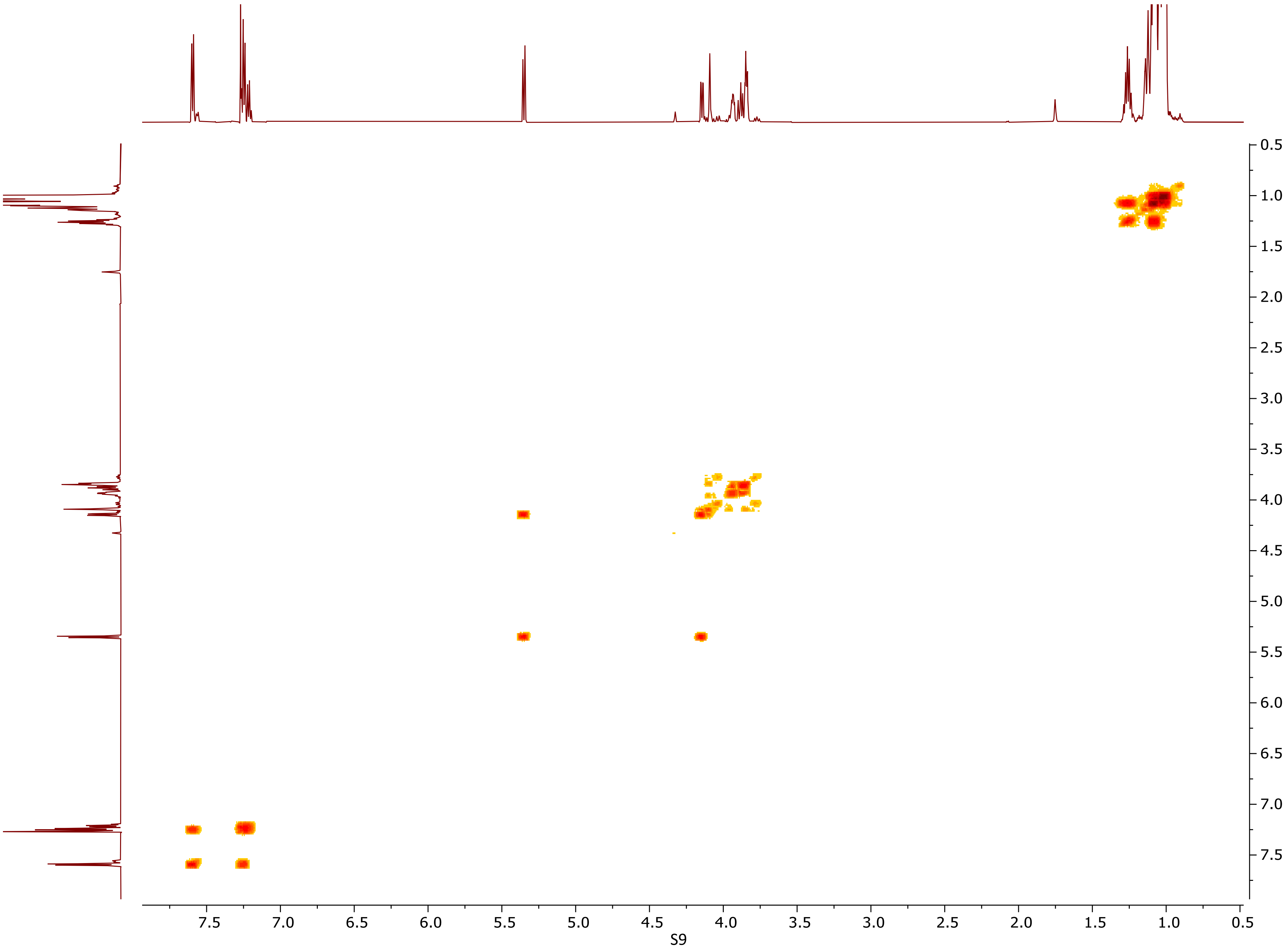

HSQC (600 MHz) spectrum of compound 2 in $CDCl_3$ (243K)

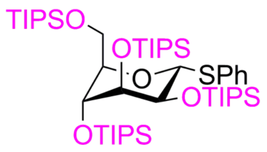

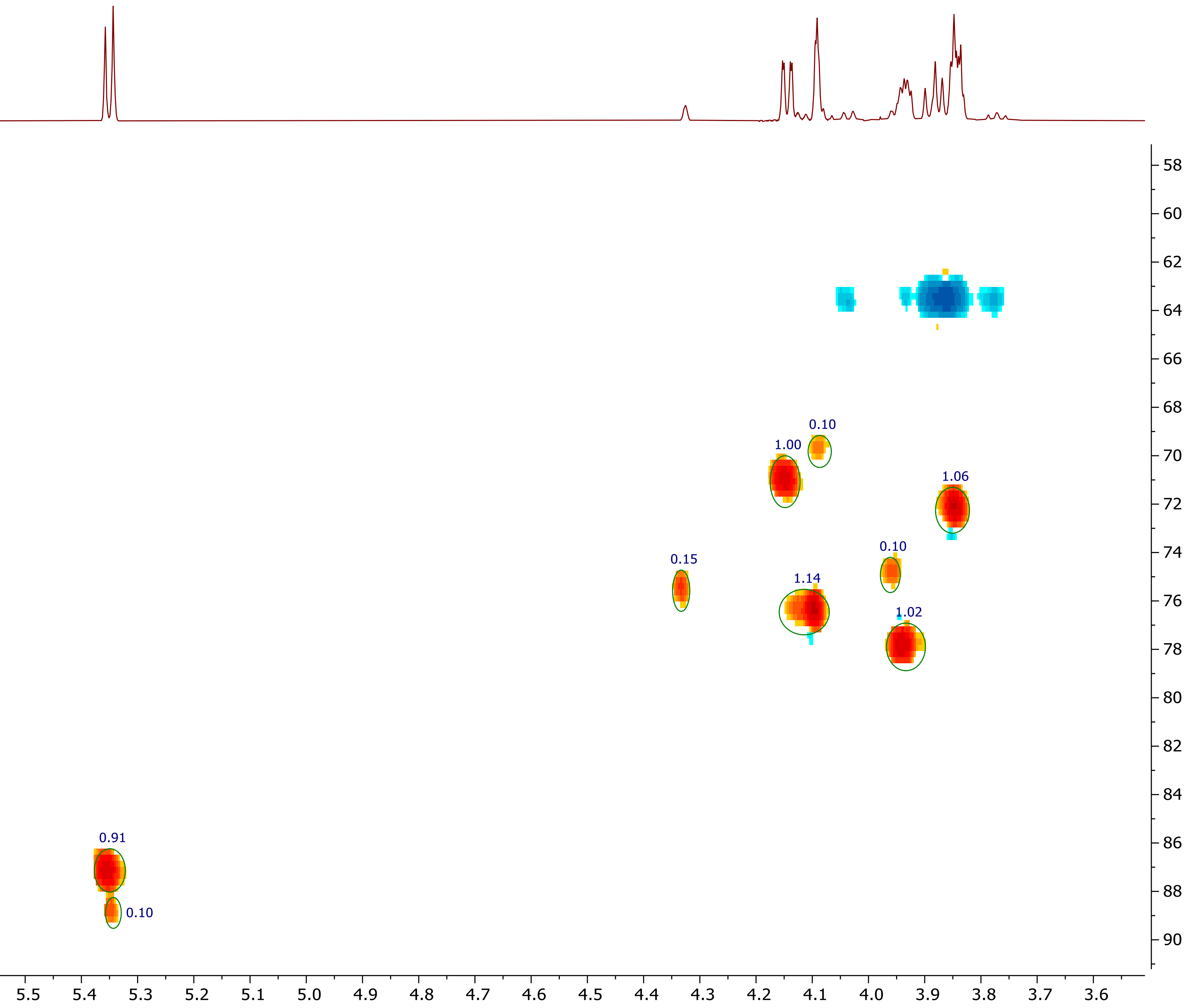

^1H NMR (300 MHz) spectrum of compound 3 in C_6D_6

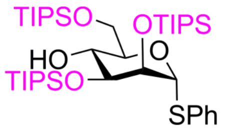

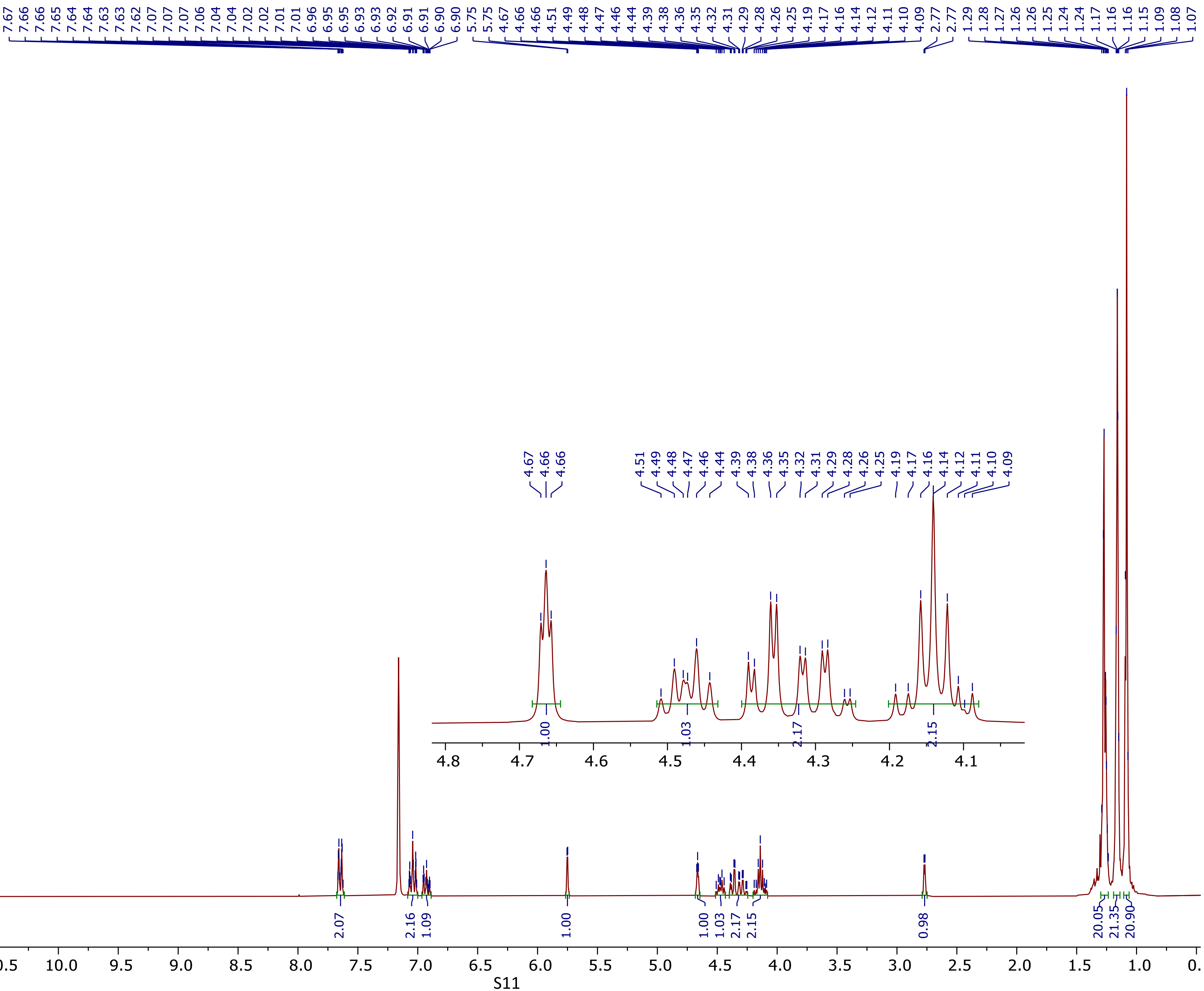

^{13}C NMR (75.5 MHz) spectrum of compound 3 in C_6D_6

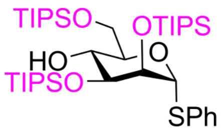

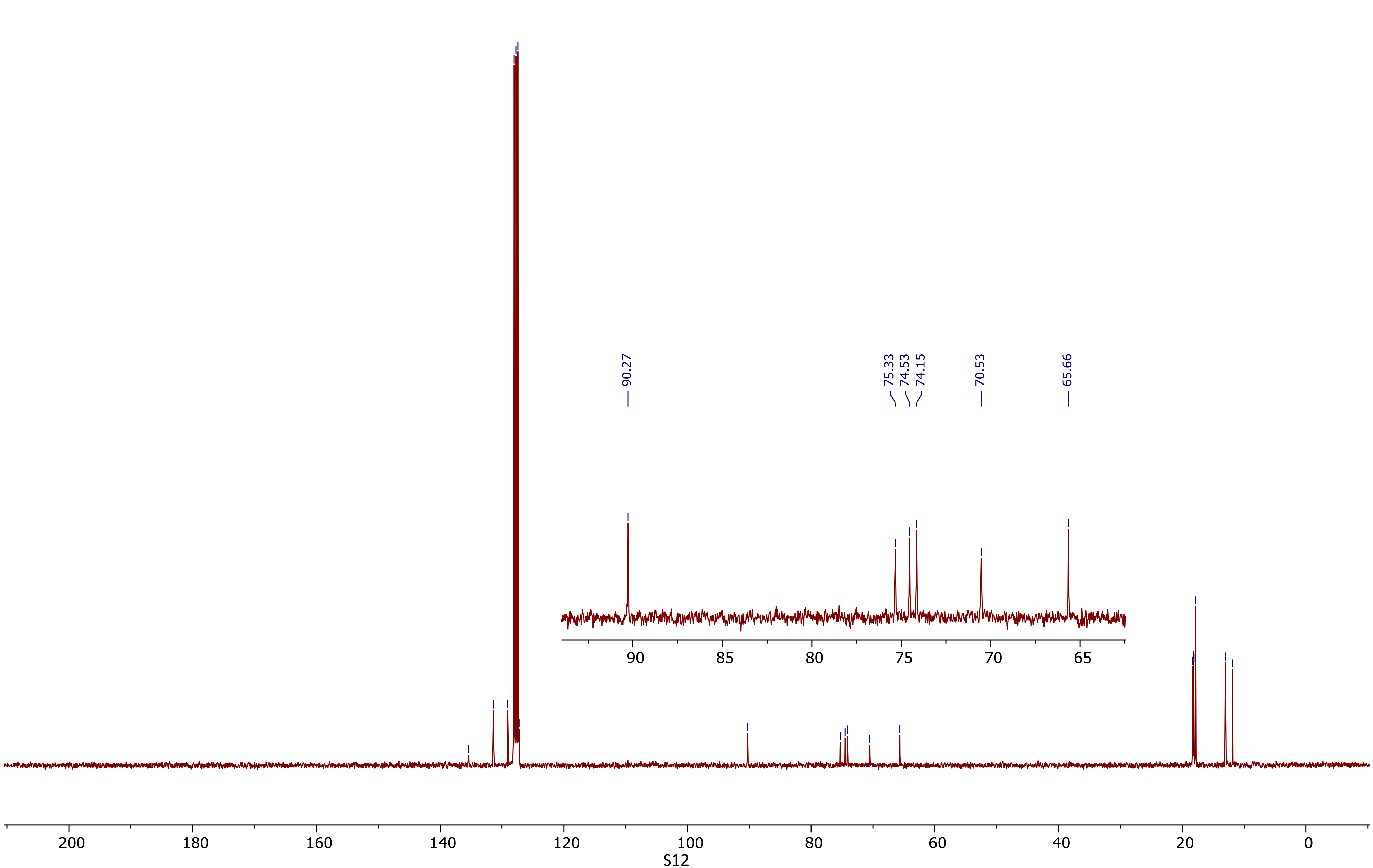

COSY (300 MHz) spectrum of compound 3 in C_6D_6

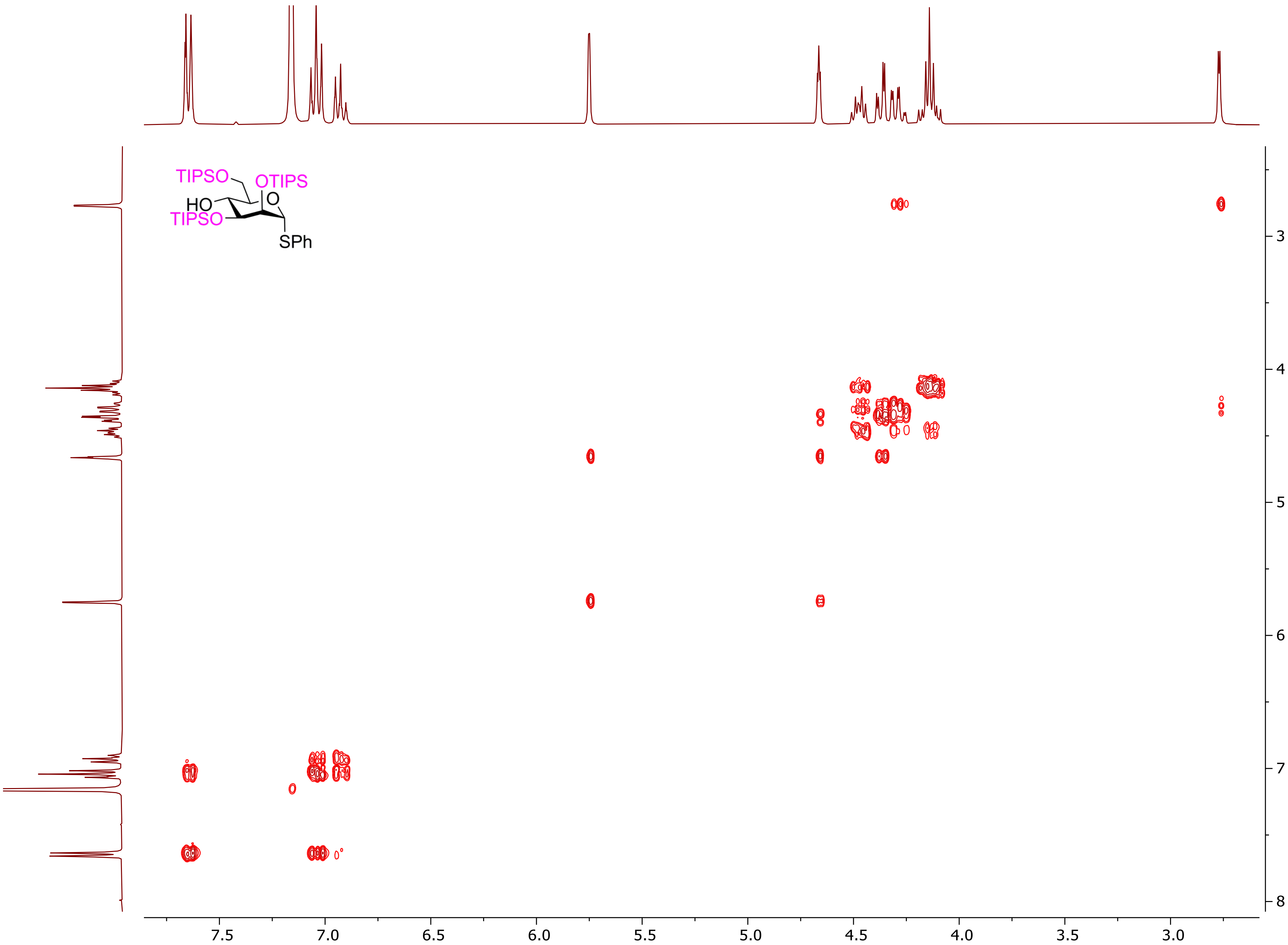

HSQC (300 MHz) spectrum of compound 3 in C_6D_6

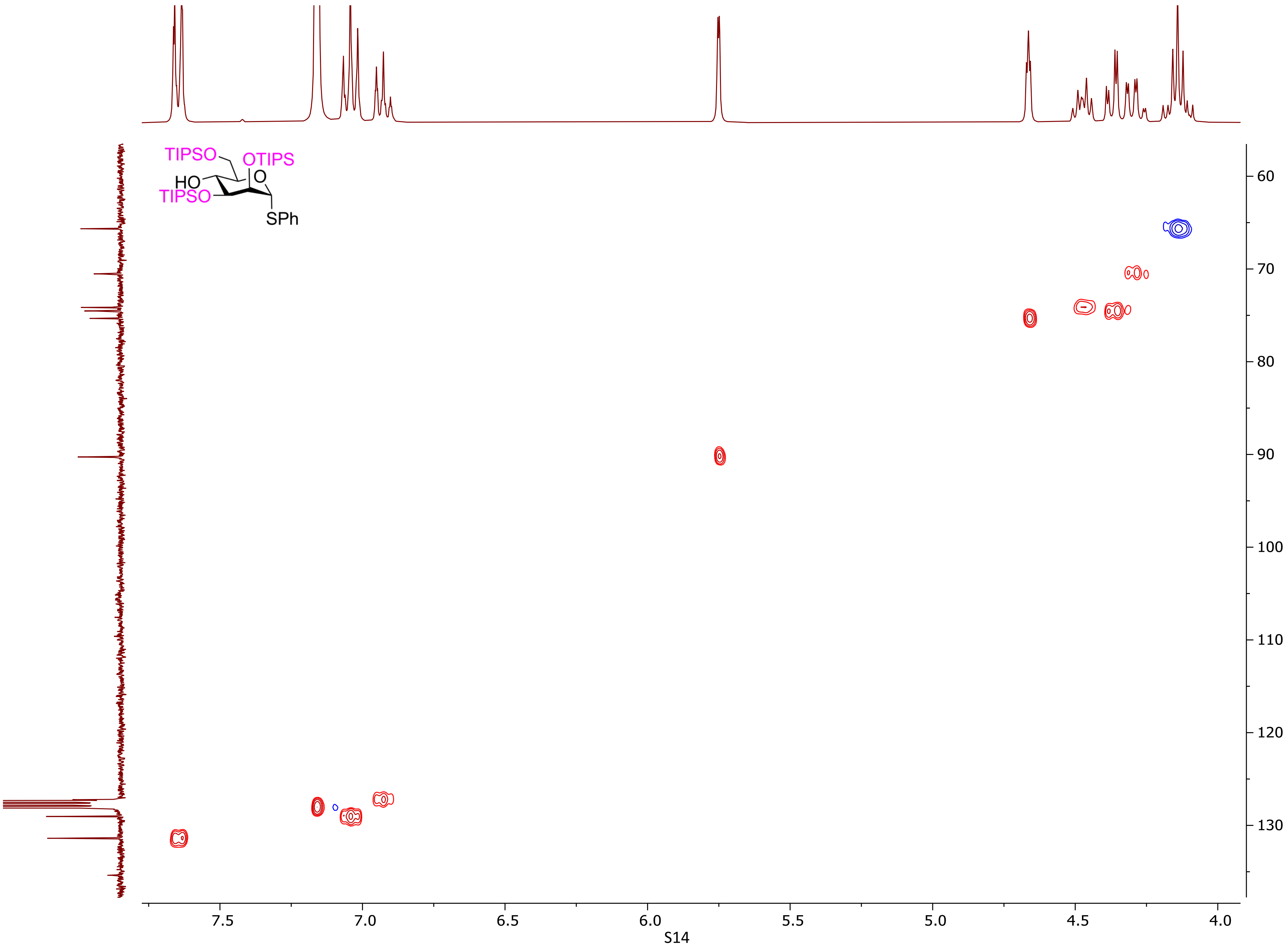

HMBC (300 MHz) spectrum of compound 3 in C_6D_6

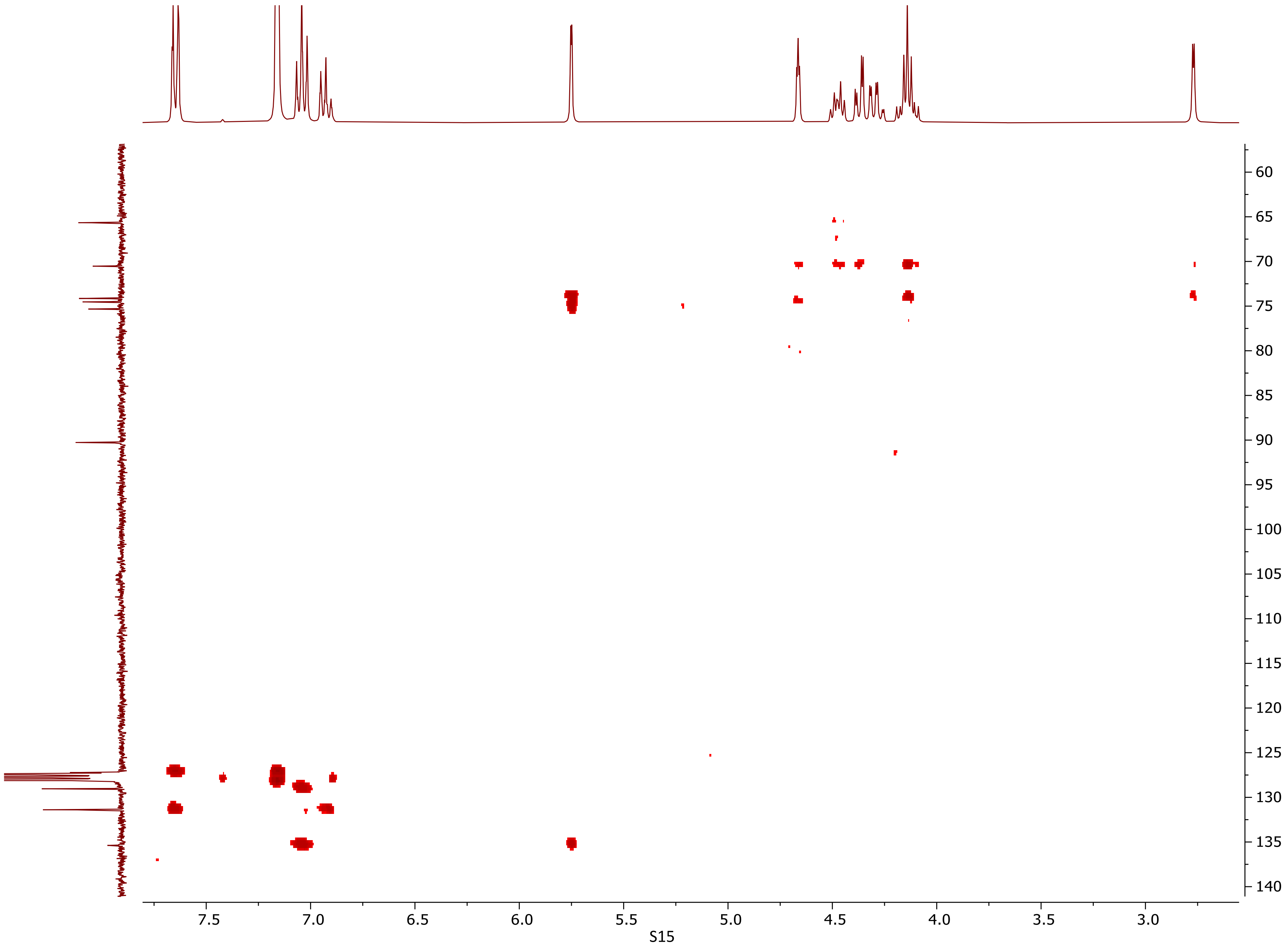

^{1}H–^{29}Si HMBC (300 MHz) spectrum of compound 3 in C_6D_6

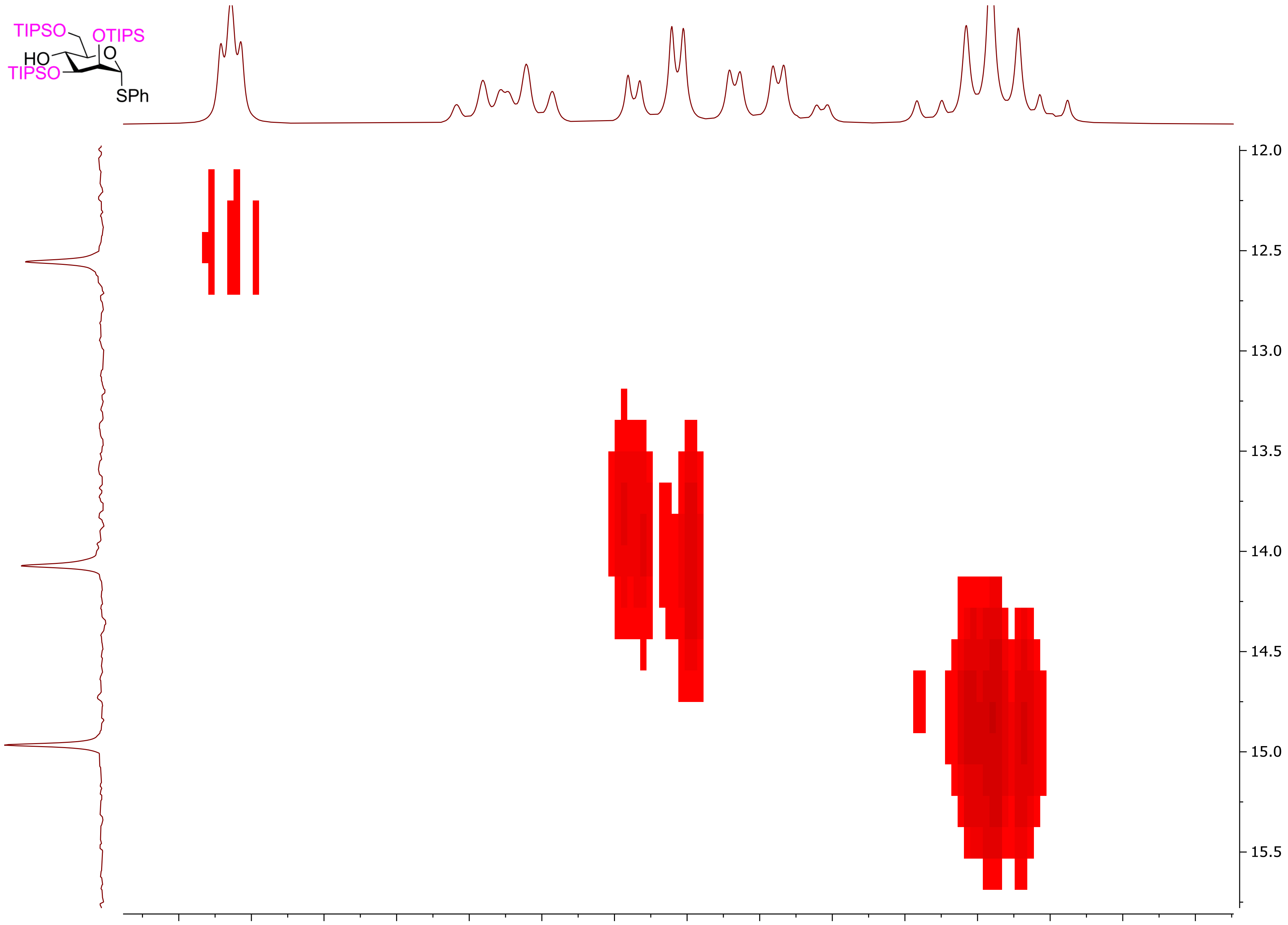

^{29}Si INEPT NMR (59 MHz) spectrum of compound 3 in C_6D_6

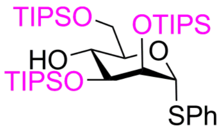

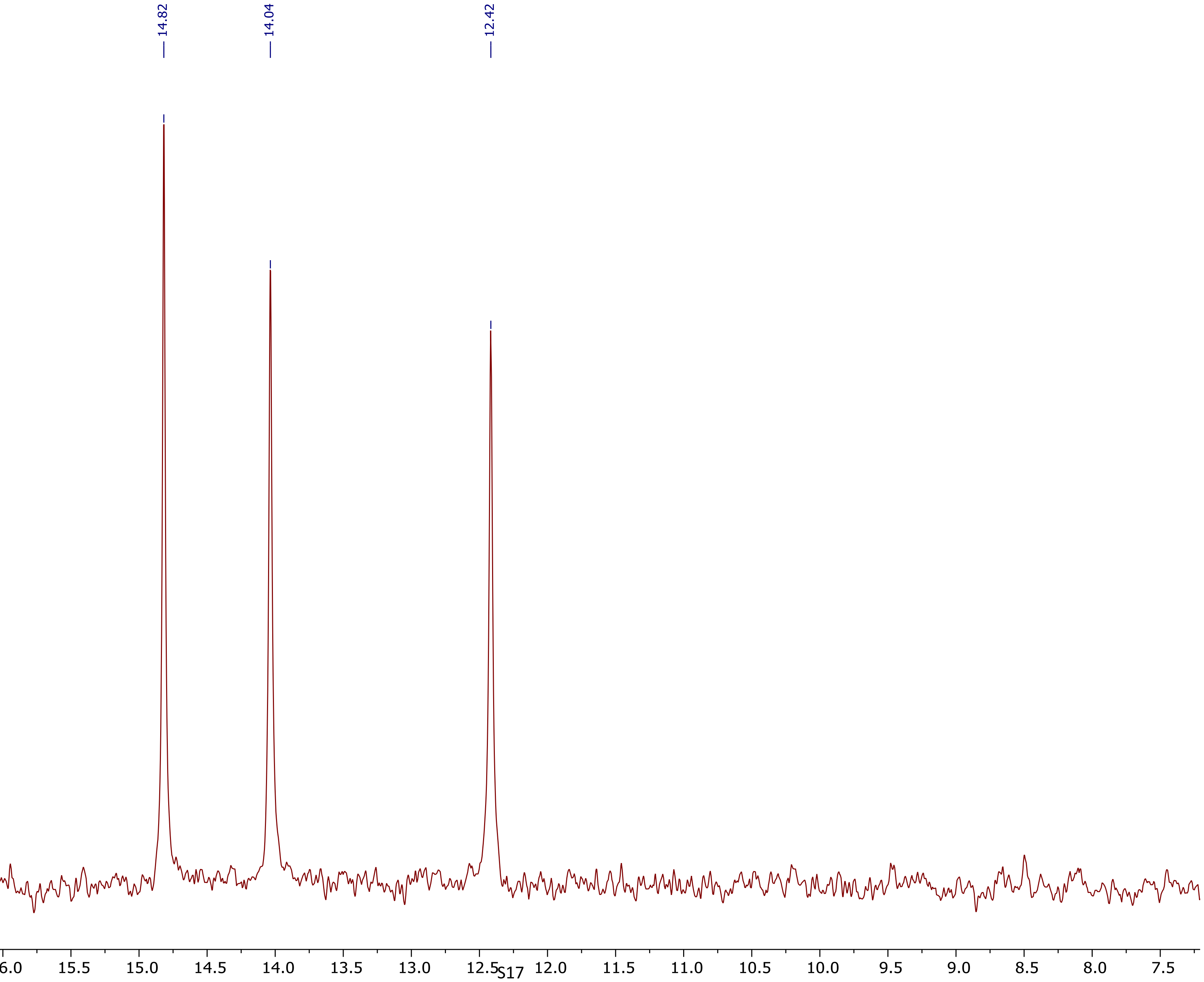

^{1}H NMR (300 MHz) spectrum of compound 4 in C_6D_6

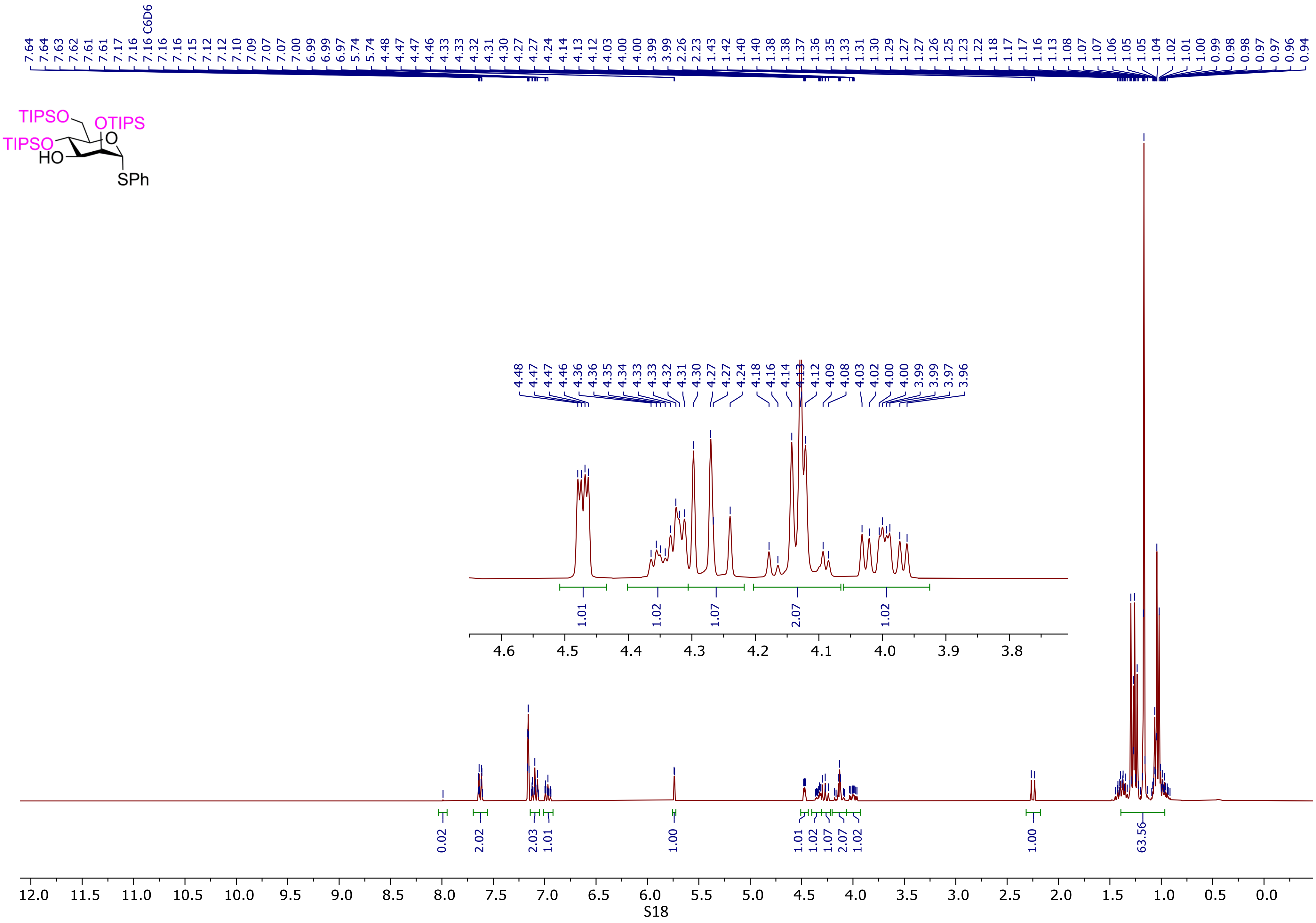

^{13}C NMR (75.5 MHz) spectrum of compound 4 in C_6D_6

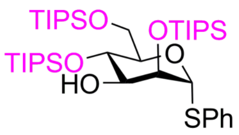

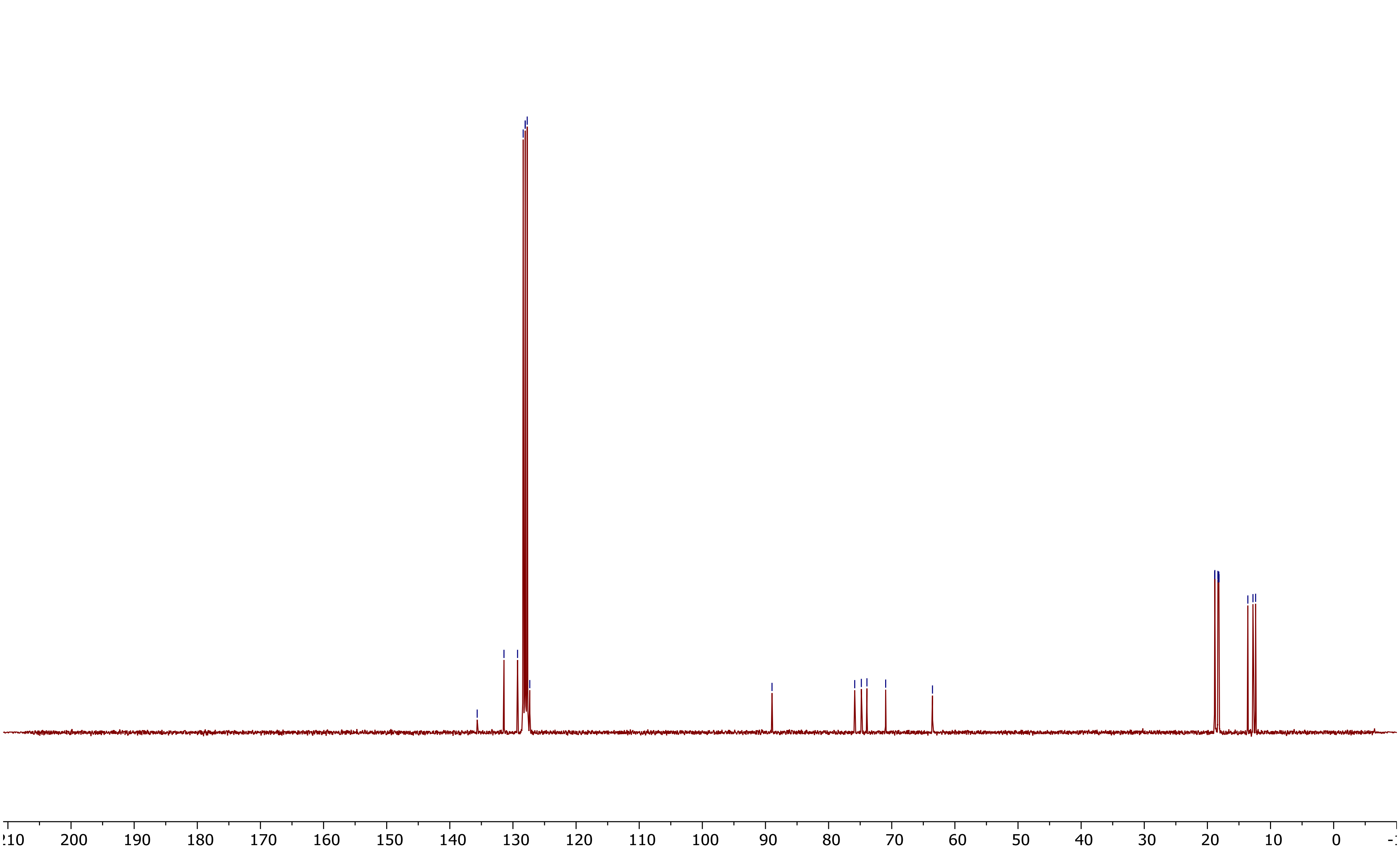

COSY (300 MHz) spectrum of compound 4 in C_6D_6

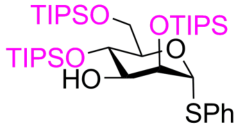

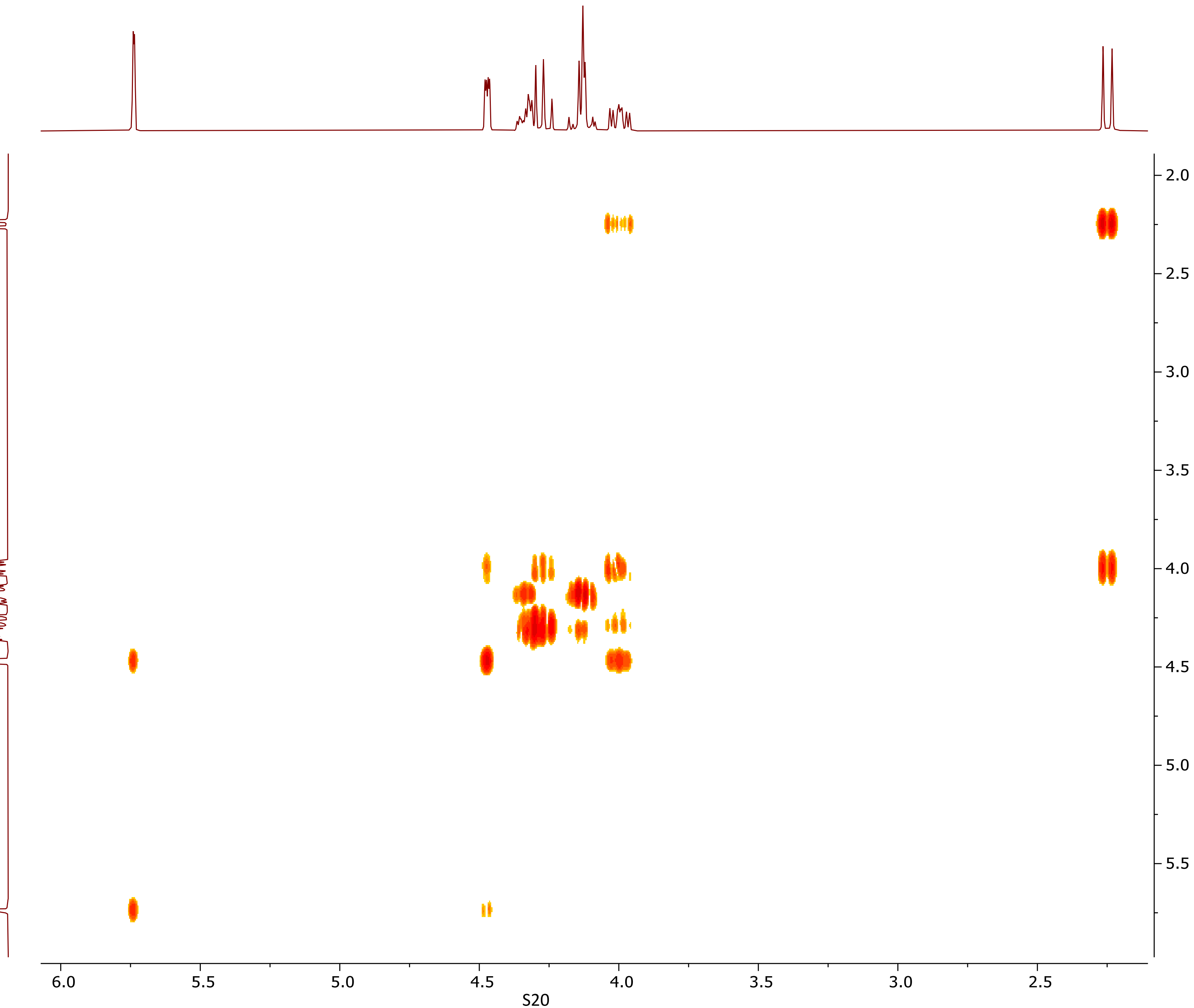

HSQC (300 MHz) spectrum of compound 4 in C_6D_6

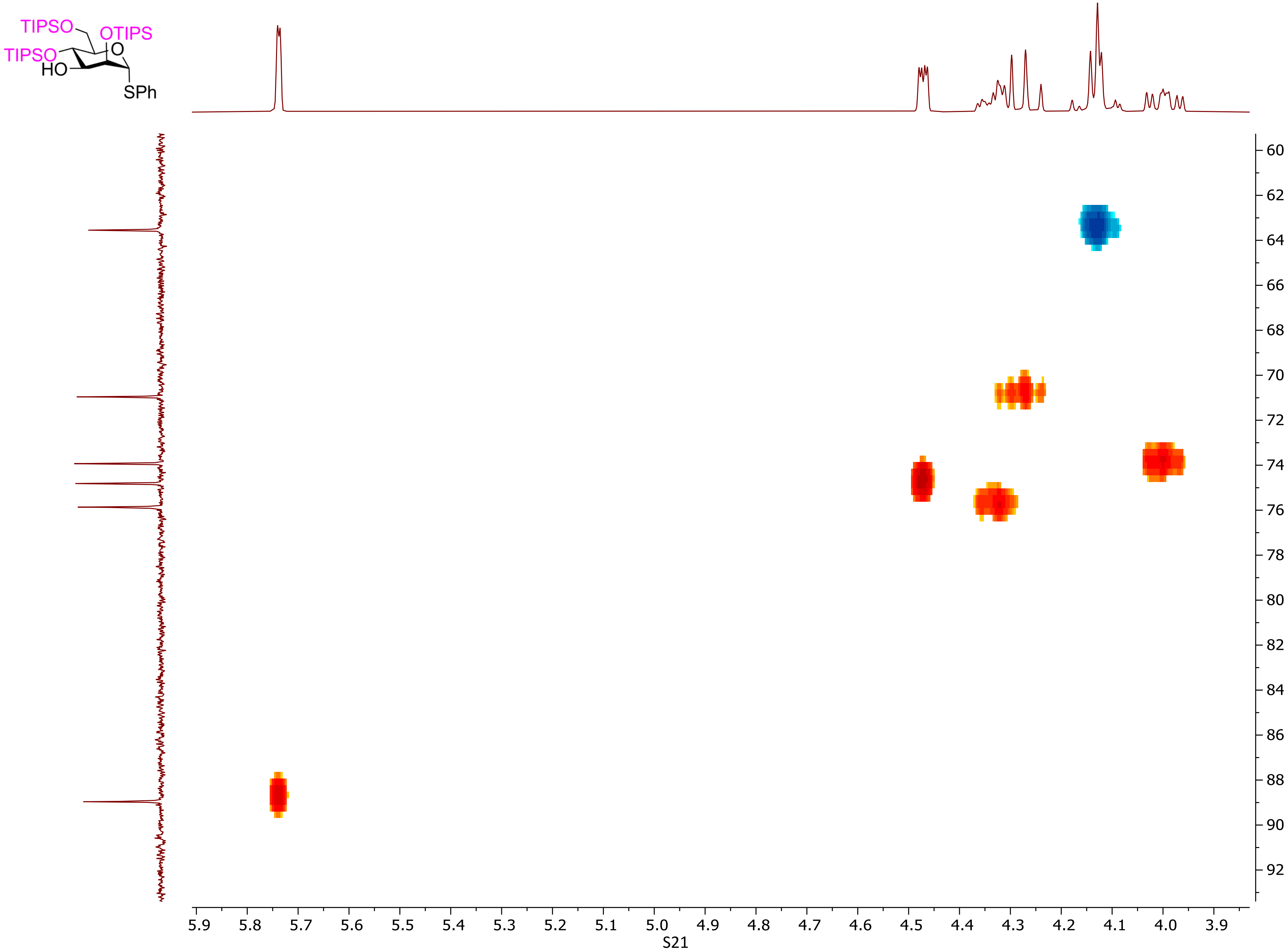

HMBC(300 MHz) spectrum of compound 4 in C_6D_6

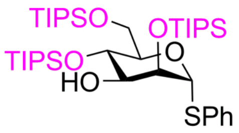

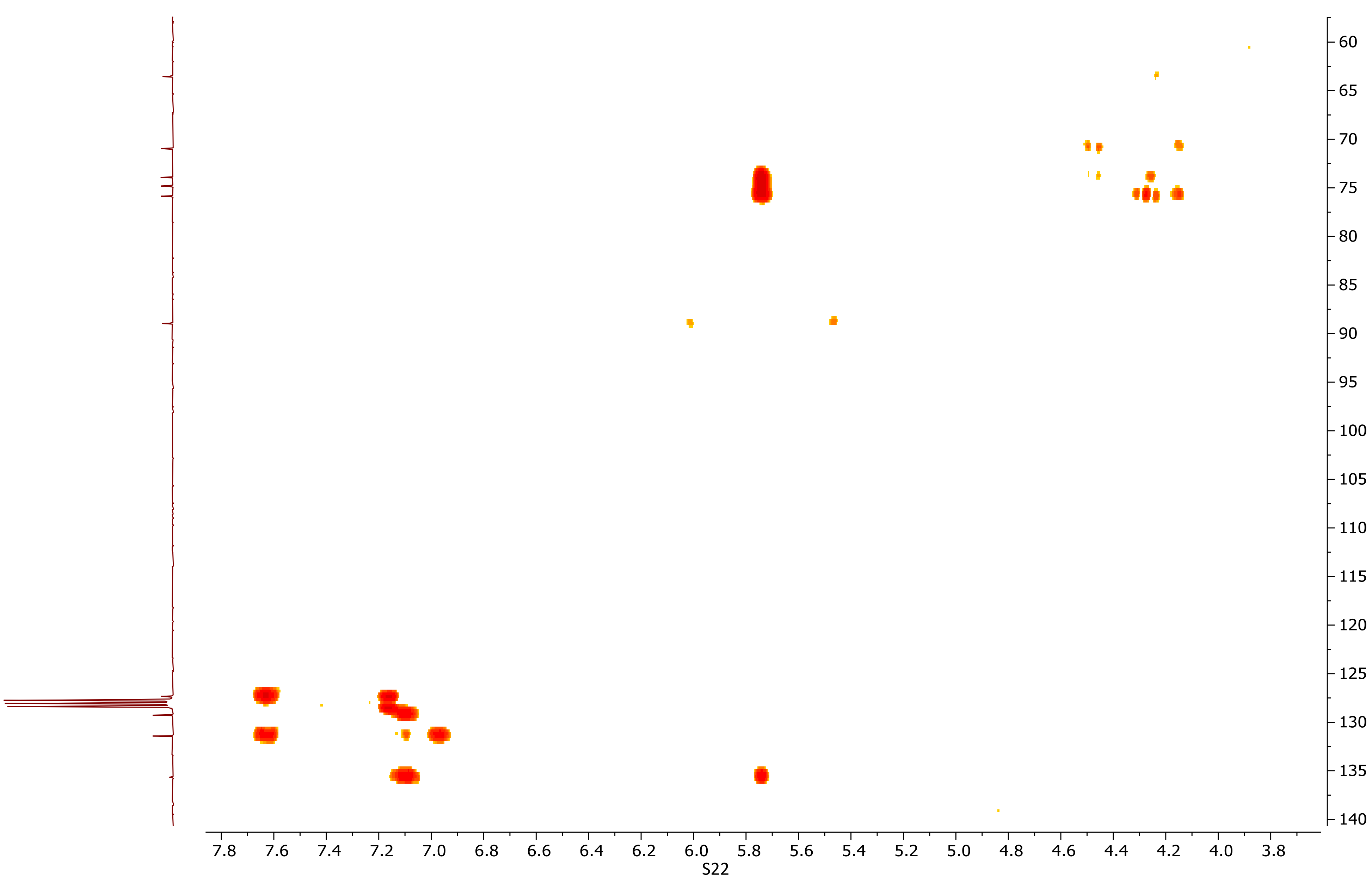

^{1}H–^{29}Si HMBC (300 MHz) spectrum of compound 4 in C_6D_6

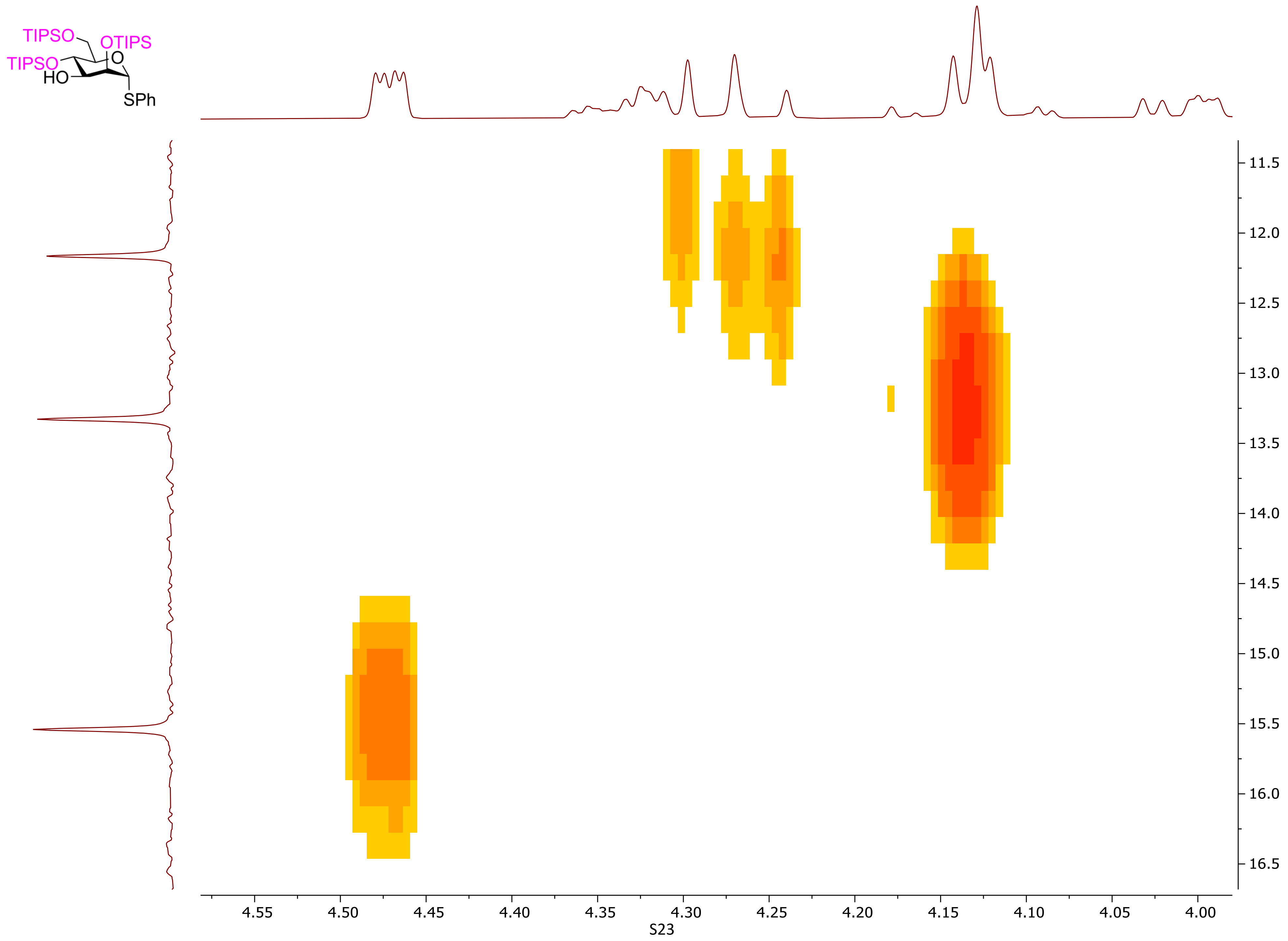

^{29}Si INEPT NMR (59 MHz) spectrum of compound 4 in C_6D_6

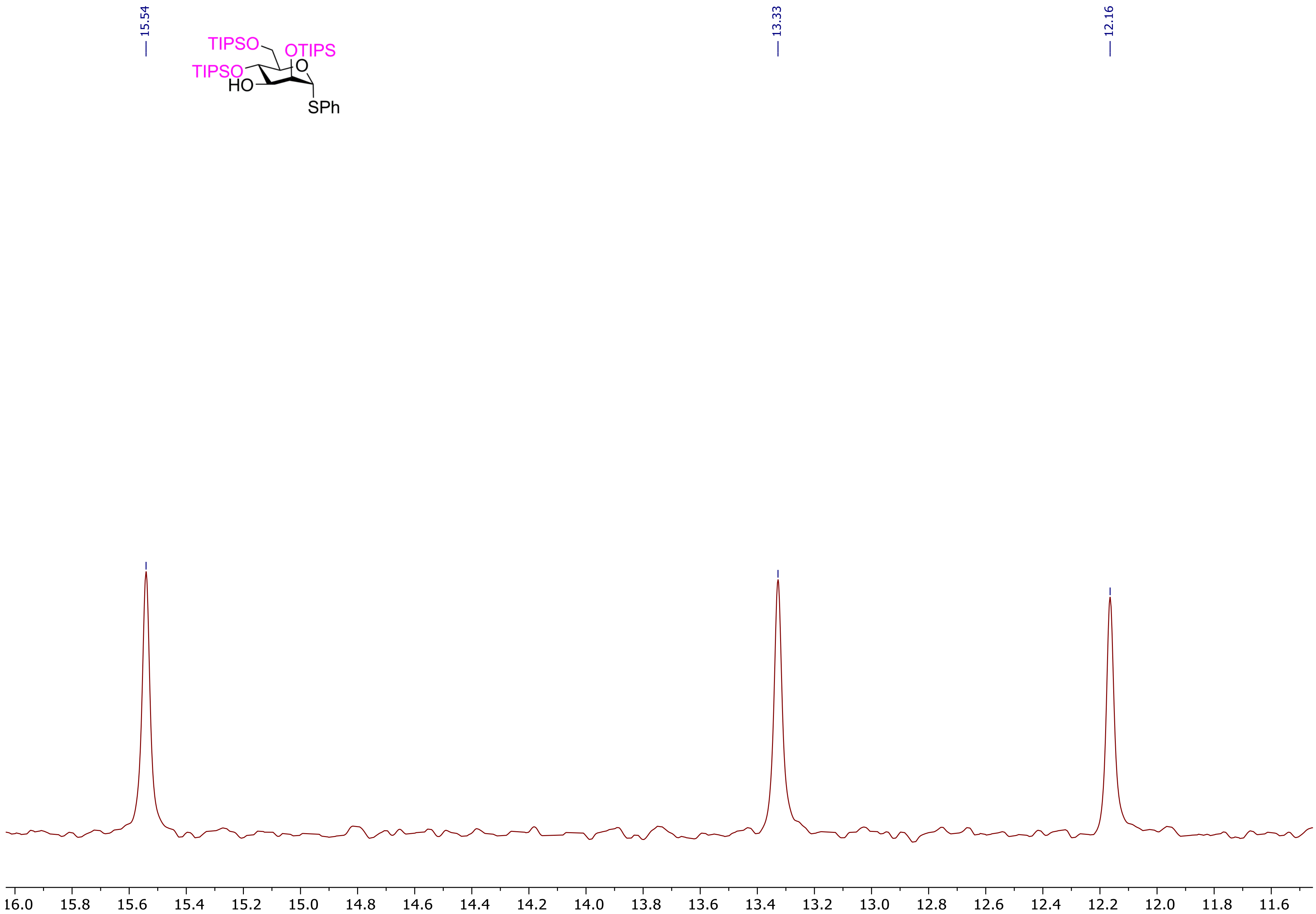

^{1}H NMR (300 MHz) spectrum of compound 5 in C_6D_6

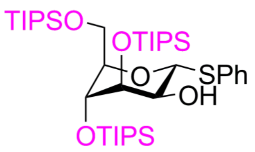

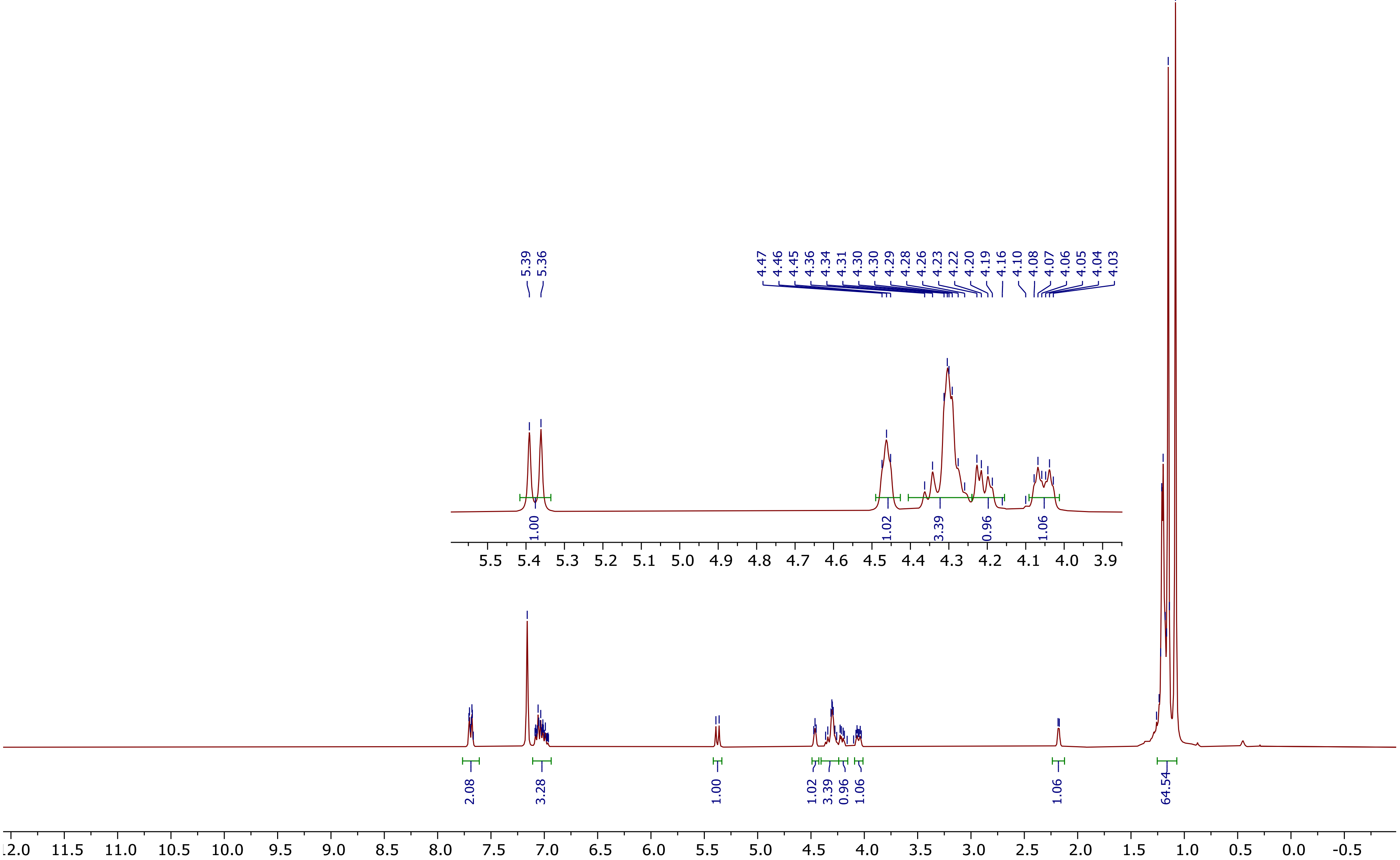

^{13}C NMR (75.5 MHz) spectrum of compound 5 in C_6D_6

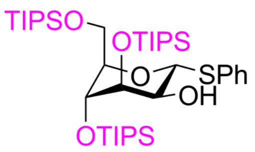

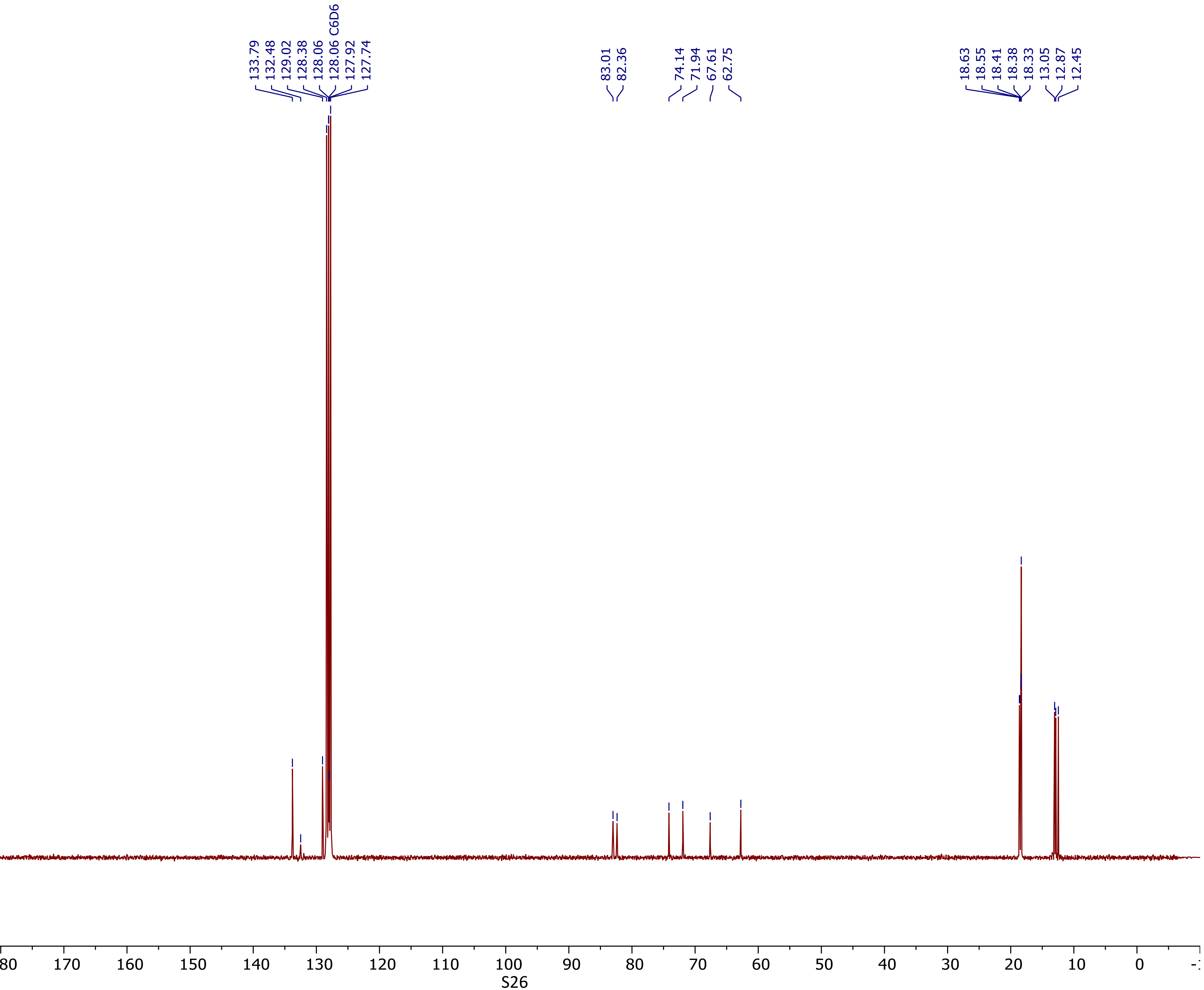

COSY (300 MHz) spectrum of compound 5 in C_6D_6

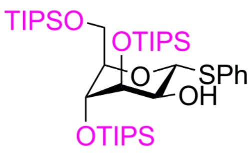

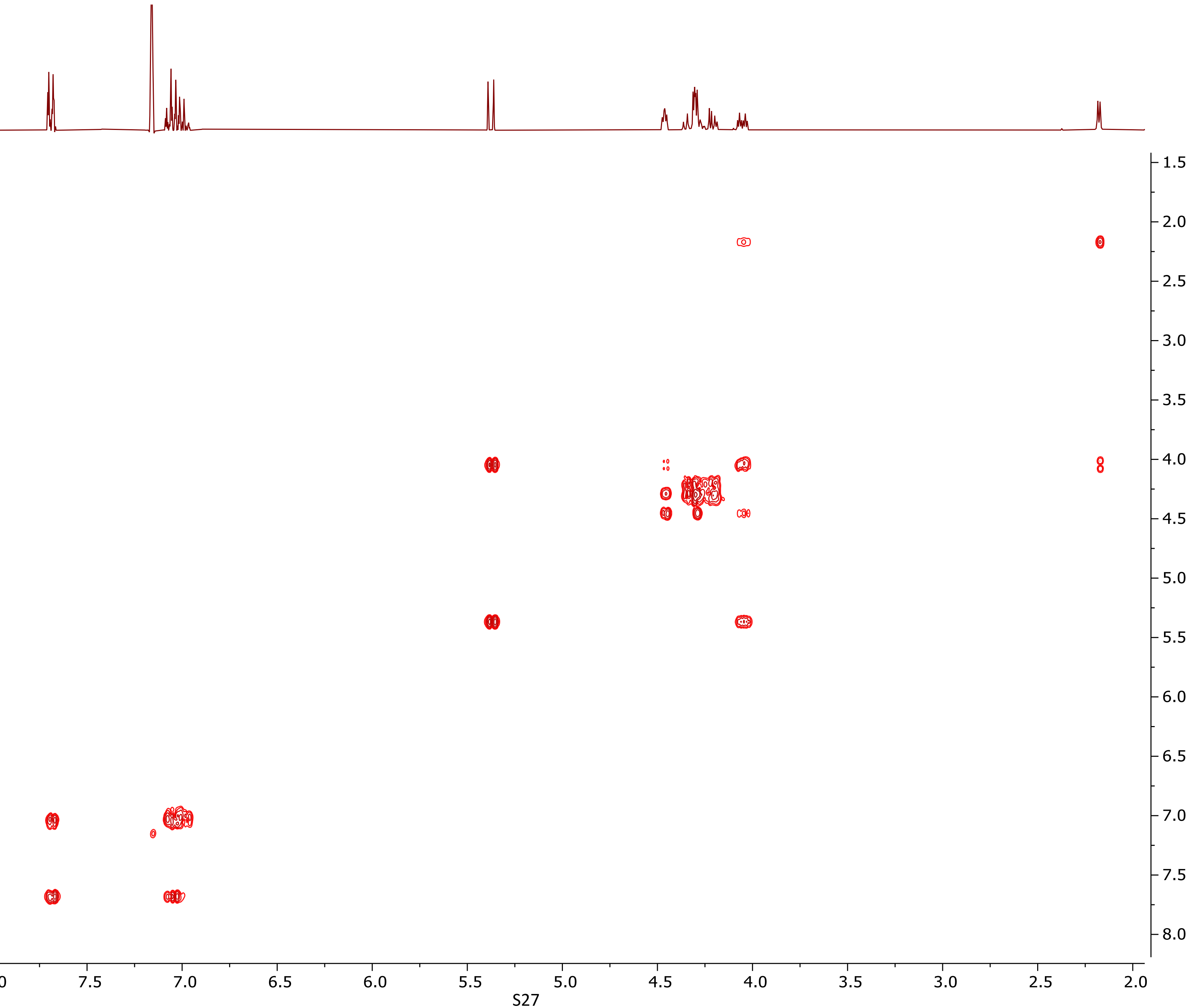

HSQC (300 MHz) spectrum of compound 5 in C_6D_6

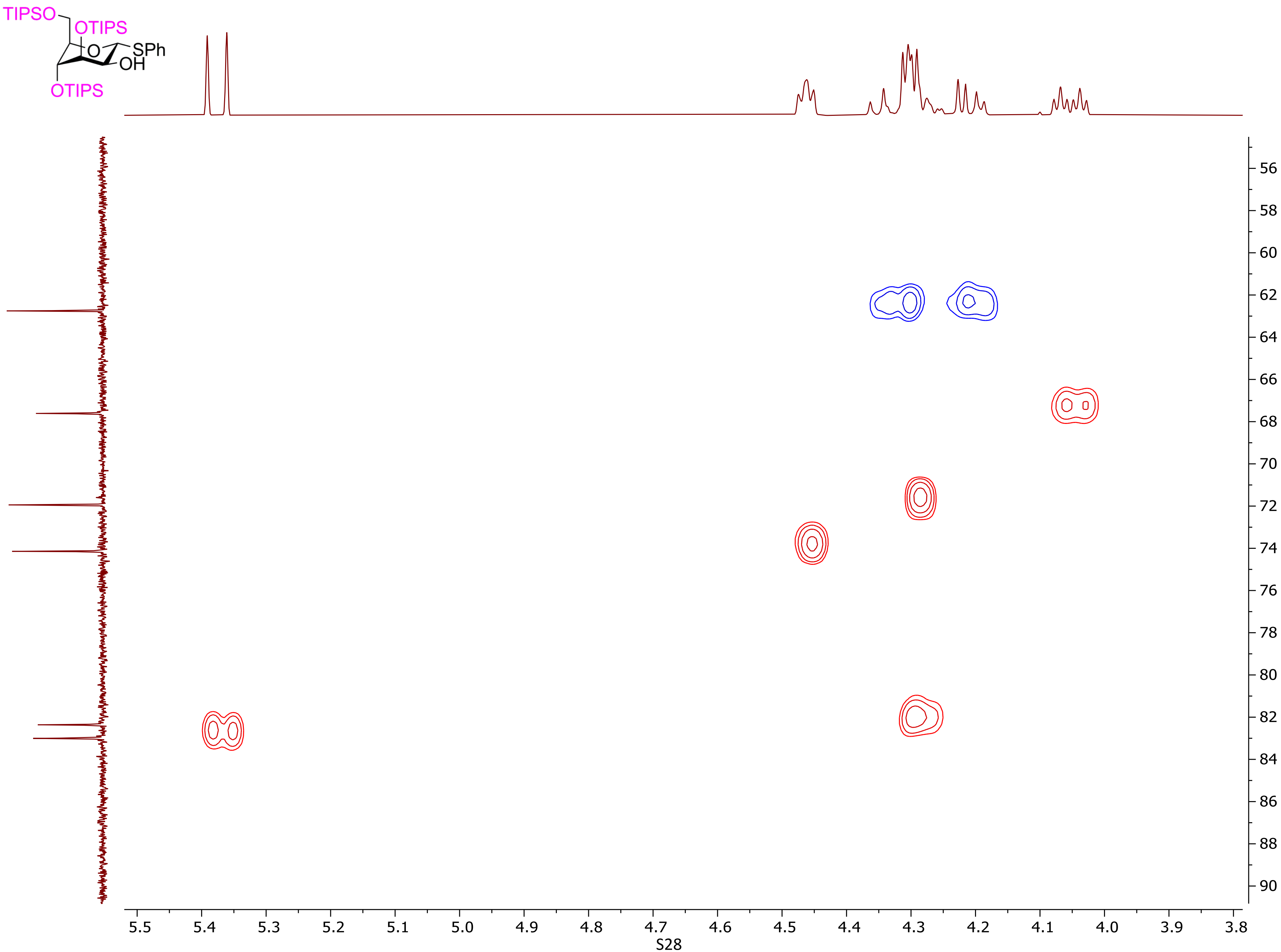

HMBC (300 MHz) spectrum of compound 5 in C_6D_6

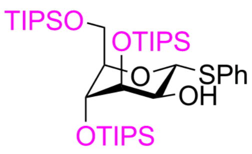

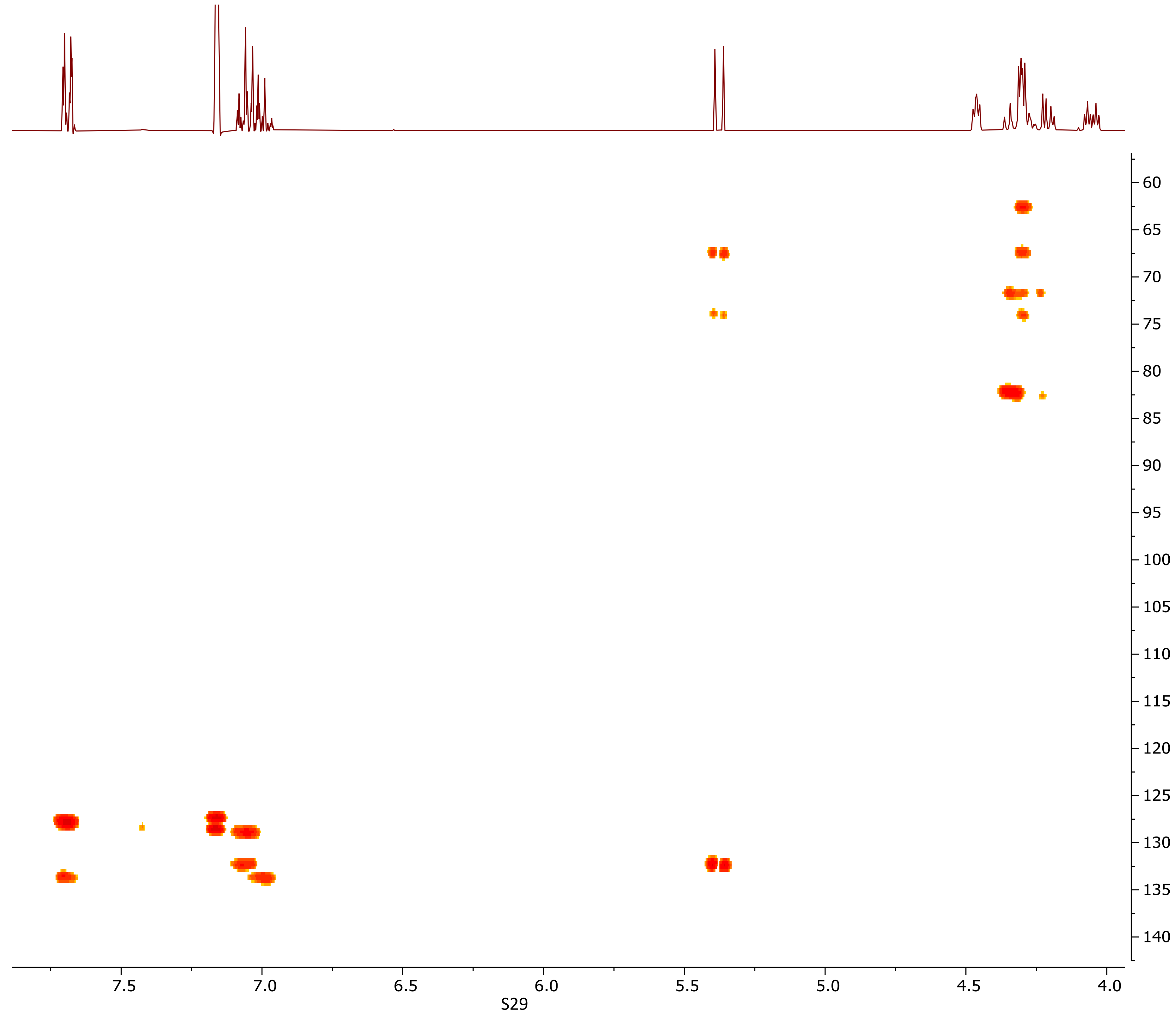

$^{1}H–^{29}Si$ HMBC (300 MHz) spectrum of compound 5 in C_6D_6

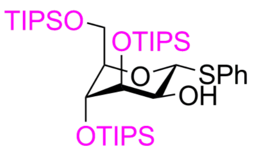

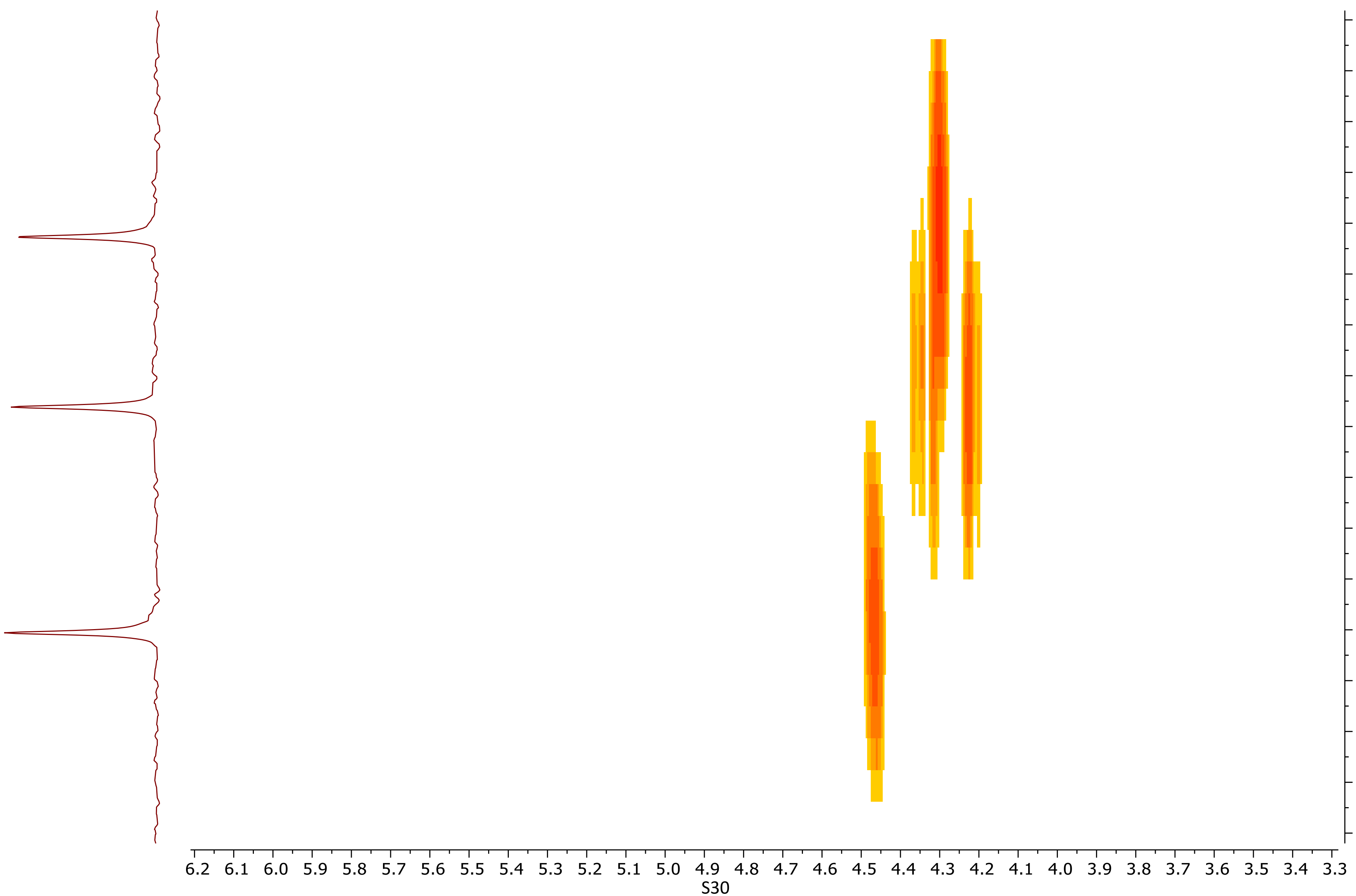

^{29}Si INEPT NMR (59 MHz) spectrum of compound 5 in C_6D_6

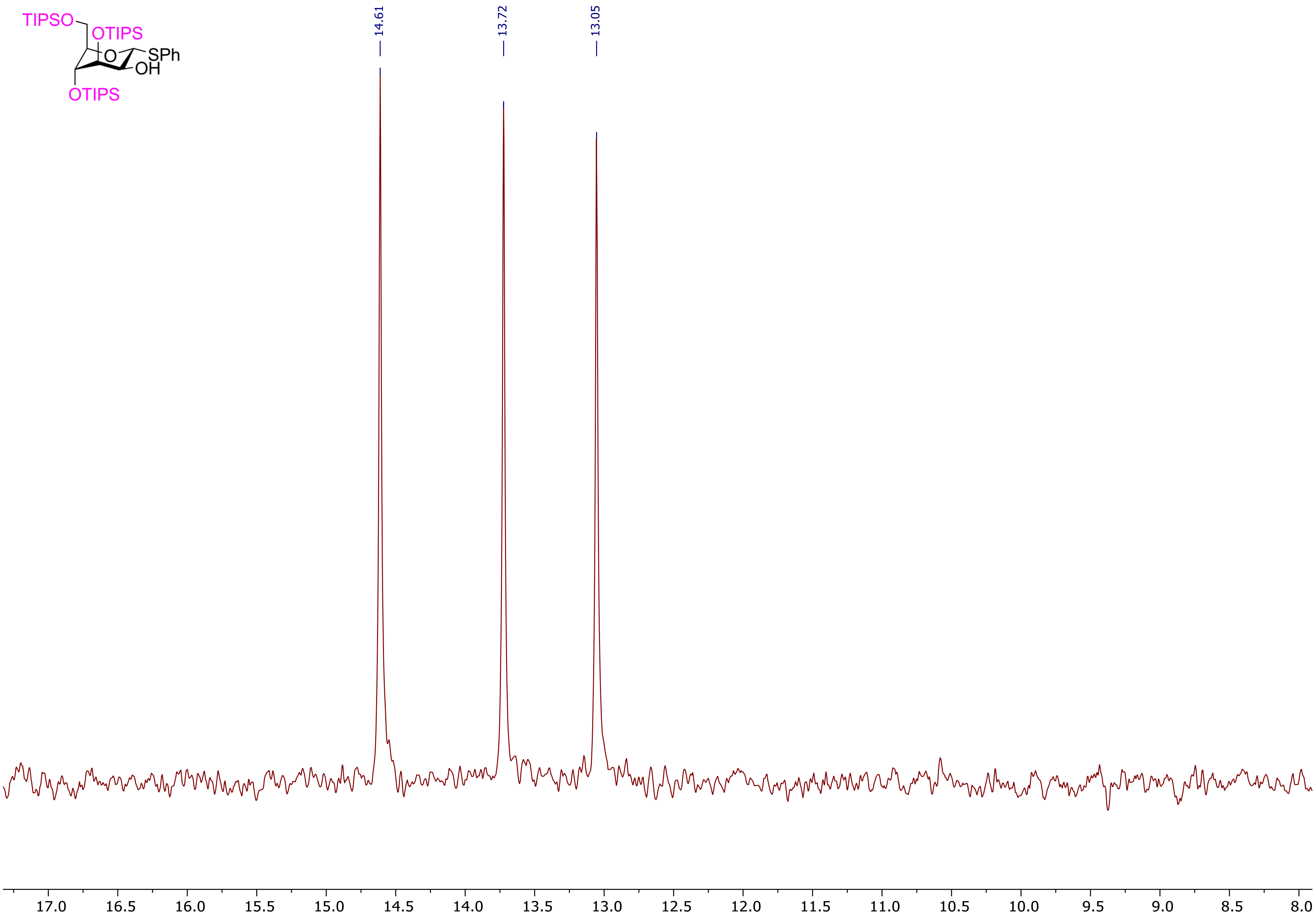

^{1}H NMR (300 MHz) spectrum of compound 6 in $CDCl_3$

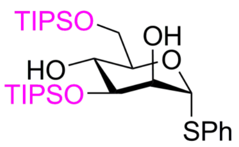

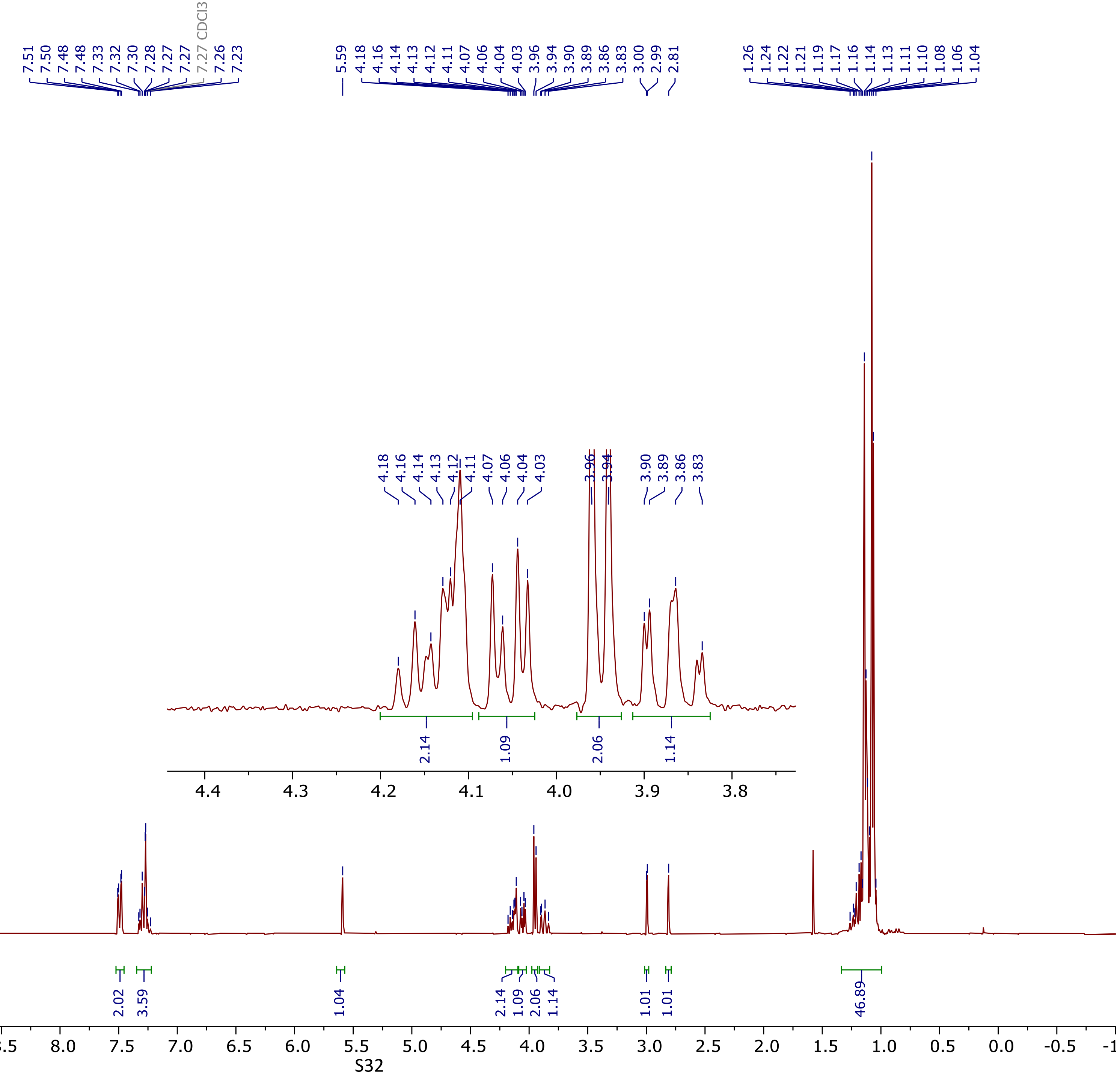

^{13}C NMR (75.5 MHz) spectrum of compound 6 in $CDCl_3$

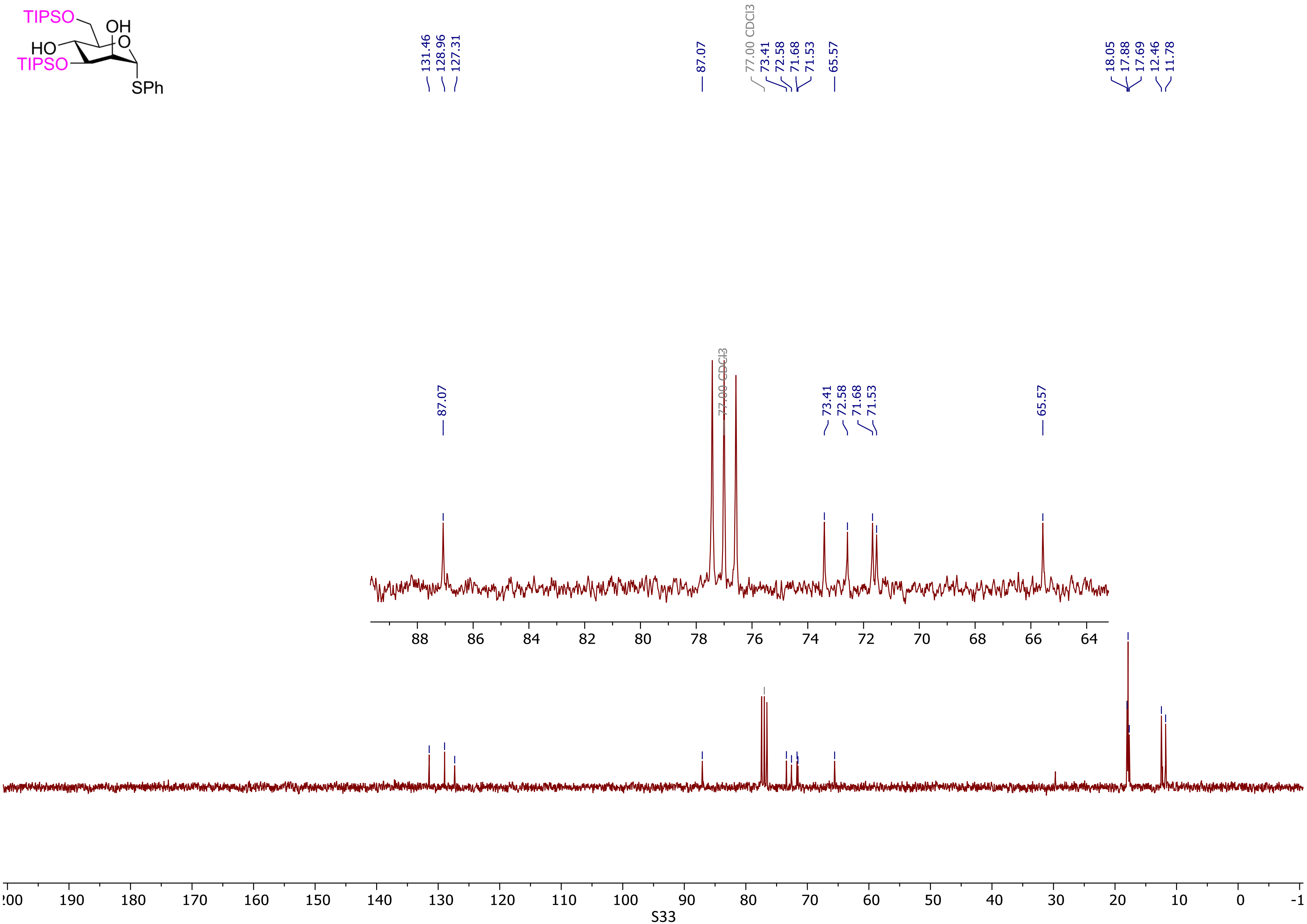

COSY (300 MHz) spectrum of compound 6 in $CDCl_3$

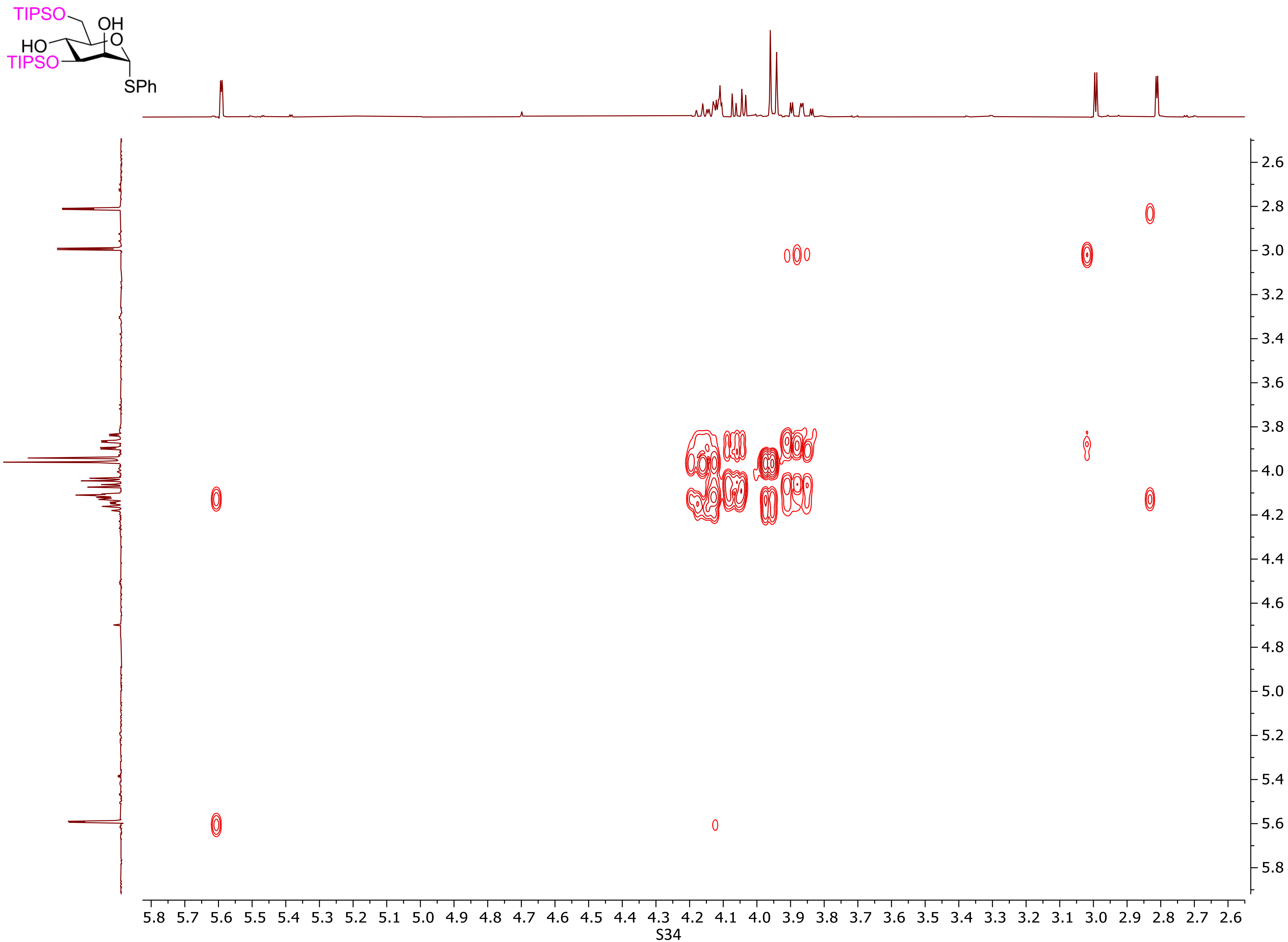

HSQC (300 MHz) spectrum of compound 6 in $CDCl_3$

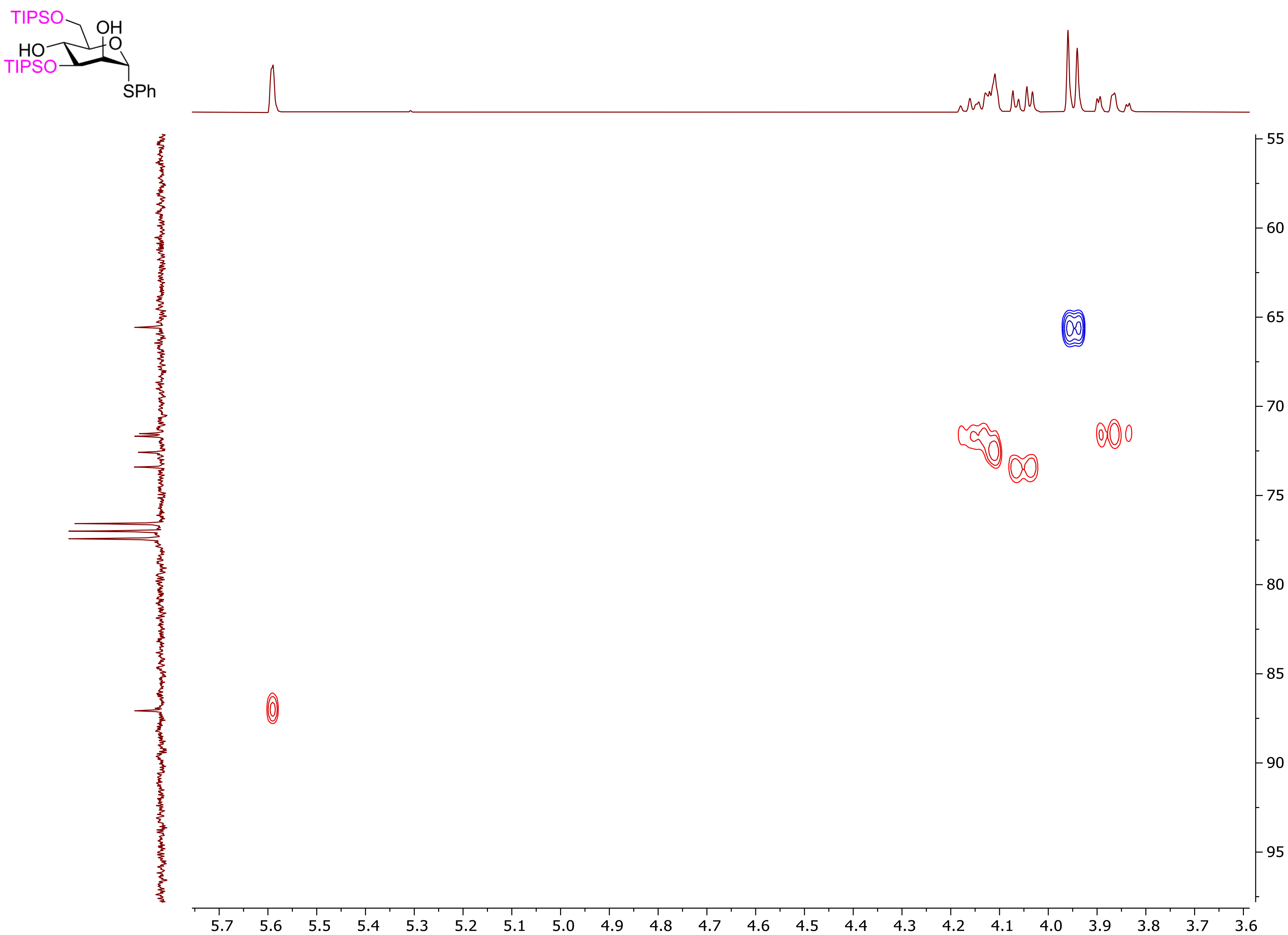

HMBC (300 MHz) spectrum of compound 6 in $CDCl_3$

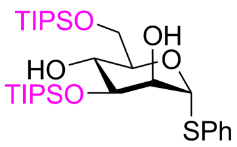

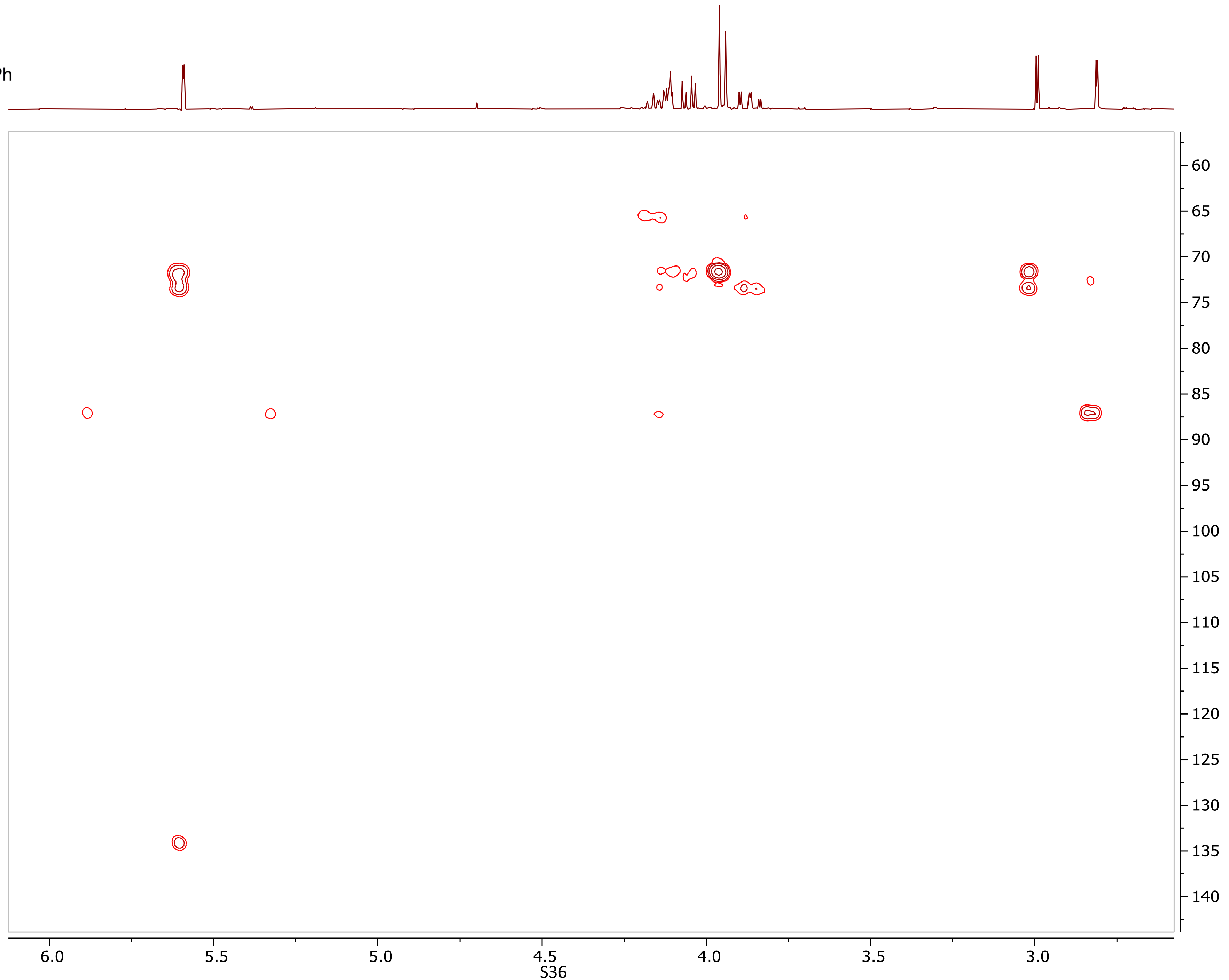

^{1}H NMR (600 MHz) spectrum of compound 7 in $CDCl_3$ (240K)

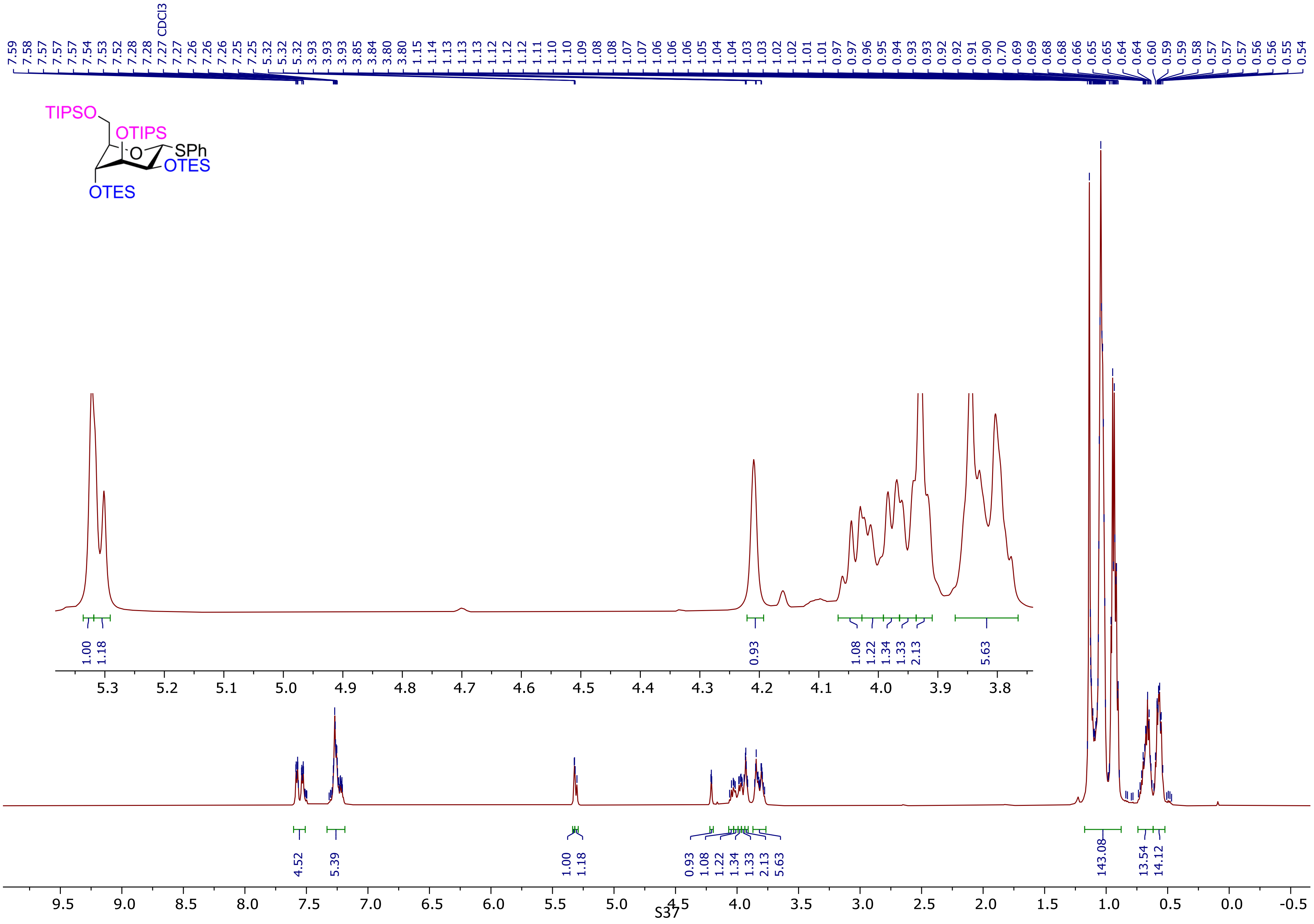

^{13}C NMR (151 MHz) spectrum of compound 7 in $CDCl_3$ (240K)

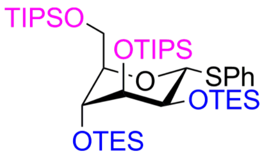

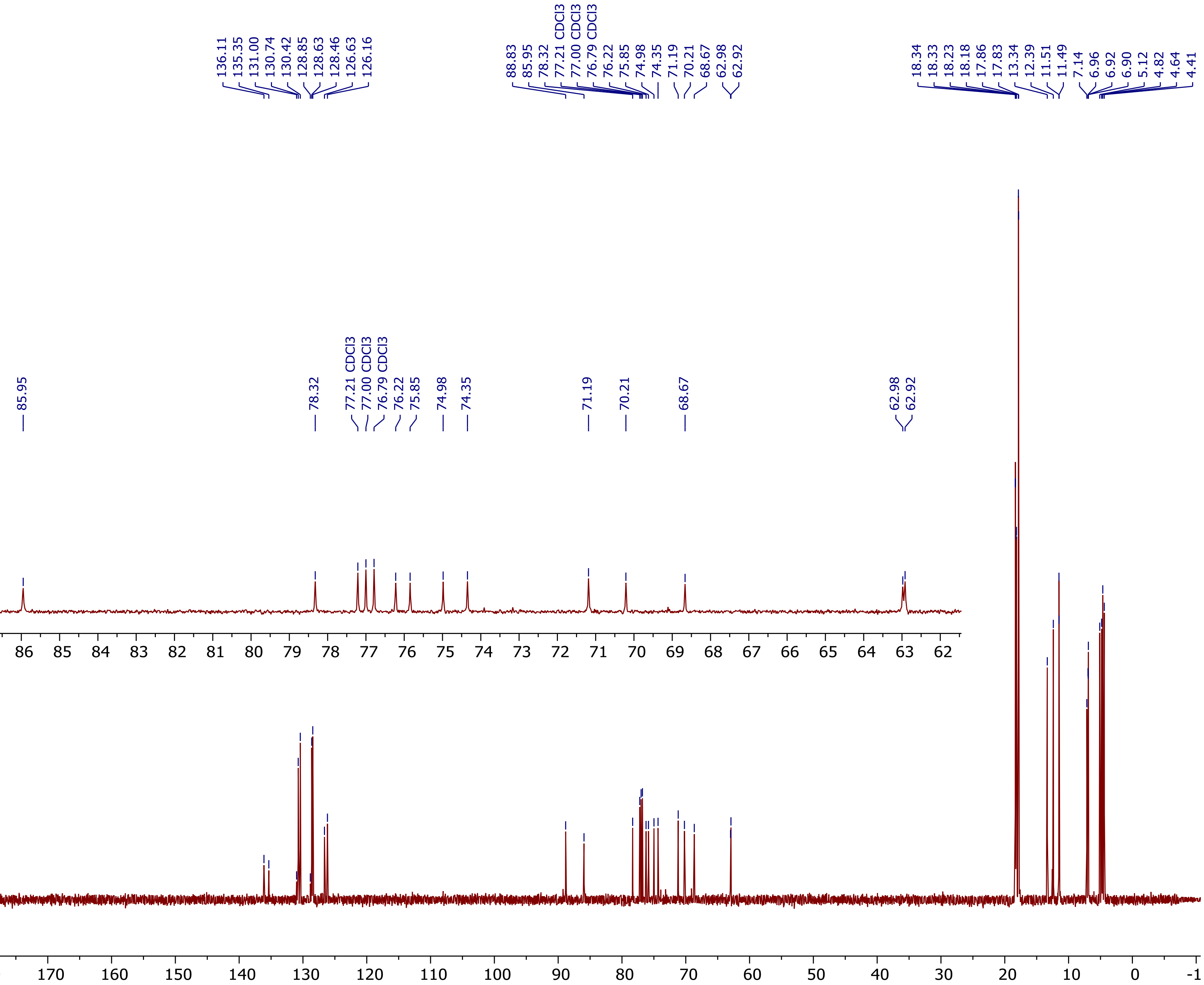

COSY (600 MHz) spectrum of compound 7 in $CDCl_3$ (243K)

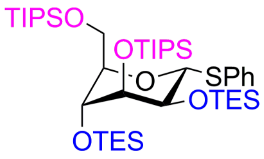

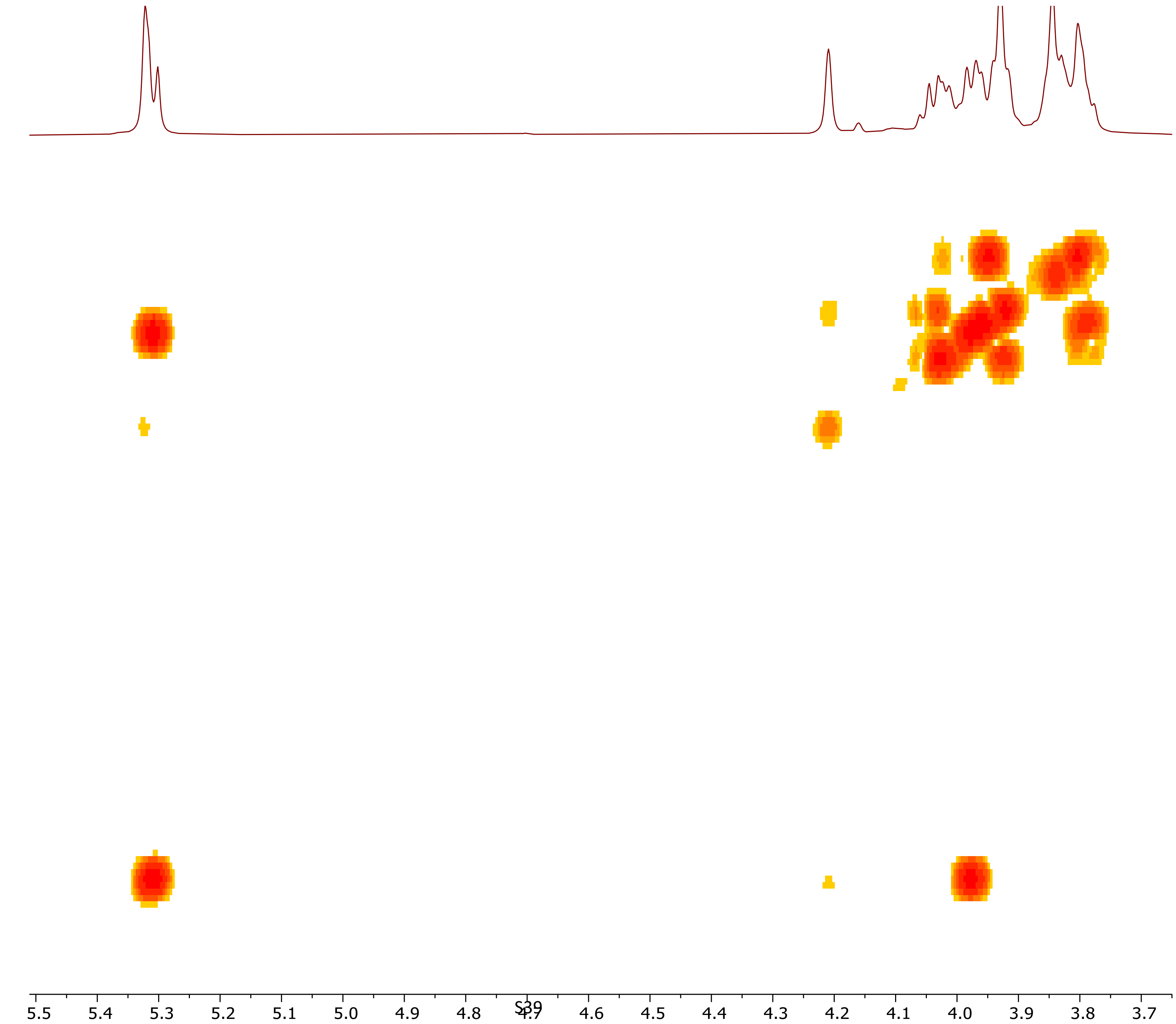

HSQC (600 MHz) spectrum of compound 7 in $CDCl_3$ (243K)

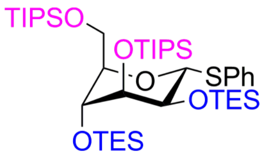

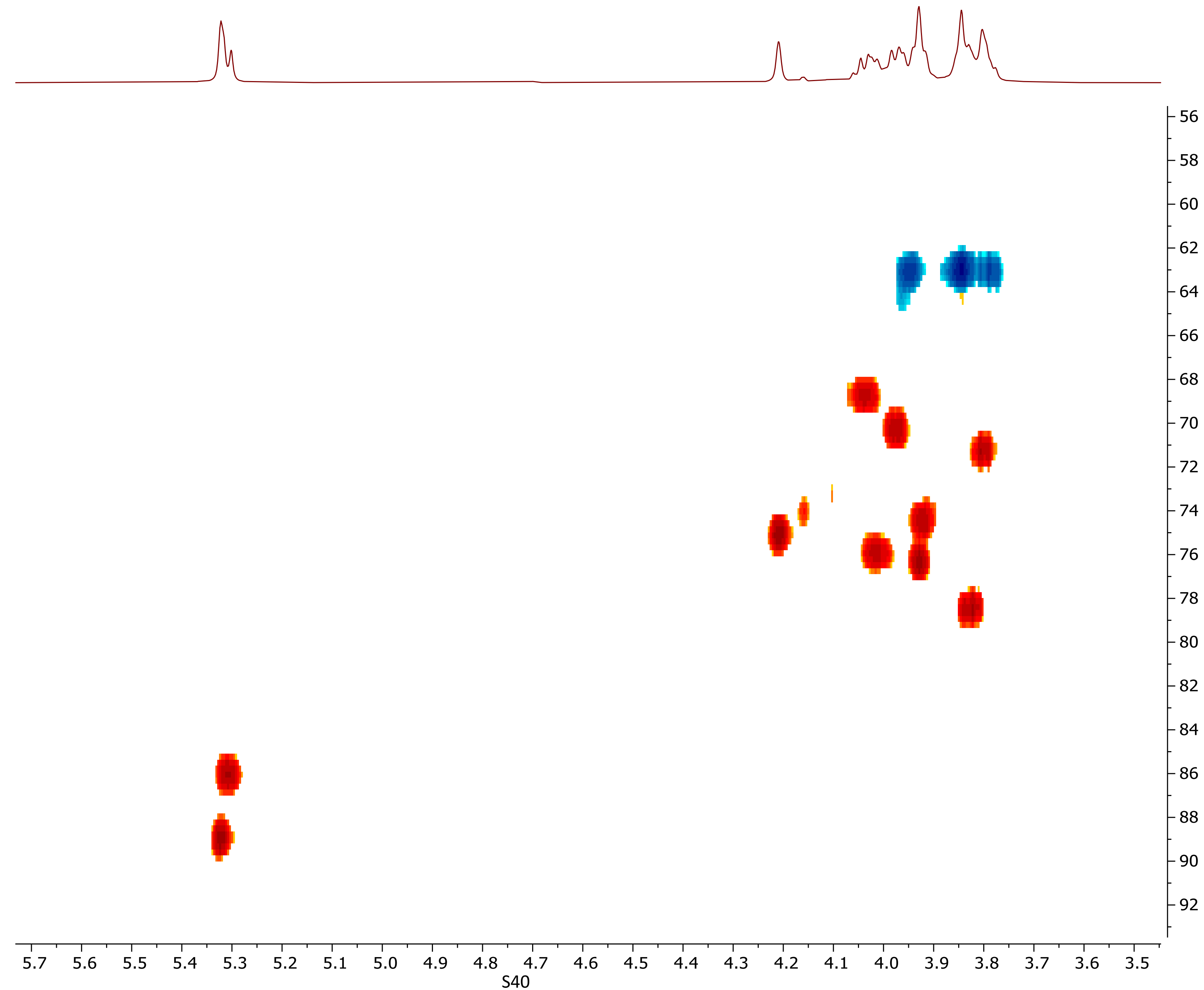

HMBC (600 MHz) spectrum of compound 7 in $CDCl_3$ (243K)

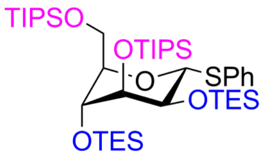

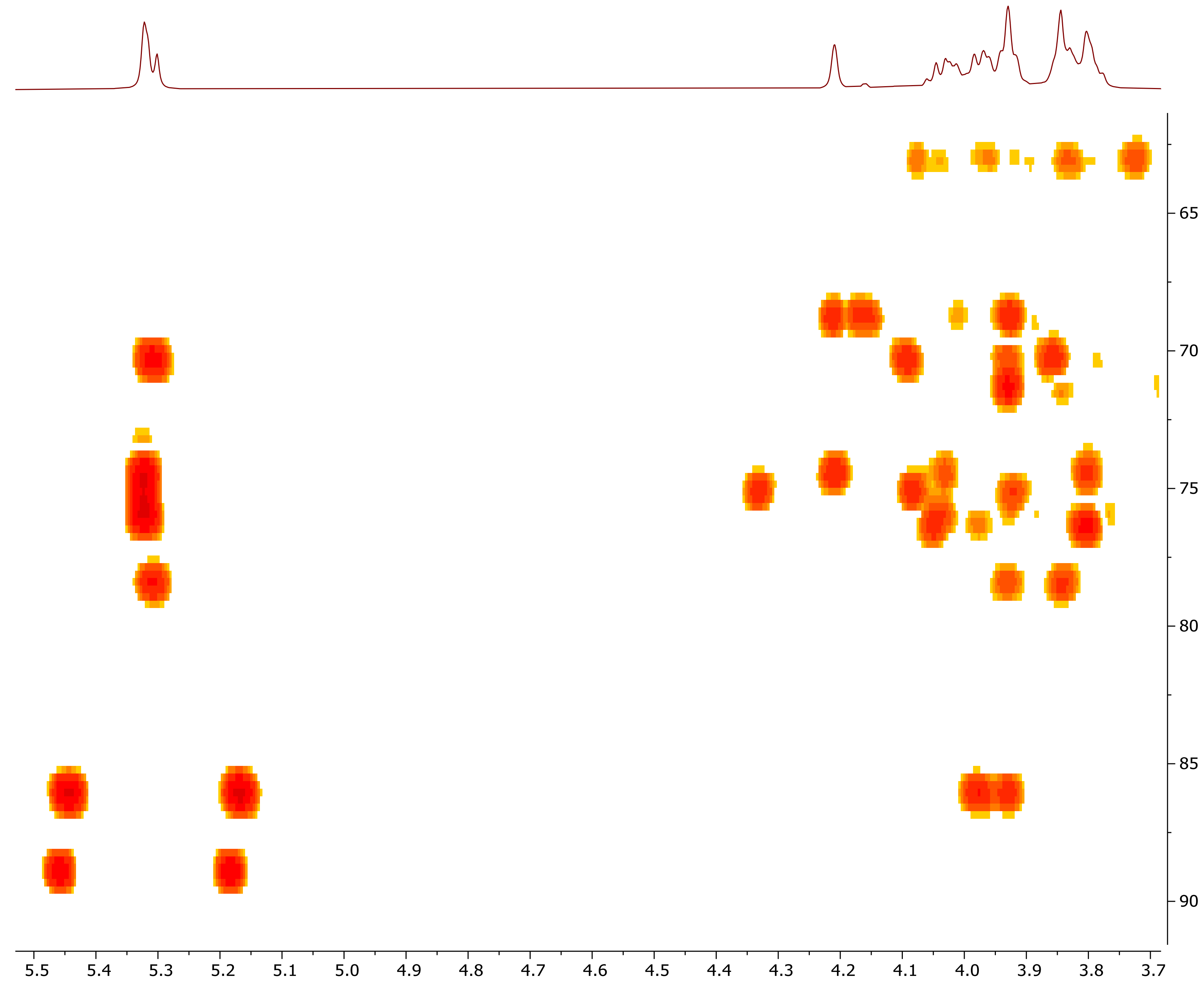

^{1}H–^{29}Si HMBC (600 MHz) spectrum of compound 7 in $CDCl_3$ (243K)

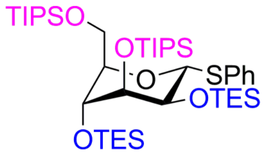

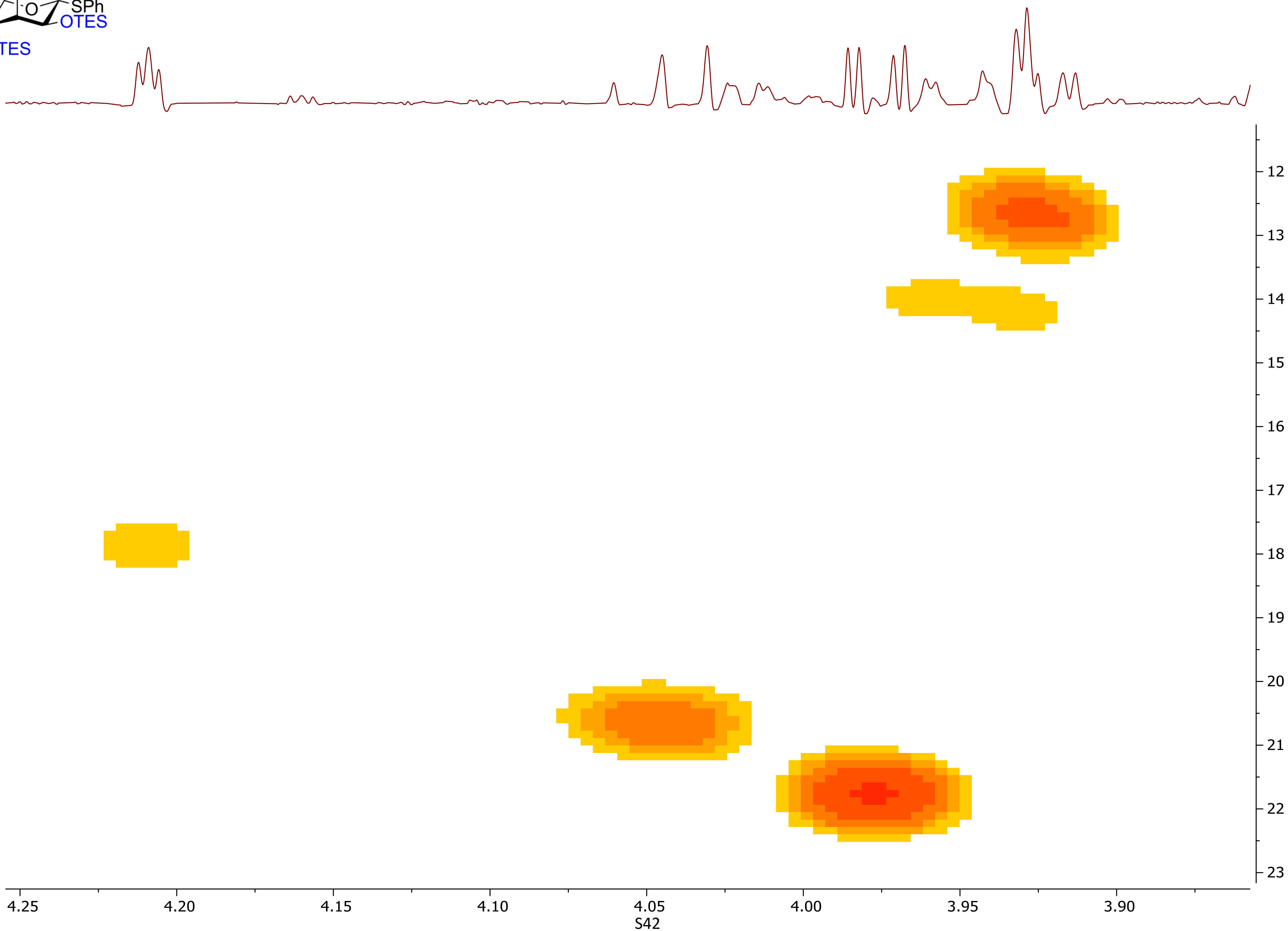

^{1}H NMR (600 MHz) spectrum of compound 10 in $CDCl_3$ (240K)

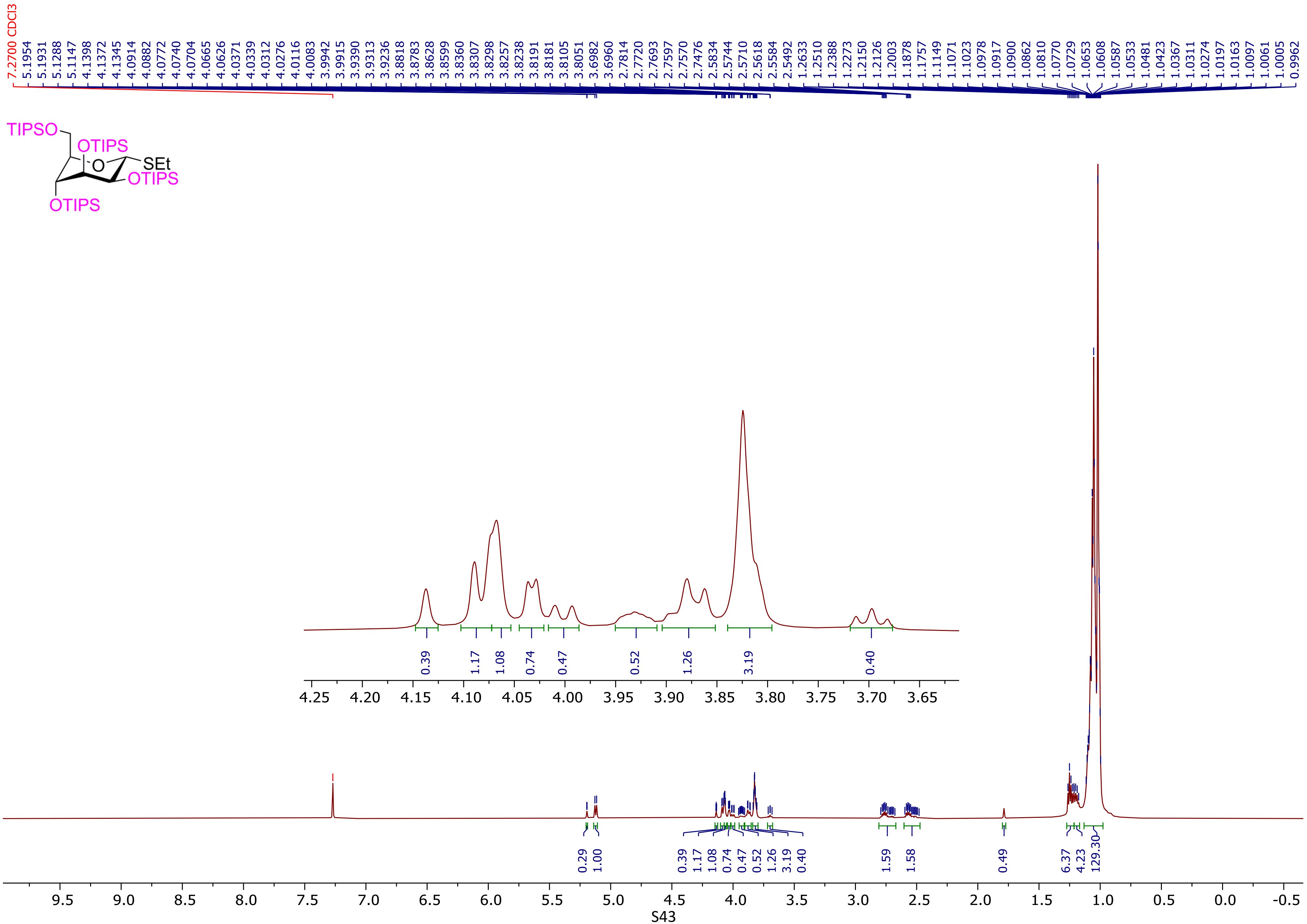

^{13}C NMR (151 MHz) spectrum of compound 10 in $CDCl_3$ (240K)

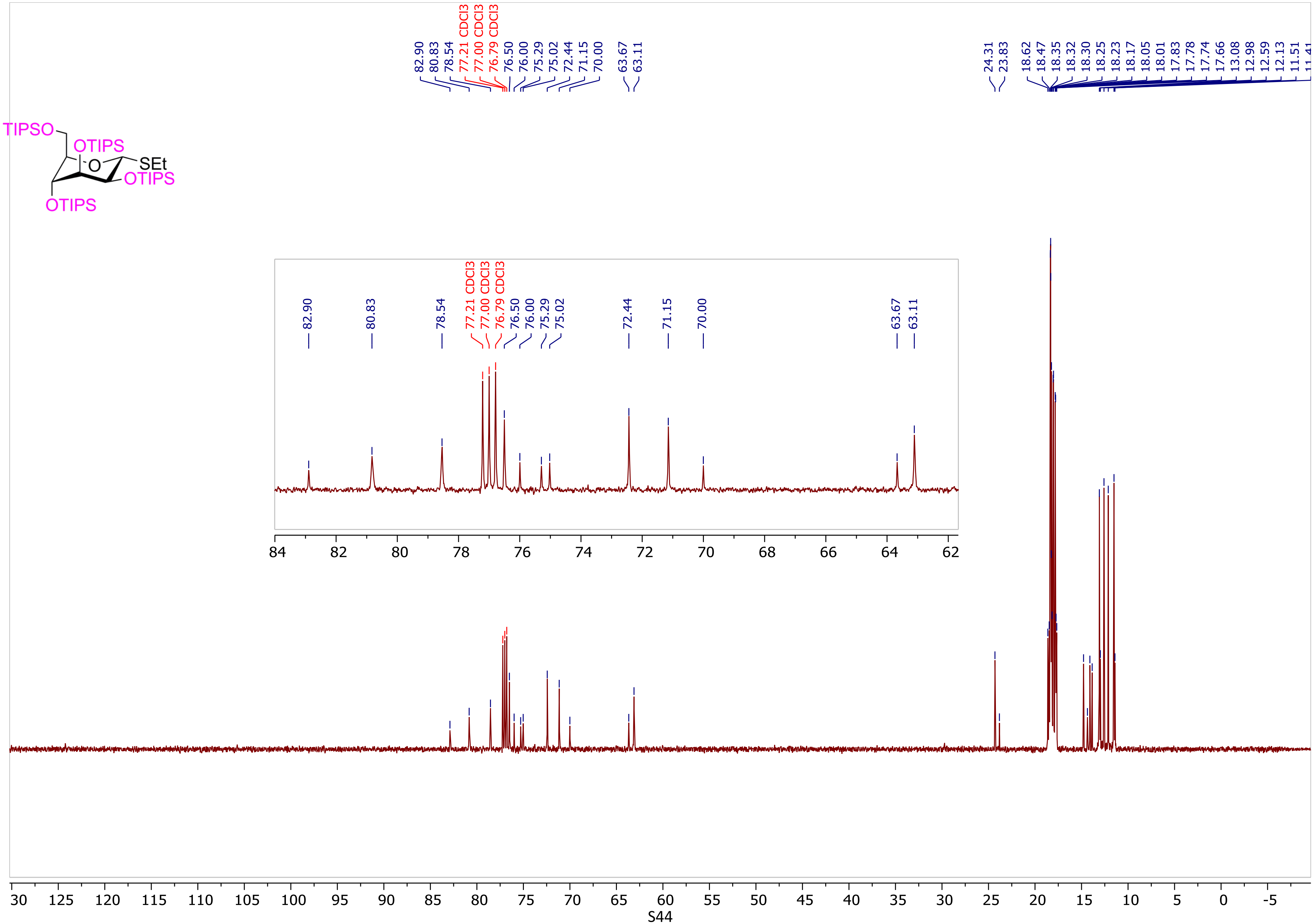

COSY (600 MHz) spectrum of compound 10 in $CDCl_3$ (240K)

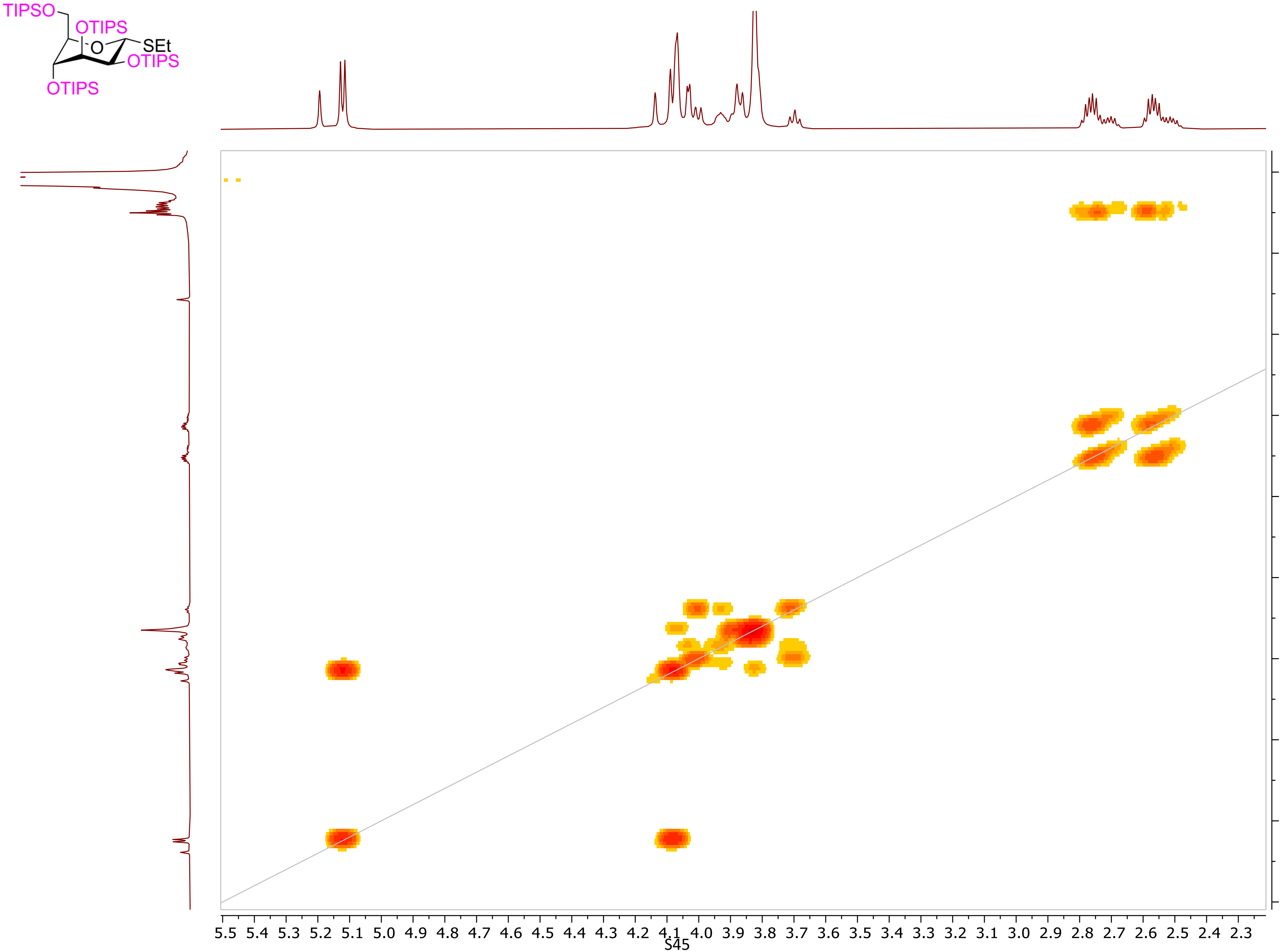

HSQC (600 MHz) spectrum of compound 10 in $CDCl_3$ (240K)

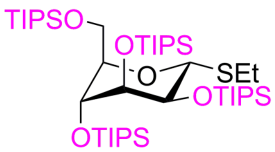

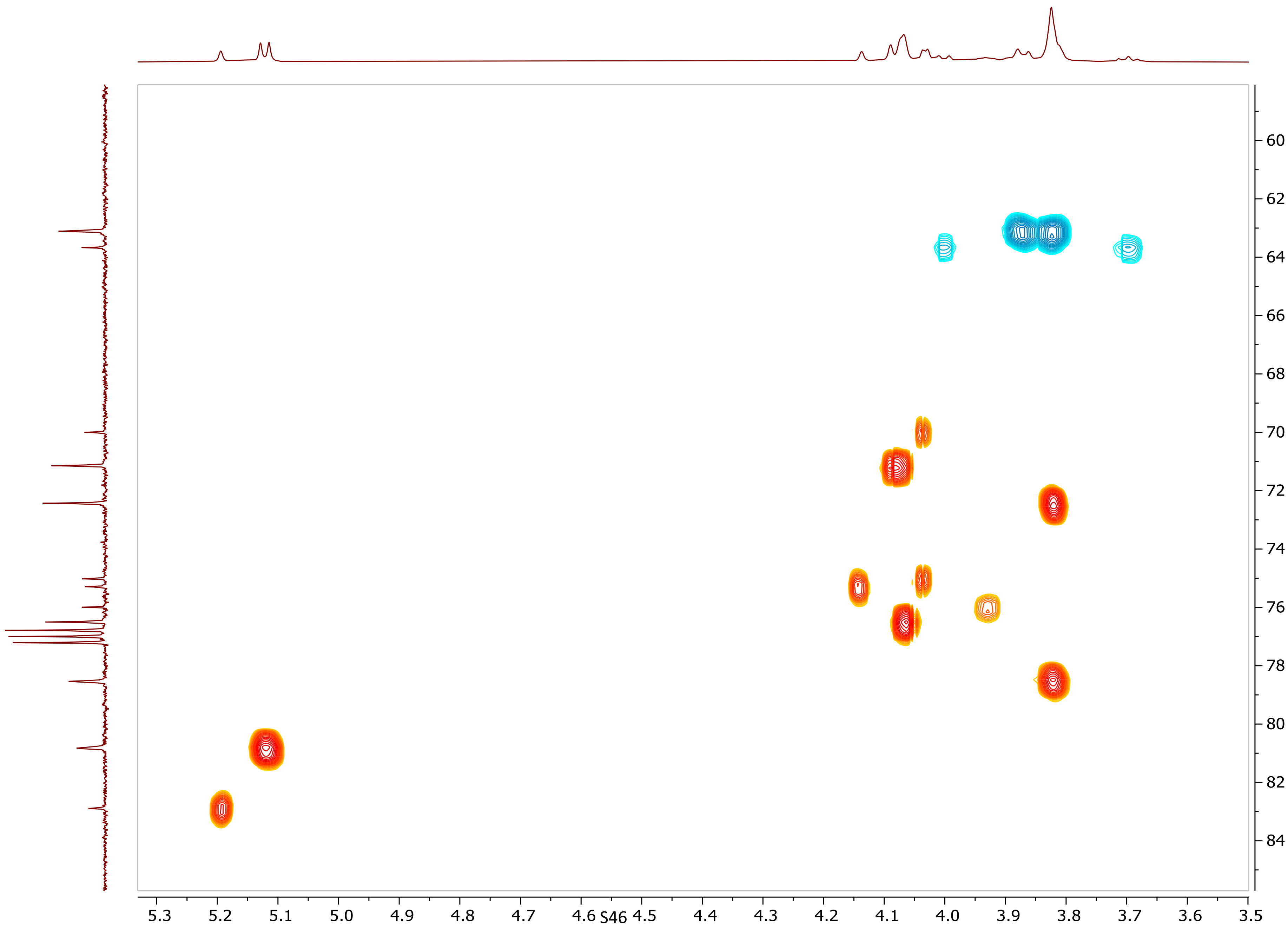

HMBC (600 MHz) spectrum of compound 10 in $CDCl_3$ (240K)

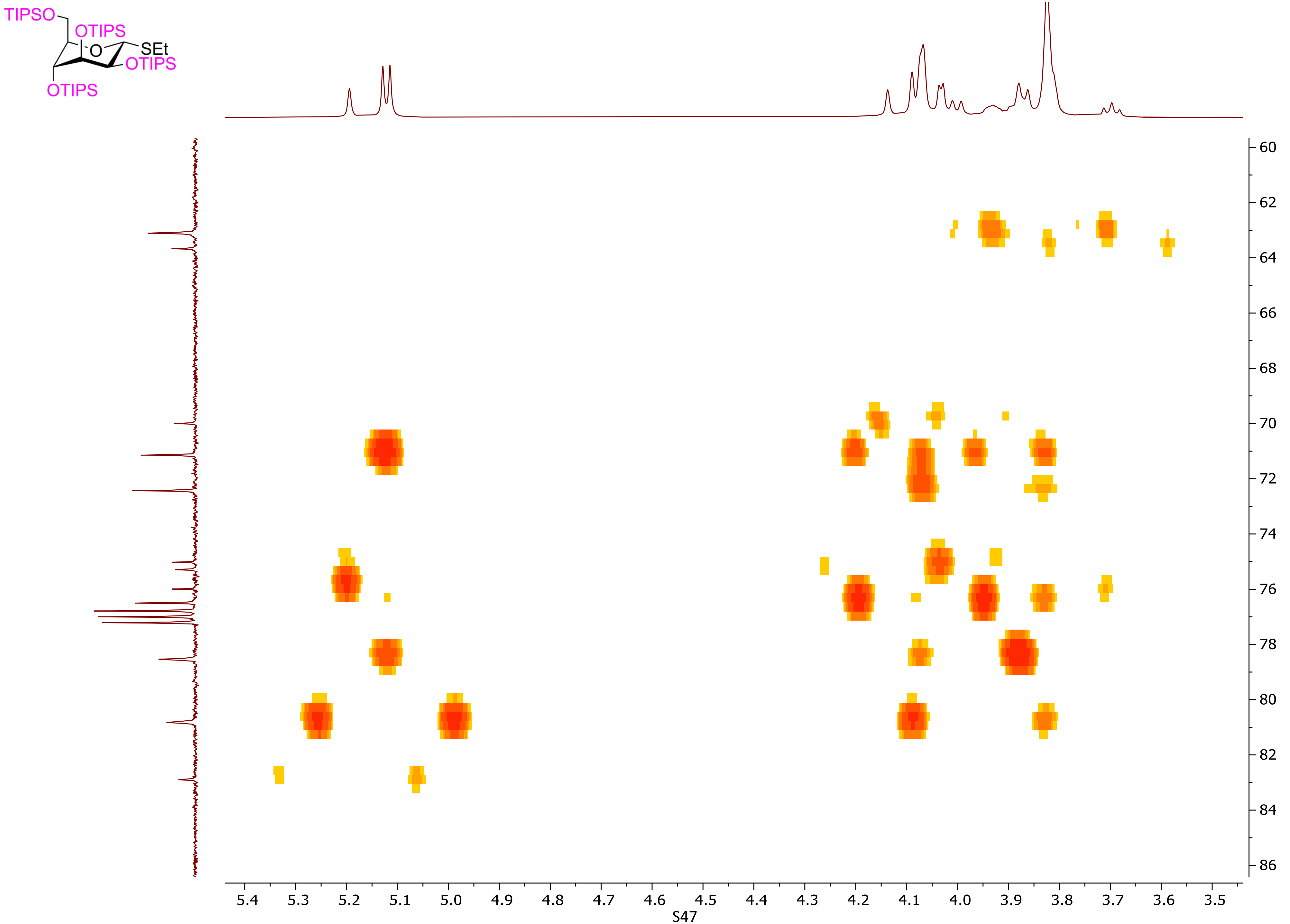

^{1}H–^{29}Si HMBC (600 MHz) spectrum of compound 10 in $CDCl_3$ (240K)

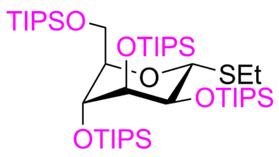

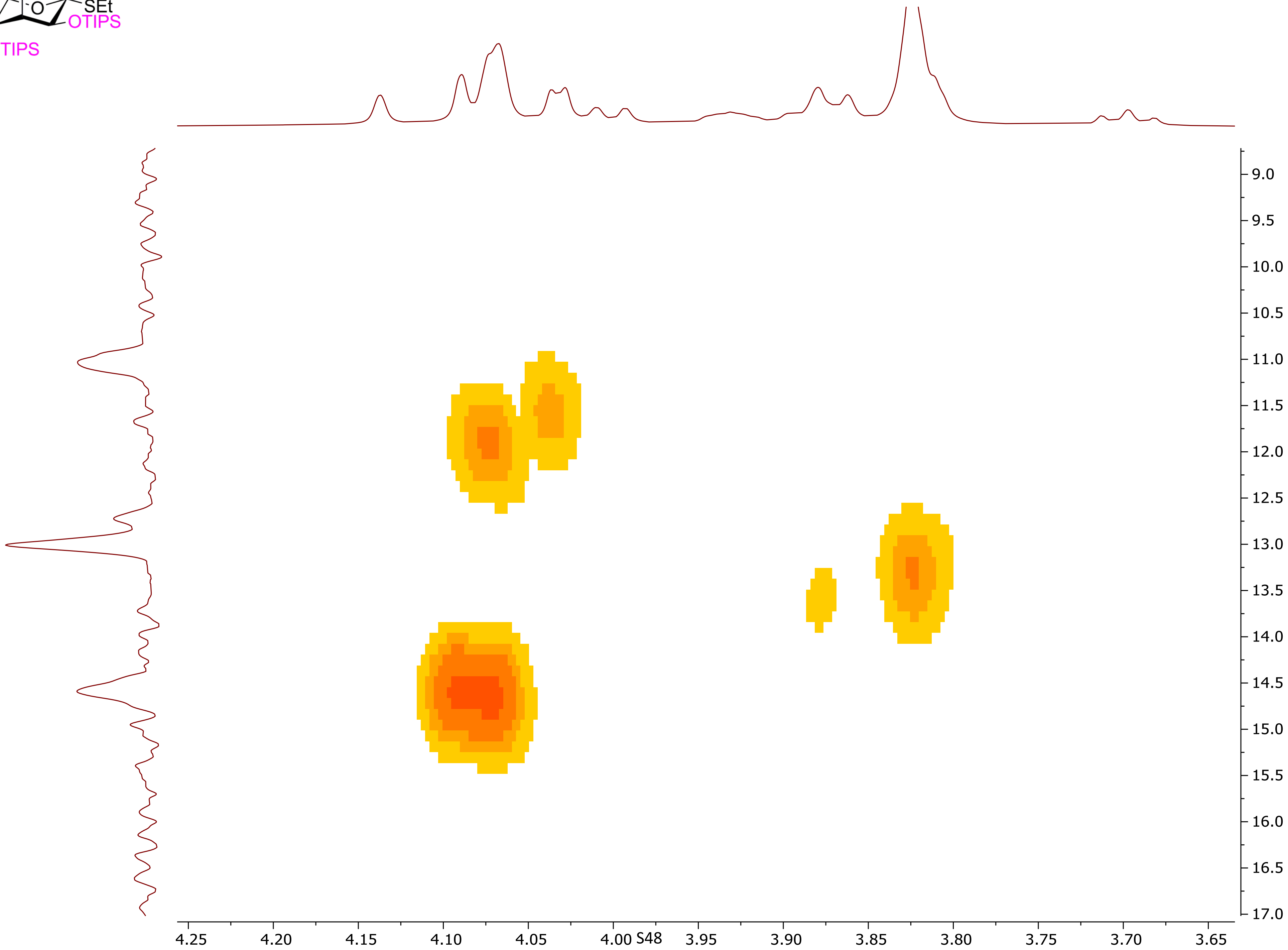

^{29}Si INEPT NMR (60 MHz) spectrum of compound 10 in $CDCl_3$ (240K)

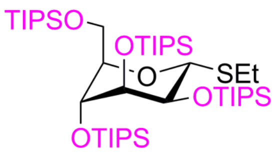

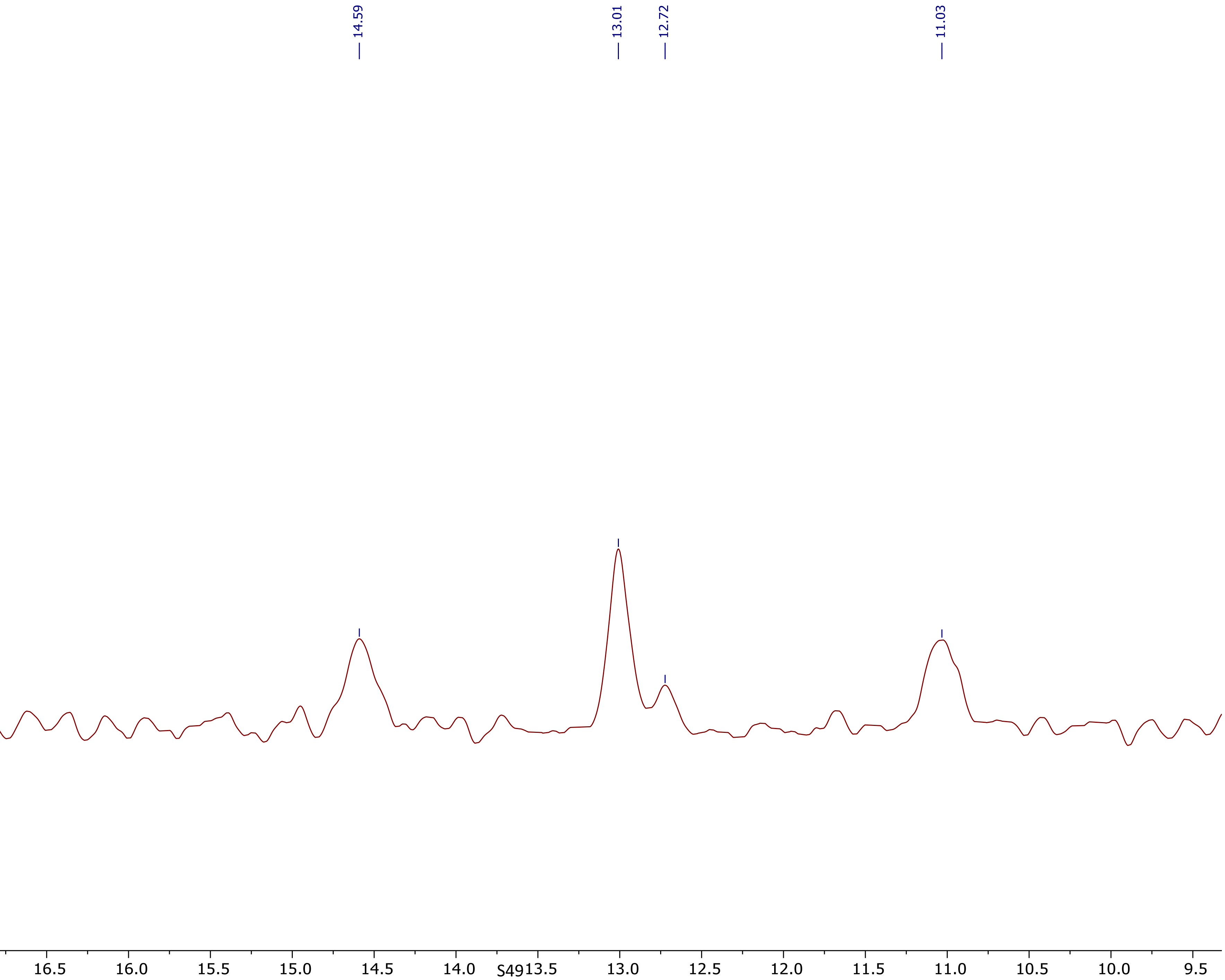

^{1}H NMR (600 MHz) spectrum of compound 11 in $CDCl_3$

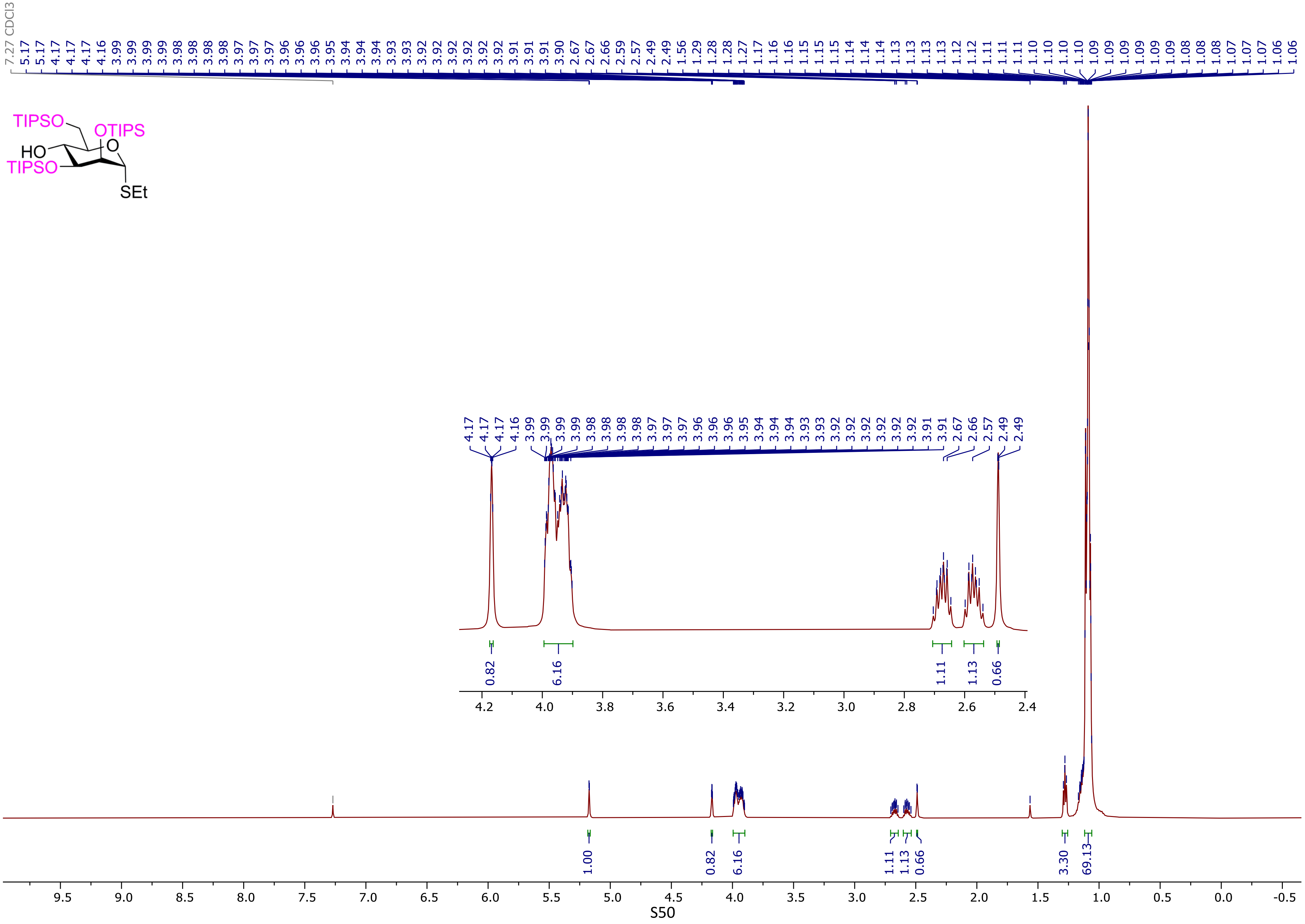

^{13}C NMR (151 MHz) spectrum of compound 11 in $CDCl_3$

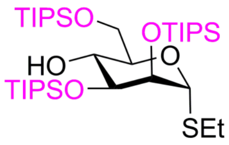

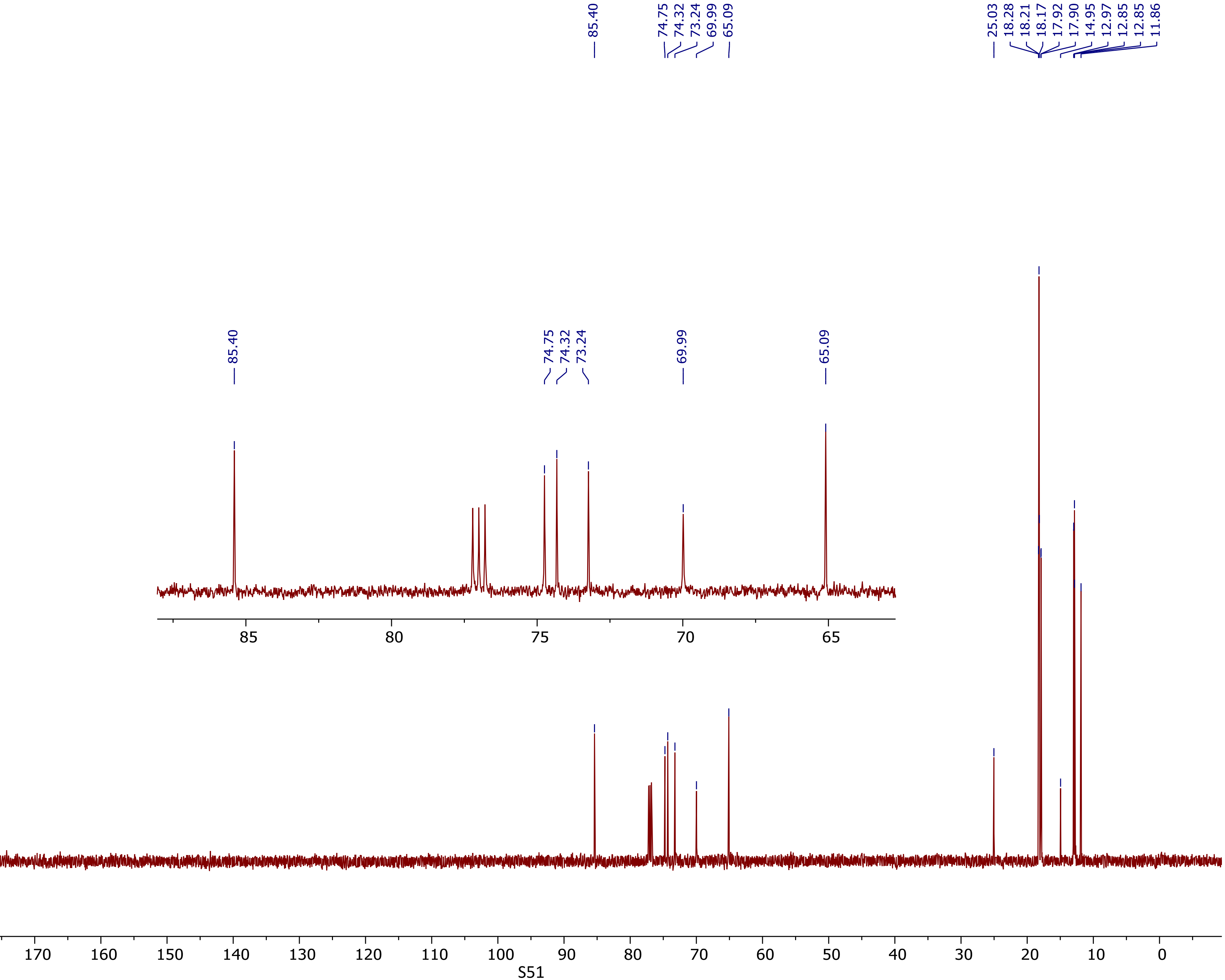

COSY (600 MHz) spectrum of compound 11 in $CDCl_3$

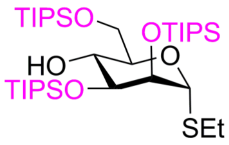

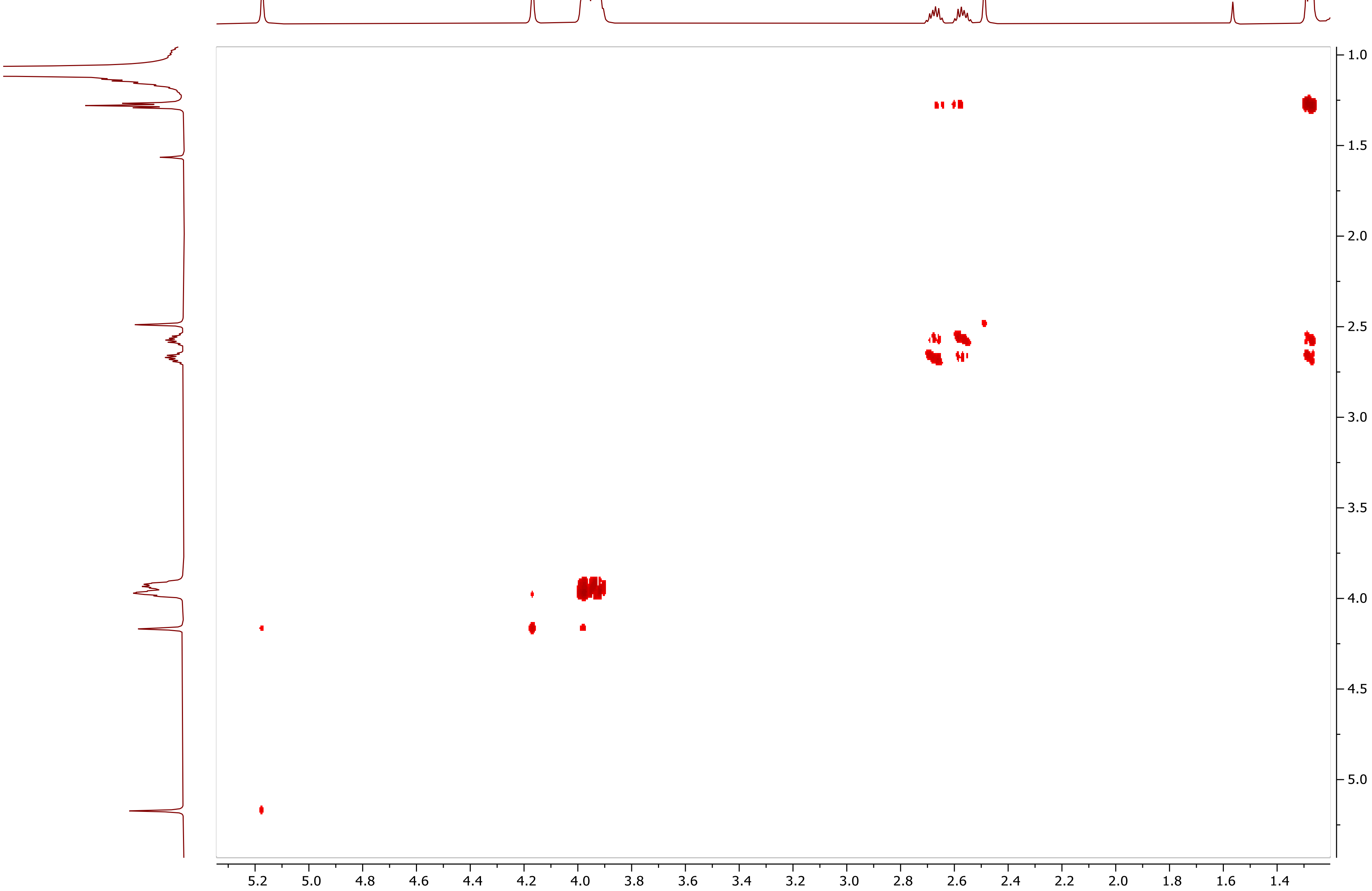

HSQC (600 MHz) spectrum of compound 11 in $CDCl_3$

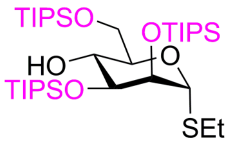

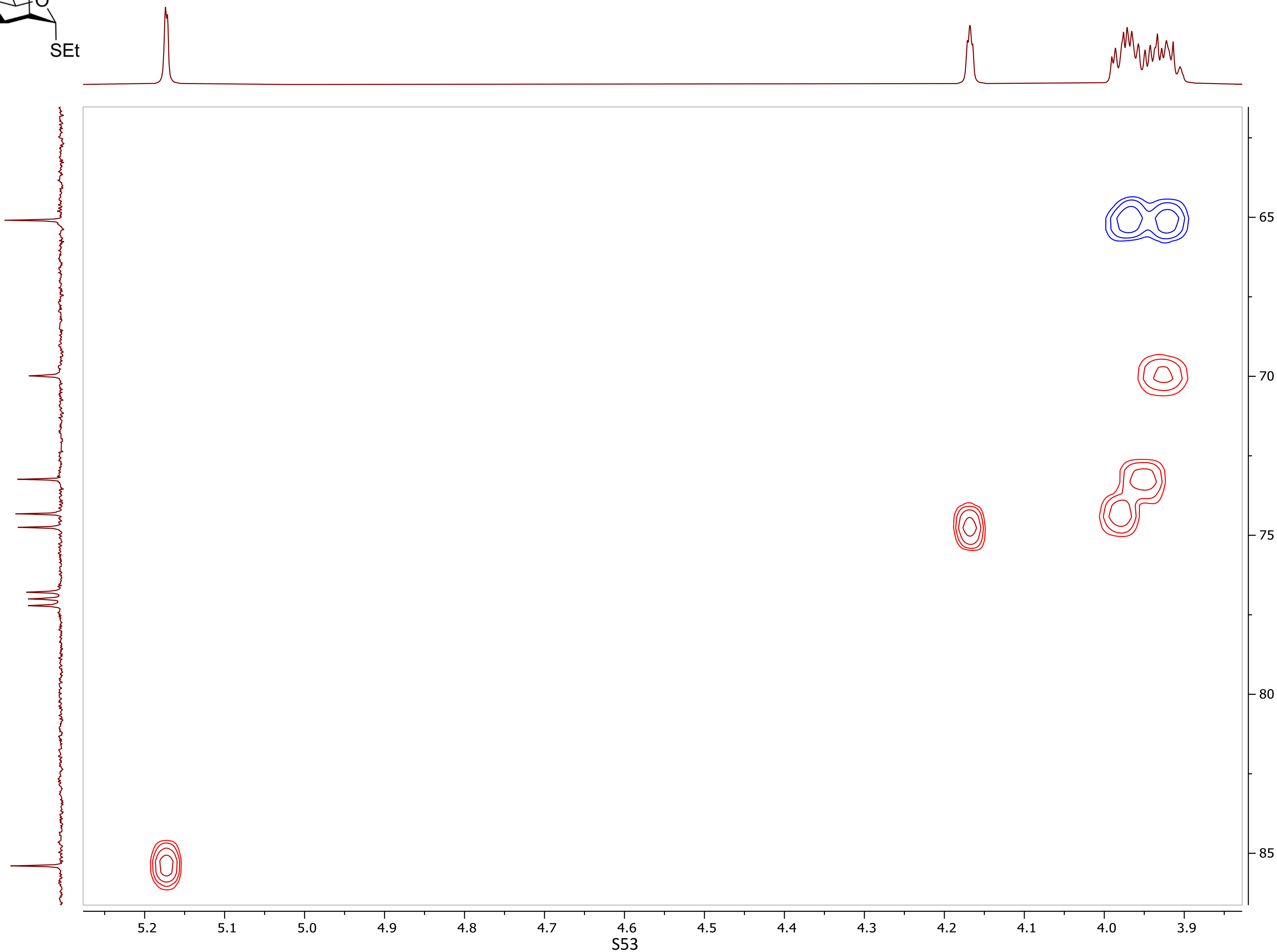

HMBC (600 MHz) spectrum of compound 12 in $CDCl_3$

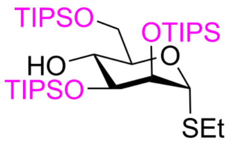

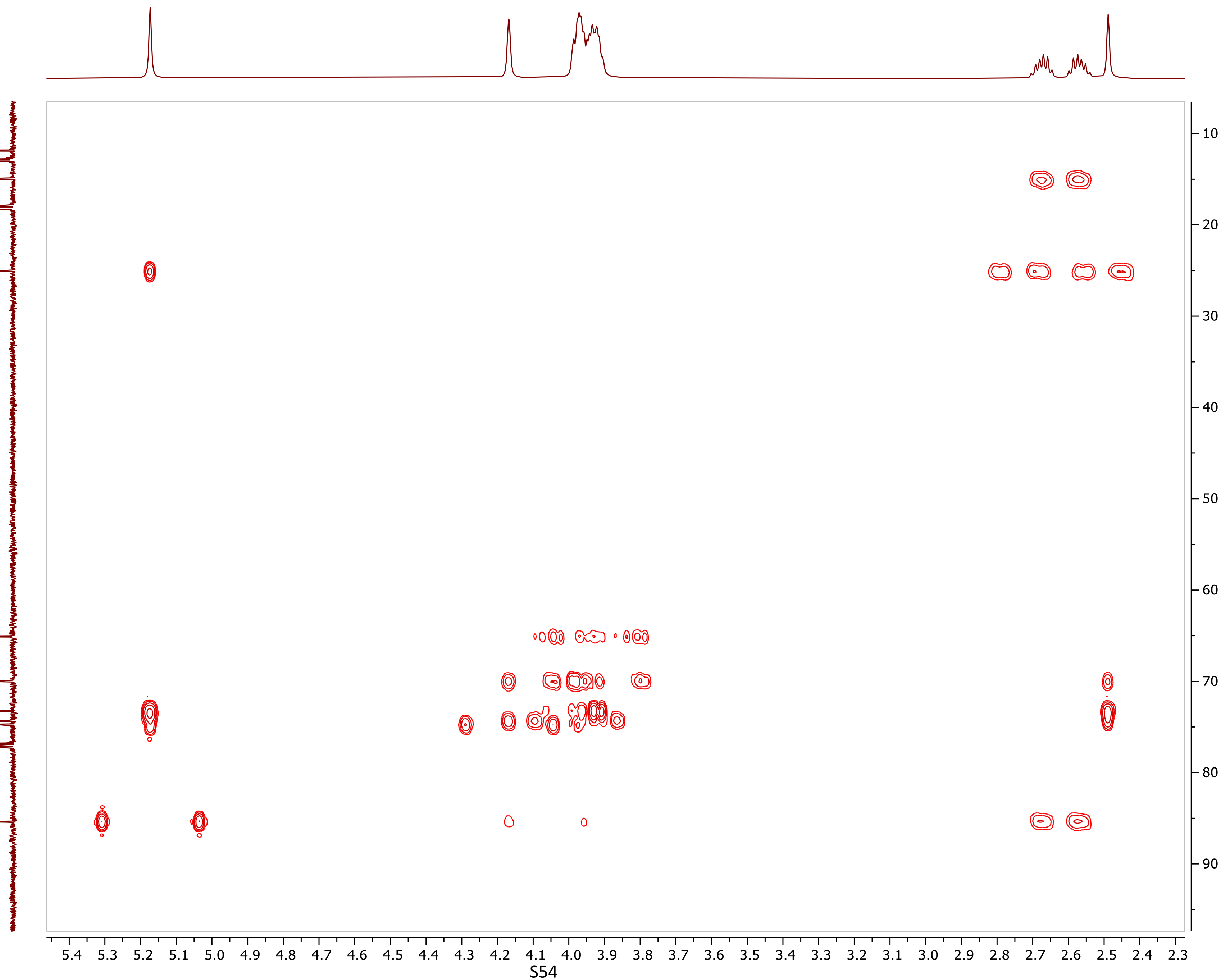

^{1}H NMR (600 MHz) spectrum of compound 12 in $CDCl_3$

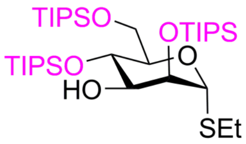

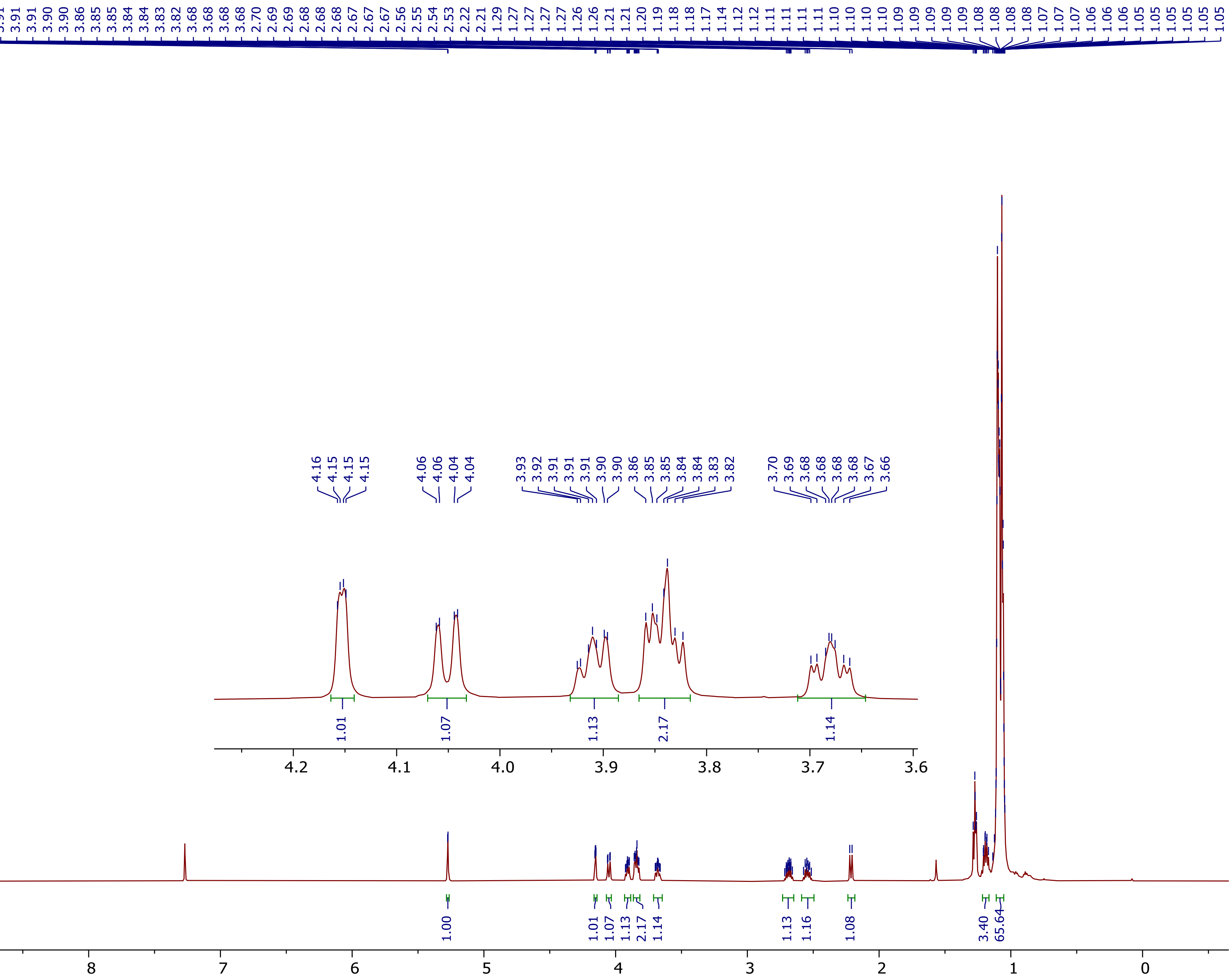

^{13}C NMR (151 MHz) spectrum of compound 12 in $CDCl_3$

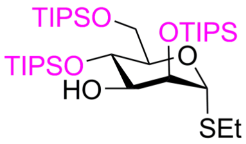

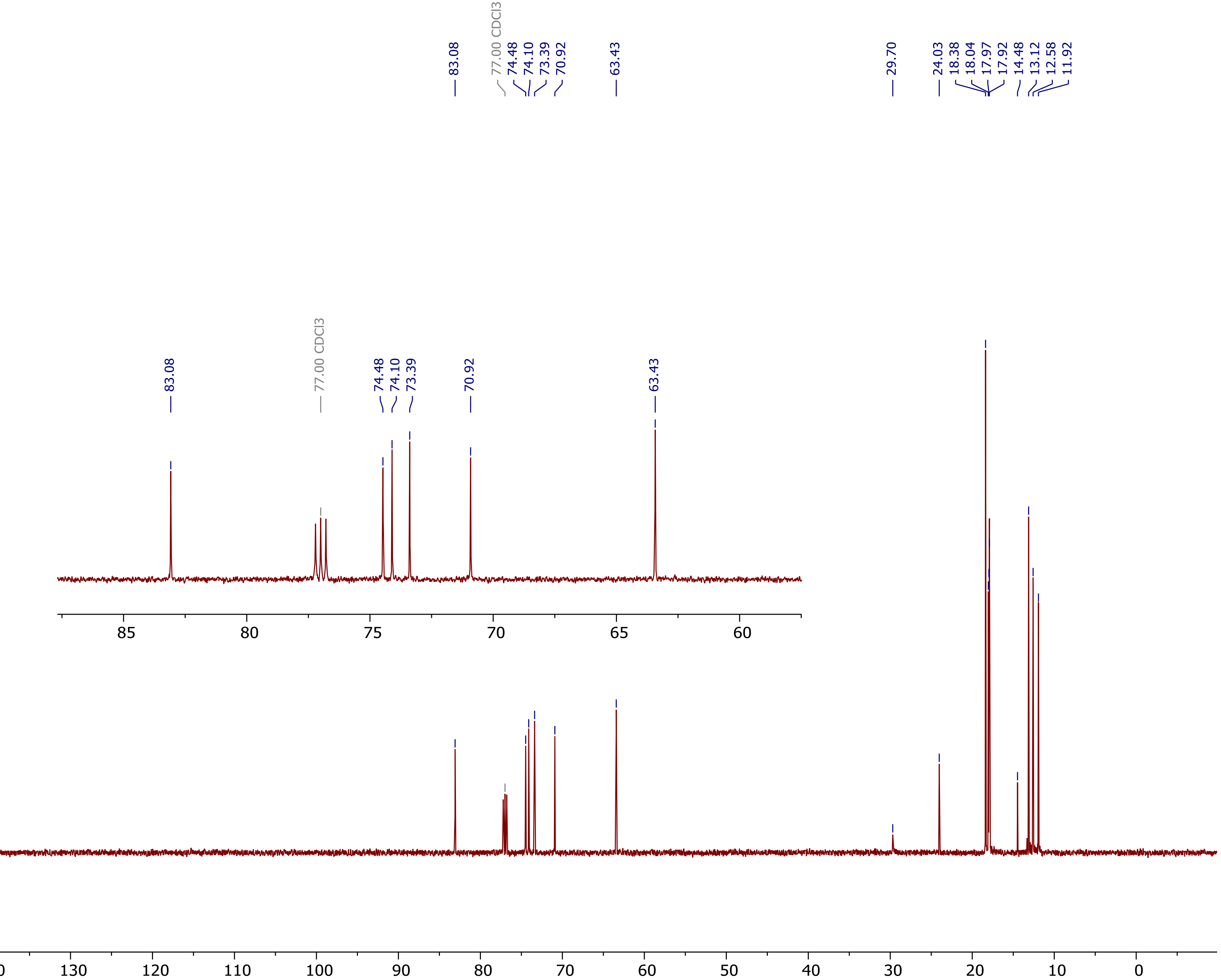

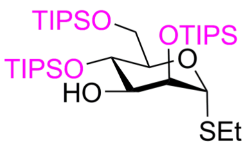

COSY (600 MHz) spectrum of compound 12 in $CDCl_3$

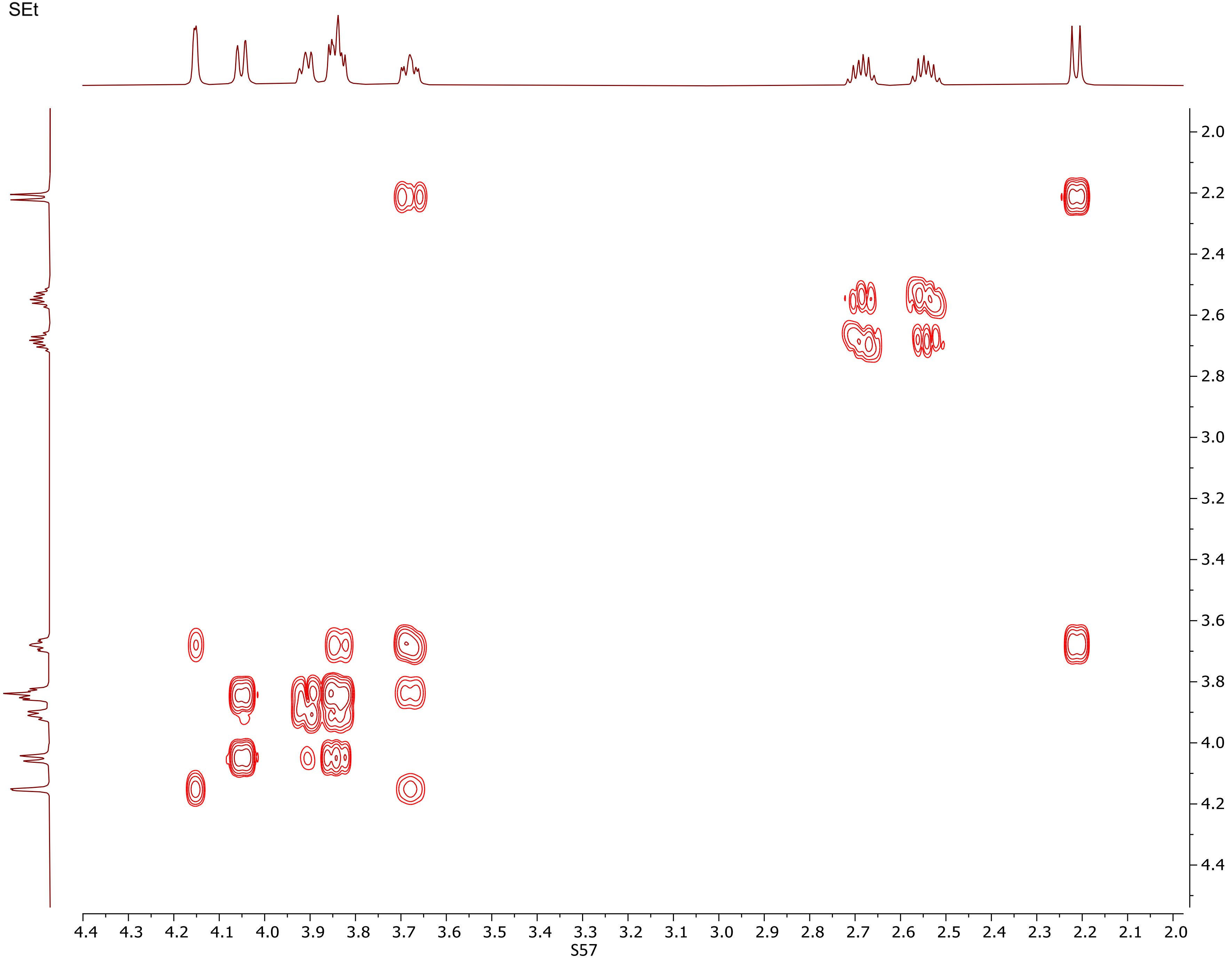

HSQC (600 MHz) spectrum of compound 12 in $CDCl_3$

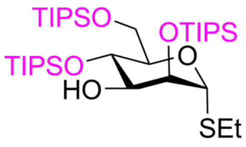

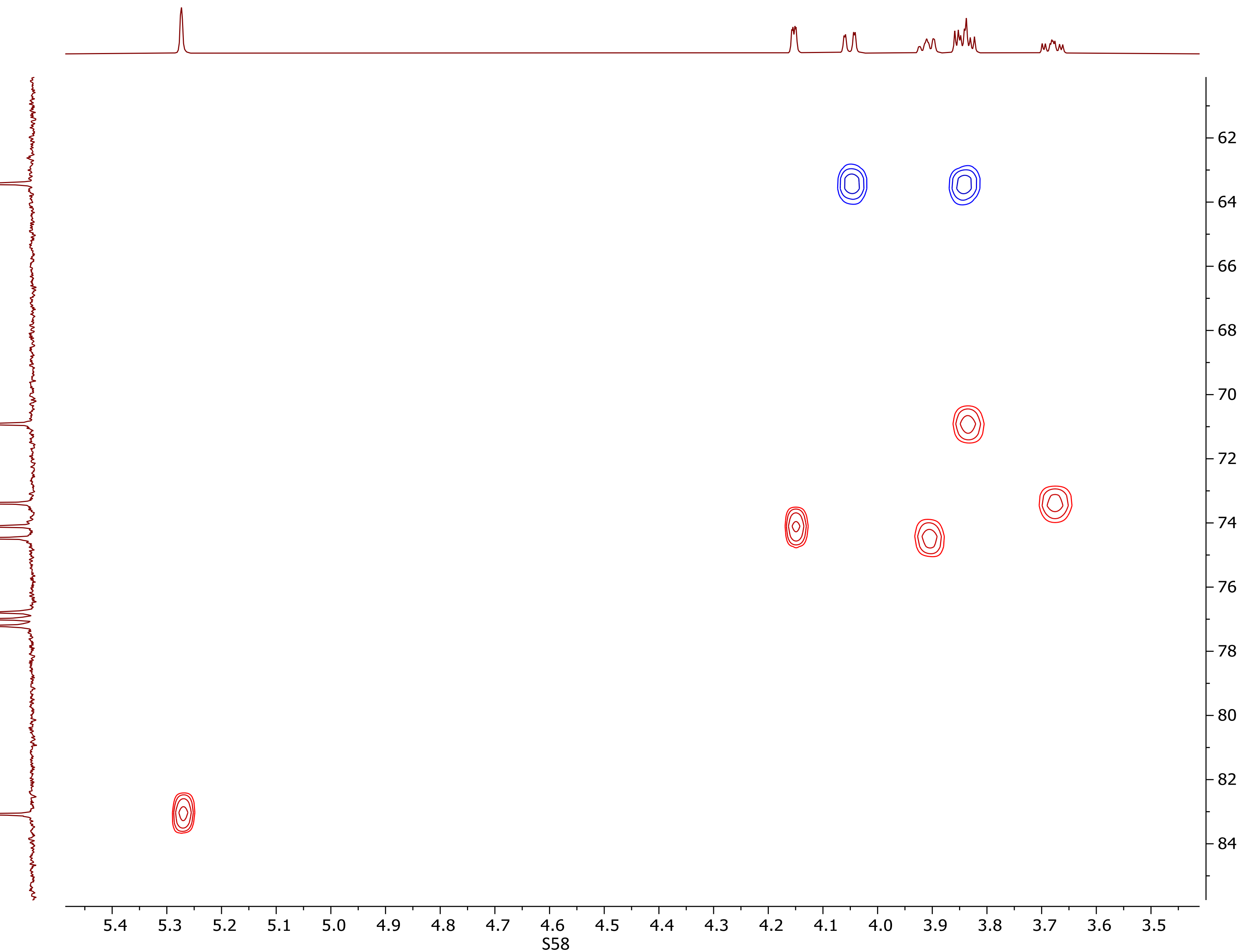

HMBC (600 MHz) spectrum of compound 12 in $CDCl_3$

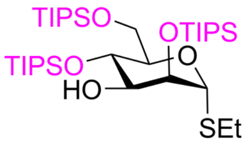

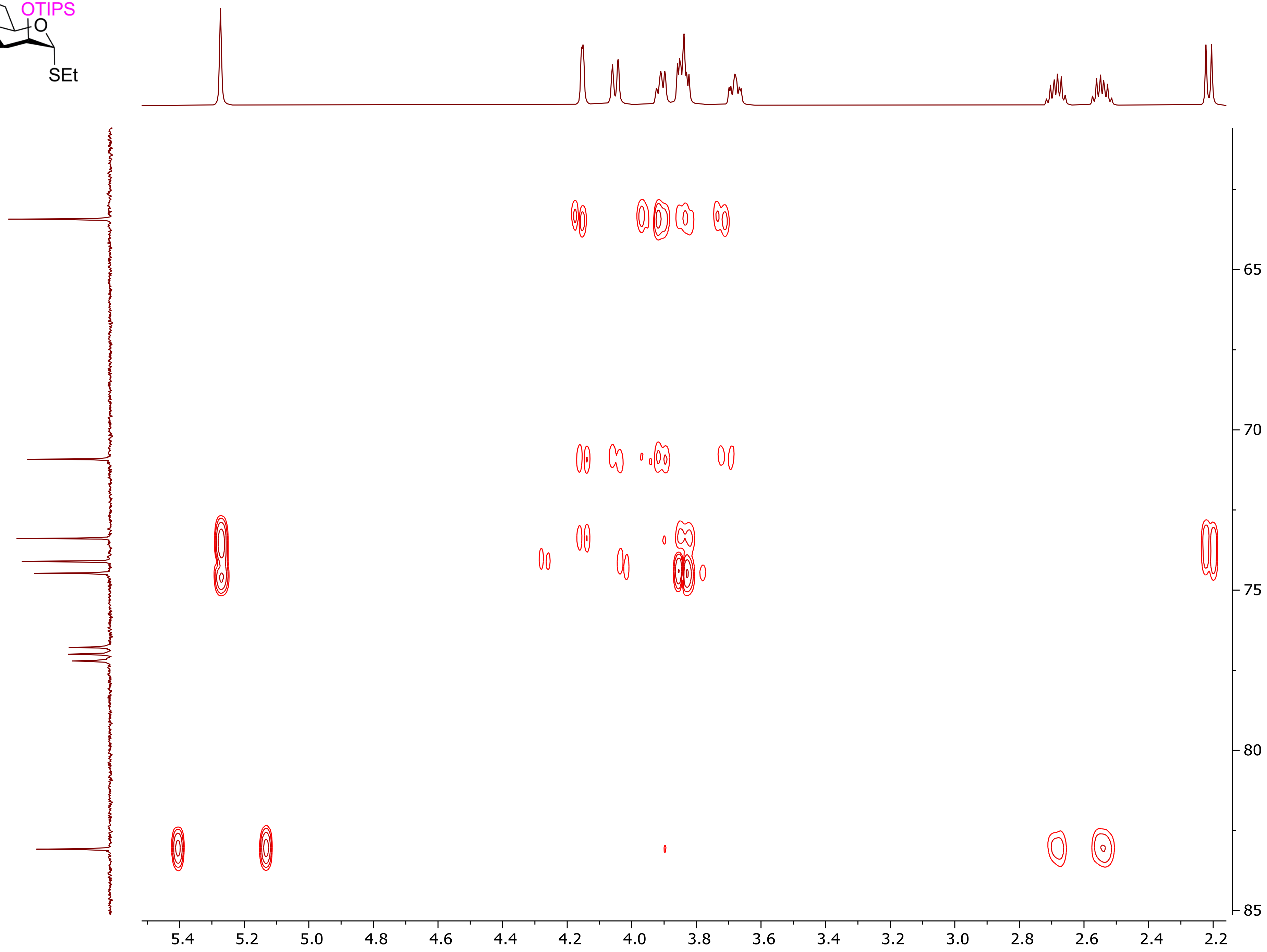

^{1}H NMR (600 MHz) spectrum of compound 13 in $CDCl_3$ (240K)

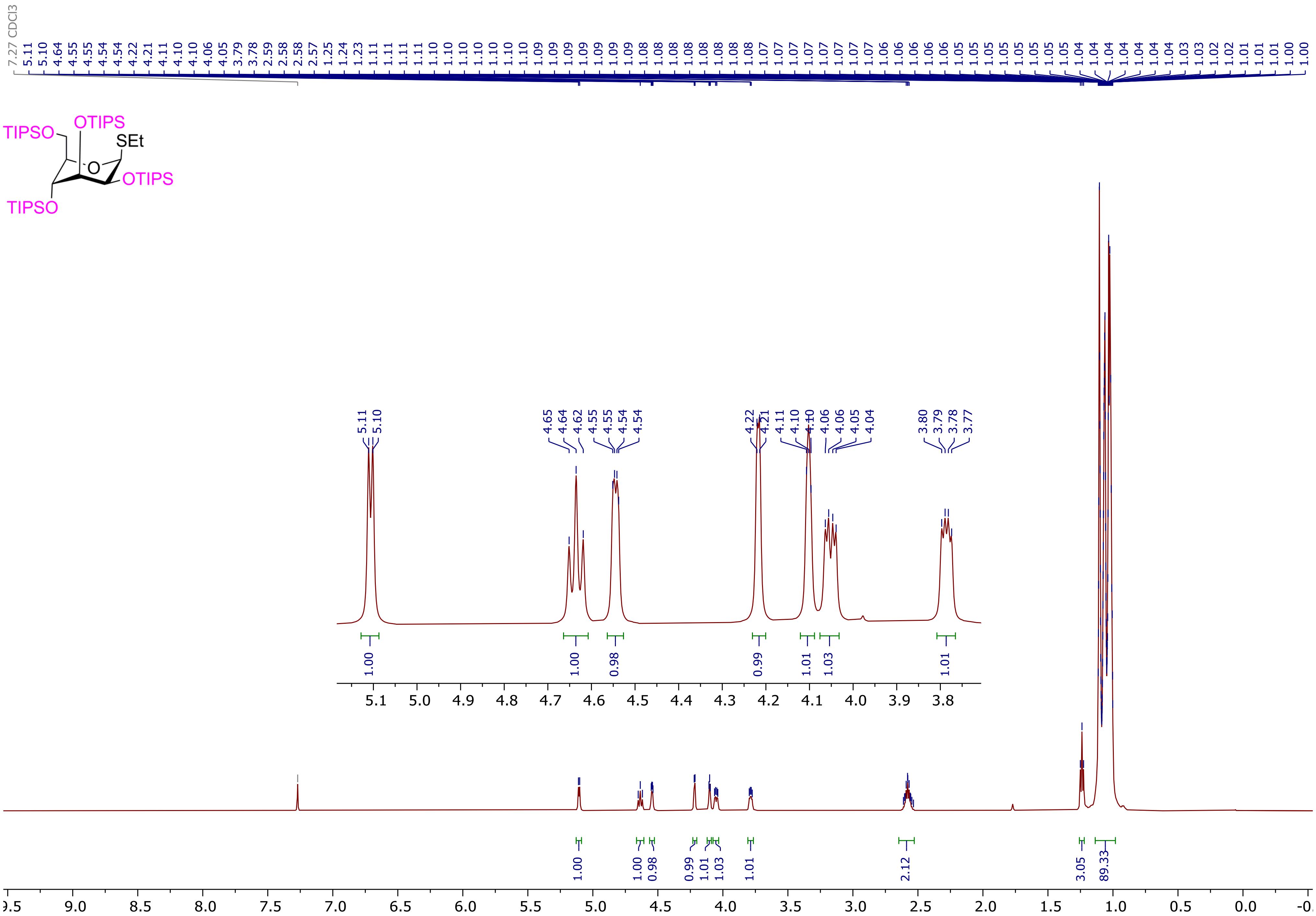

^{13}C NMR (151 MHz) spectrum of compound 13 in $CDCl_3$ (240K)

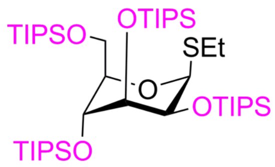

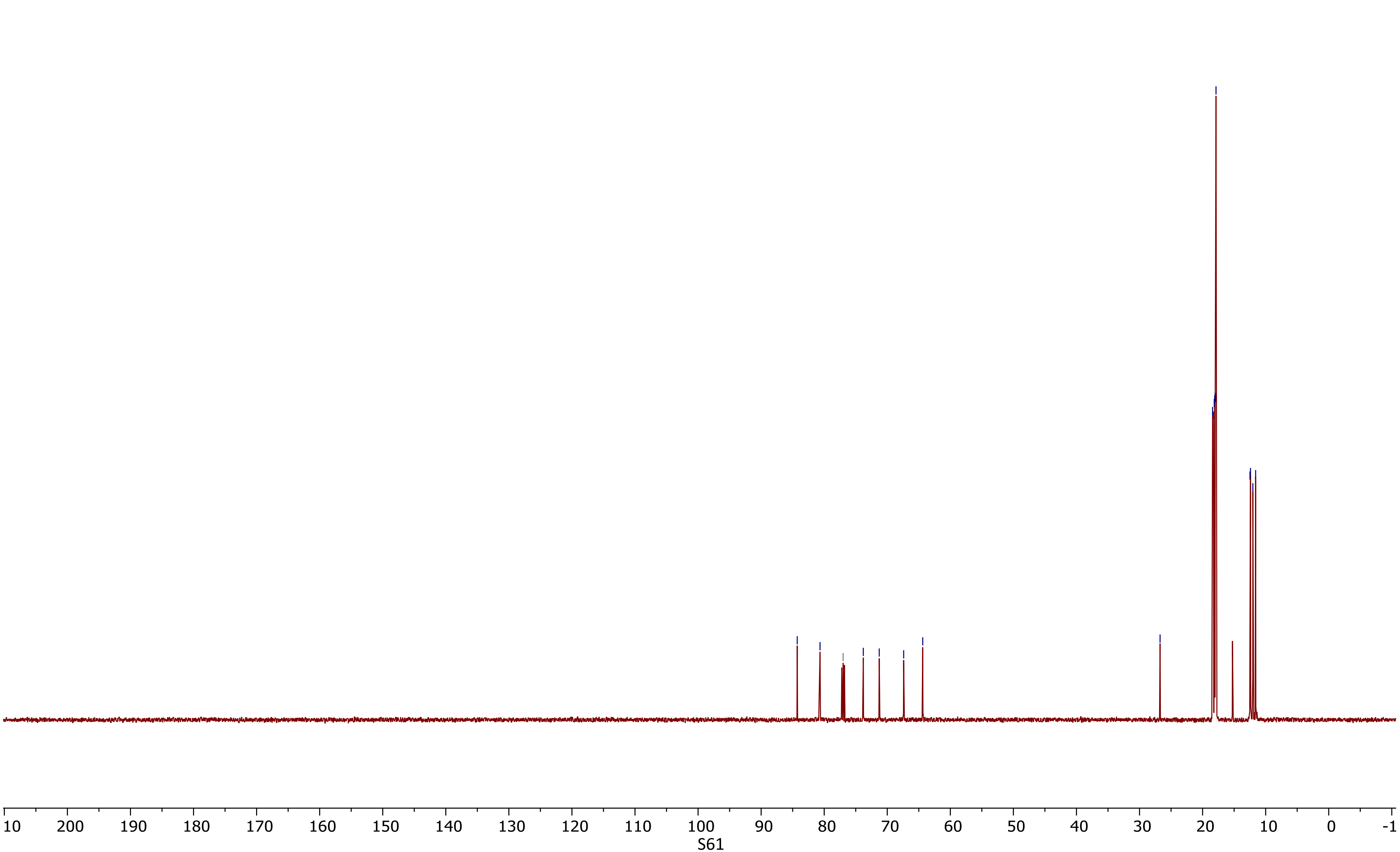

COSY (600 MHz) spectrum of compound 13 in $CDCl_3$ (240K)

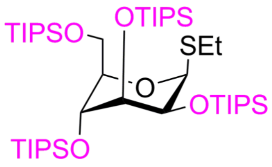

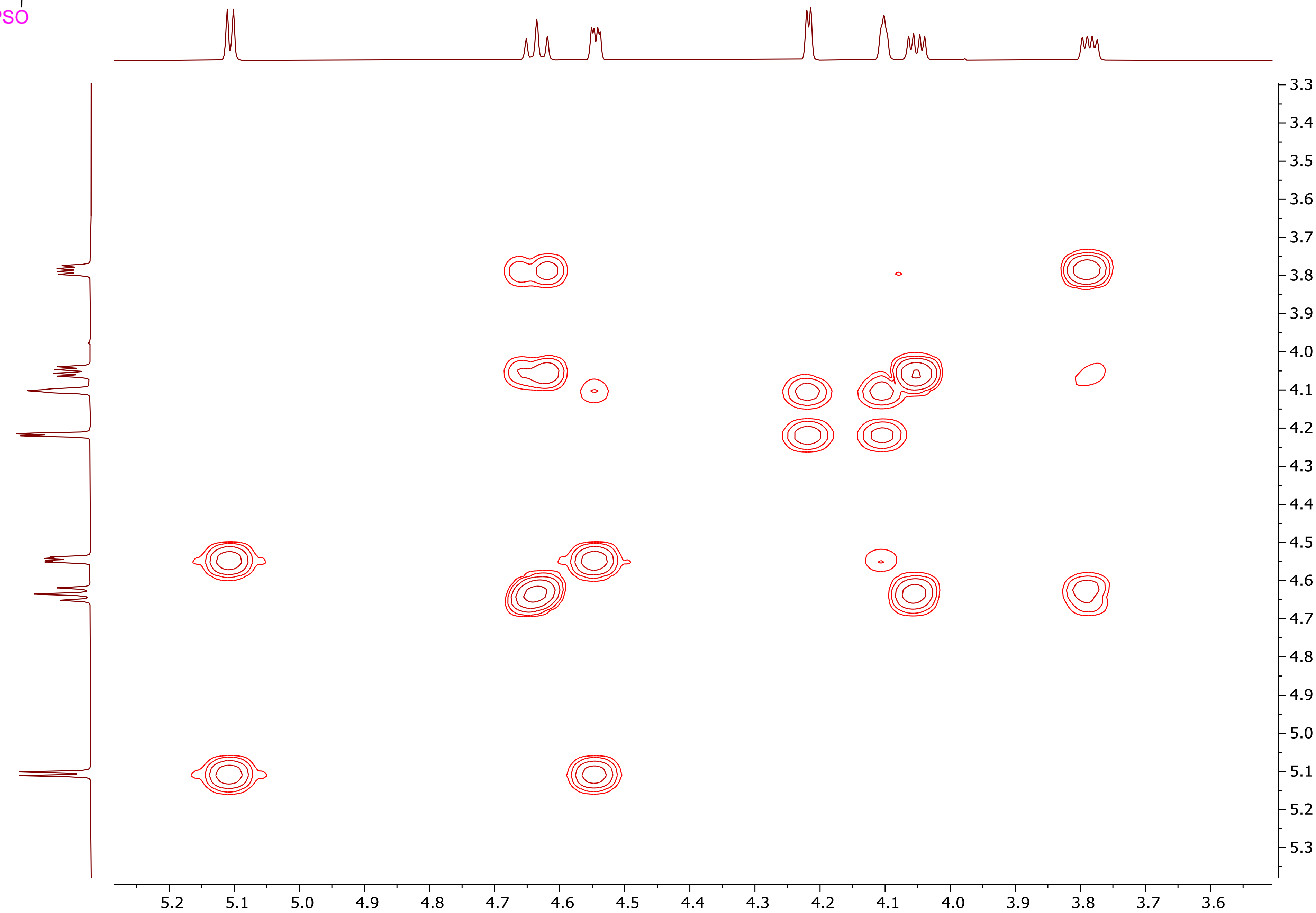

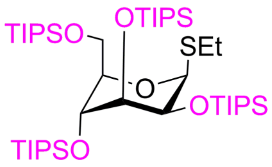

HSQC (600 MHz) spectrum of compound 13 in $CDCl_3$ (240K)

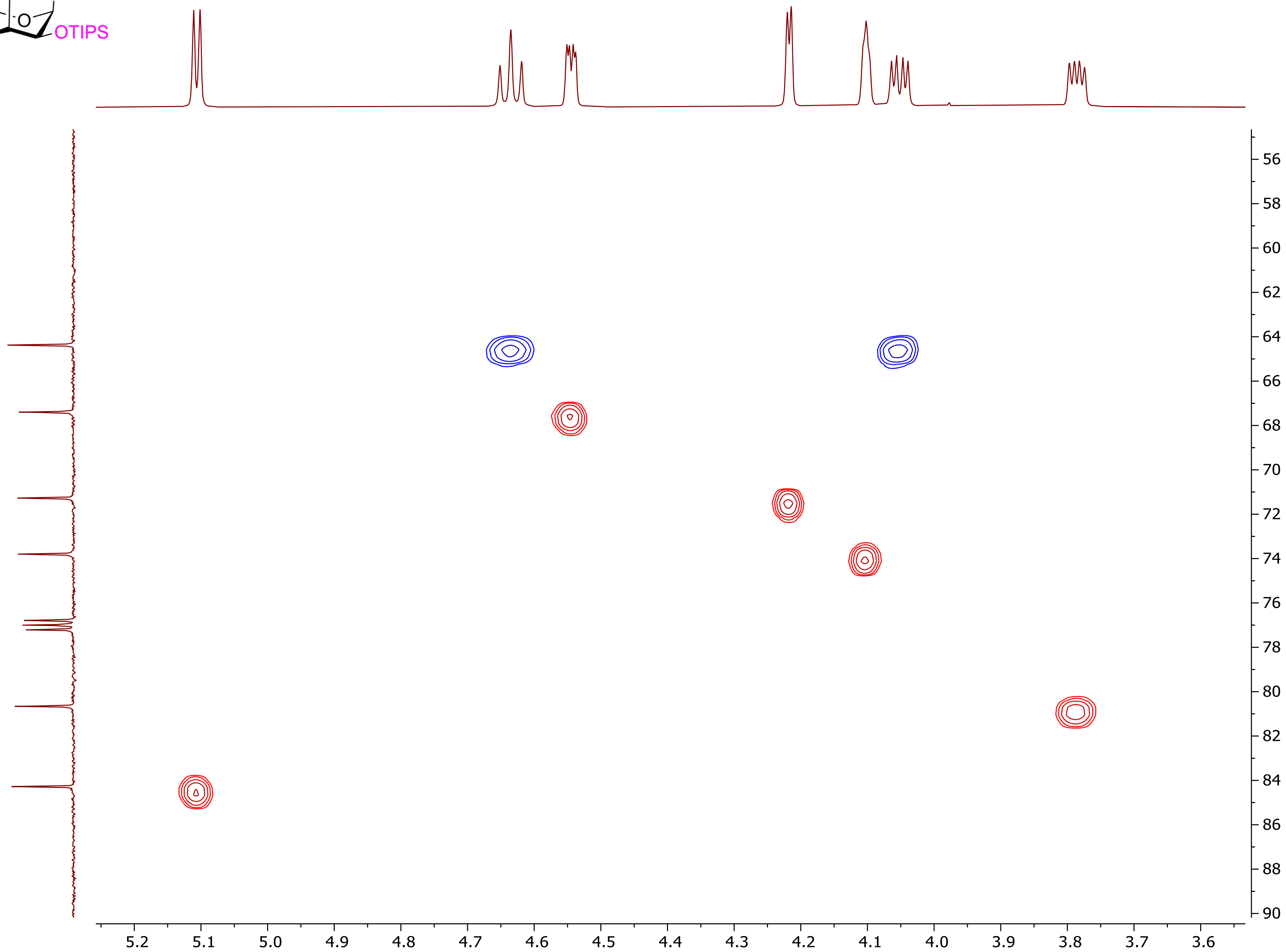

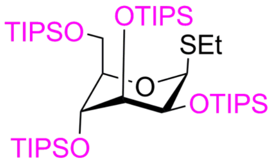

HMBC (600 MHz) spectrum of compound 13 in $CDCl_3$ (240K)

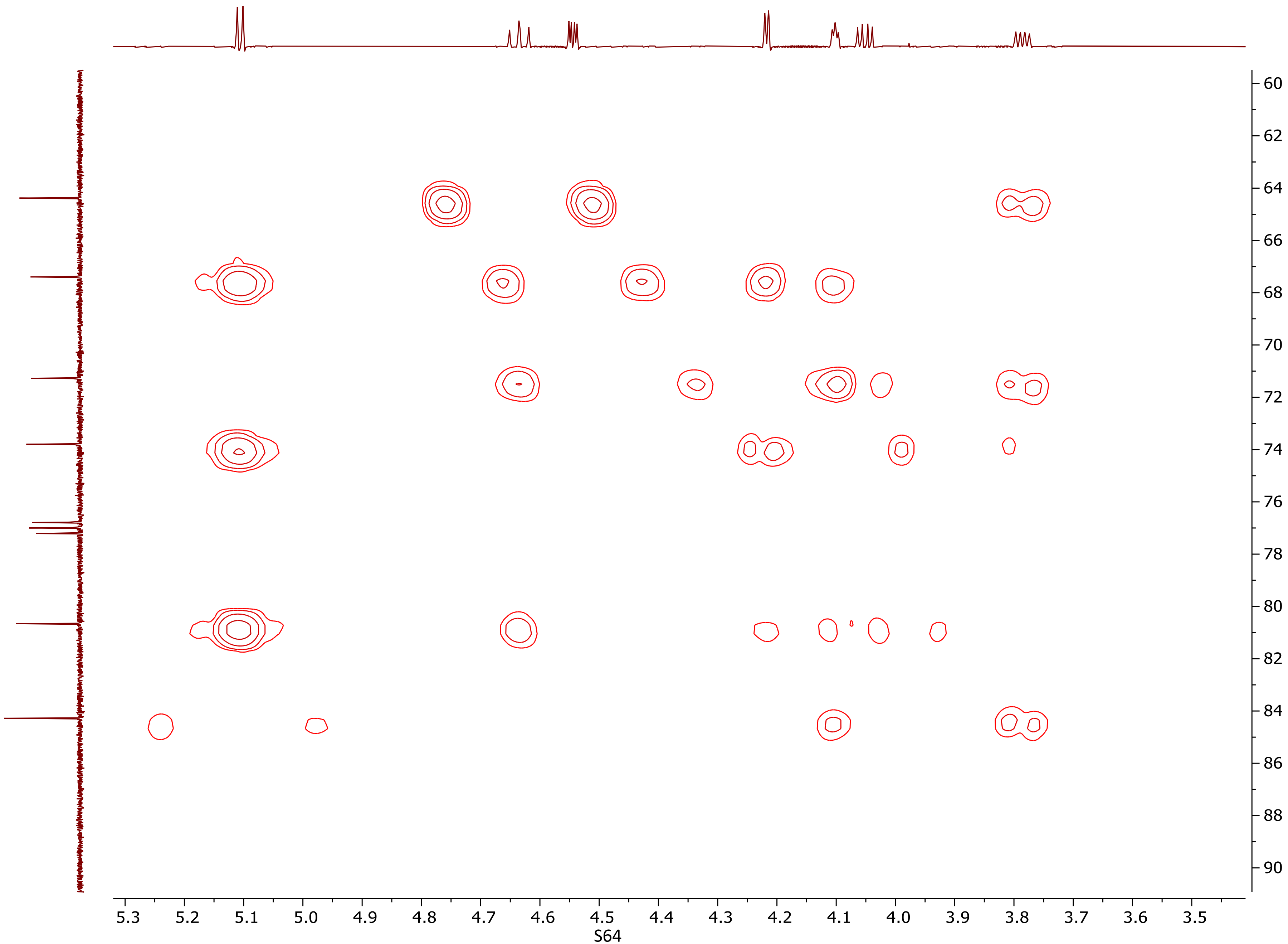

^{1}H NMR (600 MHz) spectrum of compound 15 in $CDCl_3$ (298K)

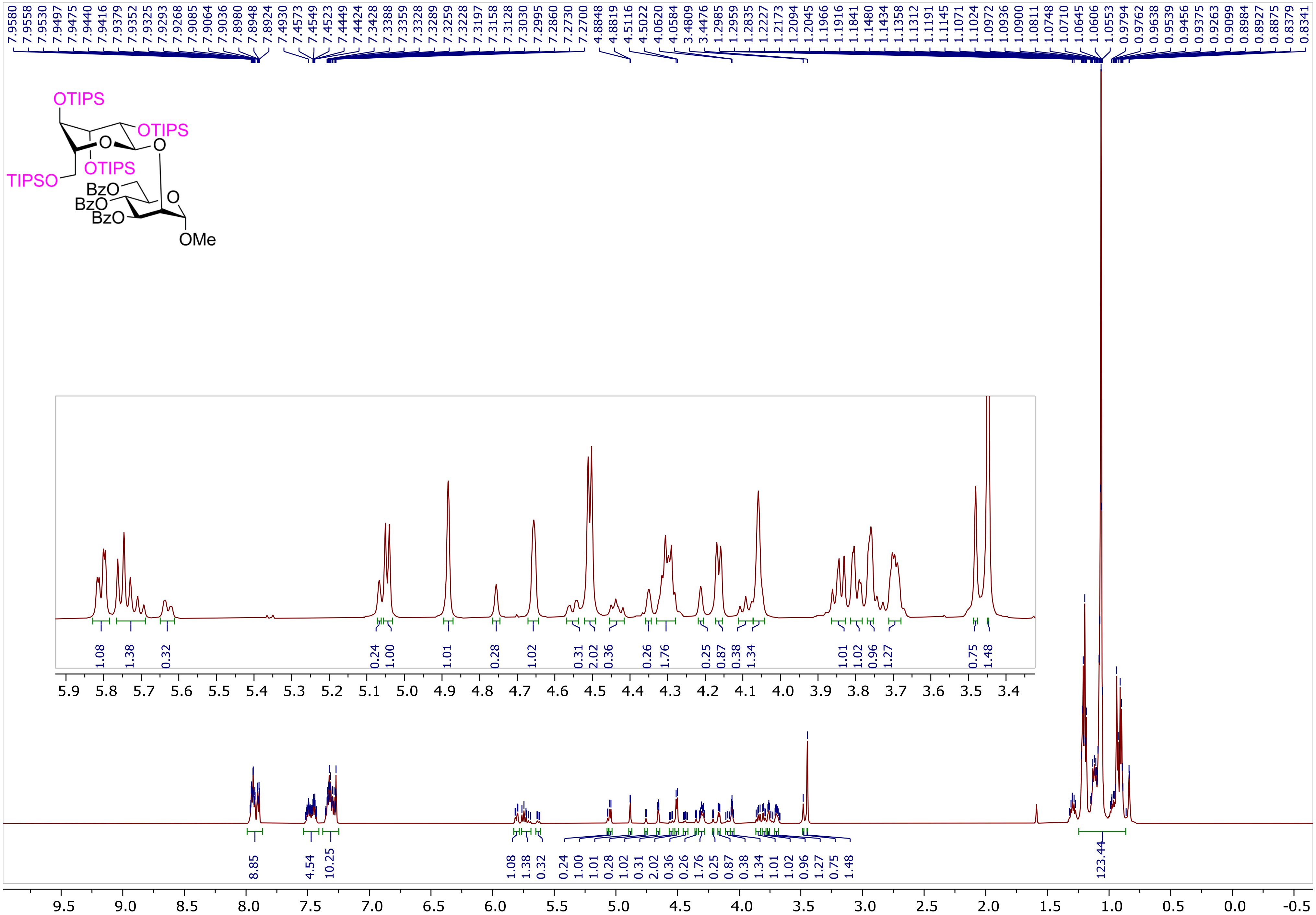

^{13}C NMR (151 MHz) spectrum of compound 15 in $CDCl_3$ (298K)

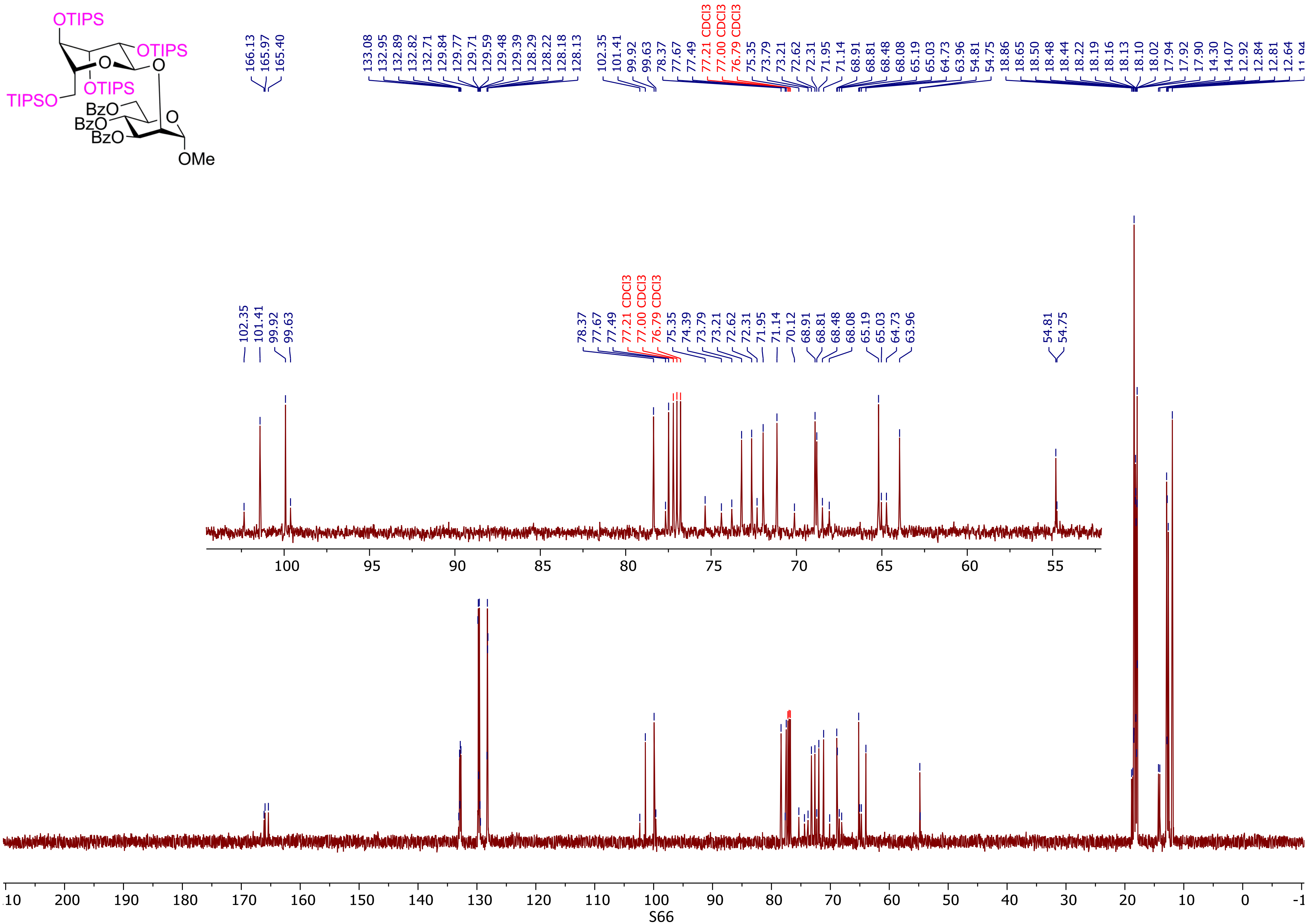

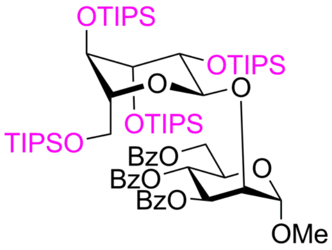

COSY (600 MHz) spectrum of compound 15 in $CDCl_3$ (298K)

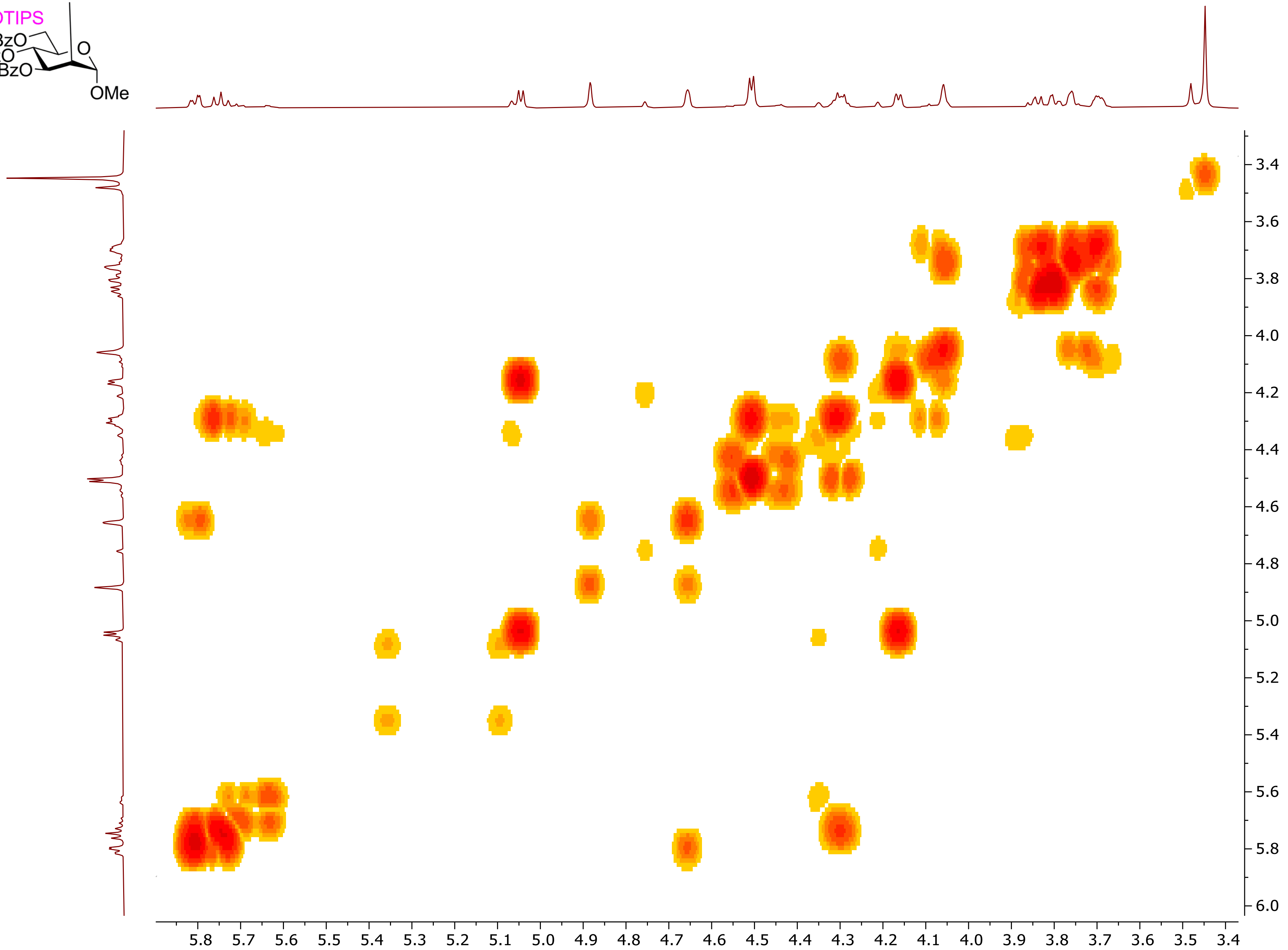

HSQC (600 MHz) spectrum of compound 15 in $CDCl_3$ (298K)

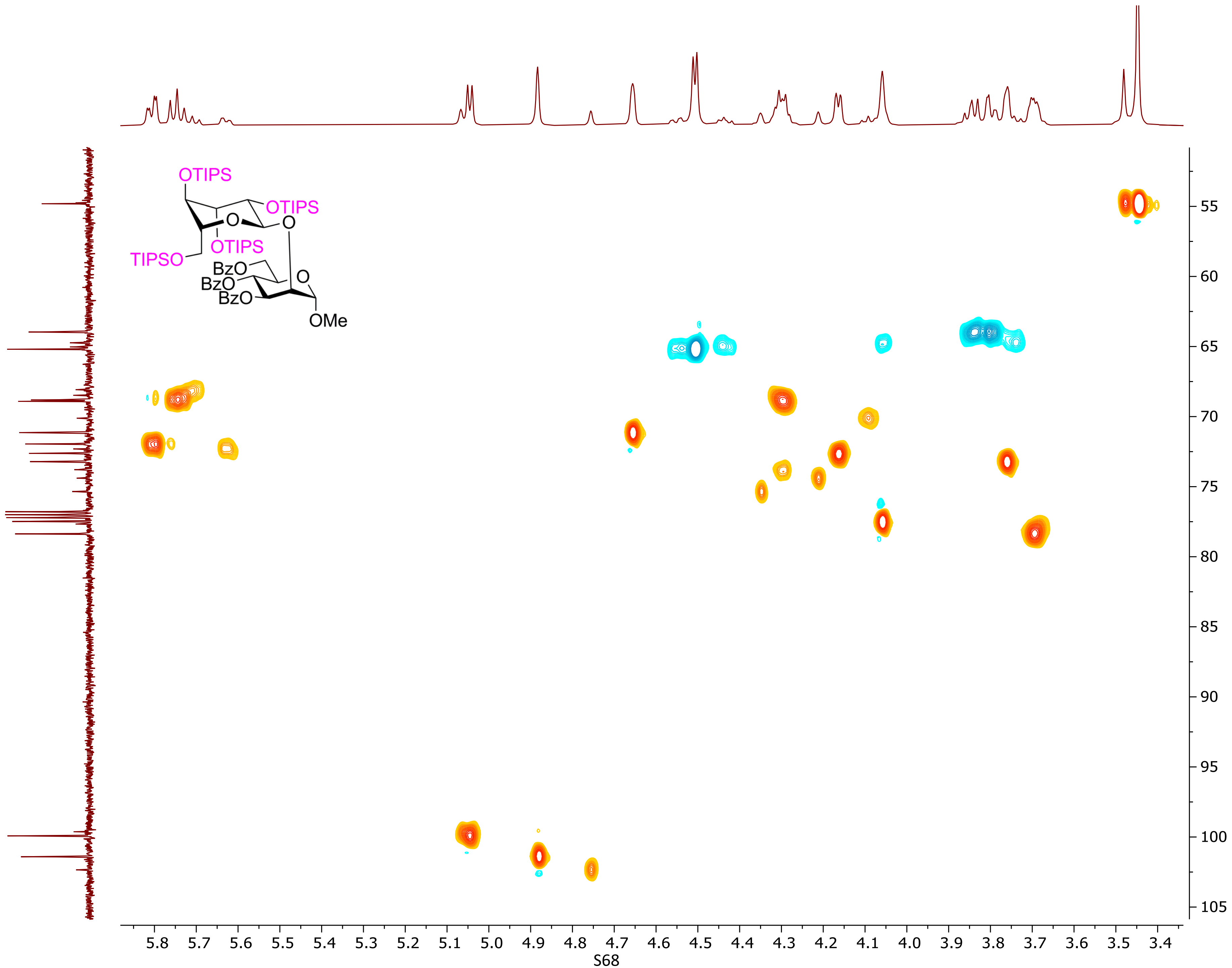

HMBC (600 MHz) spectrum of compound 15 in $CDCl_3$ (298K)

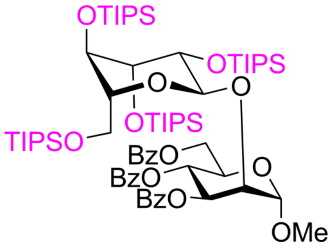

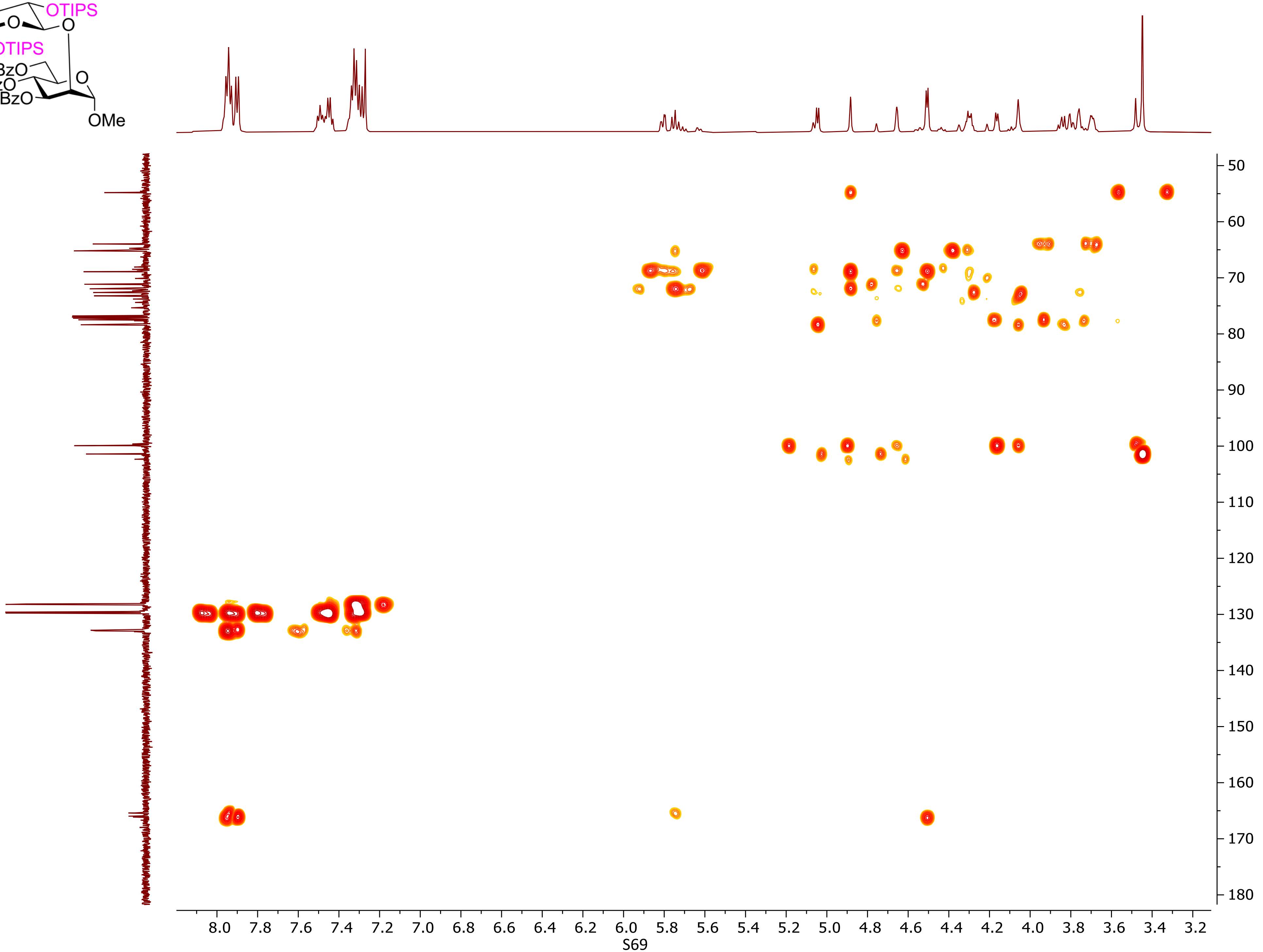

^{1}H–^{29}Si HMBC (600 MHz) spectrum of compound 15 in $CDCl_3$ (298K)

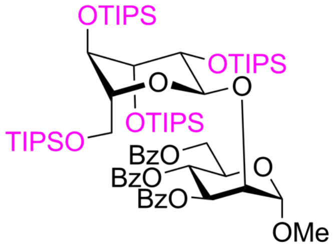

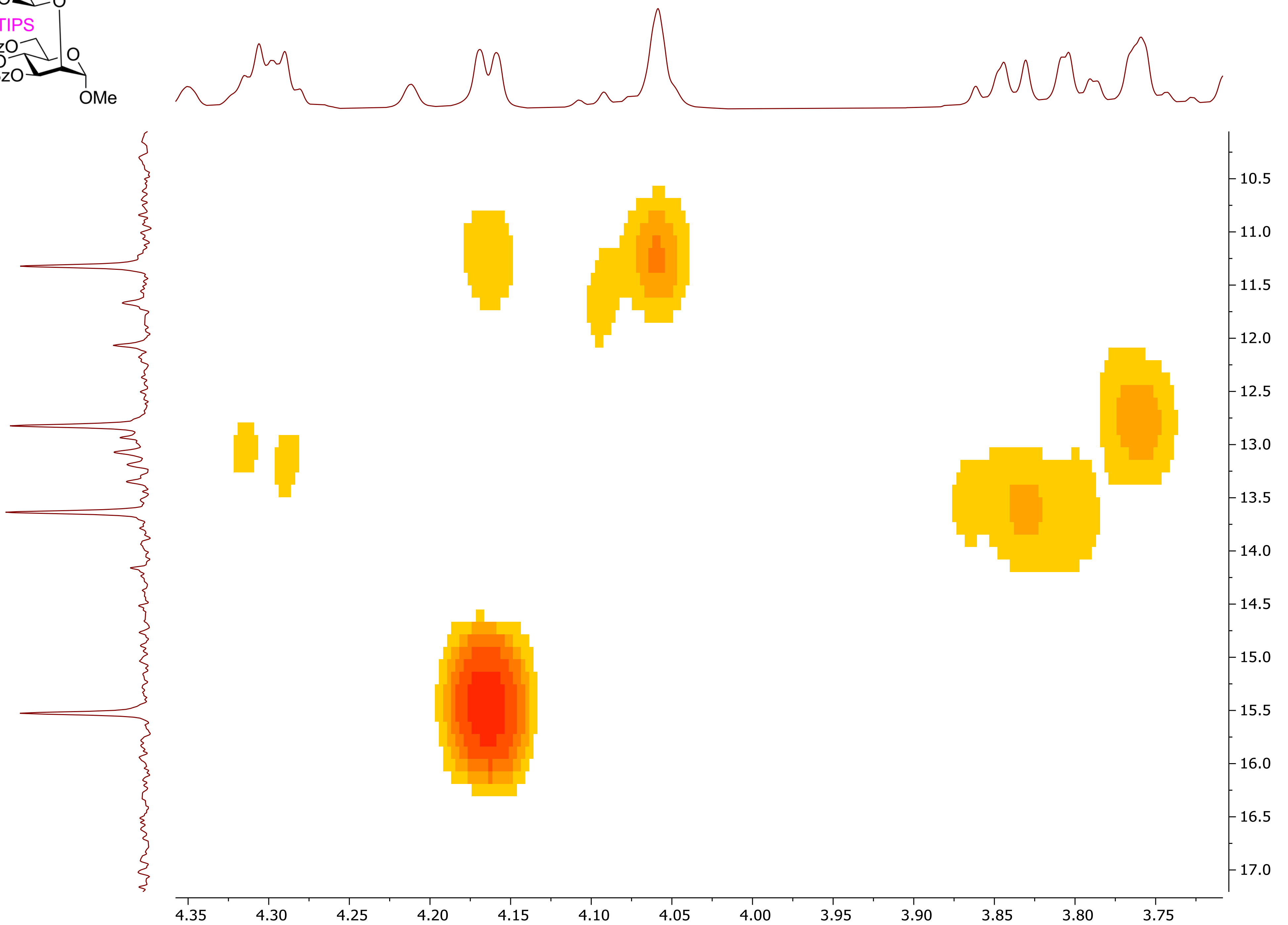

^{29}Si INEPT NMR (60 MHz) spectrum of compound 15 in $CDCl_3$ (298K)

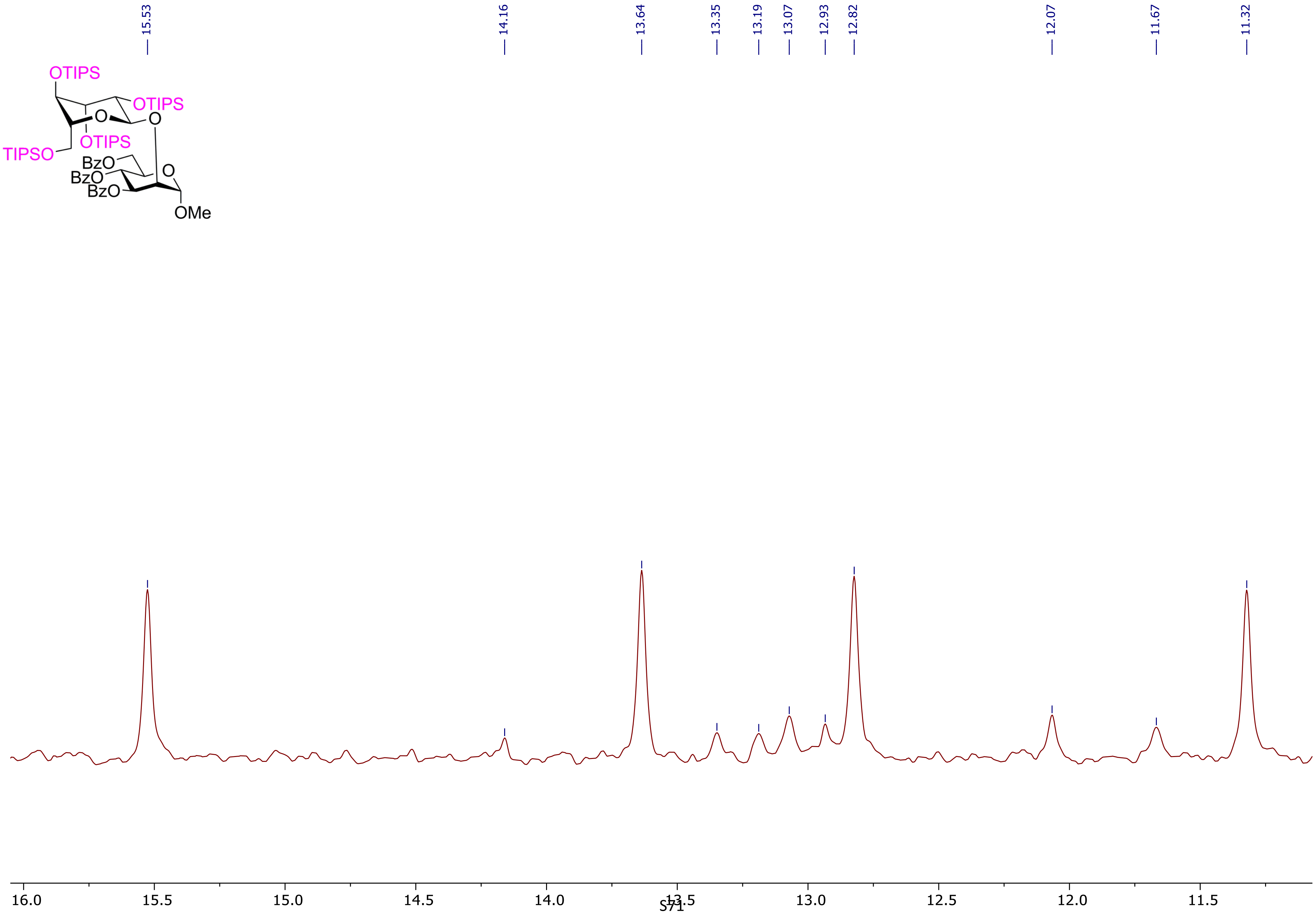

^{1}H NMR (600 MHz) spectrum of compound 15 in $CDCl_3$ (240K)

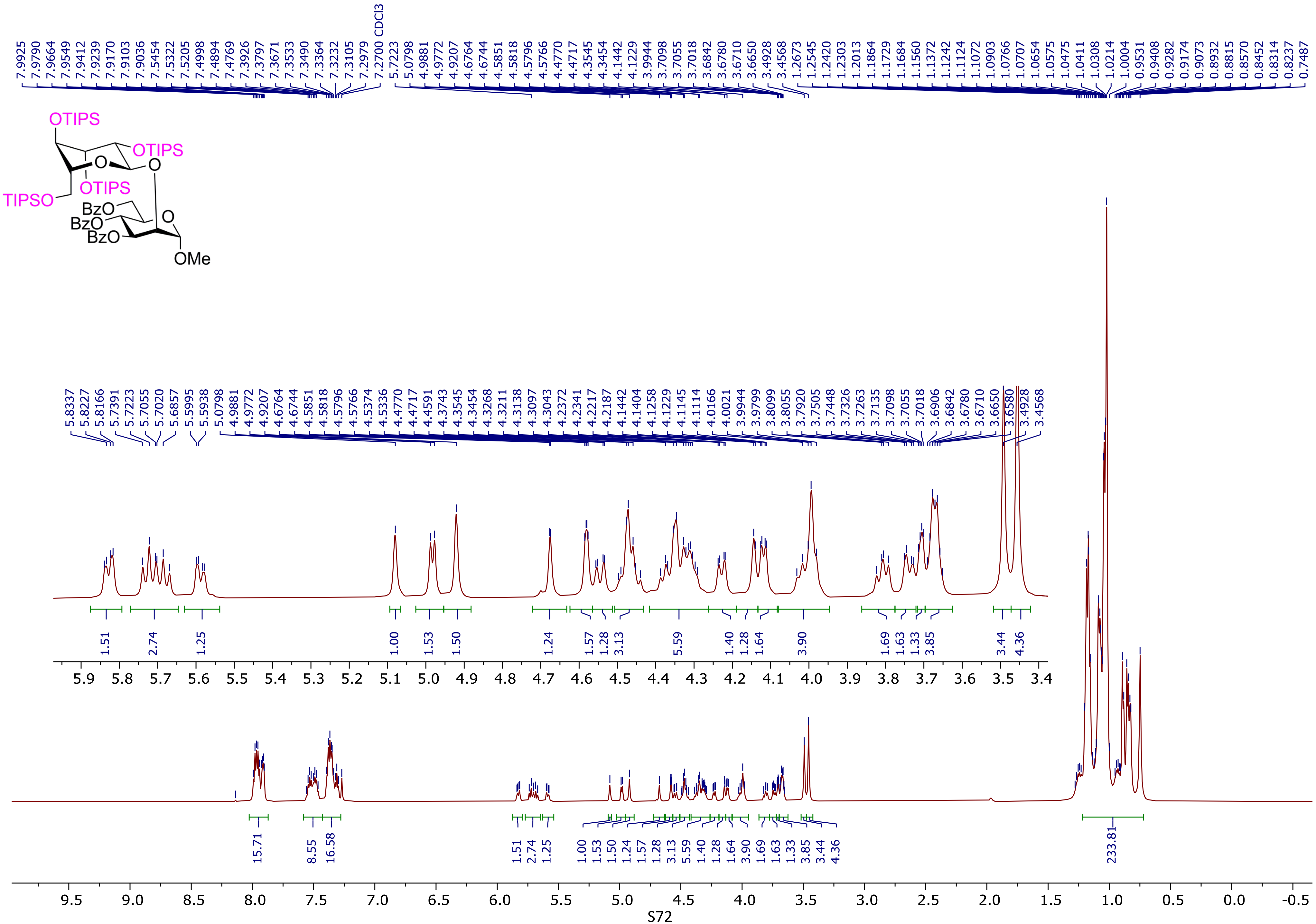

^{13}C NMR (151 MHz) spectrum of compound 15 in $CDCl_3$ (240K)

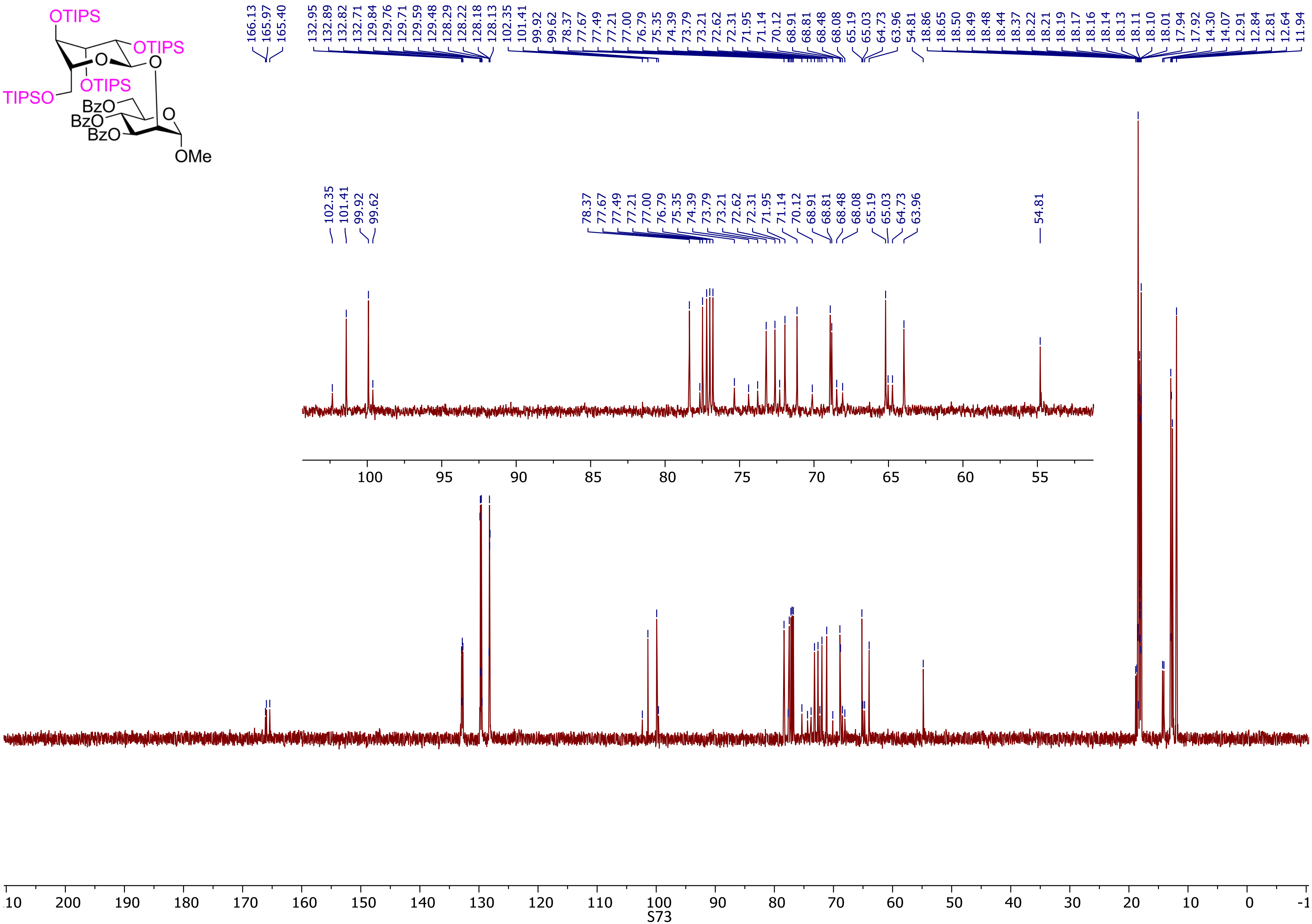

COSY (600 MHz) spectrum of compound 15 in $CDCl_3$ (240K)

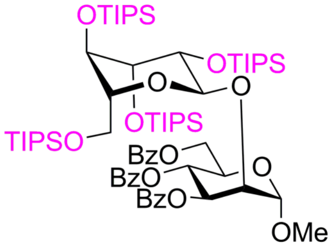

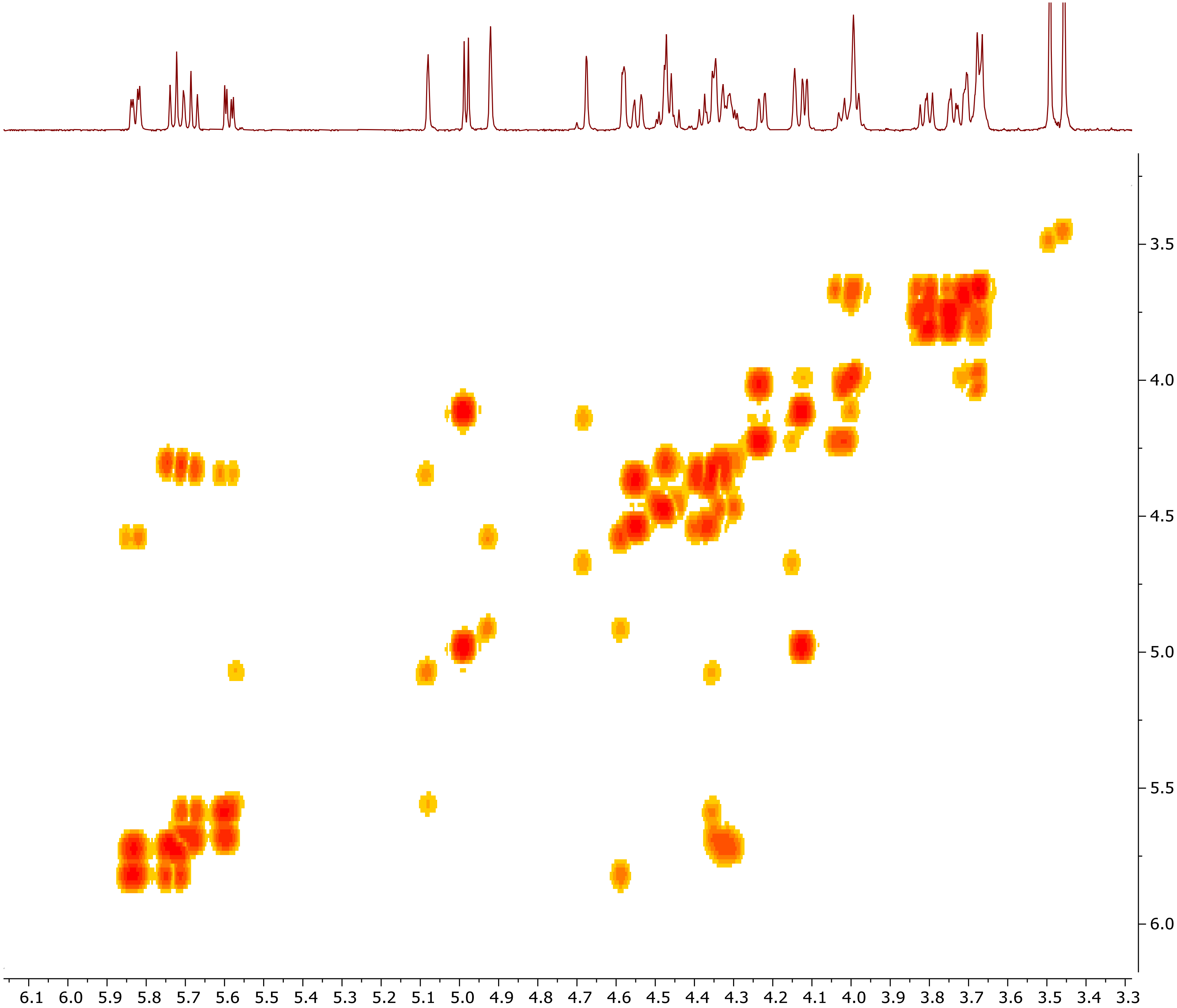

HSQC (600 MHz) spectrum of compound 15 in $CDCl_3$ (240K)

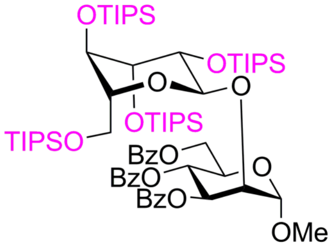

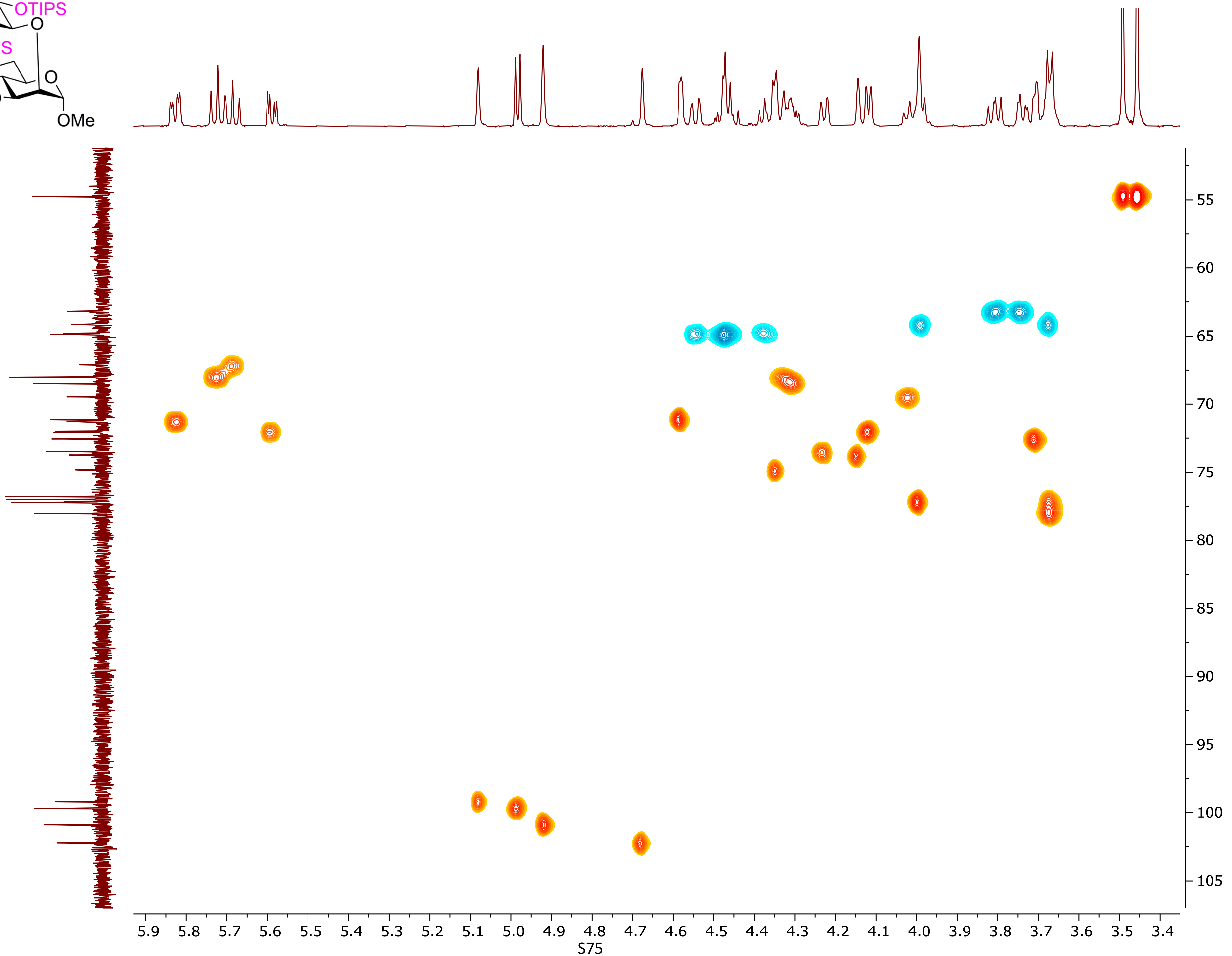

HMBC (600 MHz) spectrum of compound of compound 15 in $CDCl_3$ (240K)

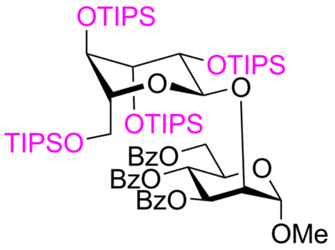

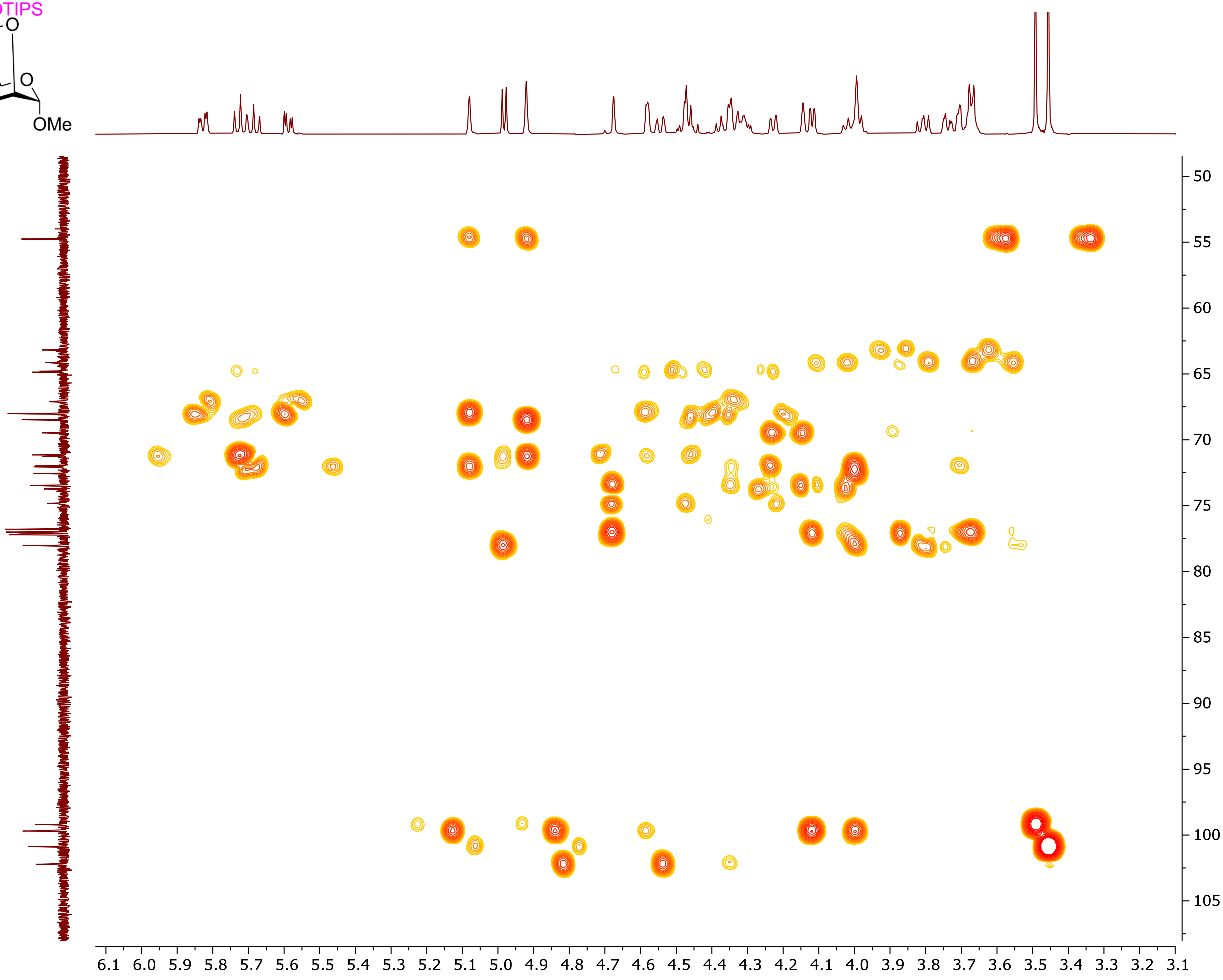

^{1}H–^{29}Si HMBC (600 MHz) spectrum of compound 15 in $CDCl_3$ (240K)

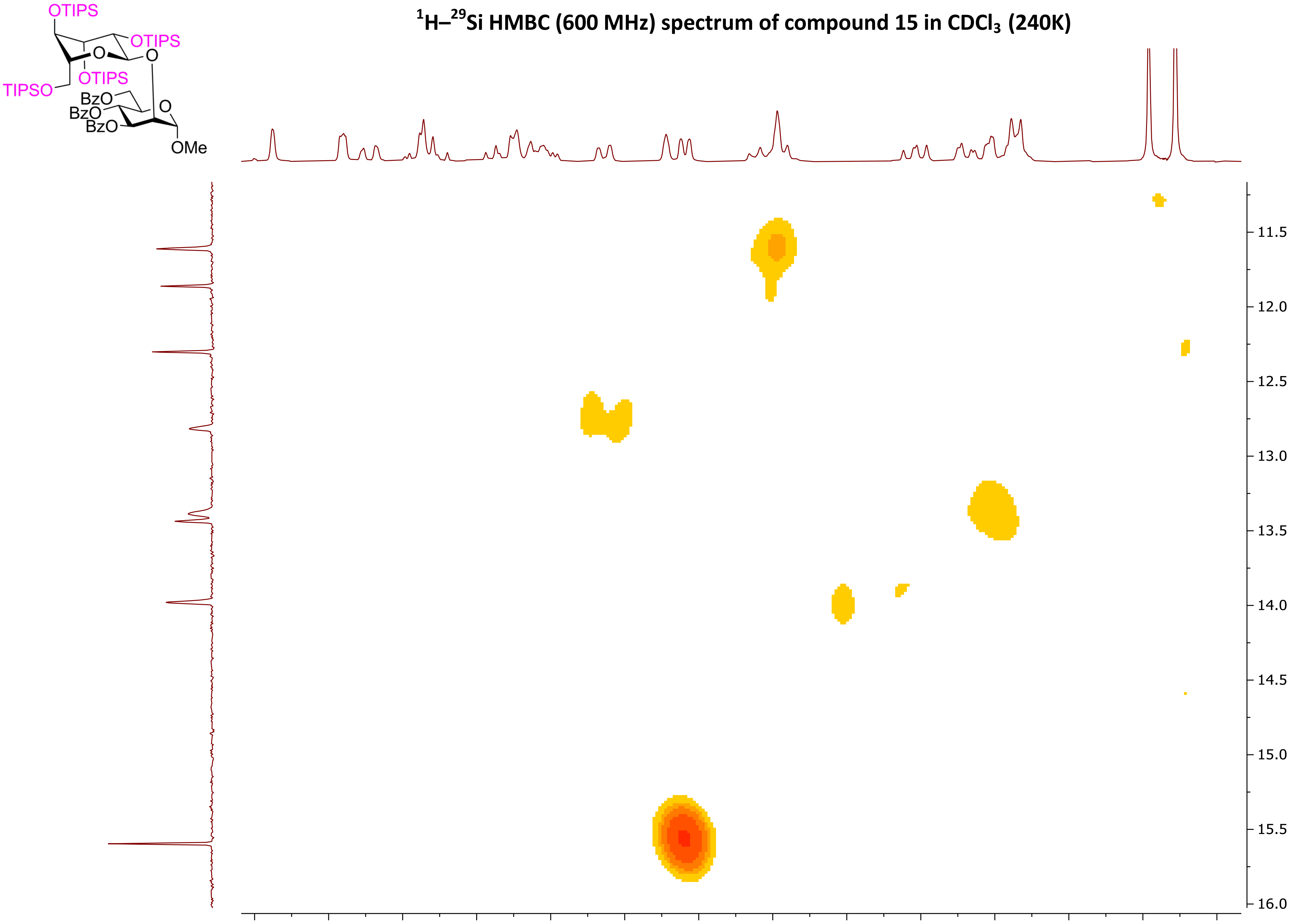

^{29}Si INEPT NMR (119 MHz) spectrum of compound 15 in $CDCl_3$ (240K)

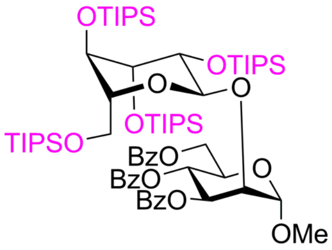

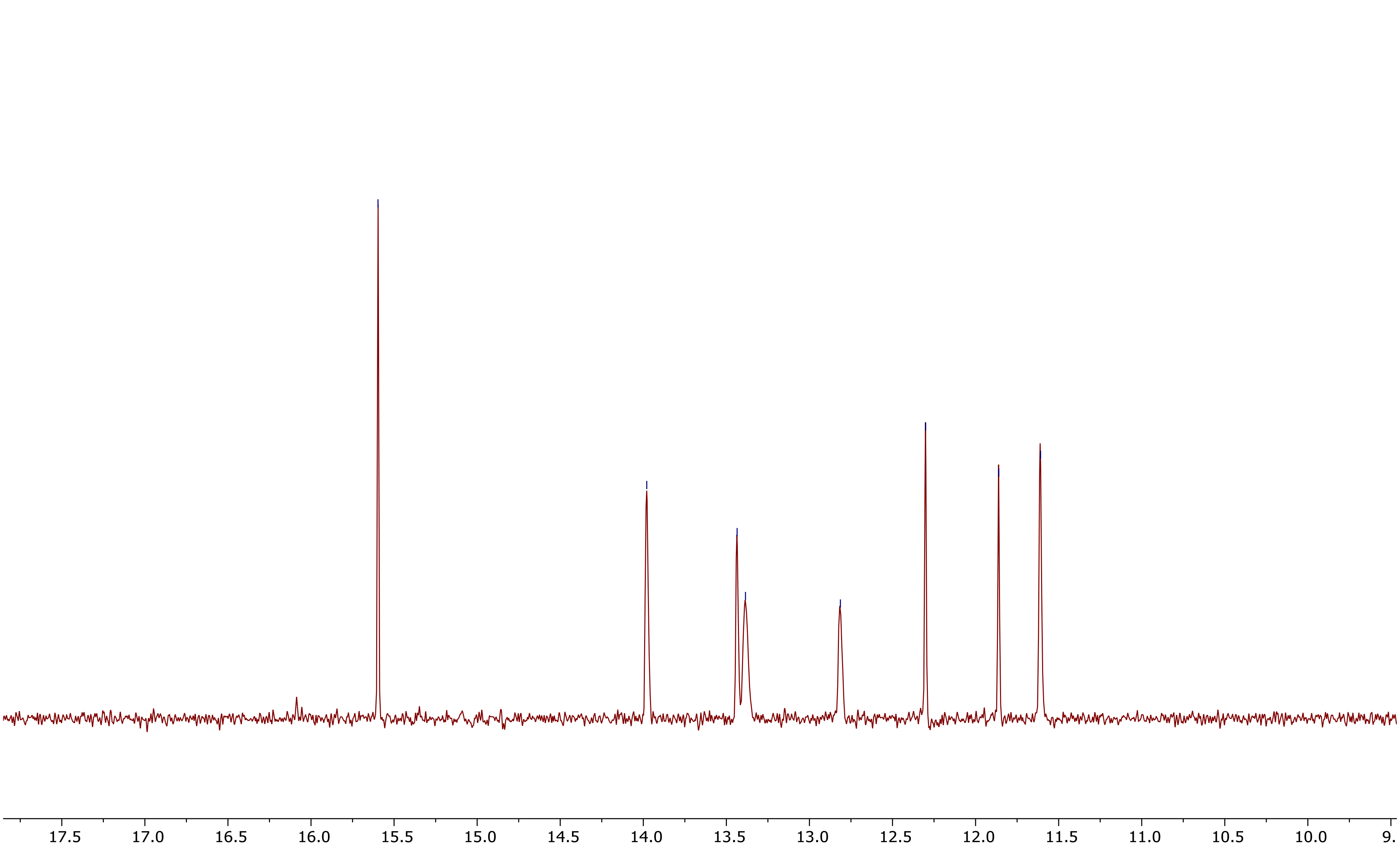

^{1}H NMR (600 MHz) spectrum of compound 16 in $CDCl_3$

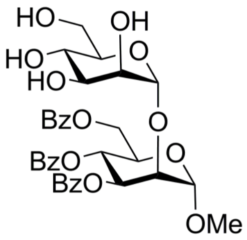

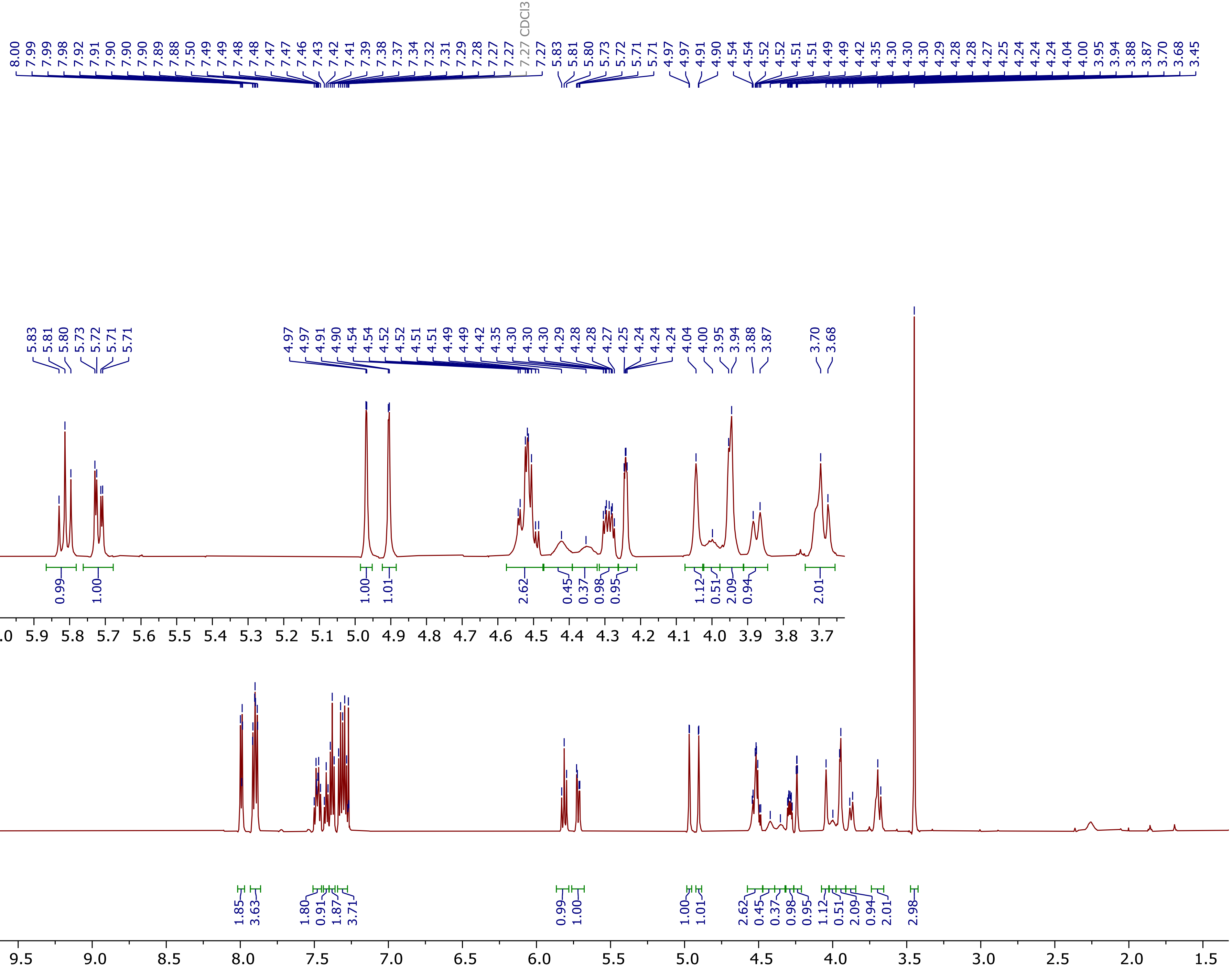

^{13}C NMR (151 MHz) spectrum of compound 16 in $CDCl_3$

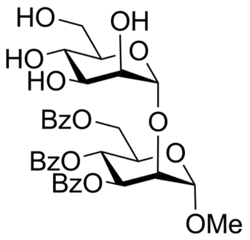

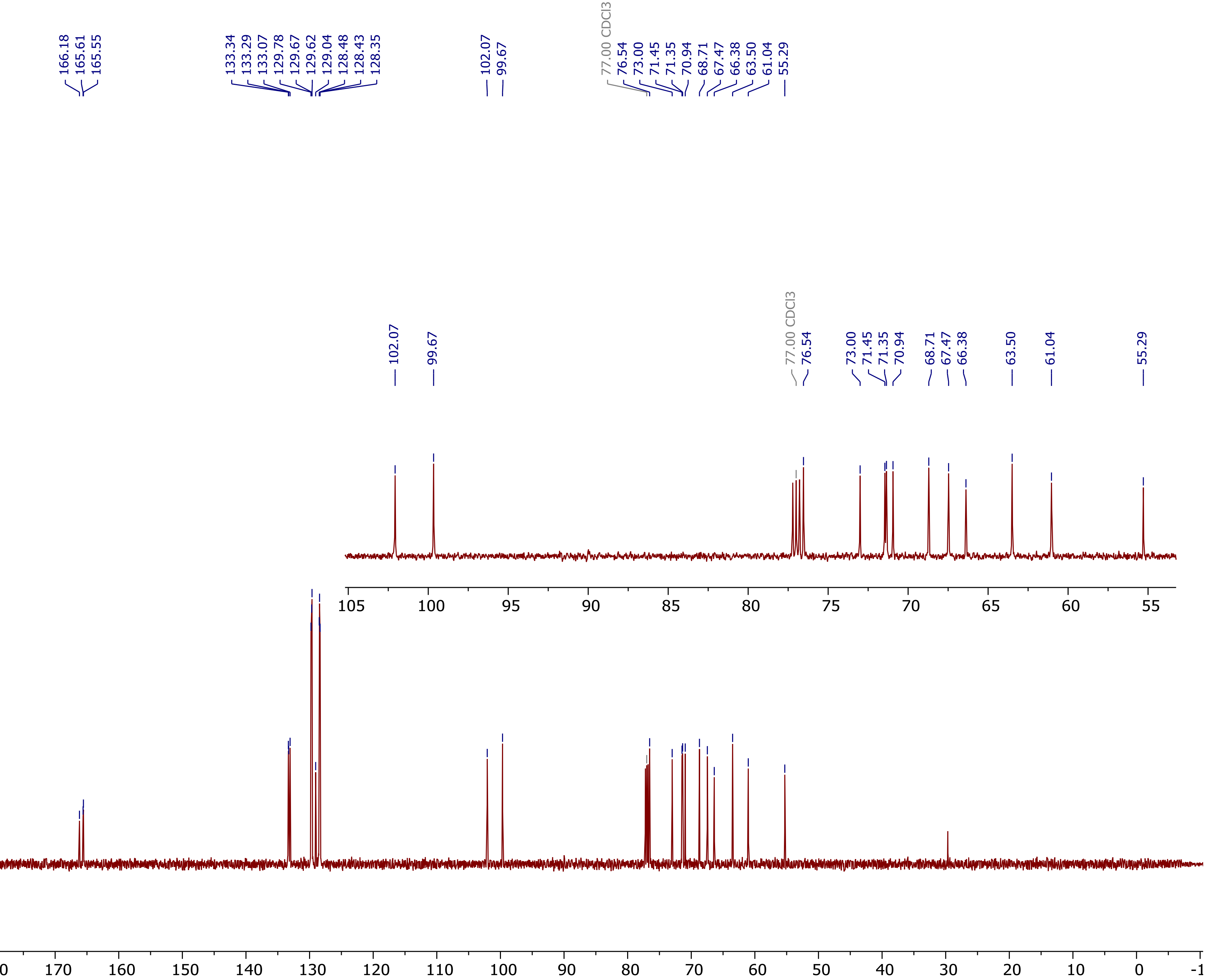

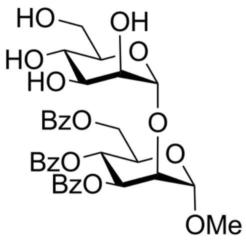

COSY (600 MHz) spectrum of compound 16 in $CDCl_3$

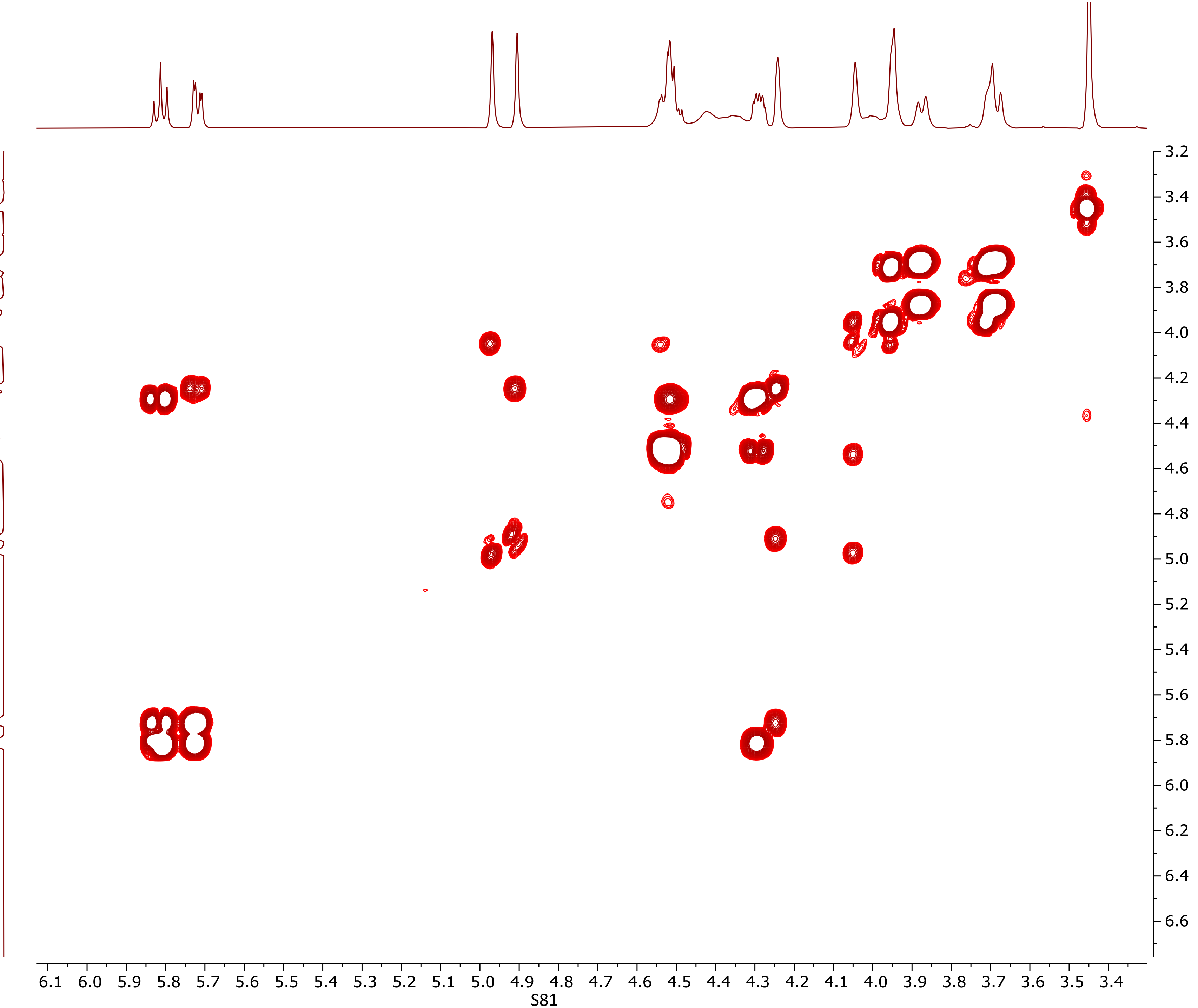

HSQC (600 MHz) spectrum of compound 16 in $CDCl_3$

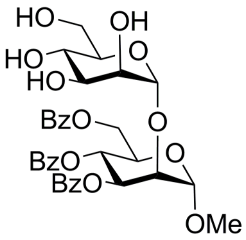

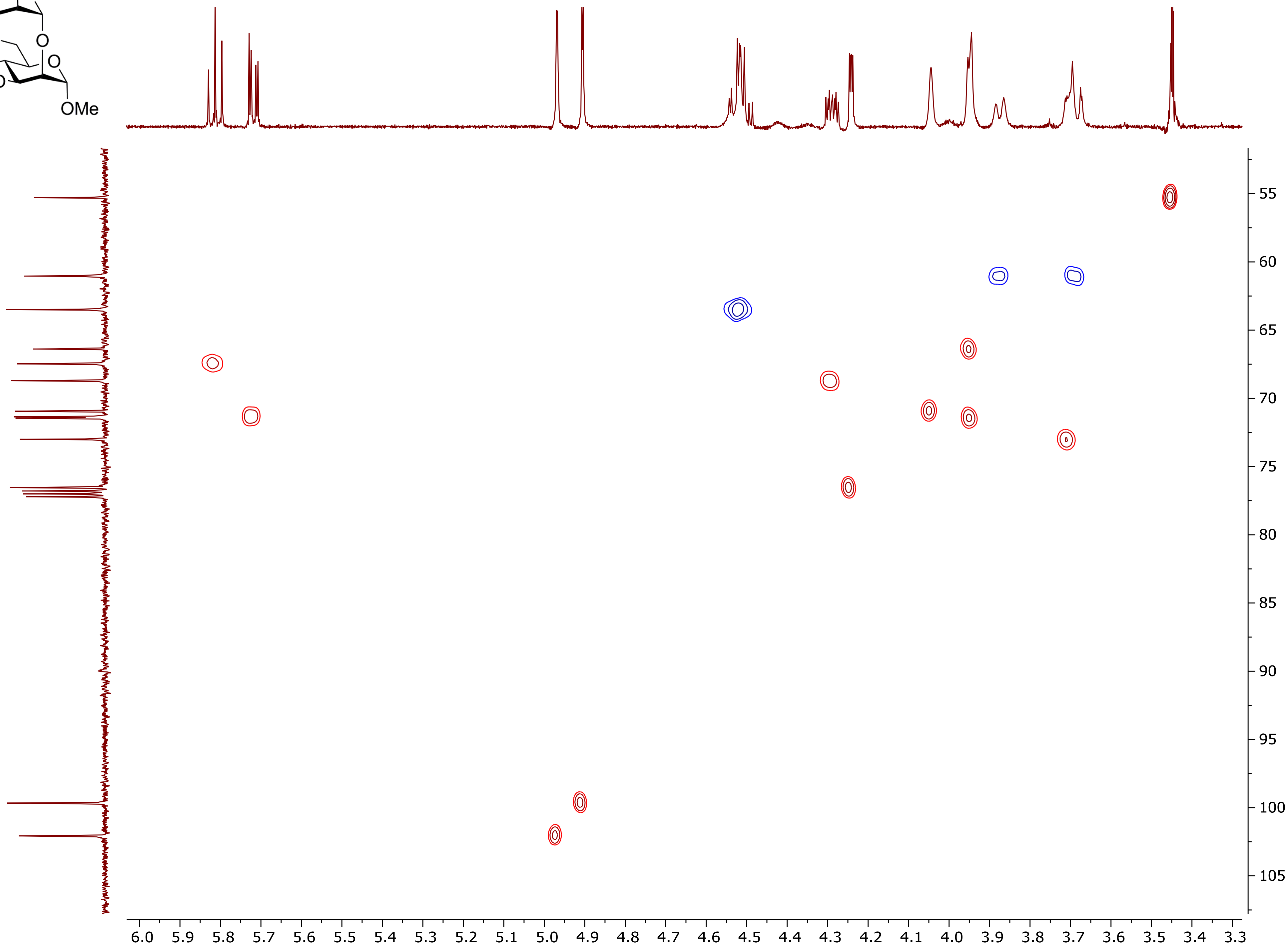

HMBC (600 MHz) spectrum of compound 16 in $CDCl_3$

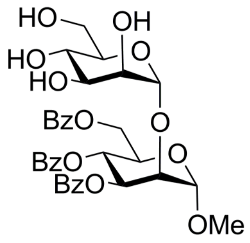

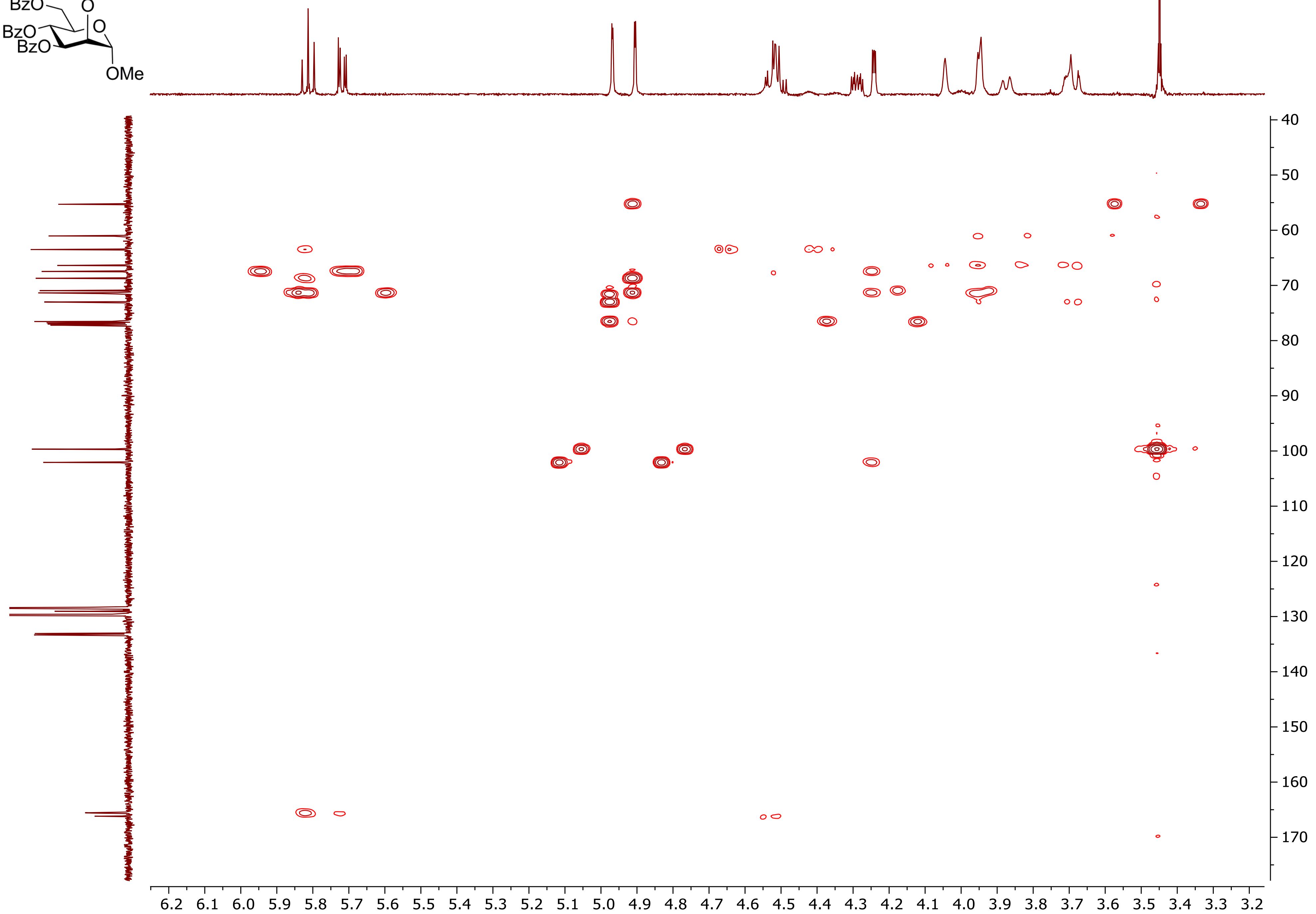

^{1}H NMR (600 MHz) spectrum of compound 18 in $CDCl_3$ (303K)

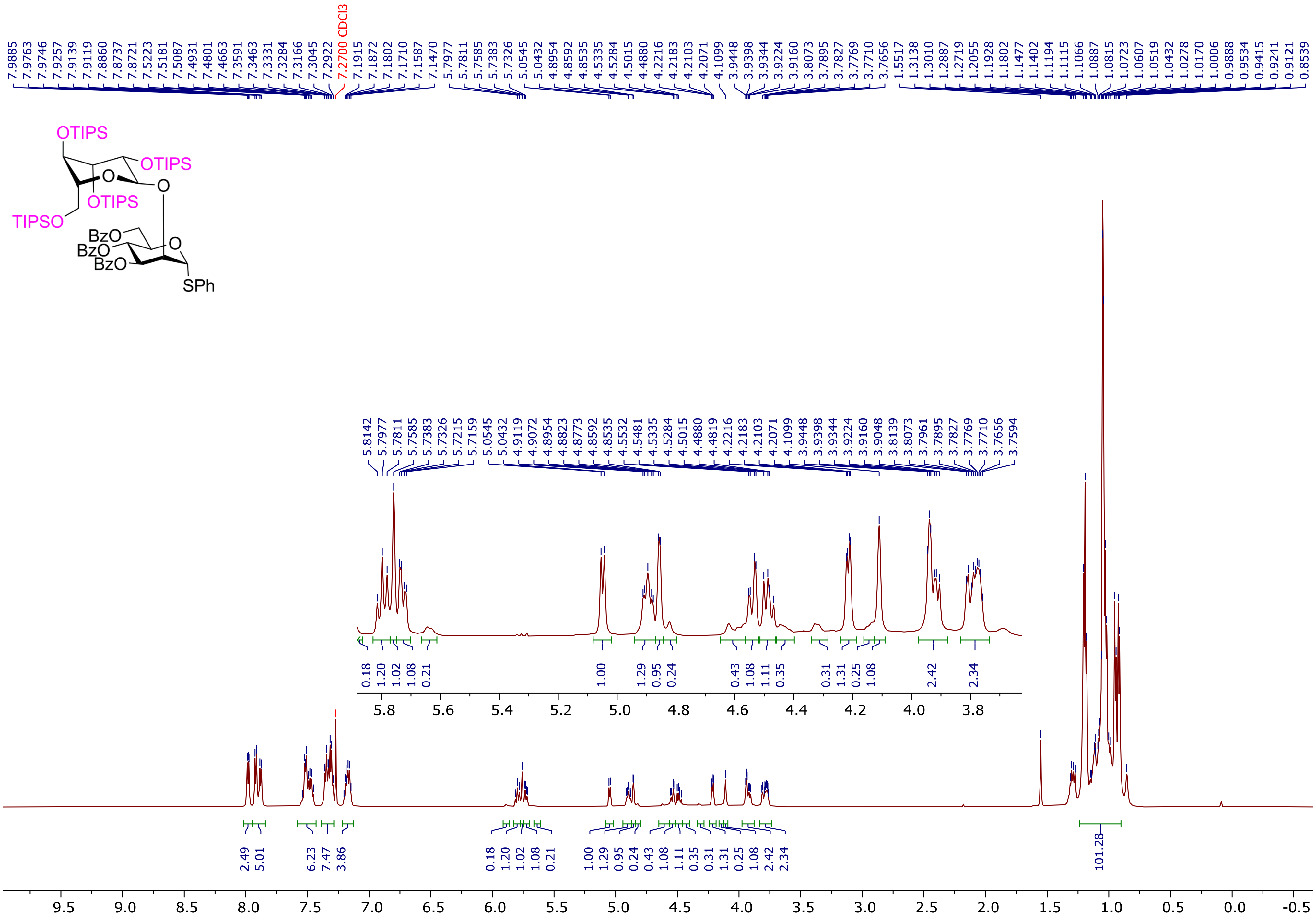

^{13}C NMR (151 MHz) spectrum of compound 18 in $CDCl_3$ (303K)

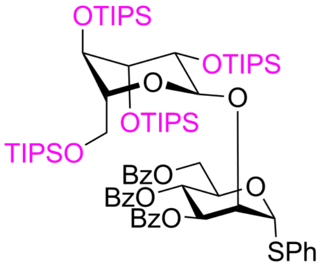

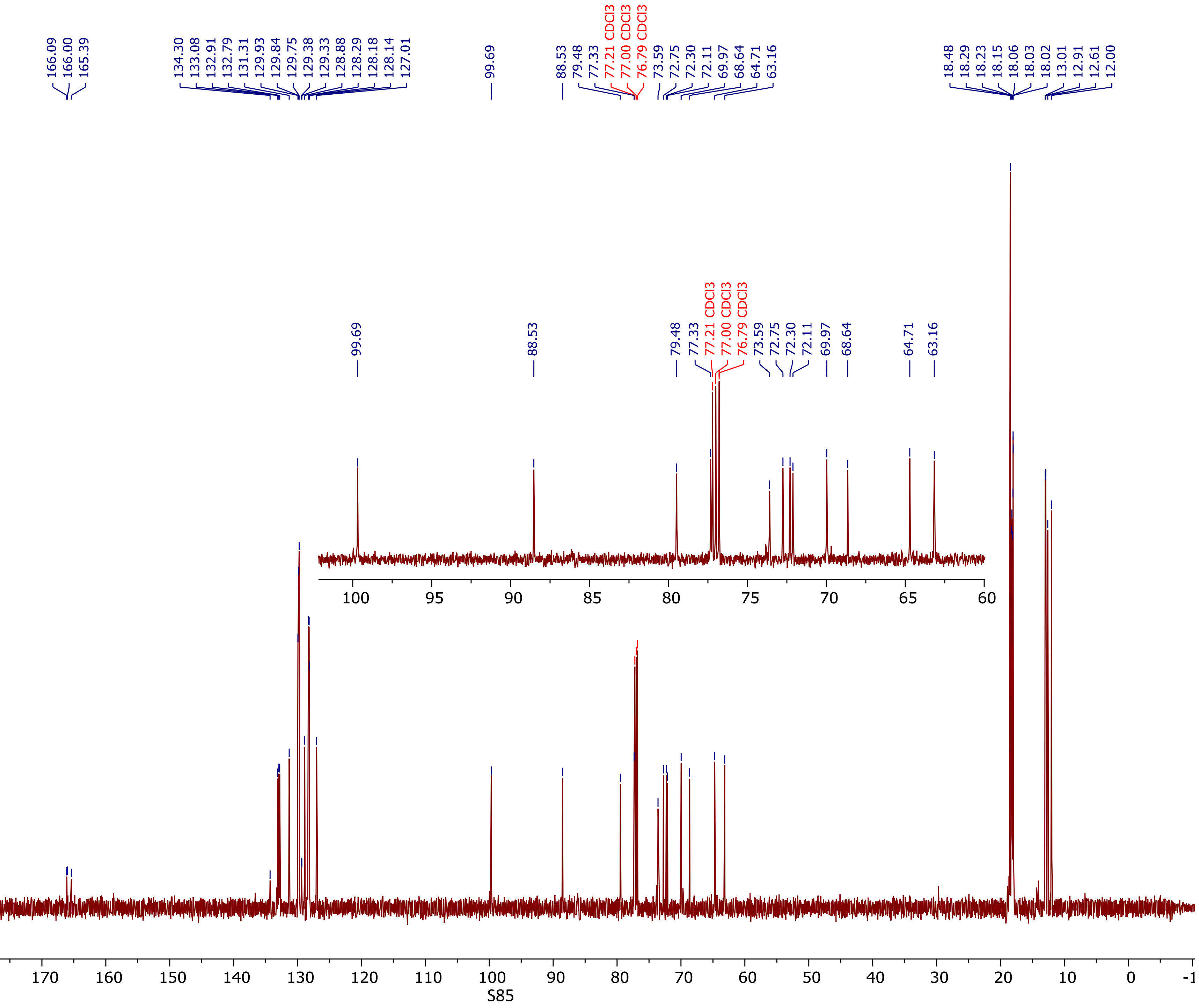

COSY (600 MHz) spectrum of compound 18 in $CDCl_3$ (303K)

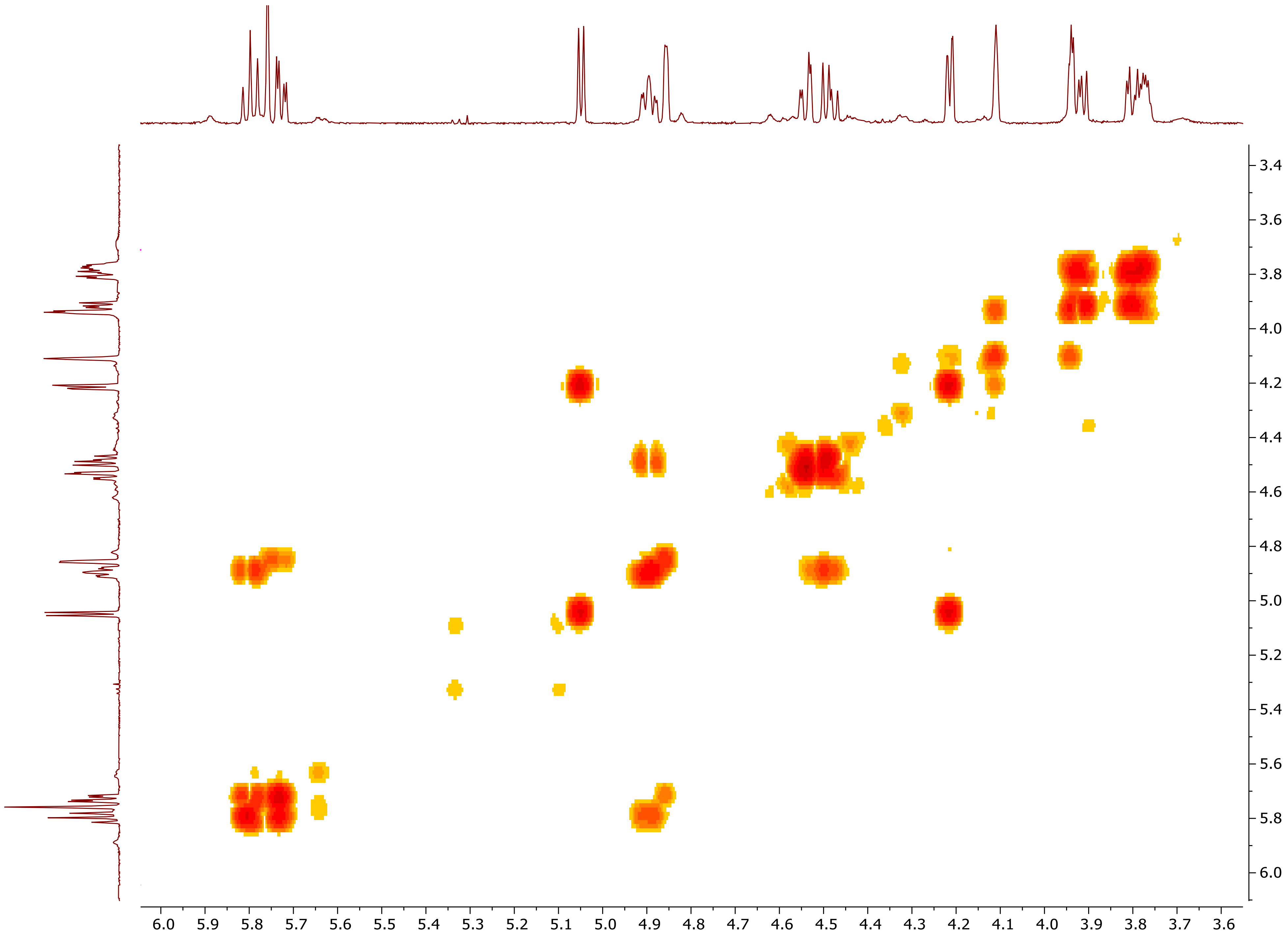

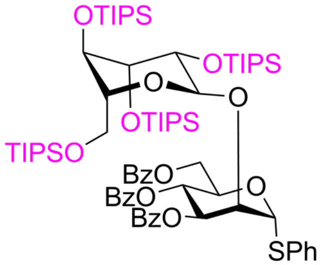

HSQC (600 MHz) spectrum of compound 18 in $CDCl_3$ (303K)

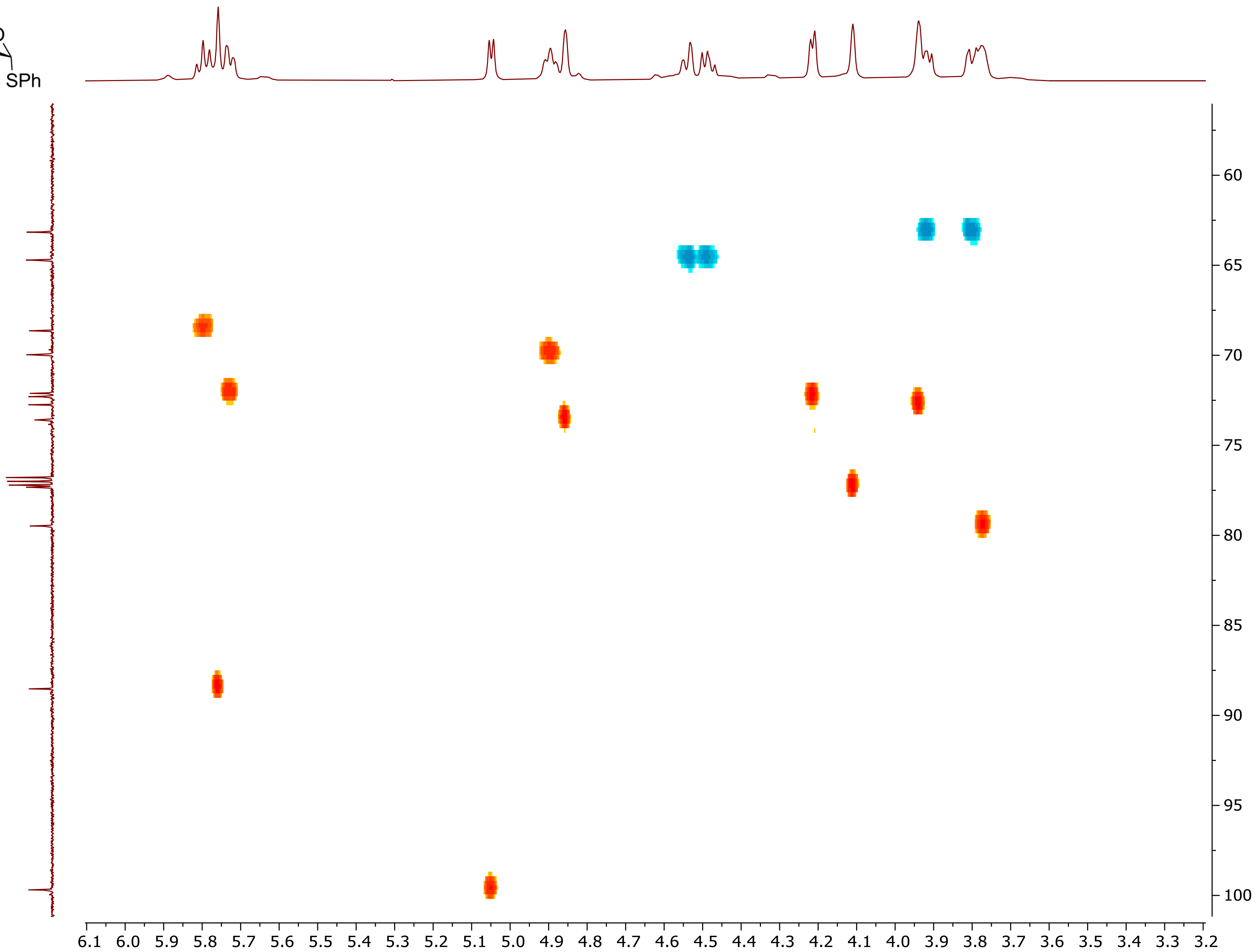

HMBC (600 MHz) spectrum of compound 18 in $CDCl_3$ (303K)

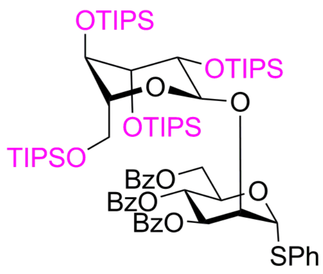

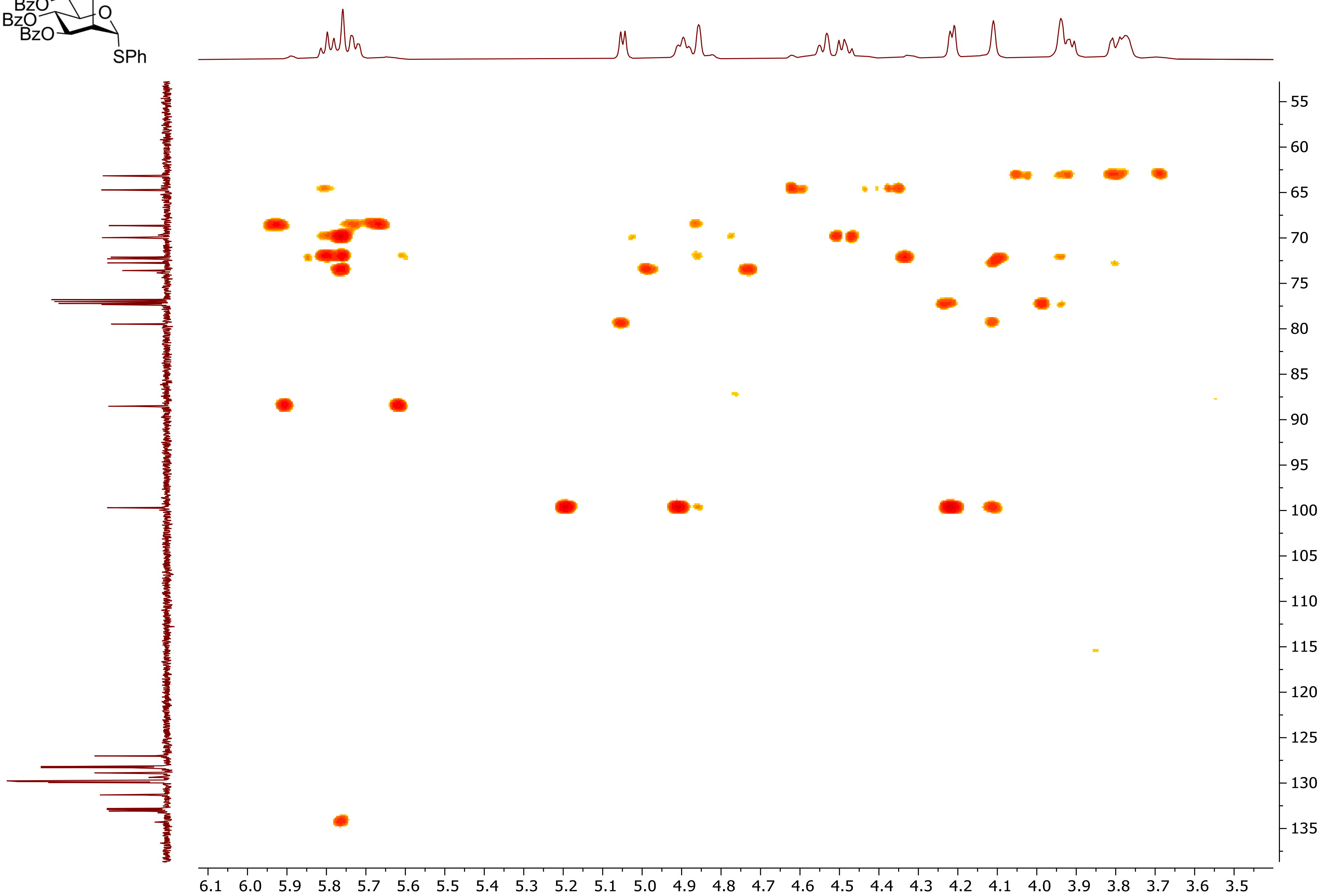

^{29}Si INEPT NMR (119 MHz) spectrum of compound 18 in $CDCl_3$ (303K)

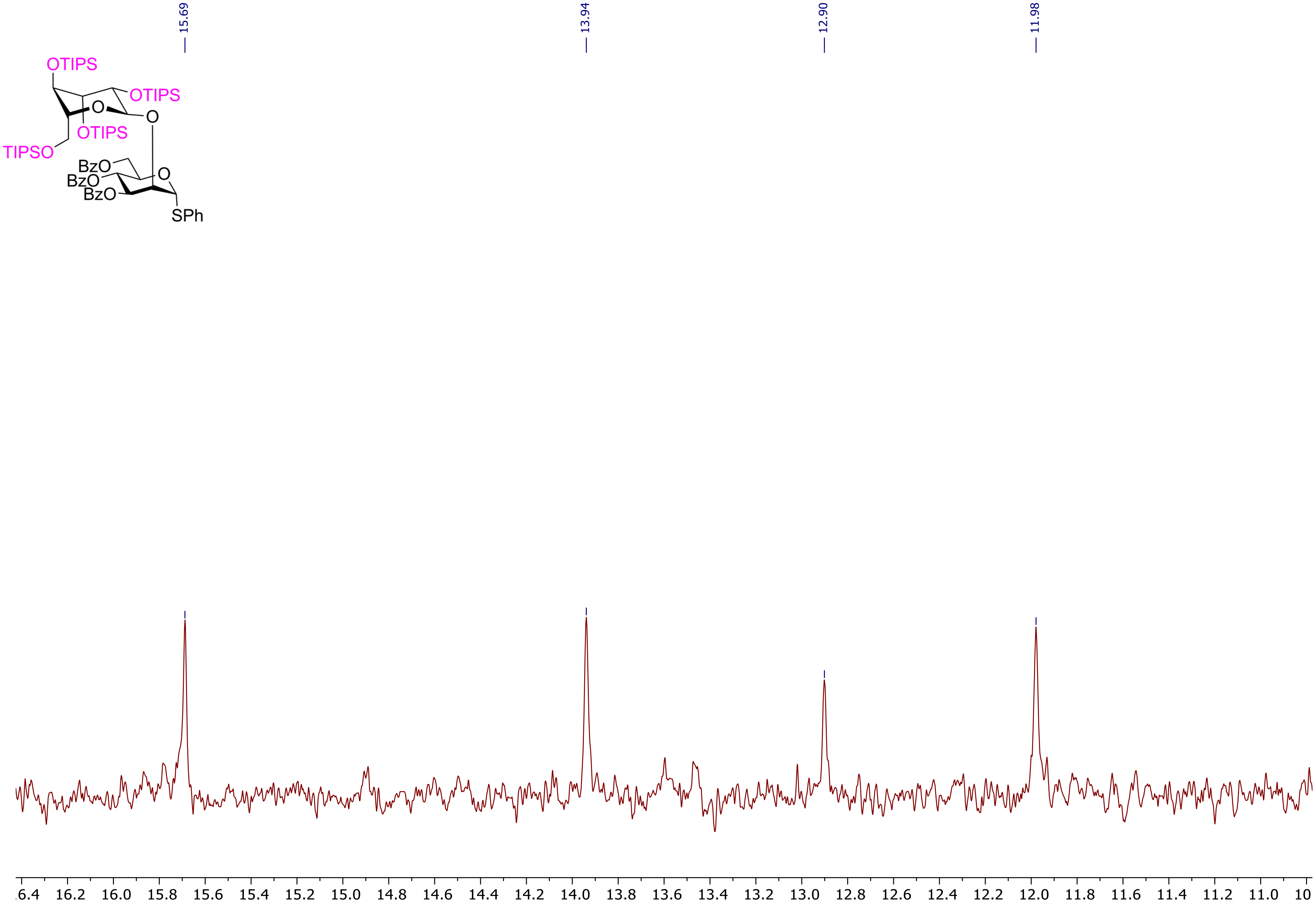

^{1}H NMR (600 MHz) spectrum of compound 18 in $CDCl_3$ (240K)

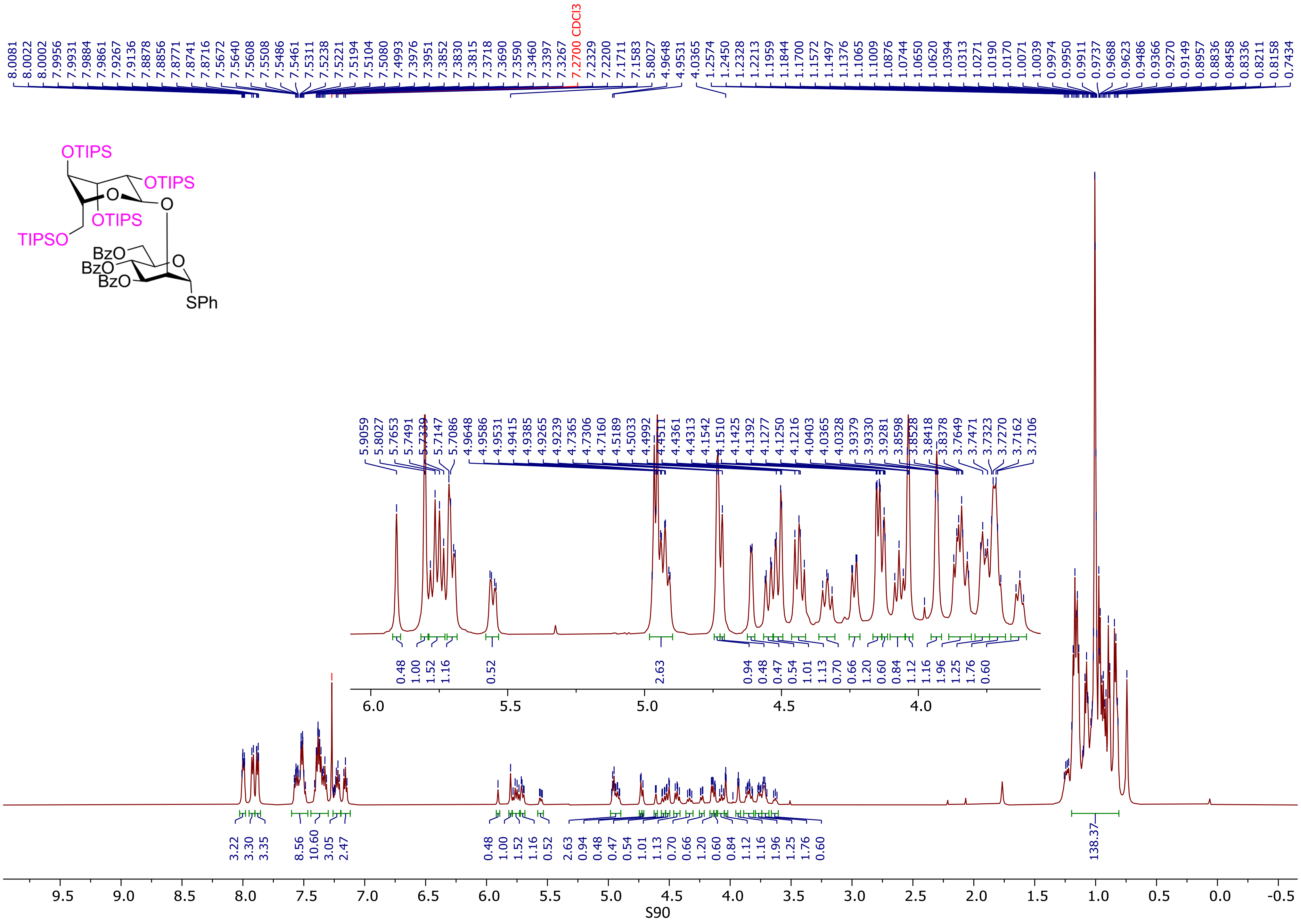

^{13}C NMR (151 MHz) spectrum of compound 18 in $CDCl_3$ (240K)

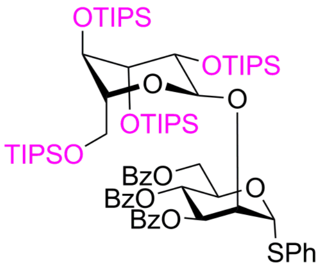

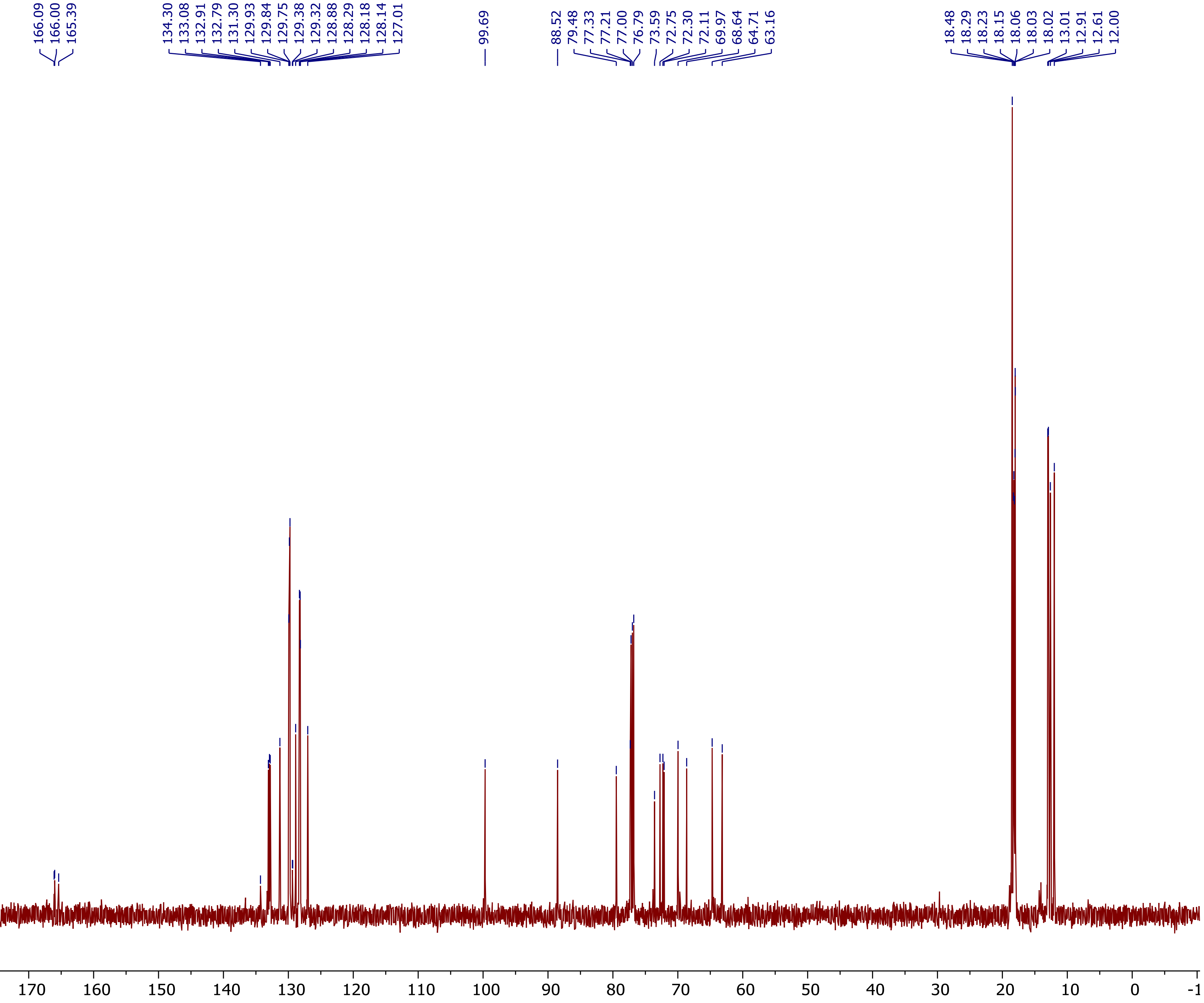

COSY (600 MHz) spectrum of compound 18 in $CDCl_3$ (240K)

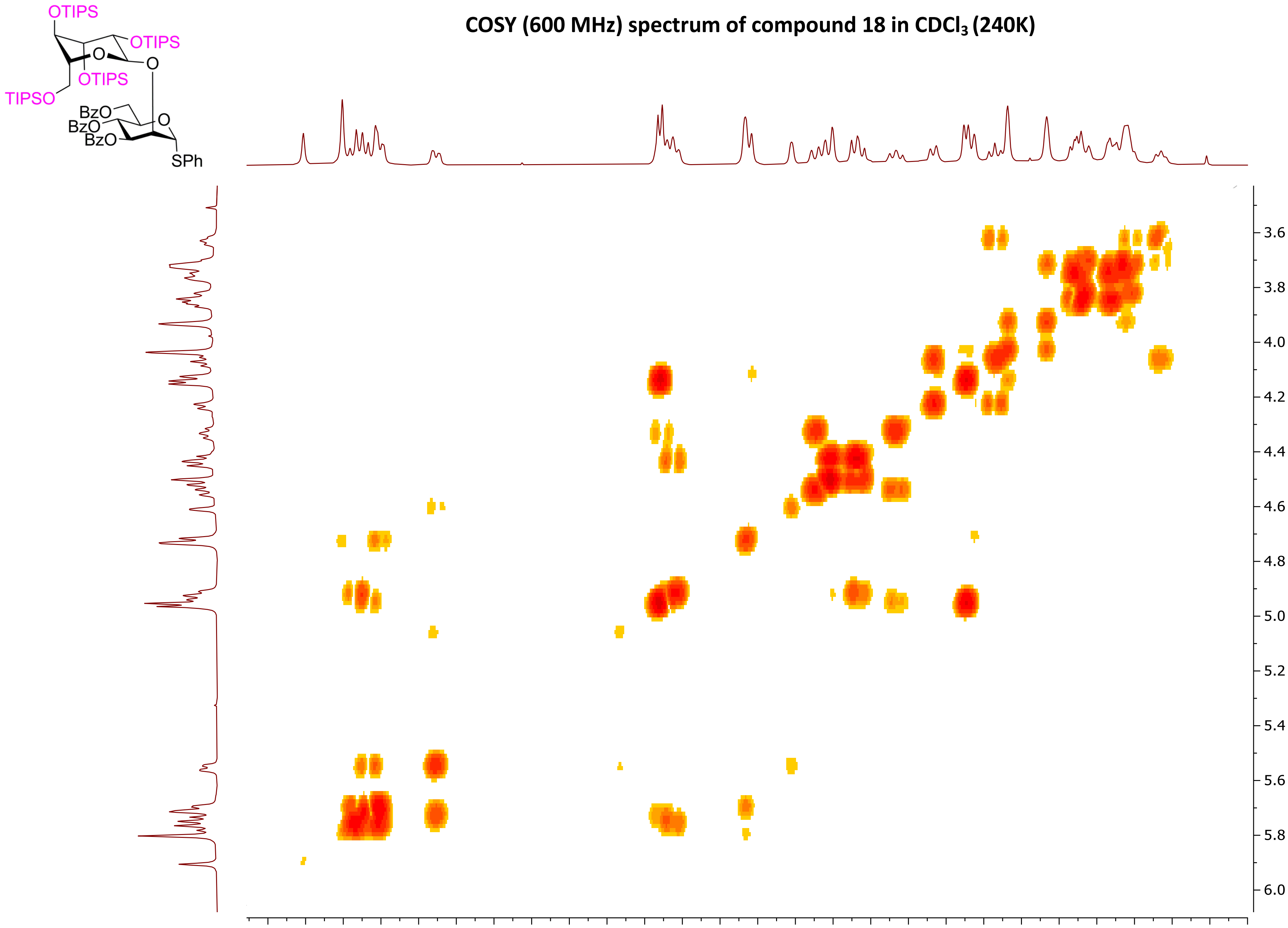

HSQC (600 MHz) spectrum of compound 18 in $CDCl_3$ (240K)

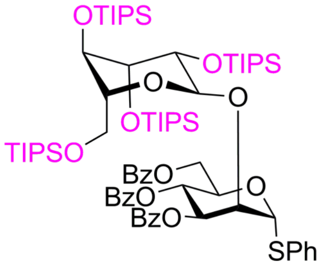

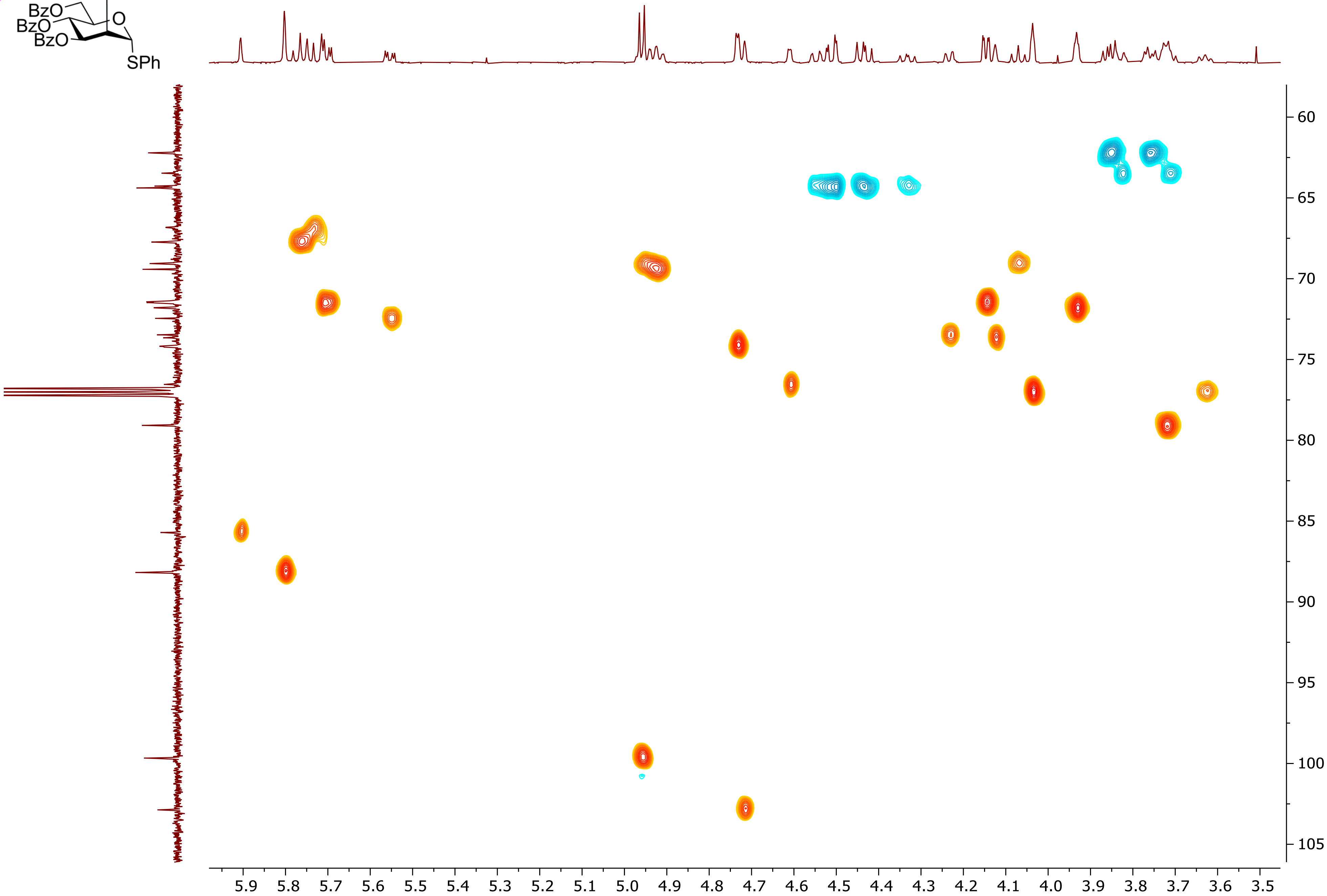

HMBC (600 MHz) spectrum of compound 18 in $CDCl_3$ (240K)

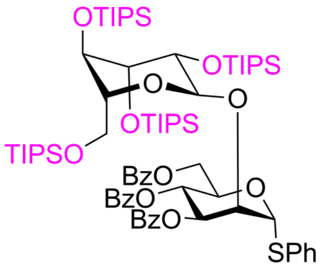

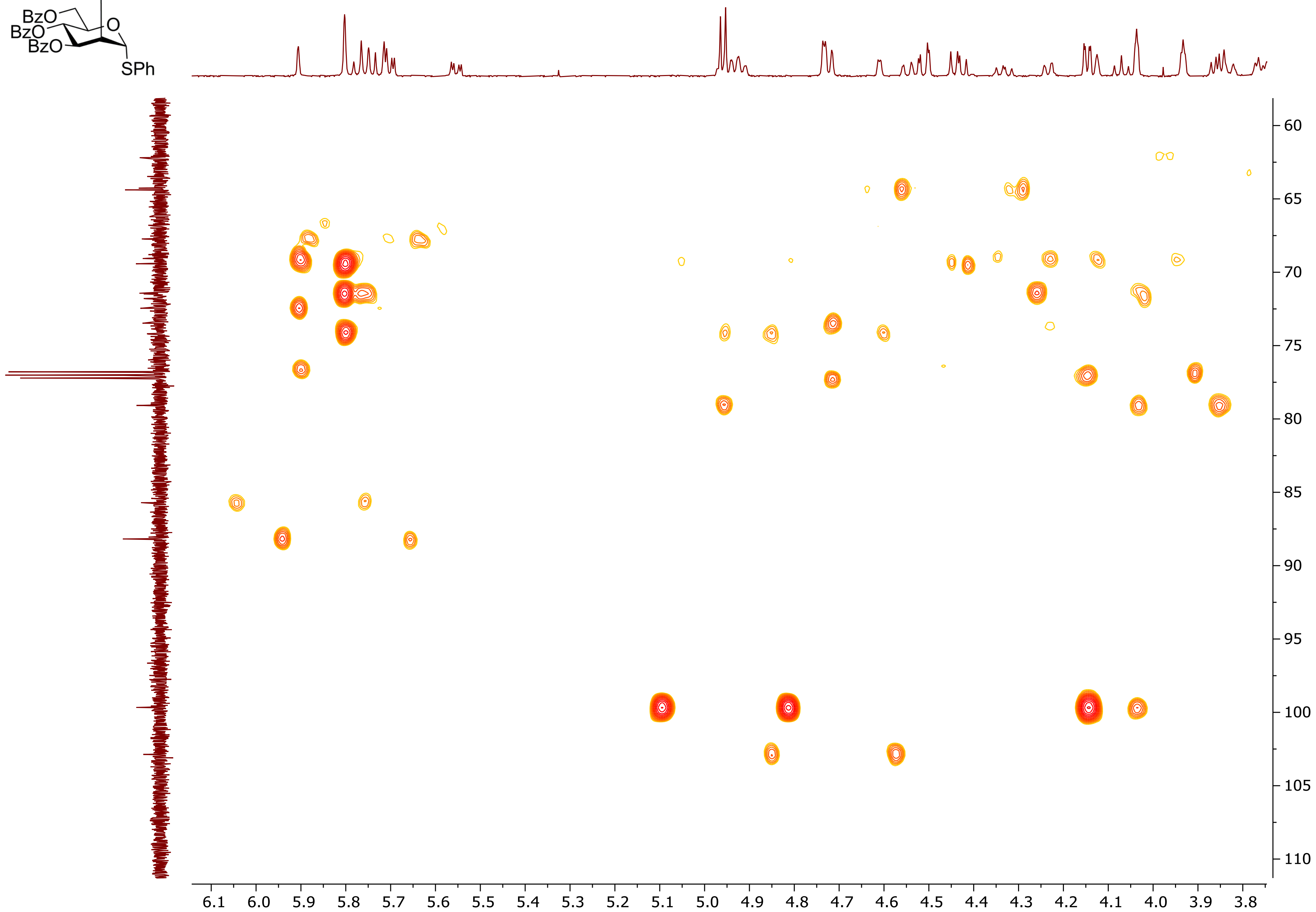

^{29}Si INEPT NMR (60 MHz) spectrum of compound 18 in $CDCl_3$ (240K)

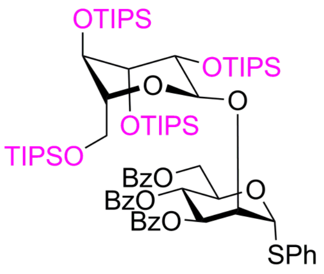

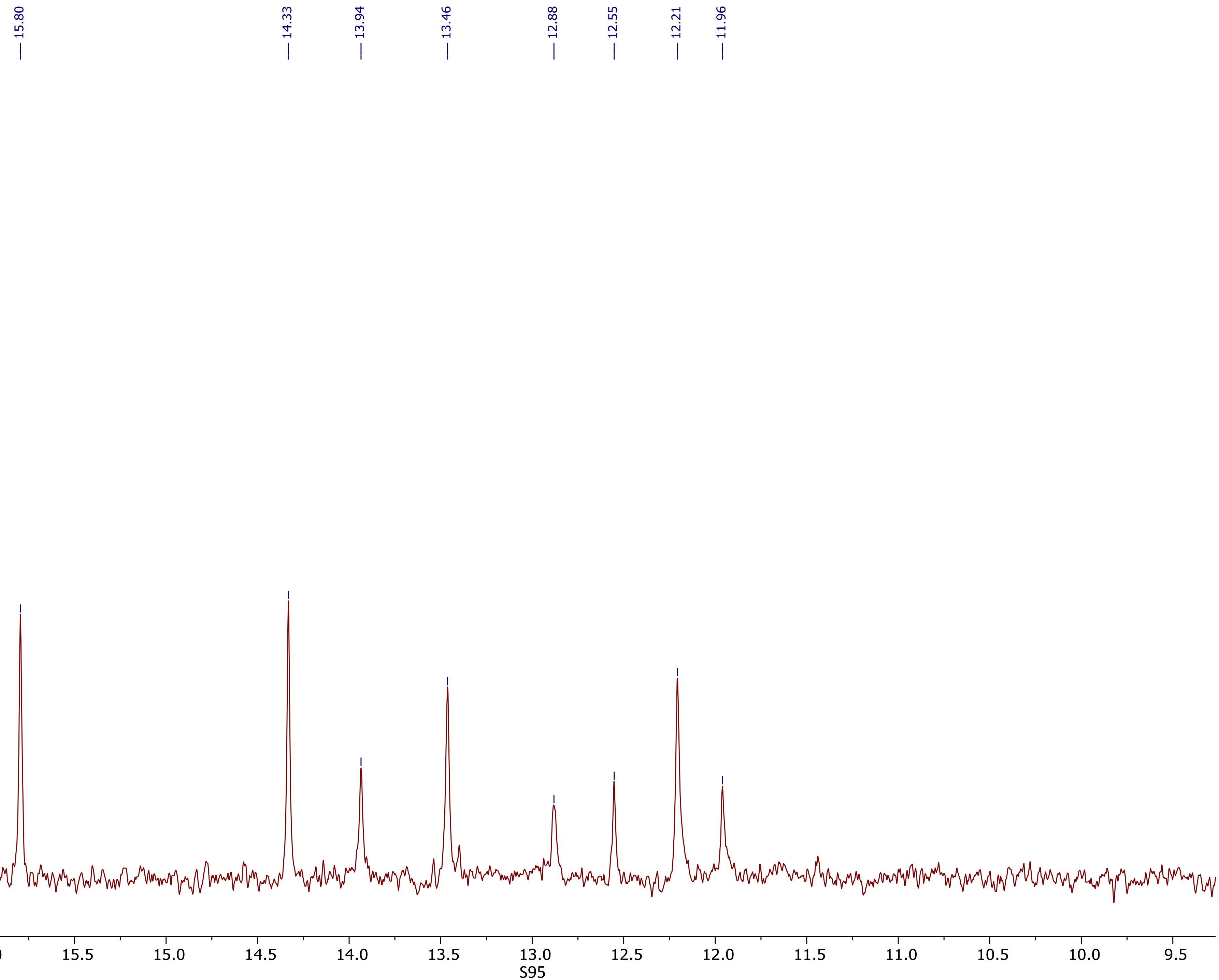

^{1}H NMR (600 MHz) spectrum of compound 22 in $CDCl_3$ (303K)

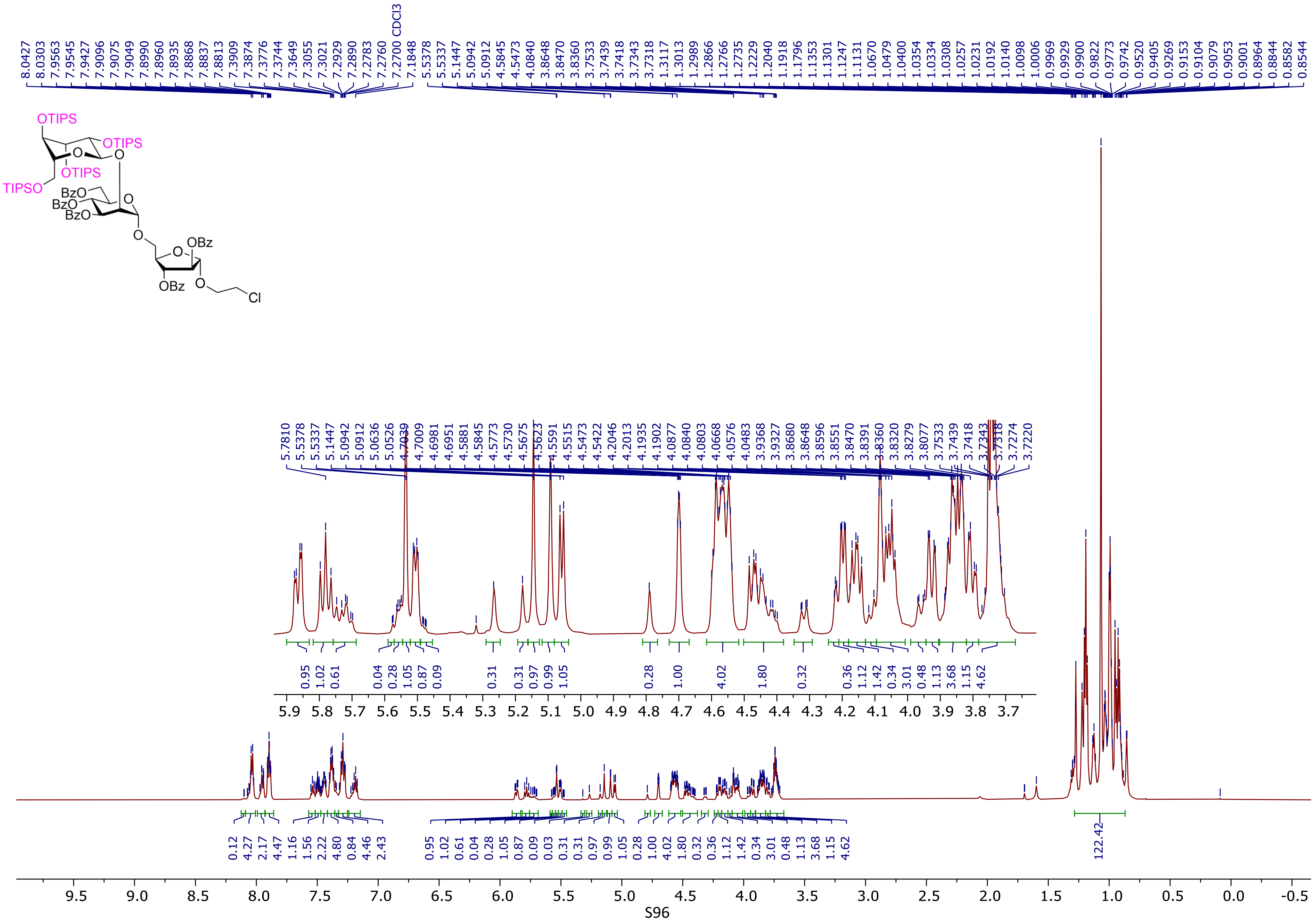

^{13}C NMR (151 MHz) spectrum of compound 22 in $CDCl_3$ (303K)

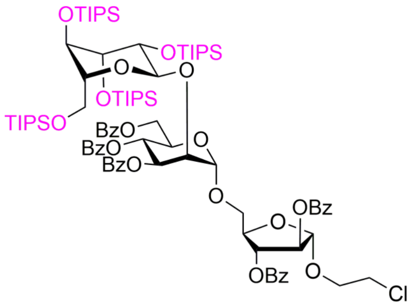

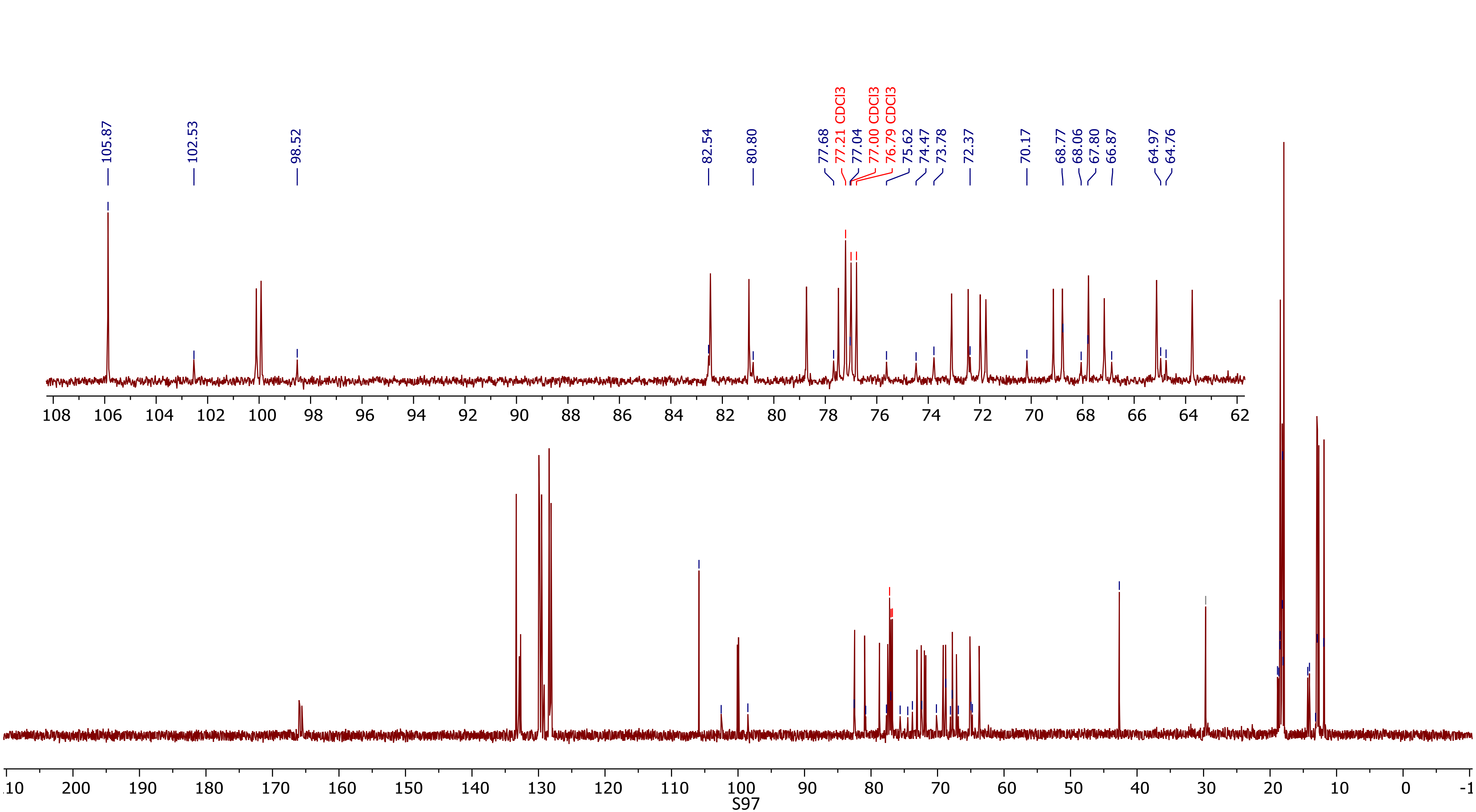

COSY (600 MHz) spectrum of compound 22 in $CDCl_3$ (303K)

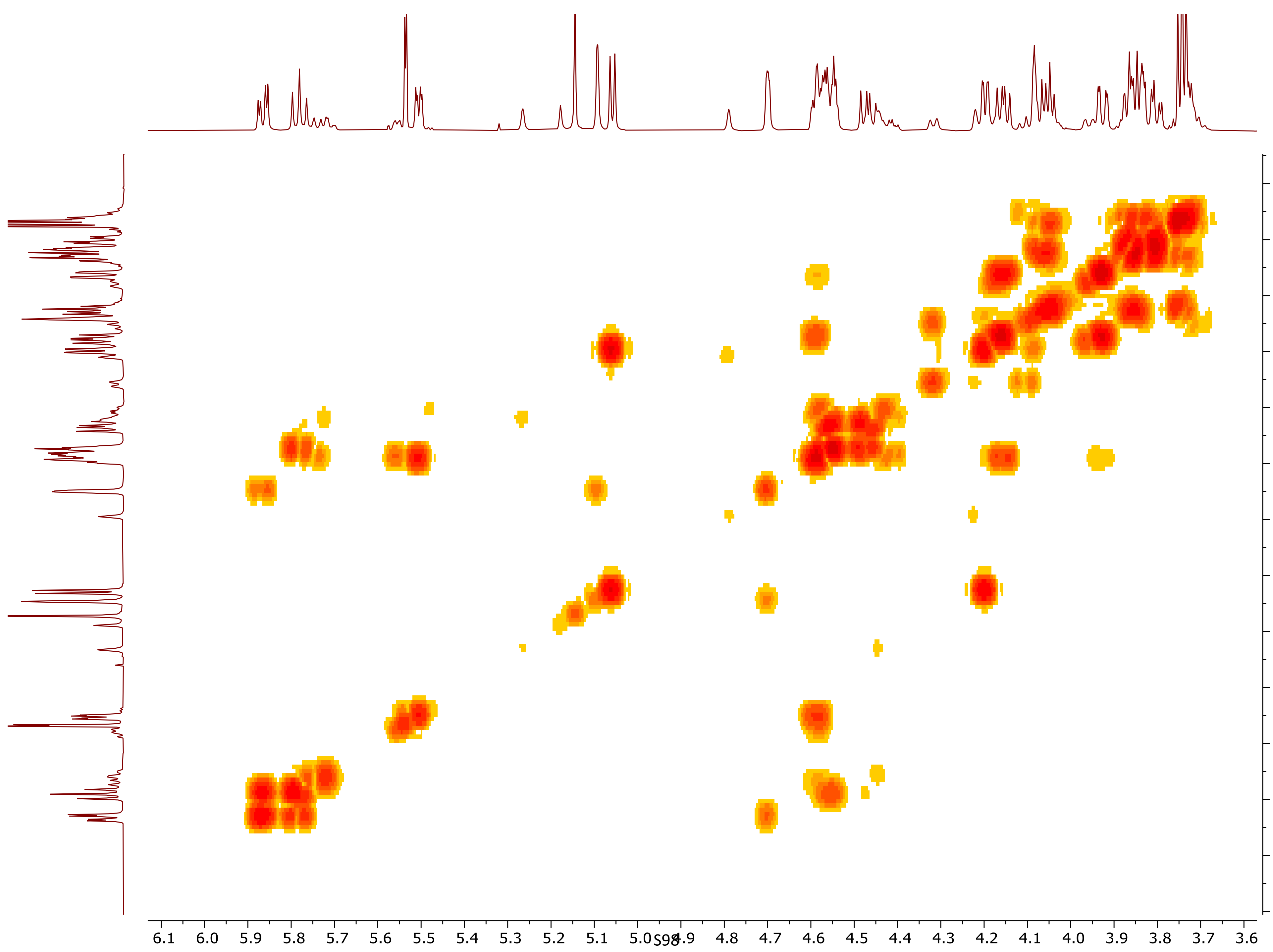

HSQC (600 MHz) spectrum of compound 22 in $CDCl_3$ (303K)

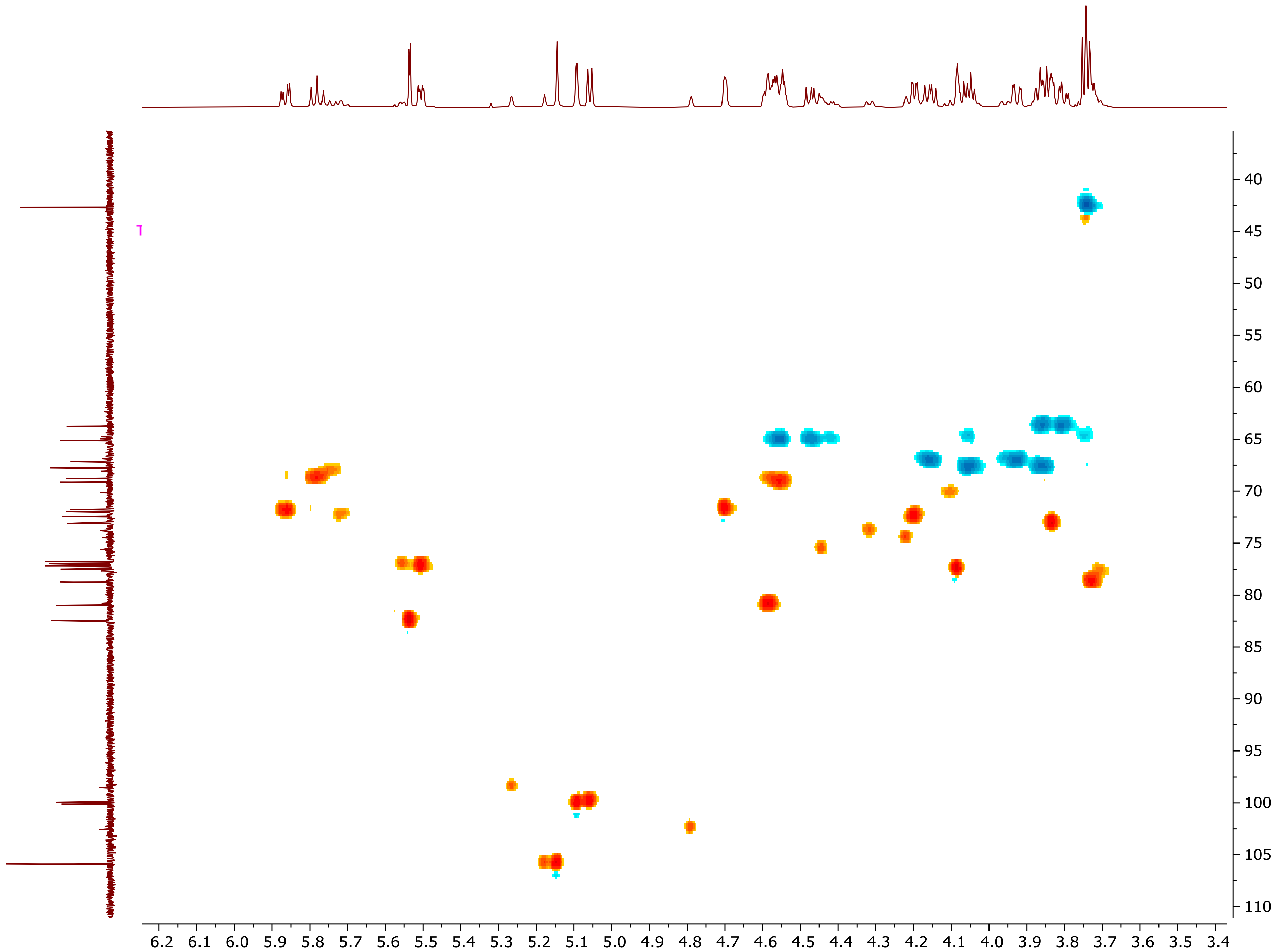

HMBC (600 MHz) spectrum of compound 22 in $CDCl_3$ (303K)

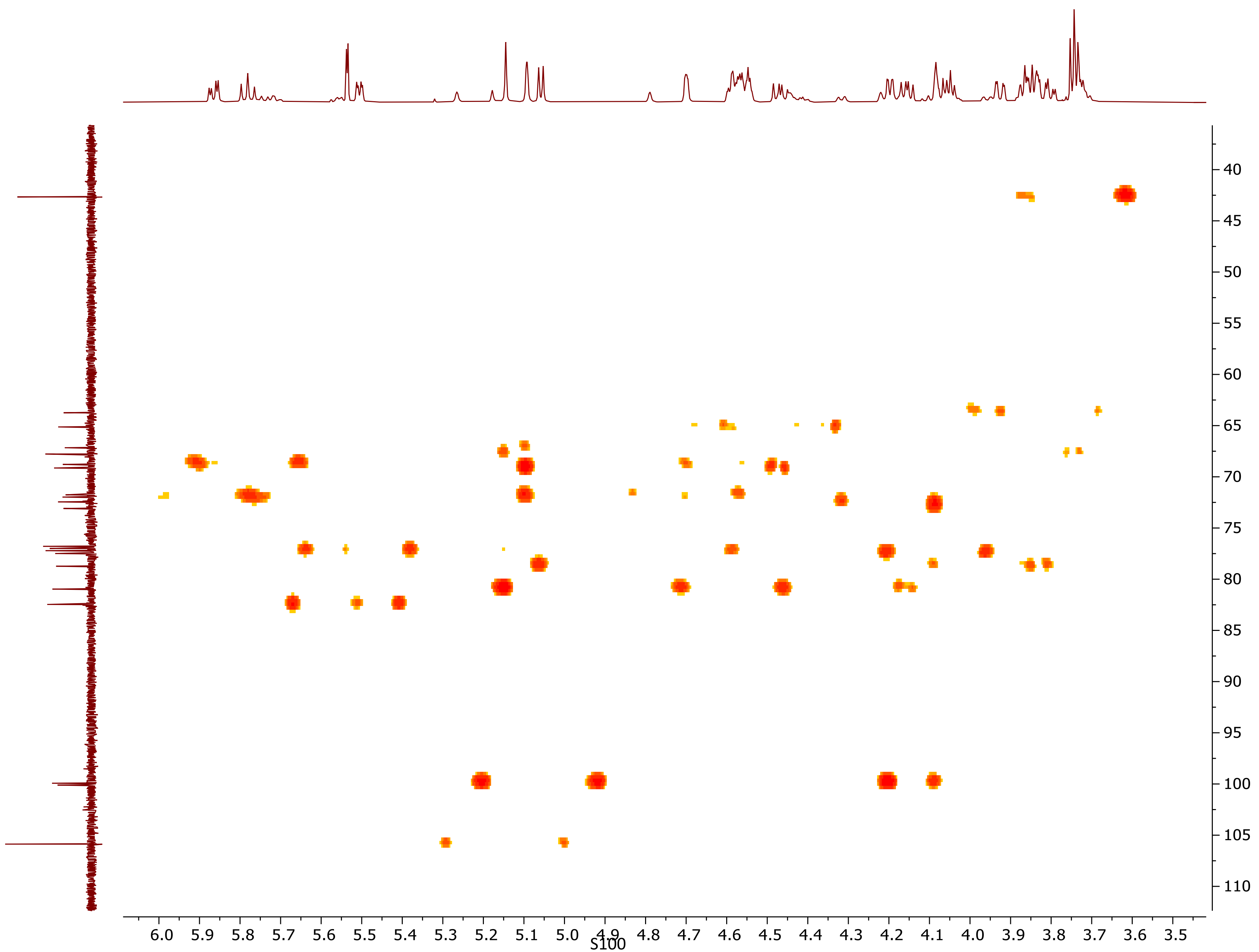

^{29}Si INEPT NMR (60 MHz) spectrum of compound 22 in $CDCl_3$ (303K)

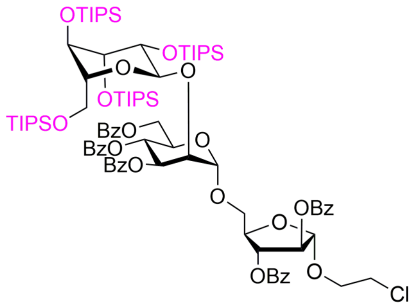

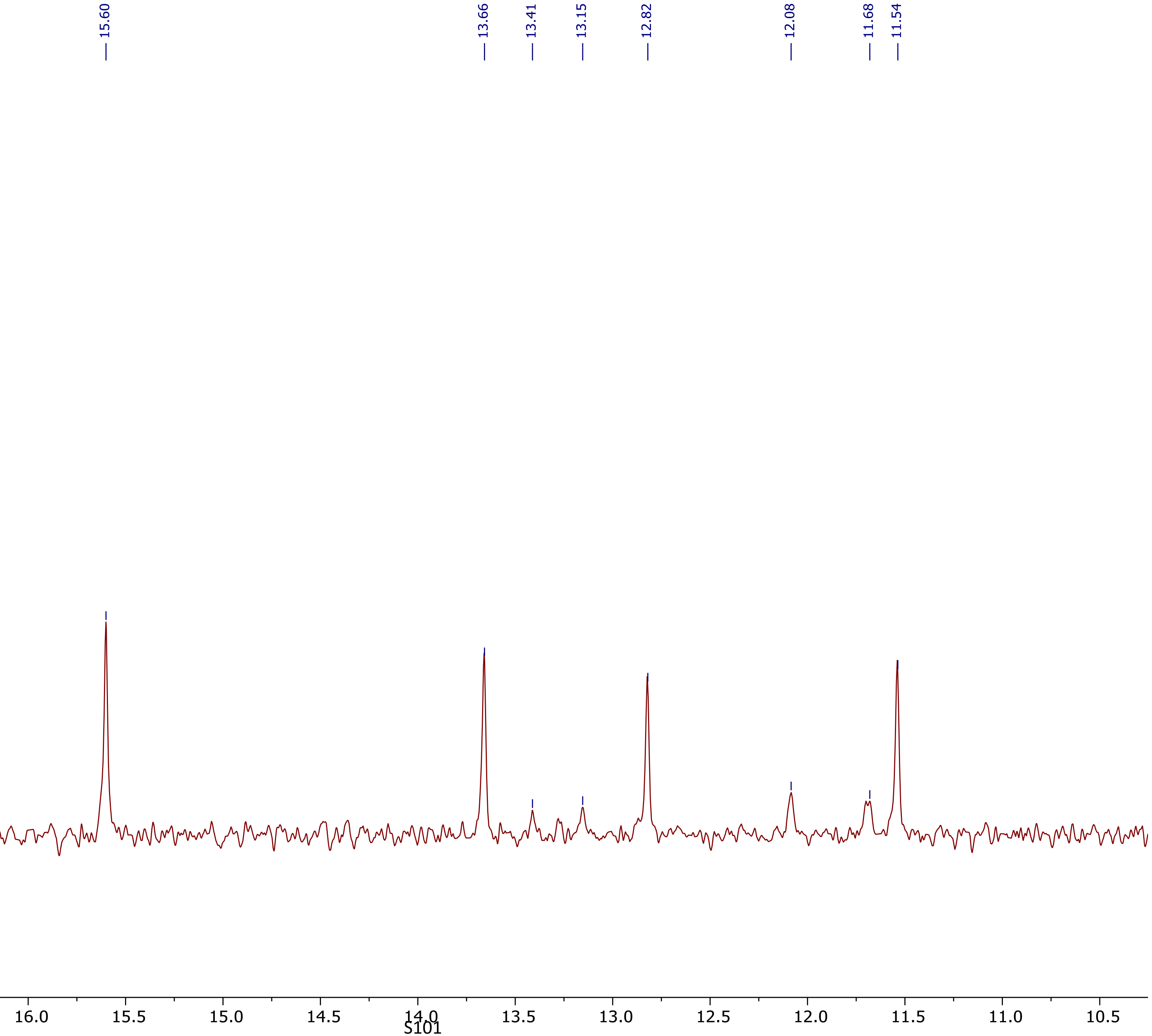

^{1}H NMR (600 MHz) spectrum of compound 22 in $CDCl_3$ (236K)

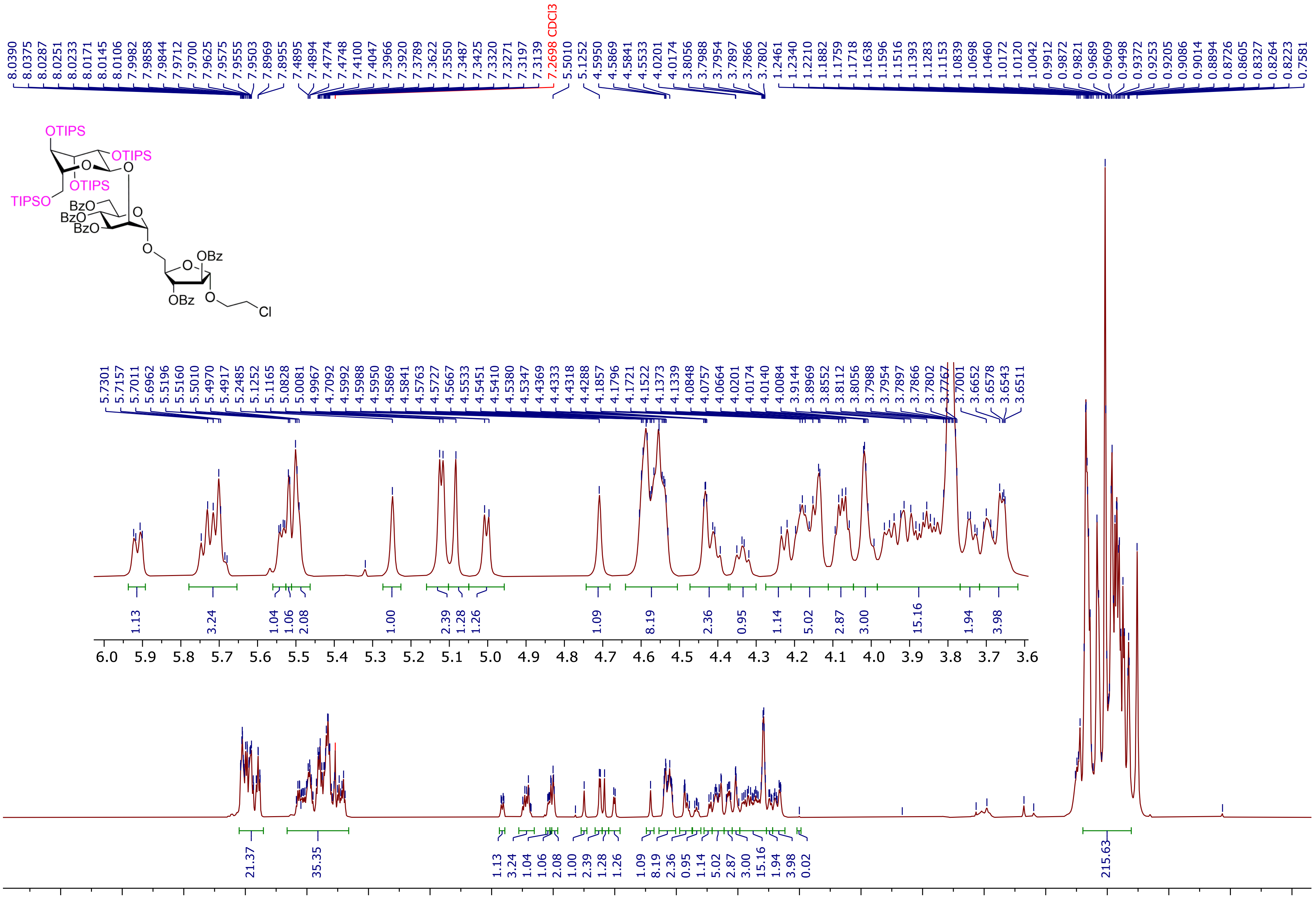

^{13}C NMR (151 MHz) spectrum of compound 22 in $CDCl_3$ (236K)

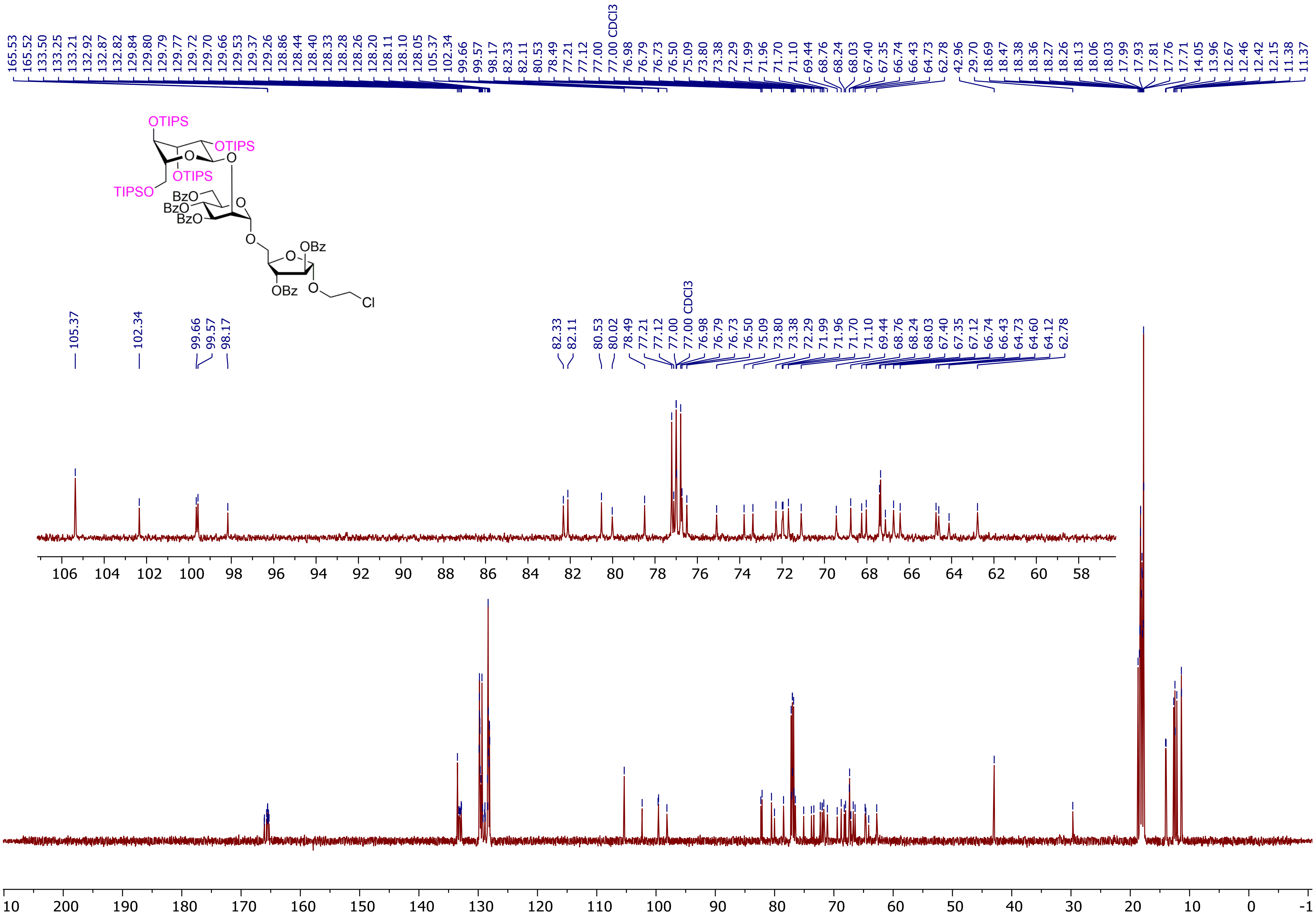

COSY (600 MHz) spectrum of compound 22 in $CDCl_3$ (236K)

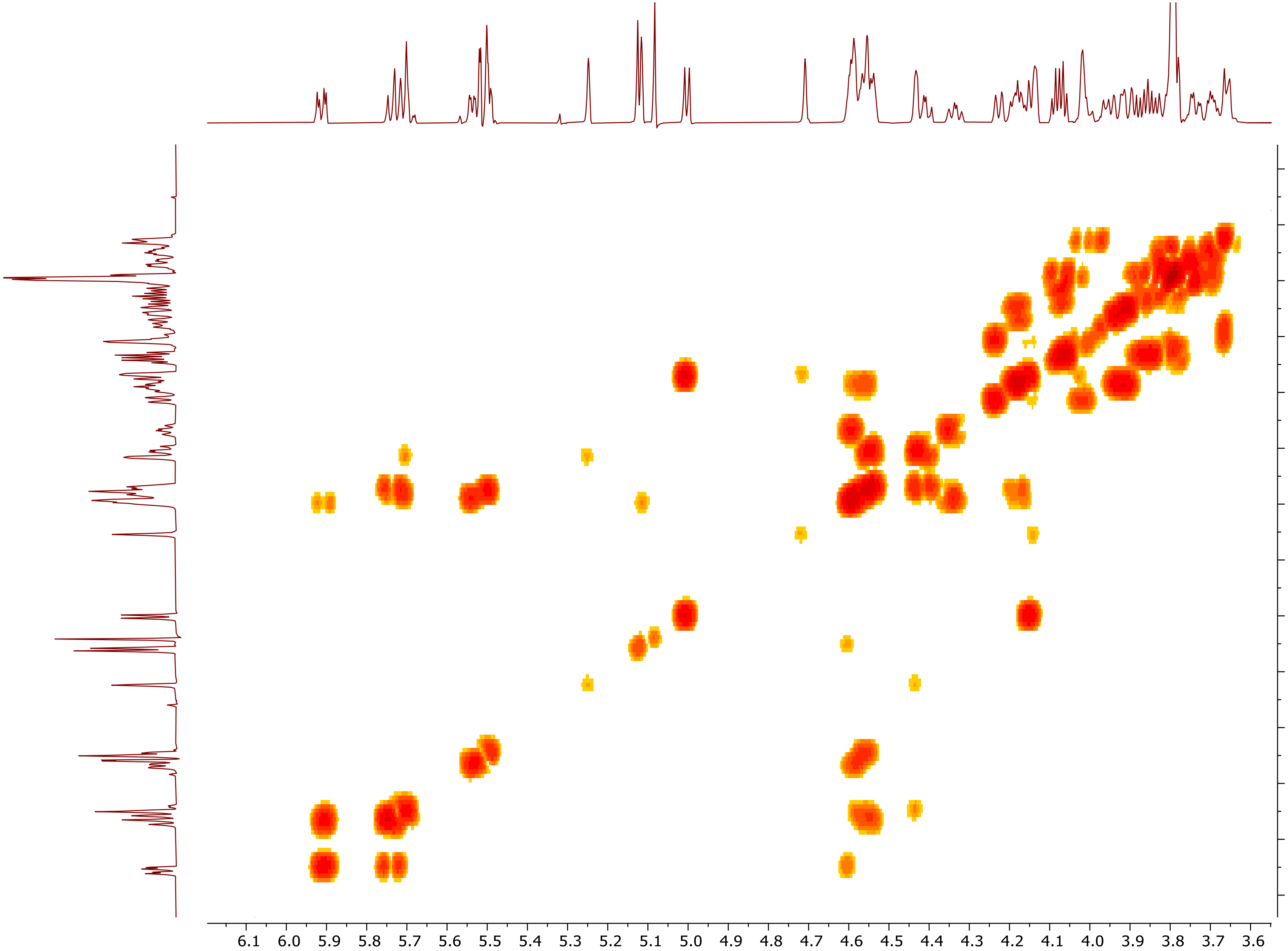

HSQC (600 MHz) spectrum of compound 22 in $CDCl_3$ (236K)

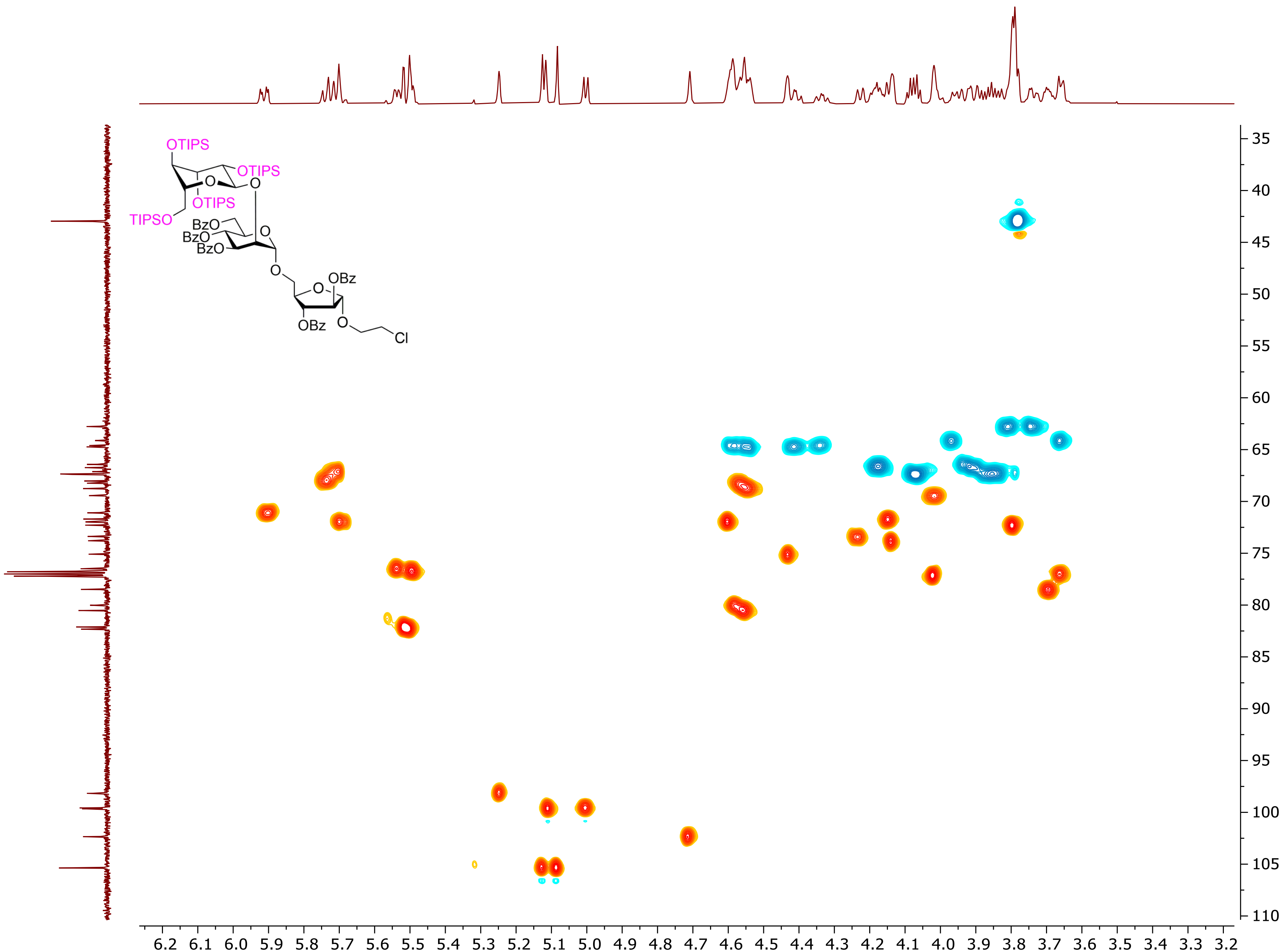

HMBC (600 MHz) spectrum of compound 22 in $CDCl_3$ (236K)

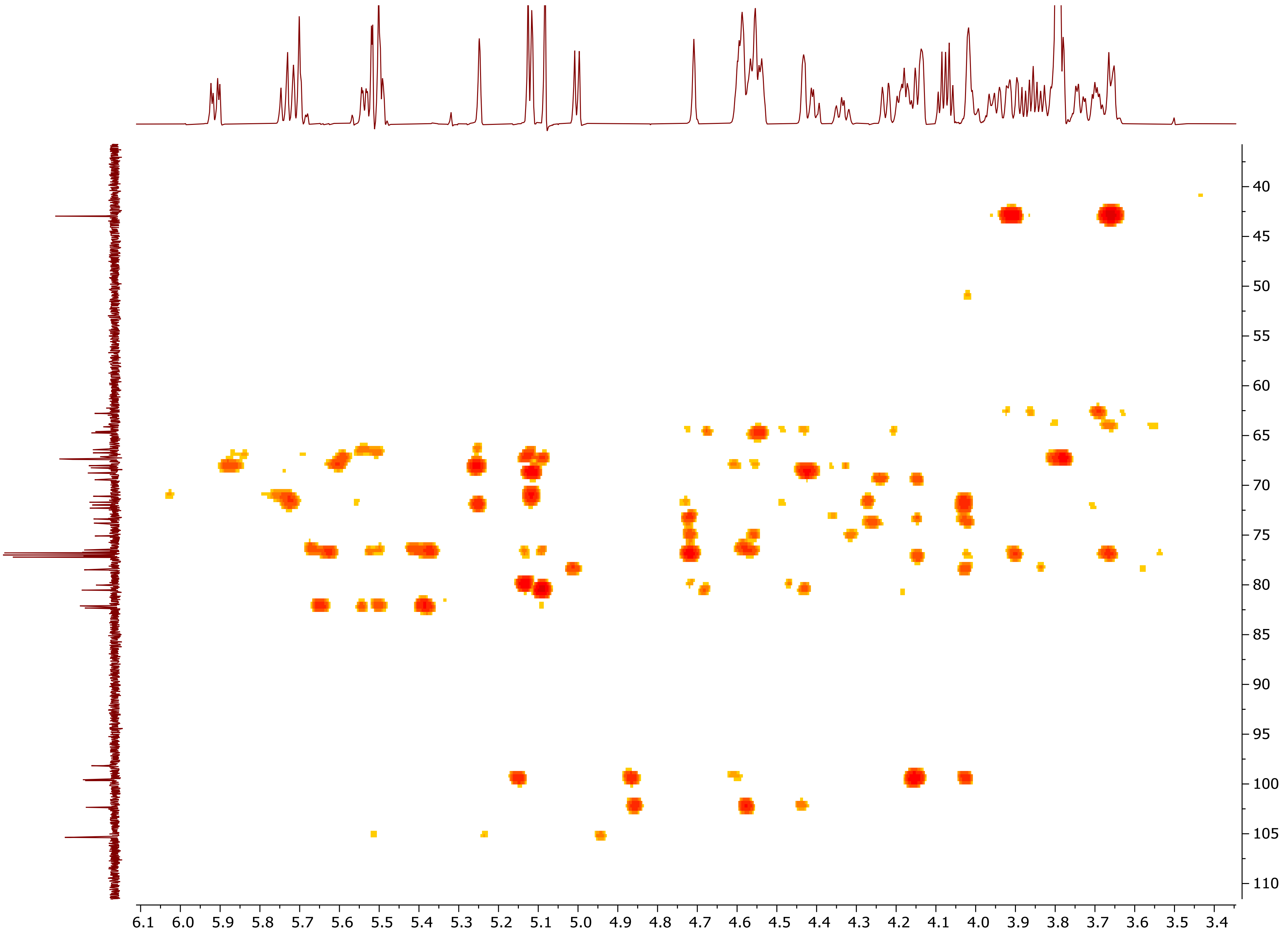

^{29}Si INEPT NMR (119 MHz) spectrum of compound 22 in $CDCl_3$ (236K)

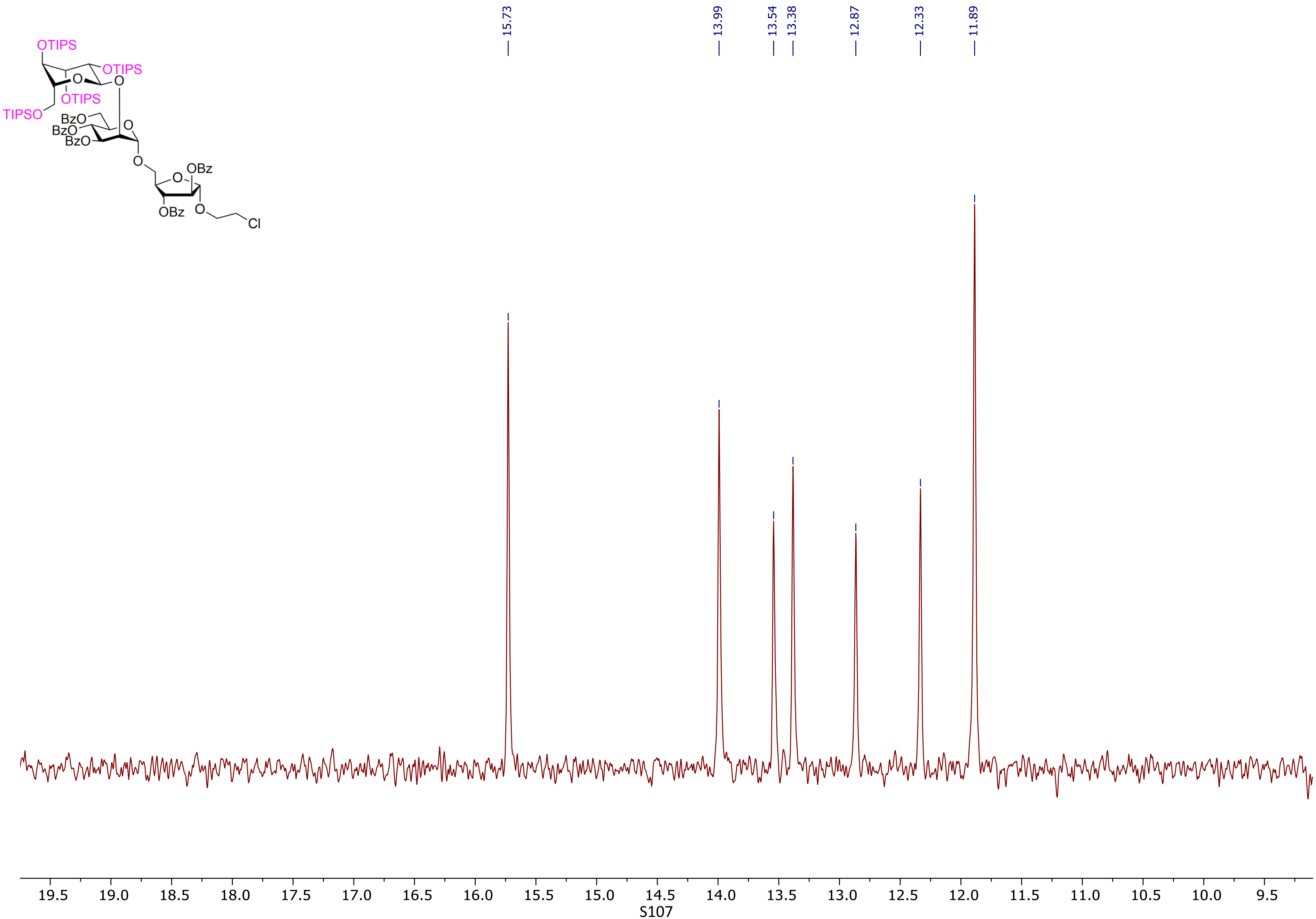

^{1}H NMR (600 MHz) spectrum of compound 23 in $CDCl_3$

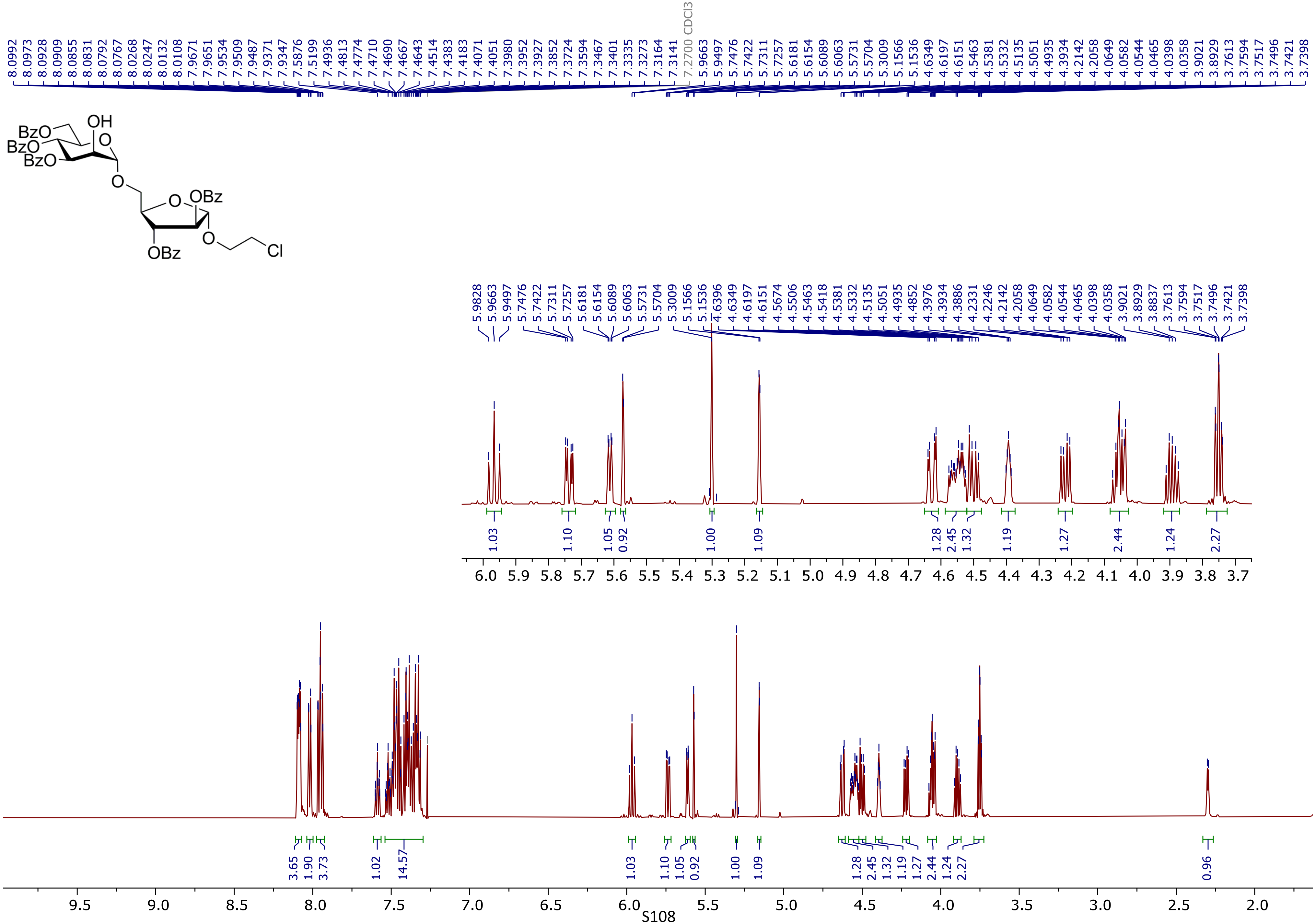

^{13}C NMR (151 MHz) spectrum of compound 23 in $CDCl_3$

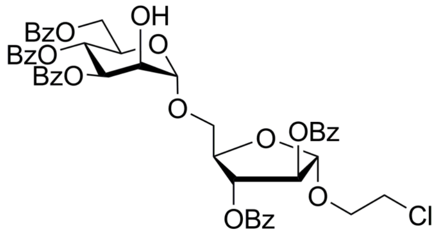

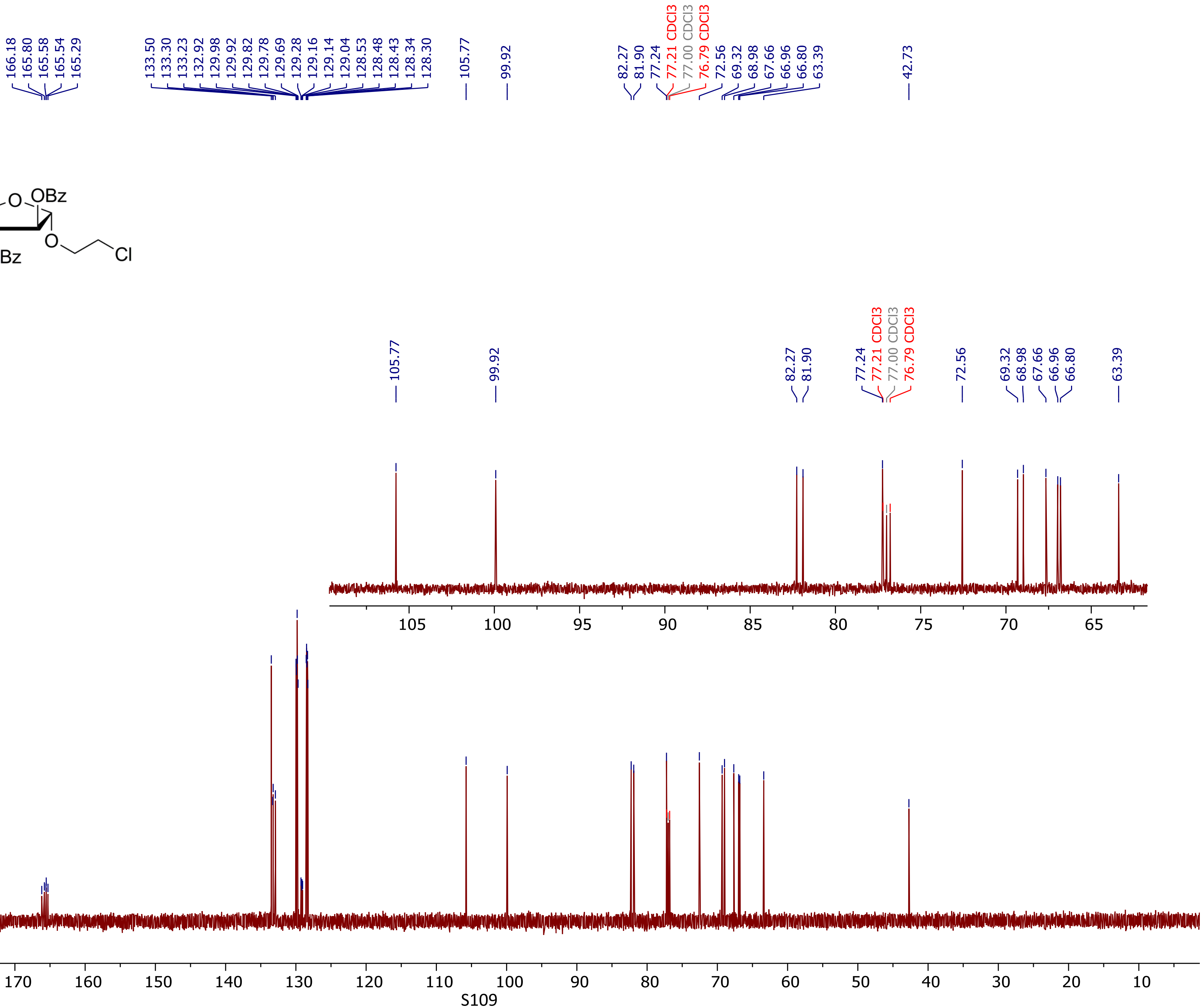

COSY (600 MHz) spectrum of compound 23 in $CDCl_3$

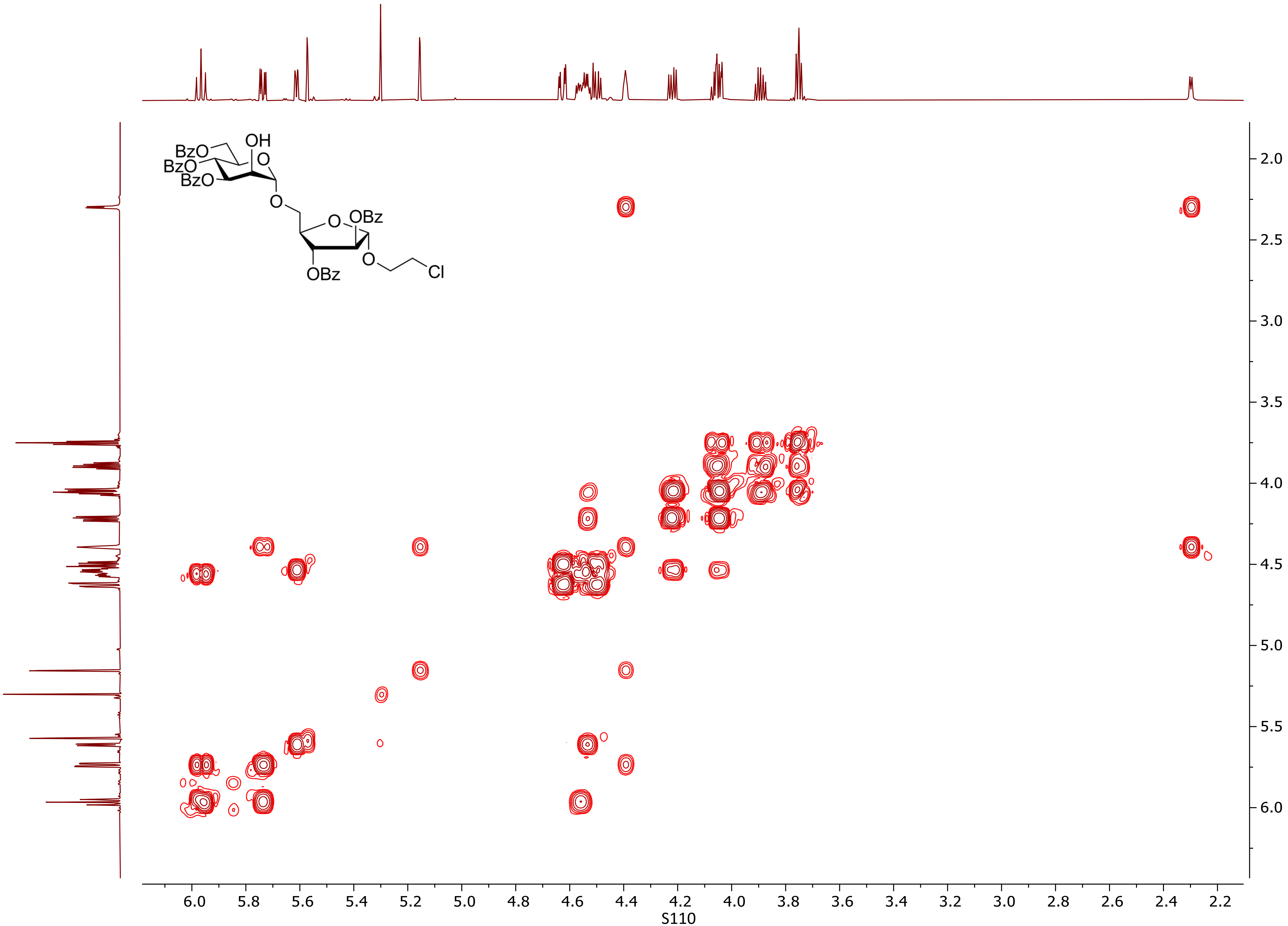

HSQC (600 MHz) spectrum of compound 23 in $CDCl_3$

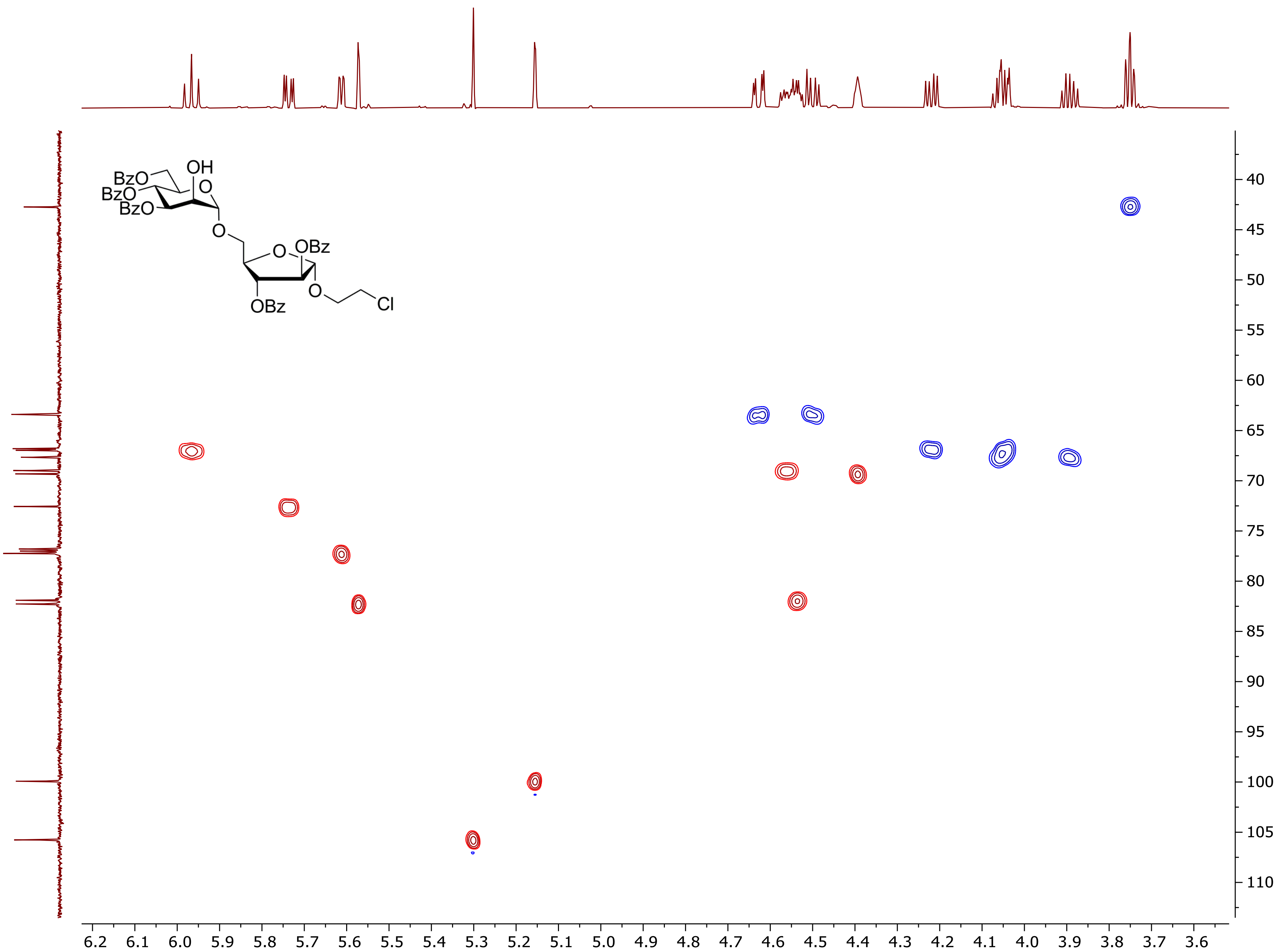

HMBC (600 MHz) spectrum of compound 23 in $CDCl_3$

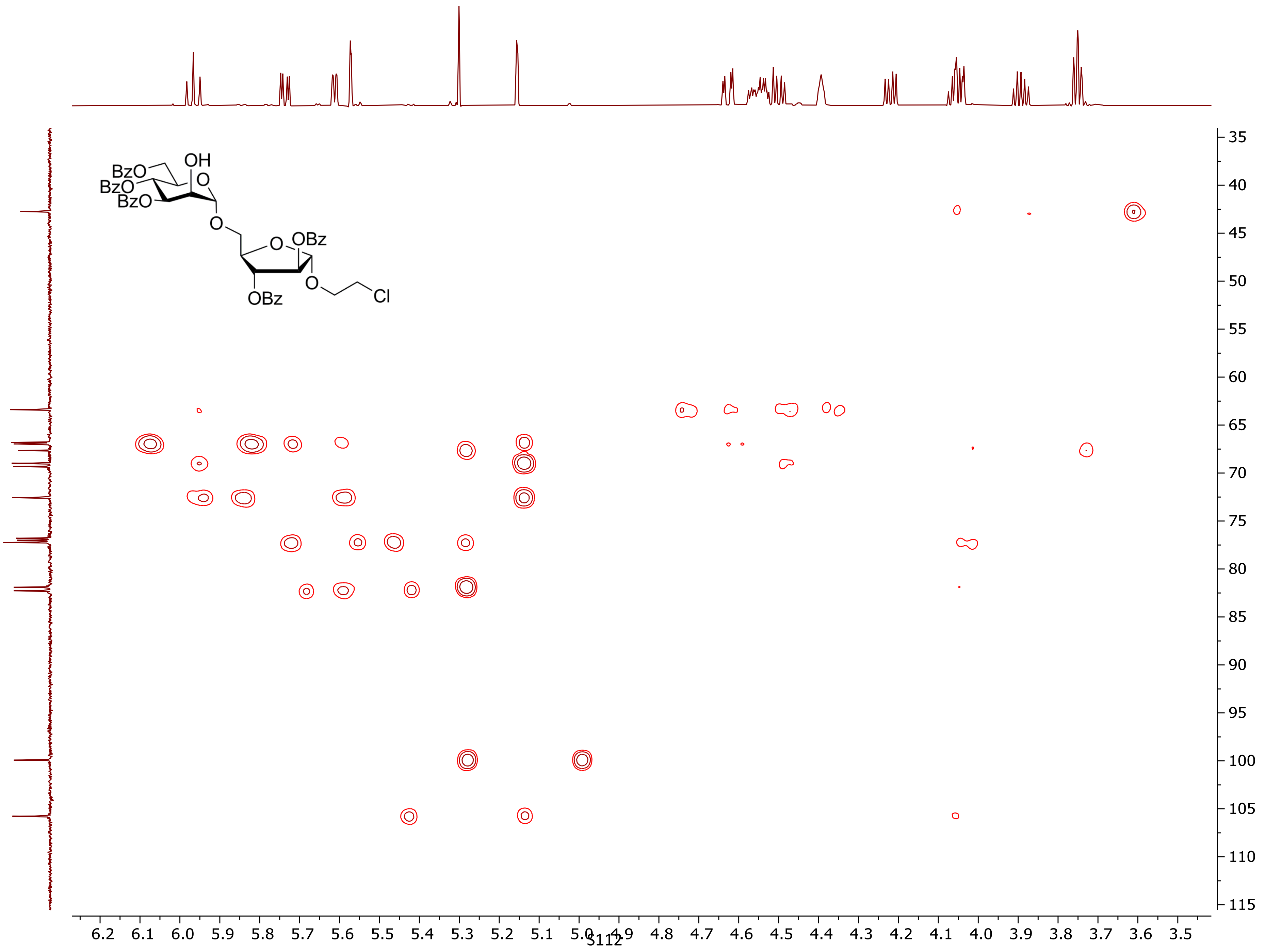

^{1}H NMR (600 MHz) spectrum of compound 24 in $CDCl_3$ (303K)

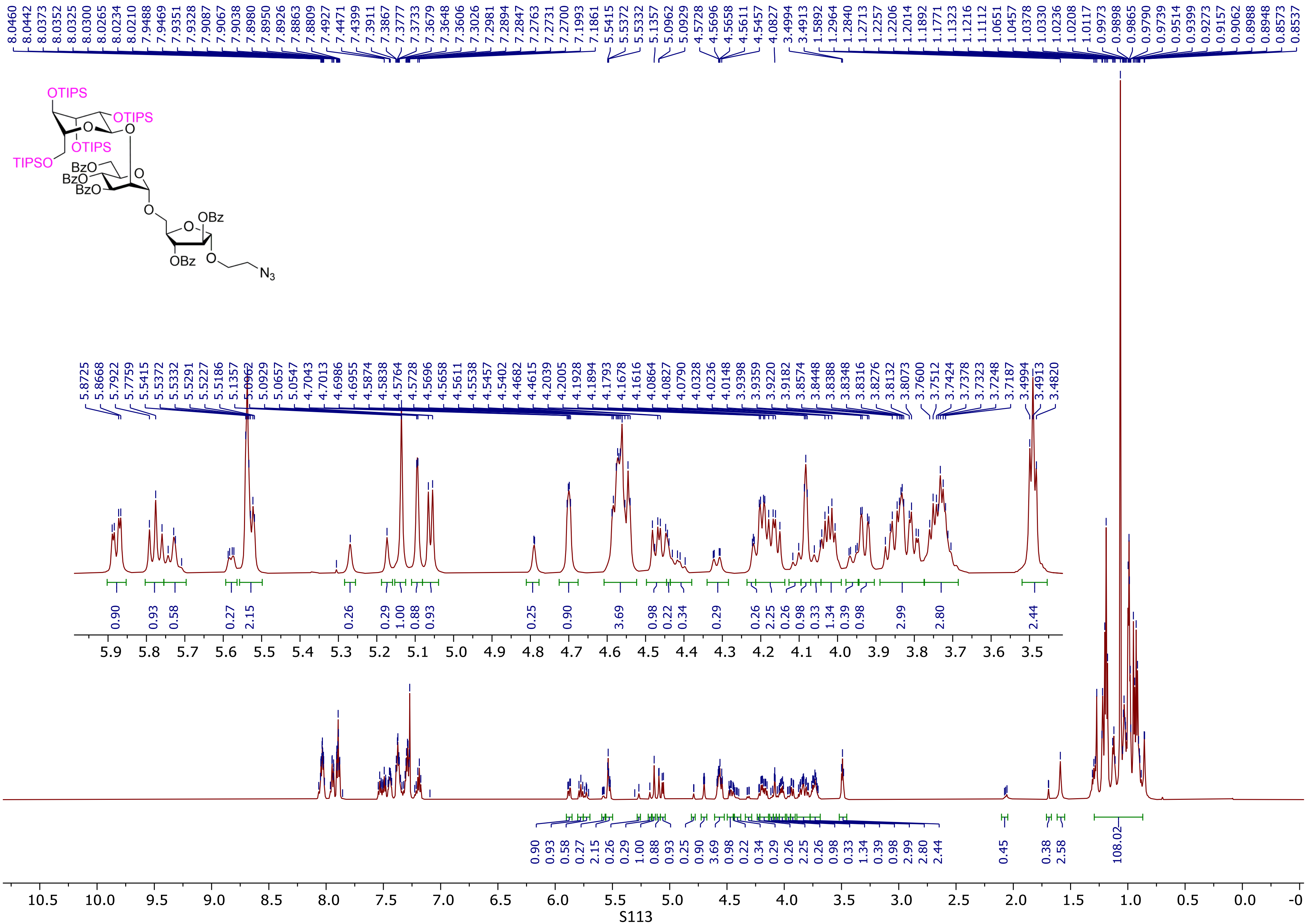

^{13}C NMR (151 MHz) spectrum of compound 24 in $CDCl_3$ (303K)

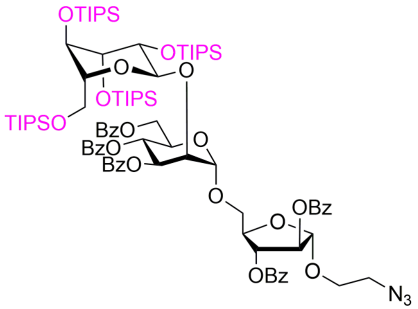

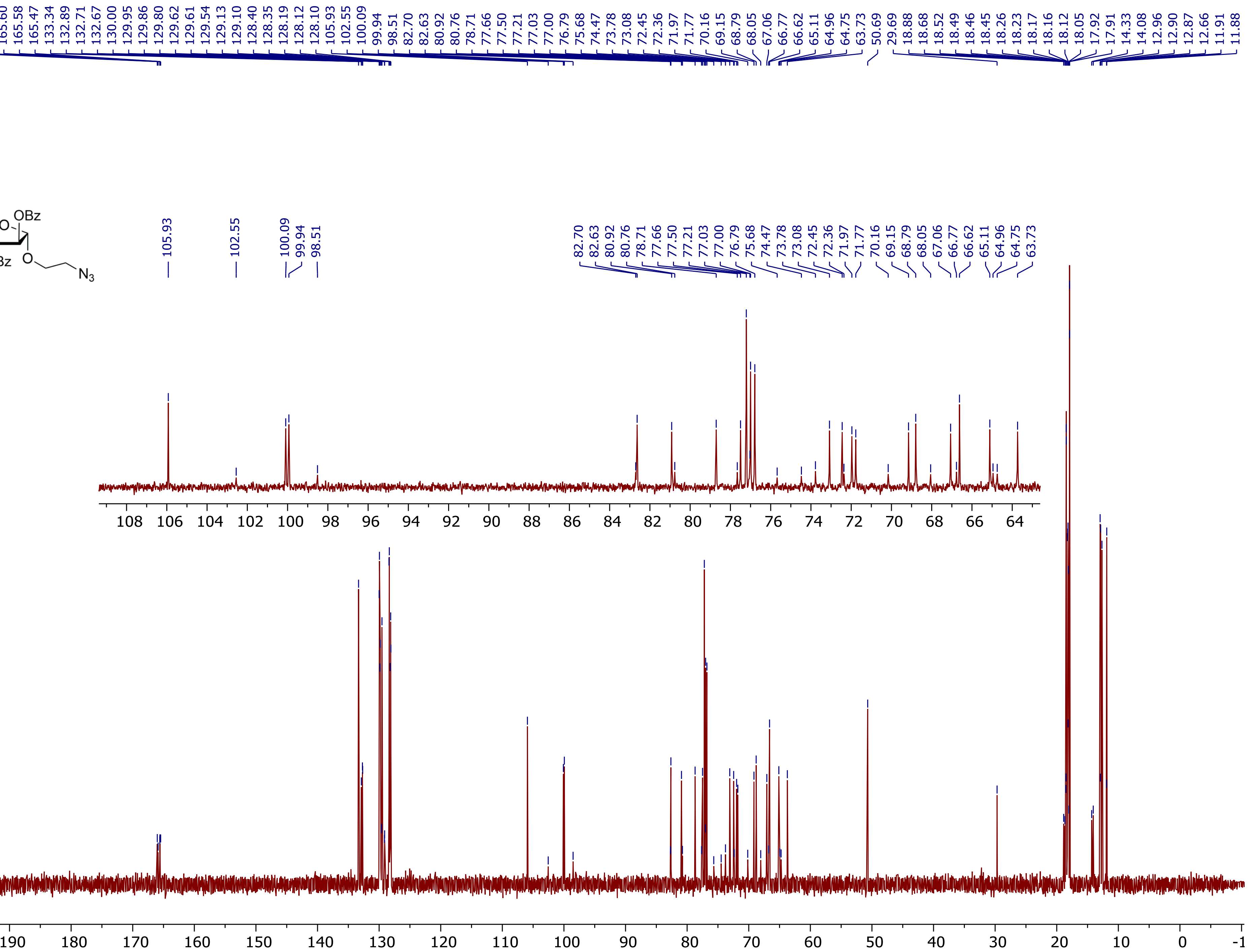

COSY (600 MHz) spectrum of compound 24 in $CDCl_3$ (303K)

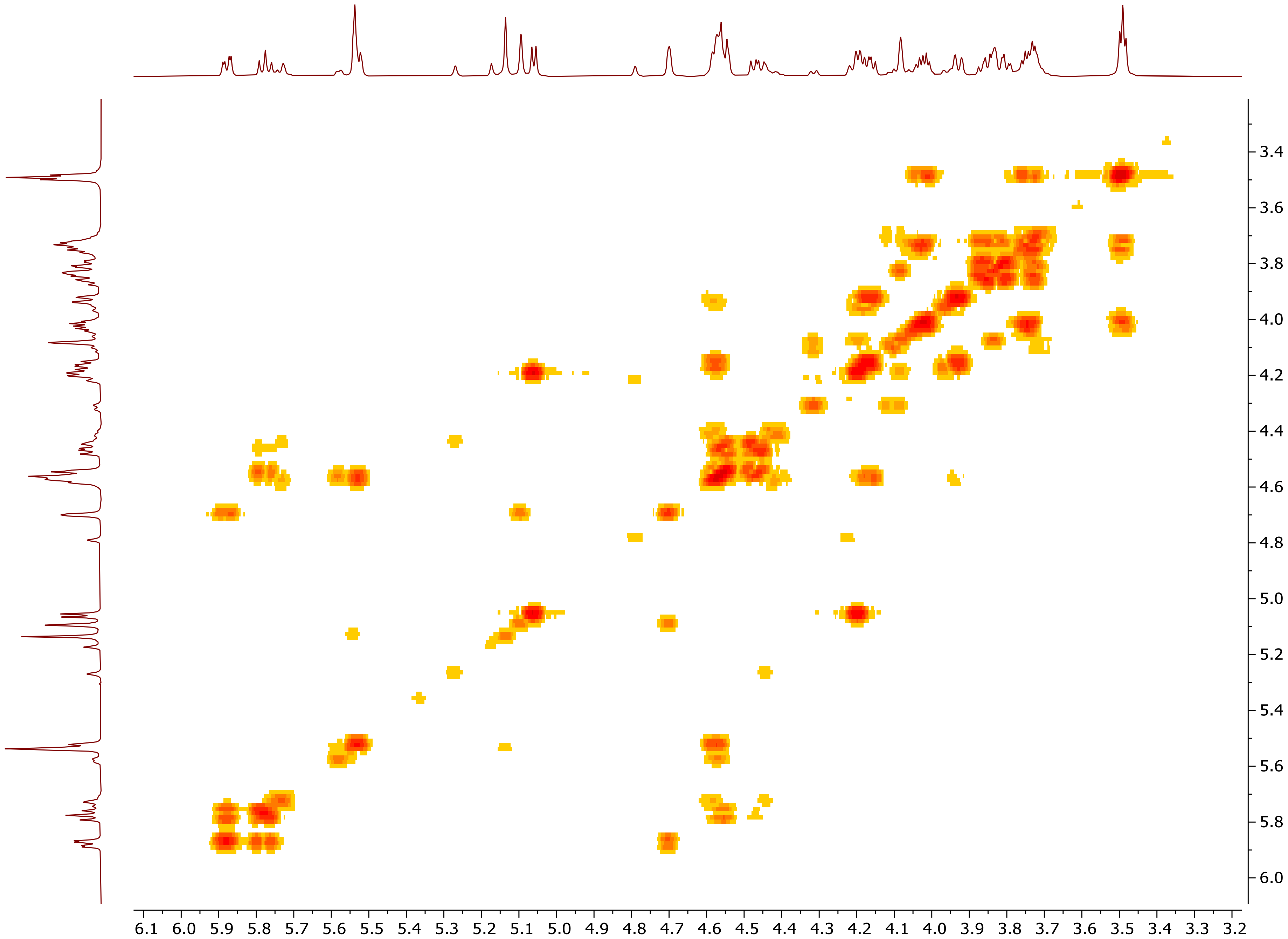

HSQC (600 MHz) spectrum of compound of compound 24 in $CDCl_3$ (303K)

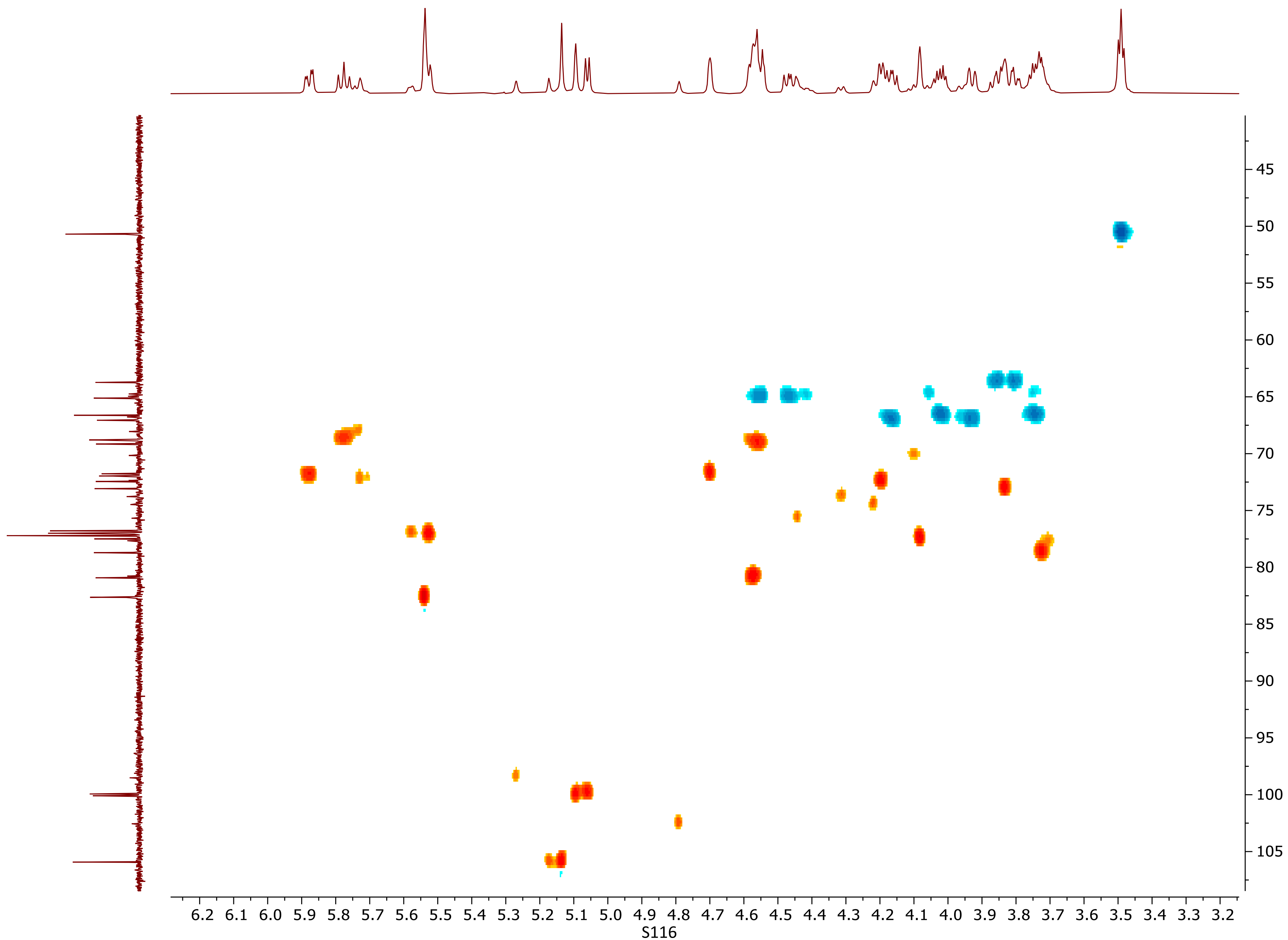

HMBC (600 MHz) spectrum of compound of compound 24 in $CDCl_3$ (303K)

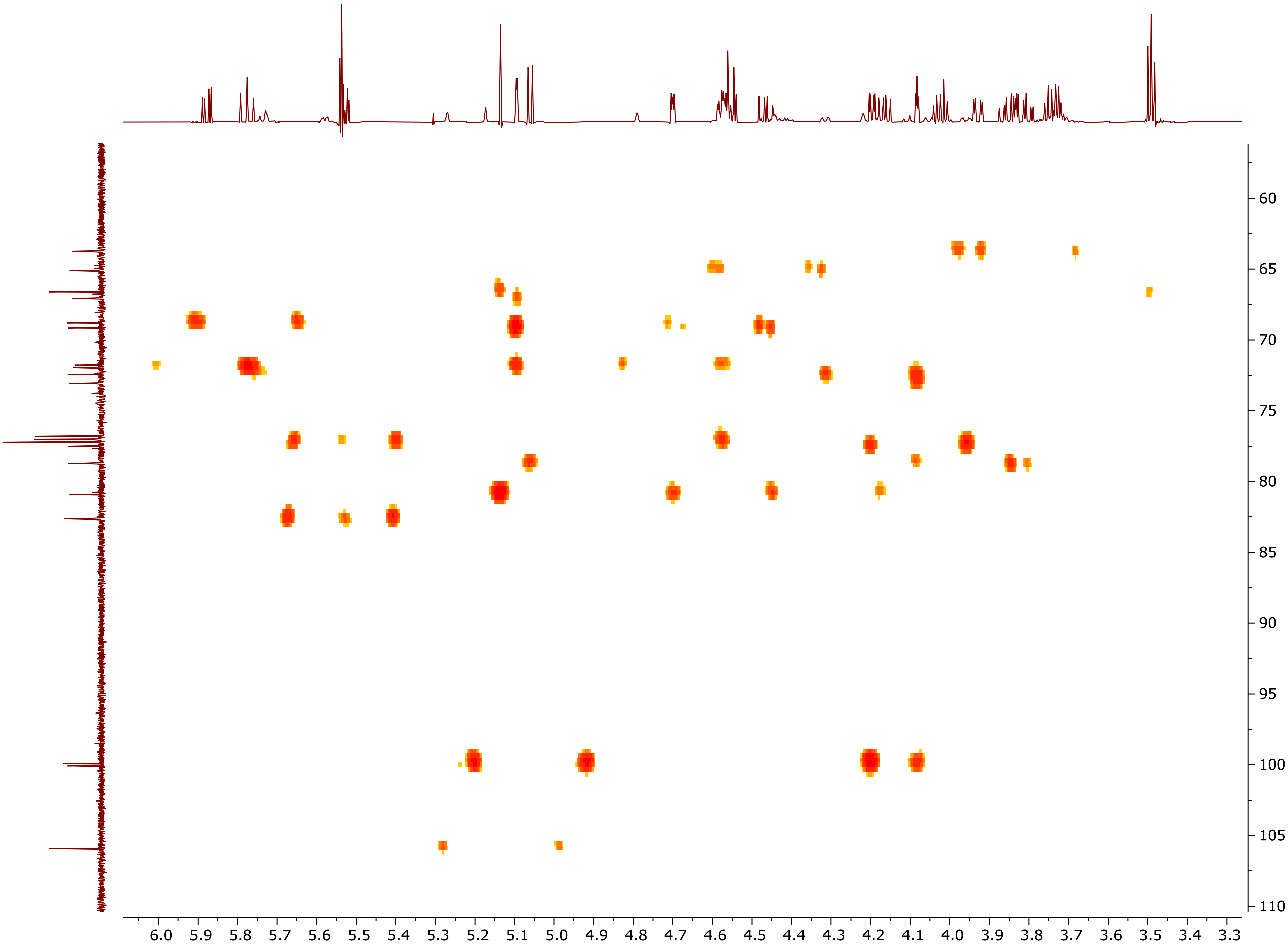

^{29}Si INEPT NMR (60 MHz) spectrum of compound 24 in $CDCl_3$ (303K)

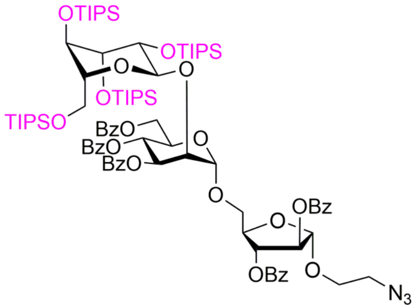

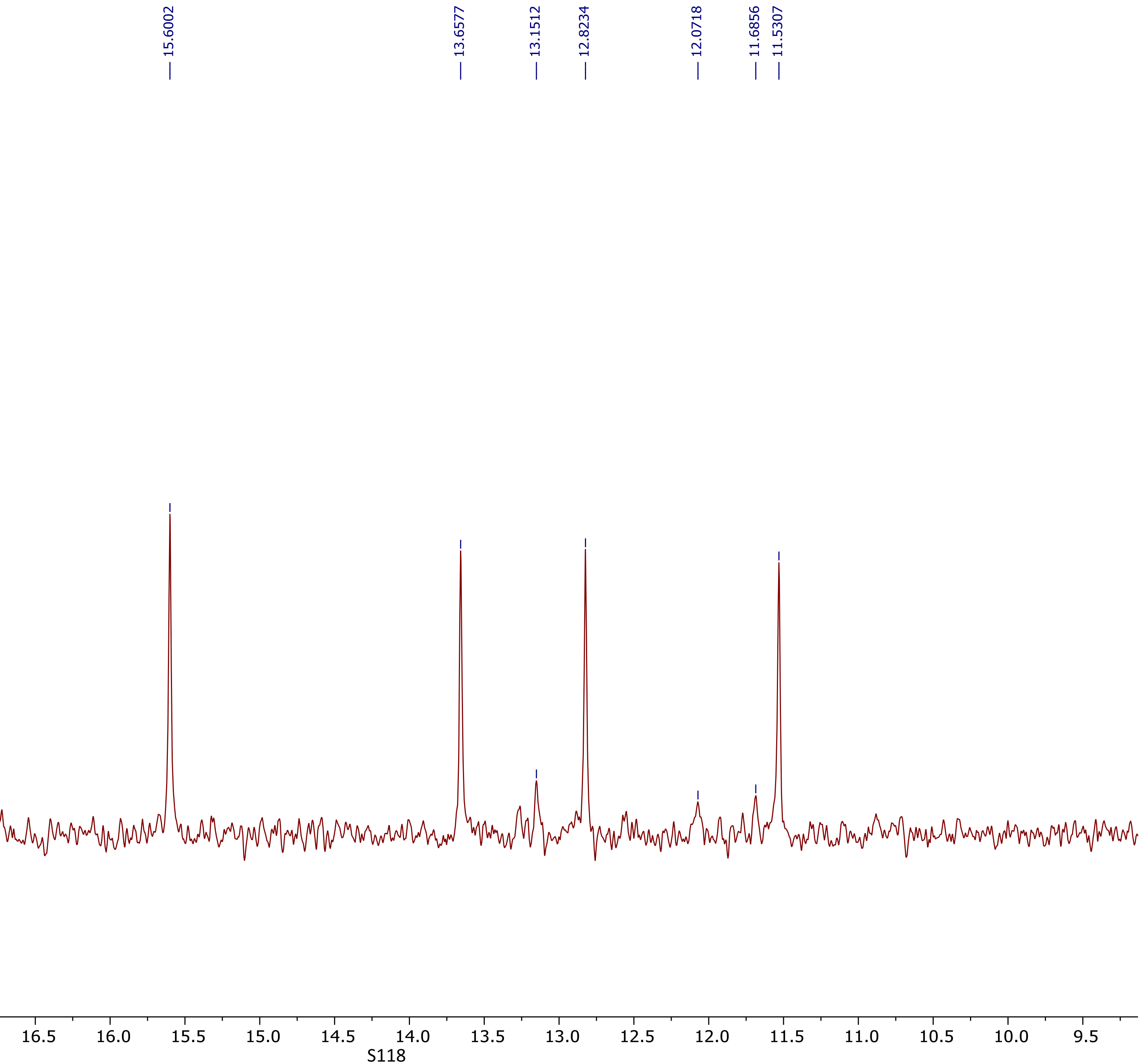

^{1}H NMR (600 MHz) spectrum of compound 24 in $CDCl_3$ (243K)

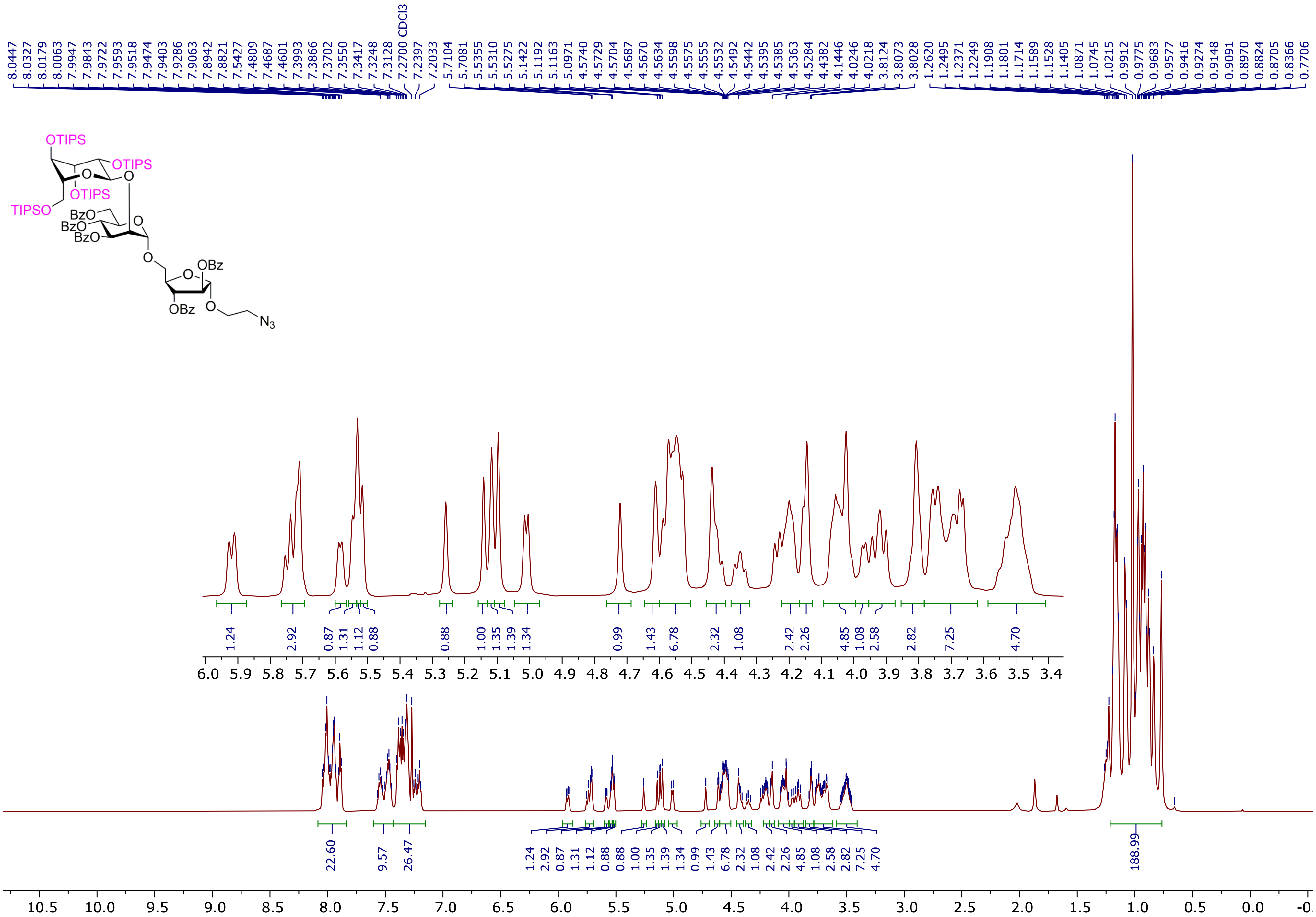

^{13}C NMR (151 MHz) spectrum of compound 24 in $CDCl_3$ (244K)

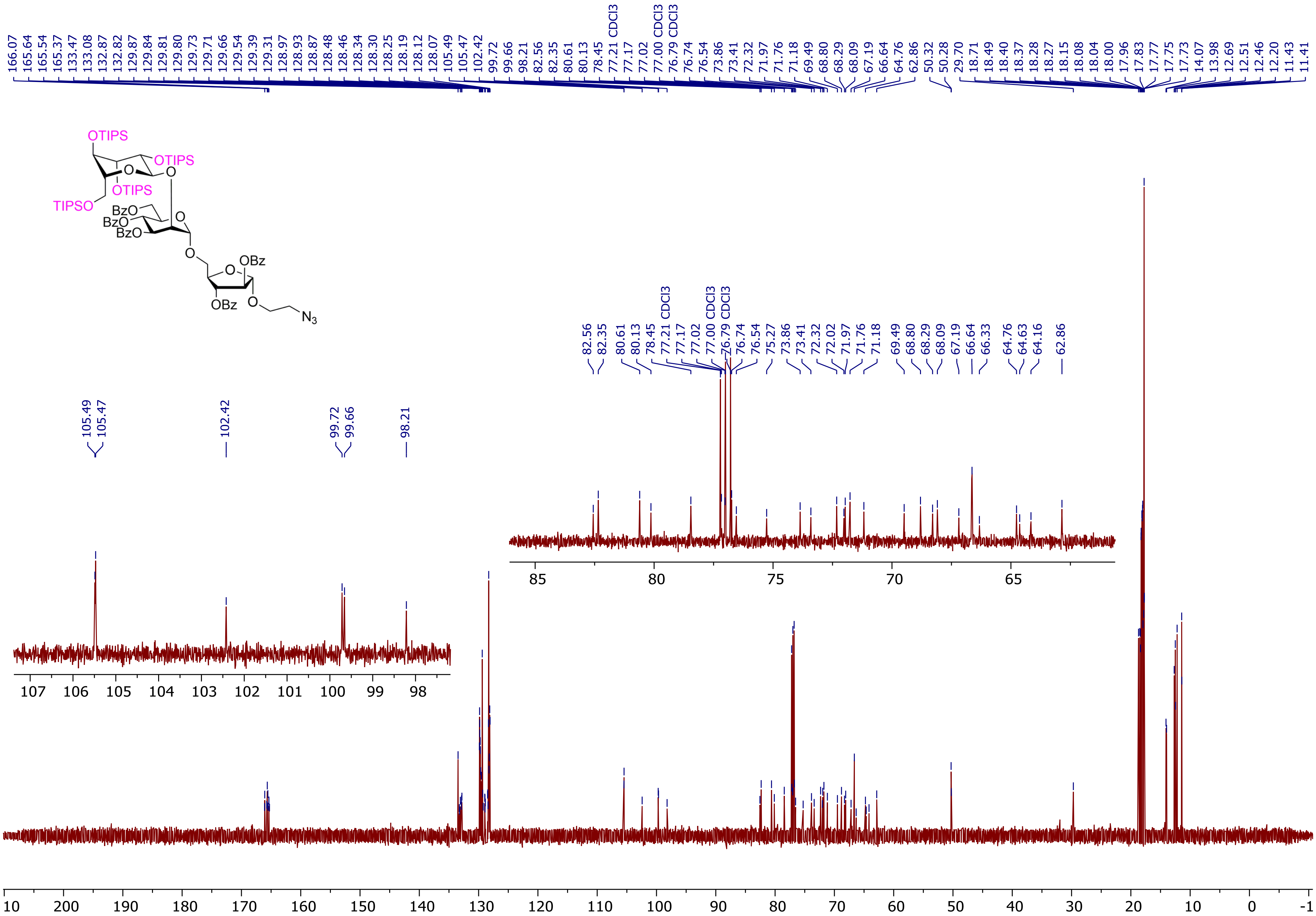

COSY (600 MHz) spectrum of compound 24 in $CDCl_3$ (244K)

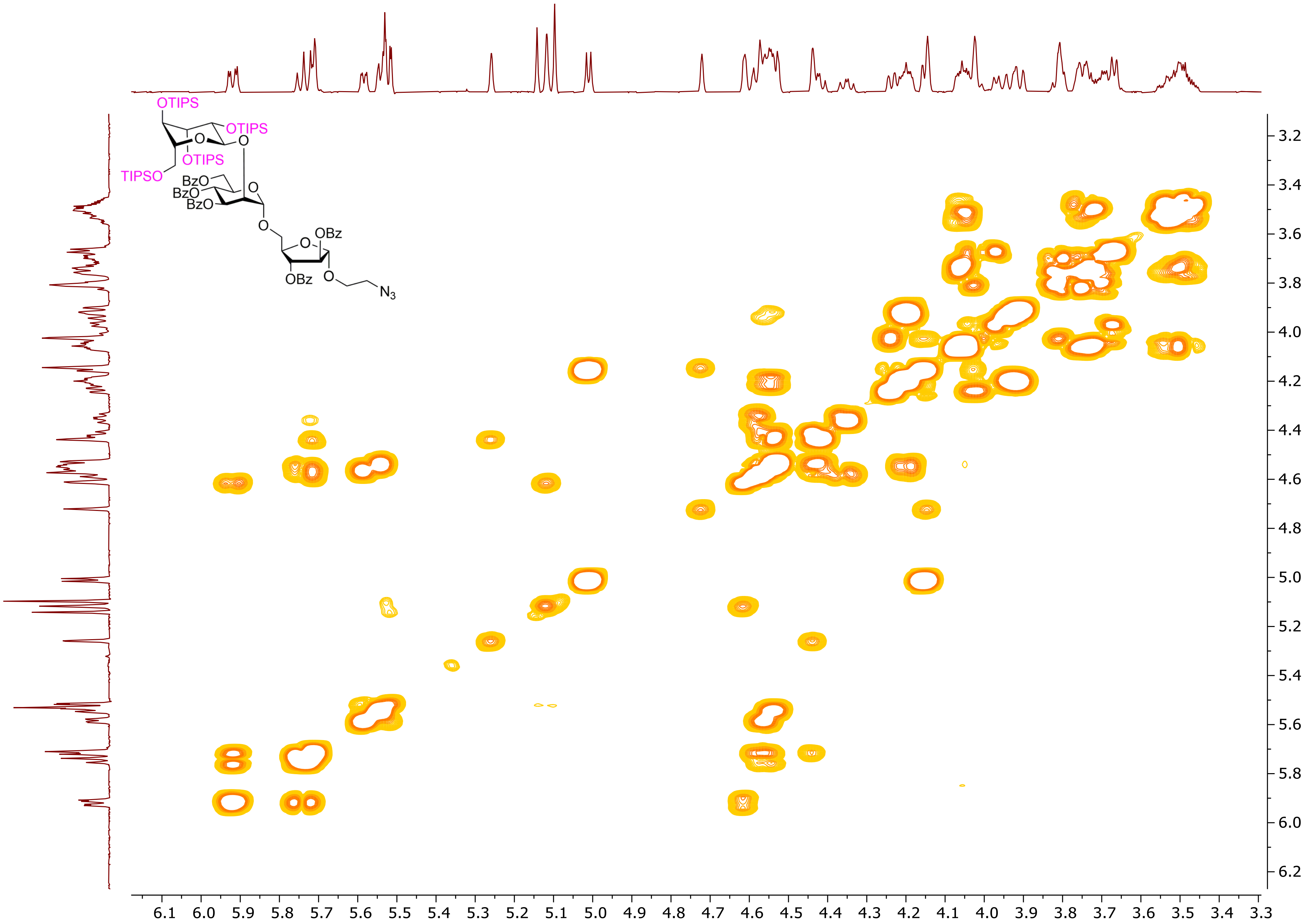

HSQC (600 MHz) spectrum of compound 24 in $CDCl_3$ (244K)

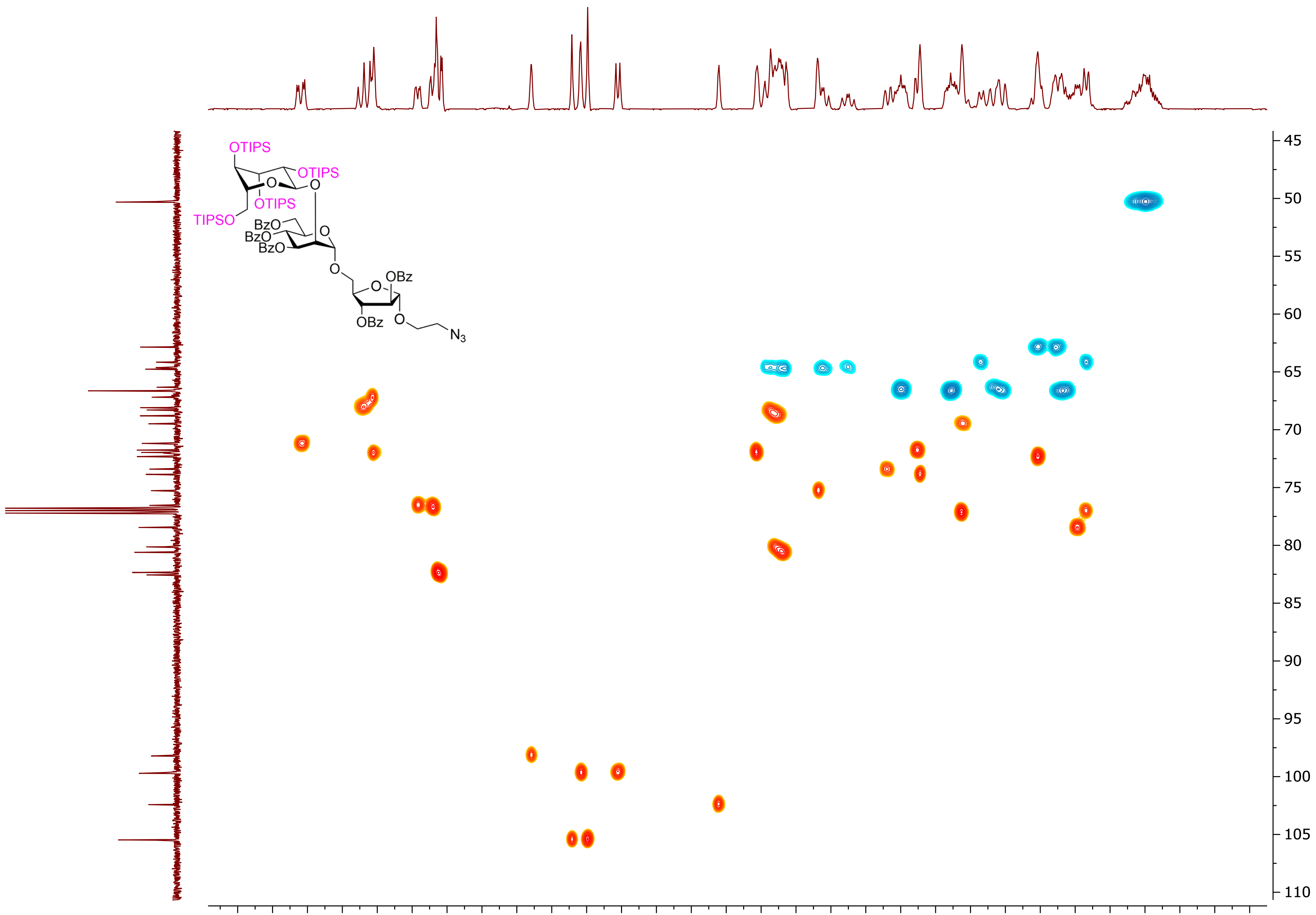

HMBC (600 MHz) spectrum of compound 24 in $CDCl_3$ (244K)

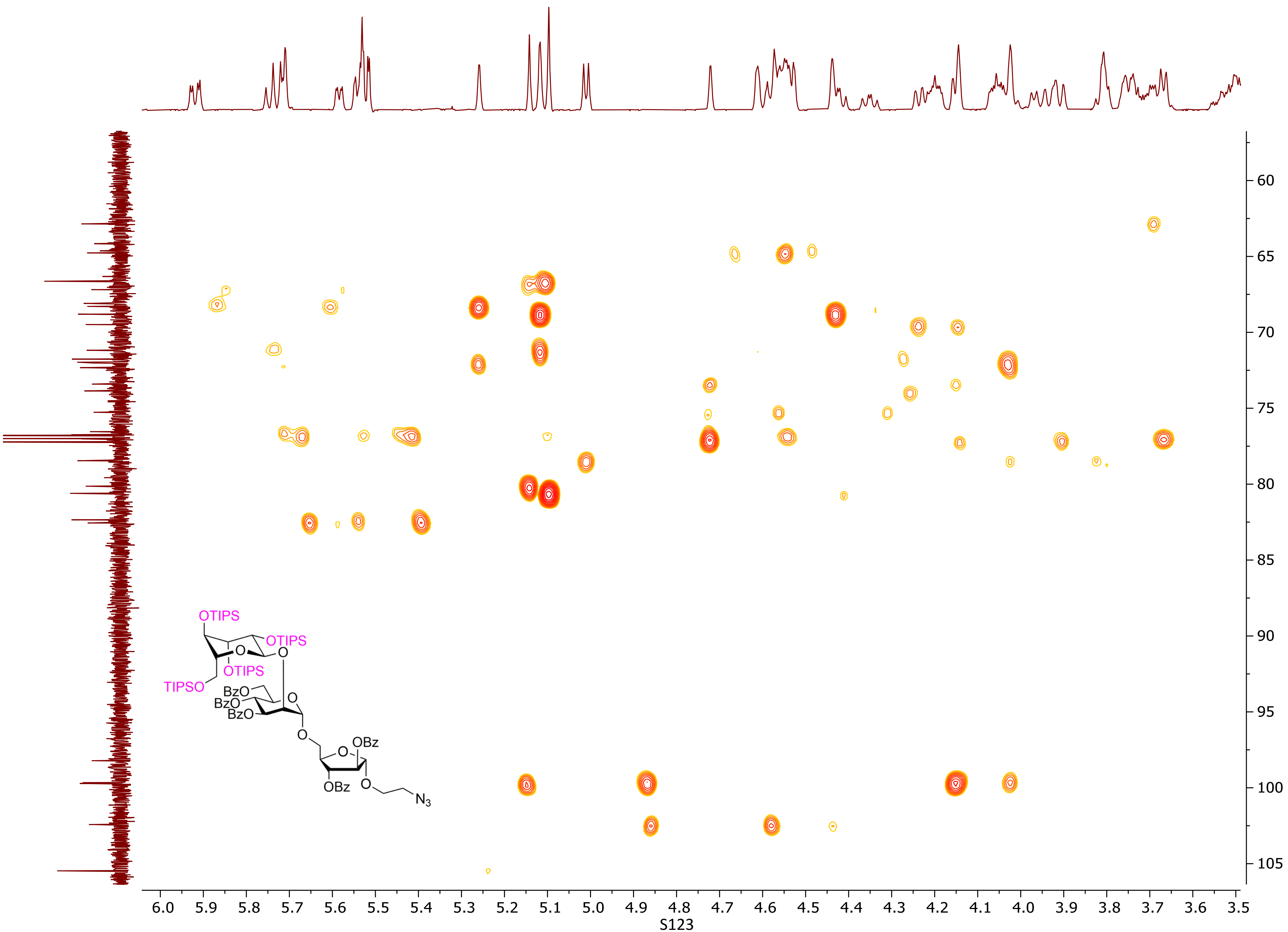

^{29}Si INEPT NMR (119 MHz) spectrum of compound 24 in $CDCl_3$ (244K)

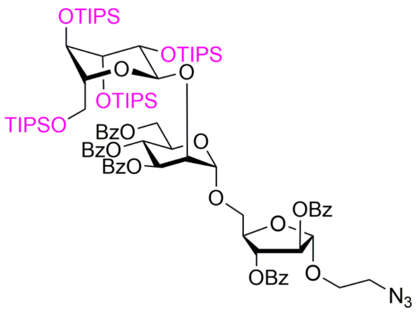

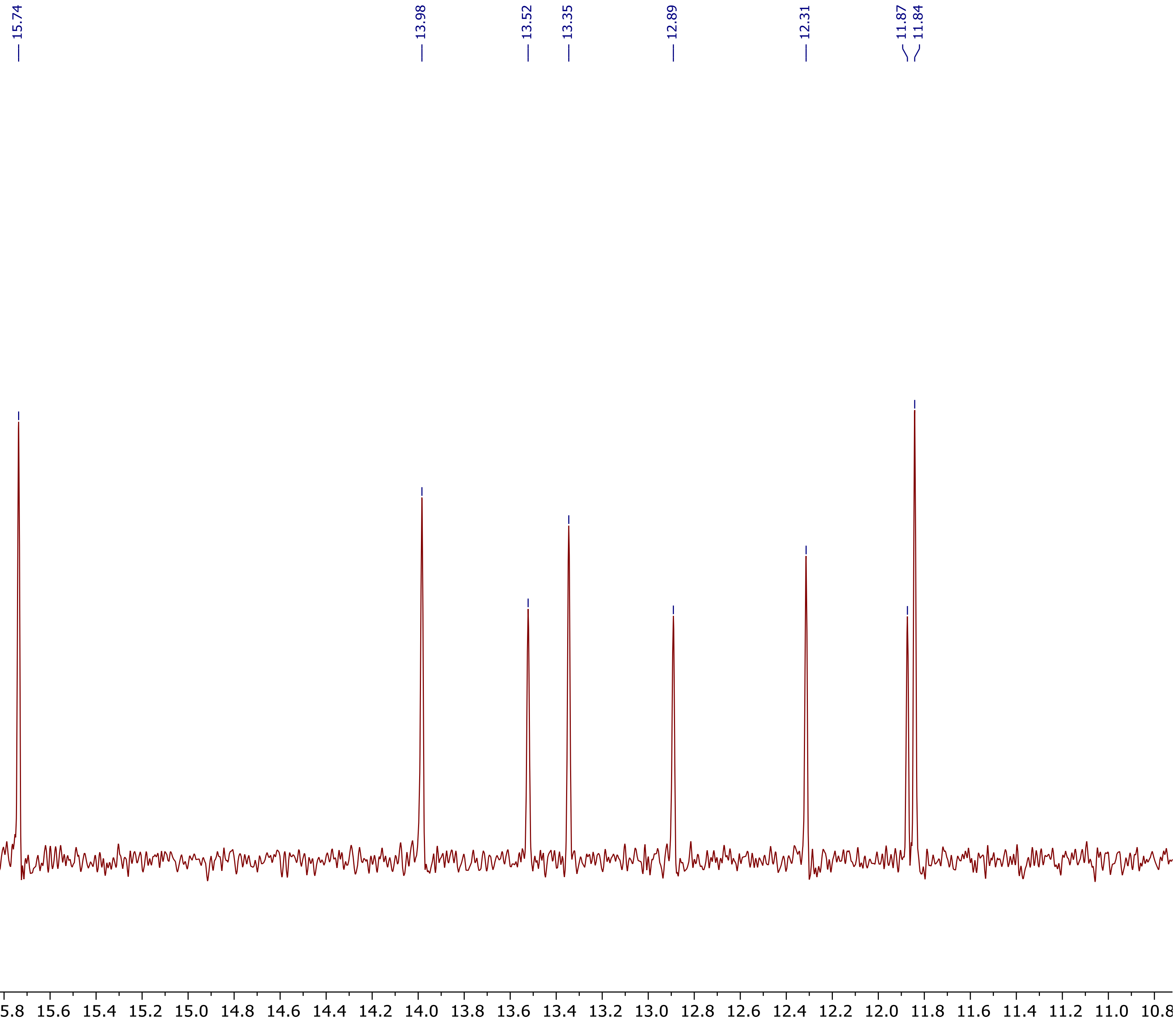

^{1}H NMR (600 MHz) spectrum of compound 25 in CD_3OD

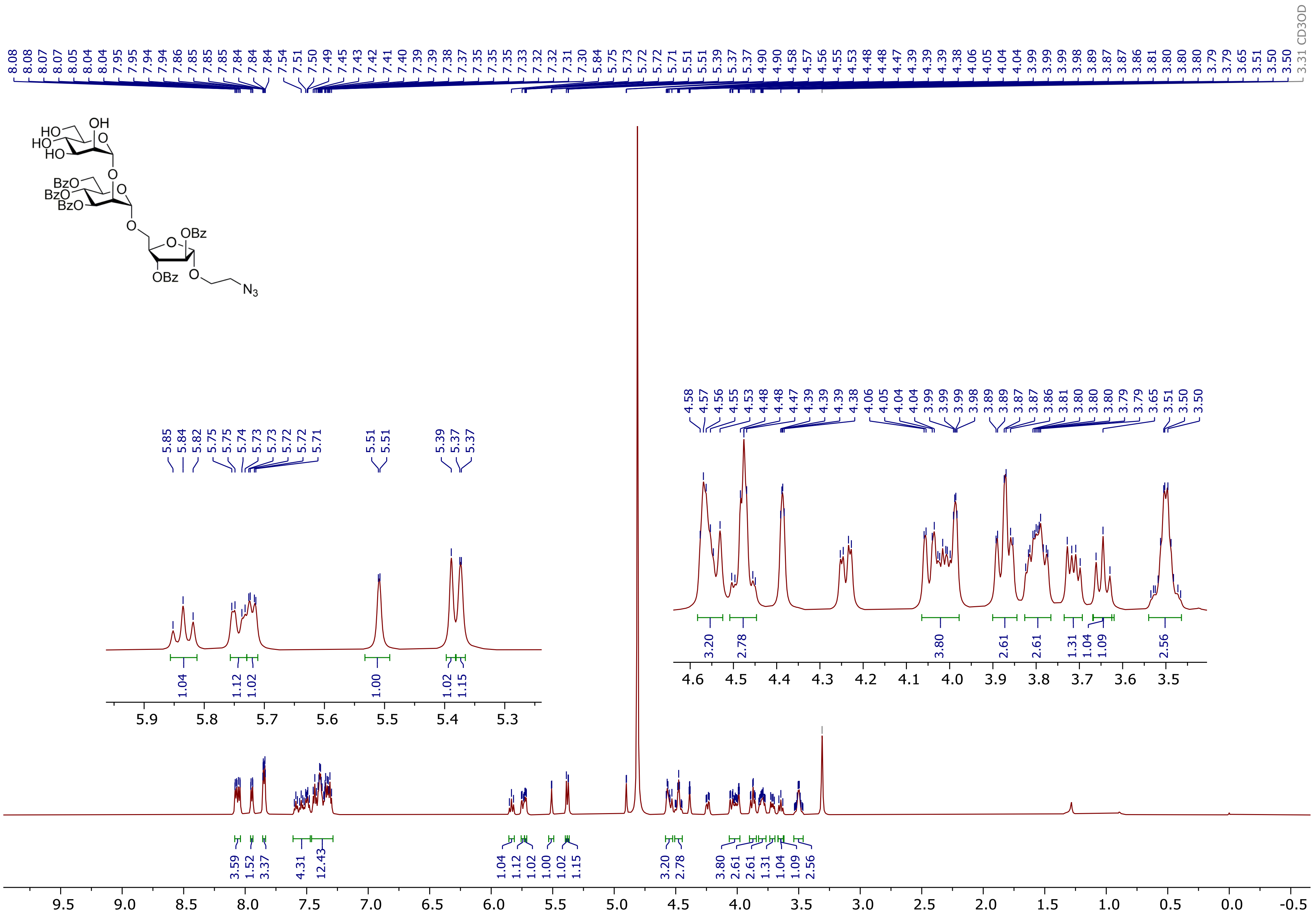

^{13}C NMR (151 MHz) spectrum of compound 25 in CD_3OD

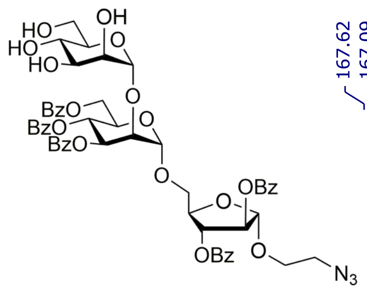

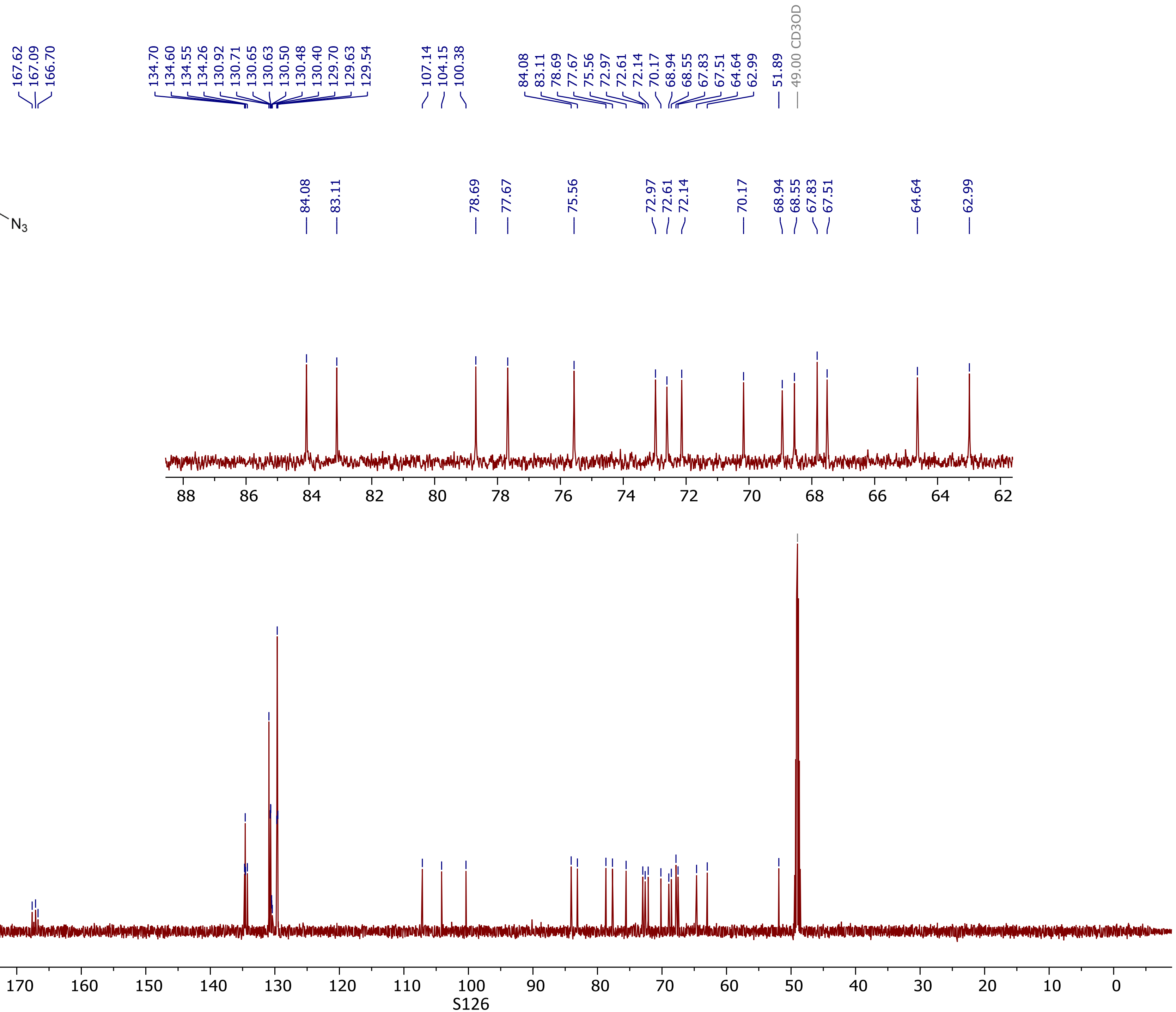

COSY (600 MHz) spectrum of compound 25 in CD_3OD

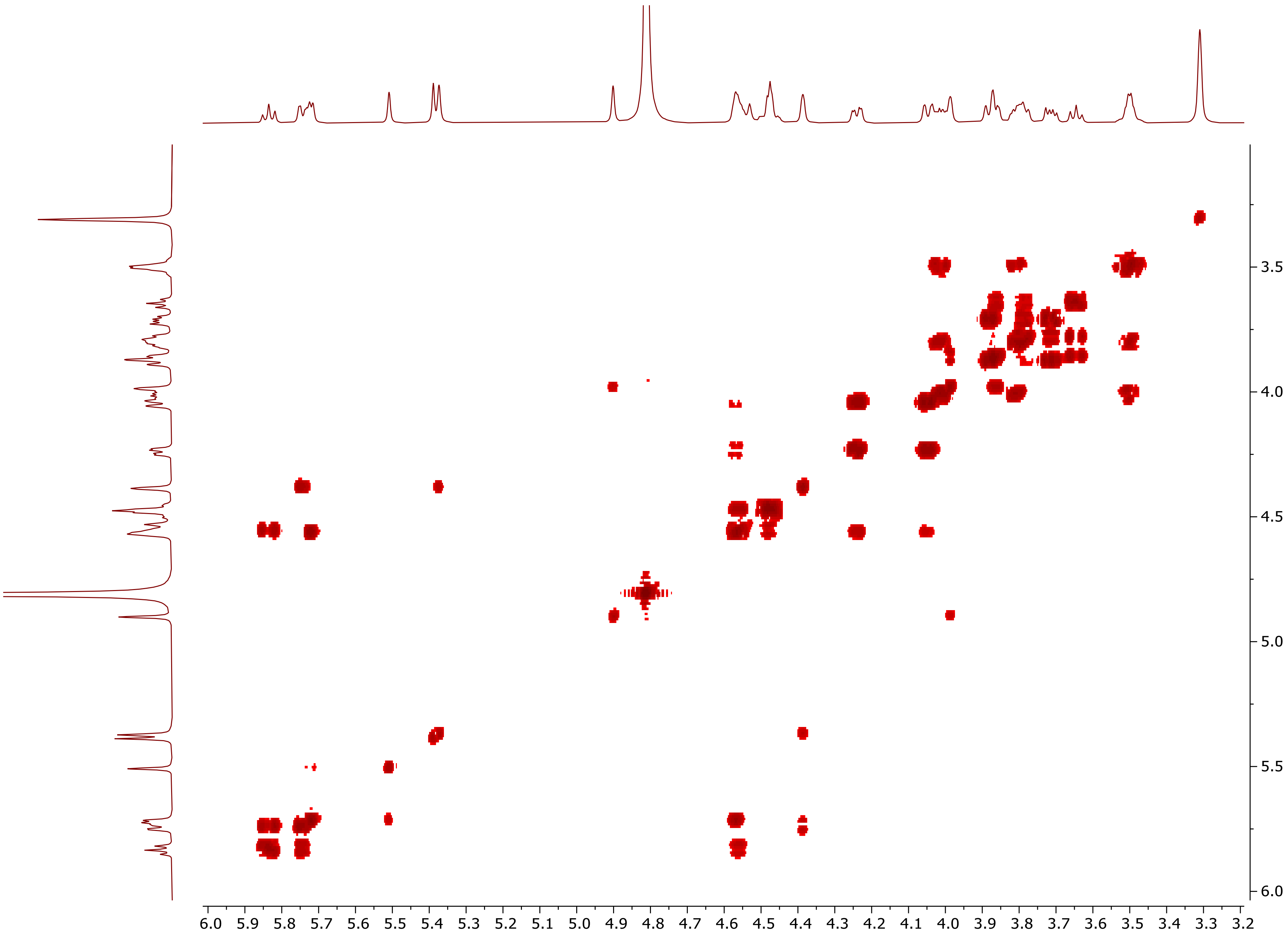

HSQC (600 MHz) spectrum of compound 25 in CD_3OD

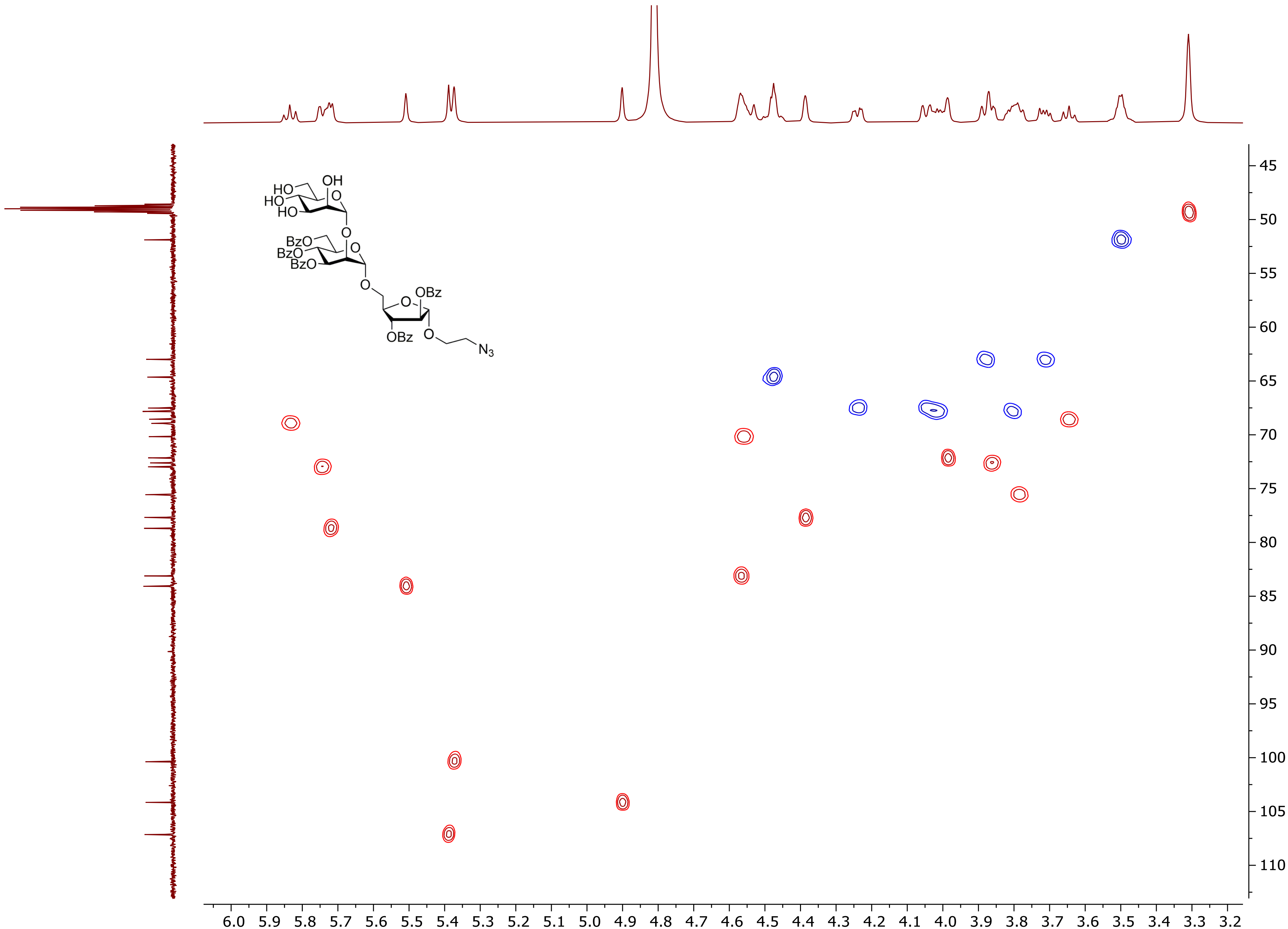

HMBC (600 MHz) spectrum of compound 25 in CD_3OD

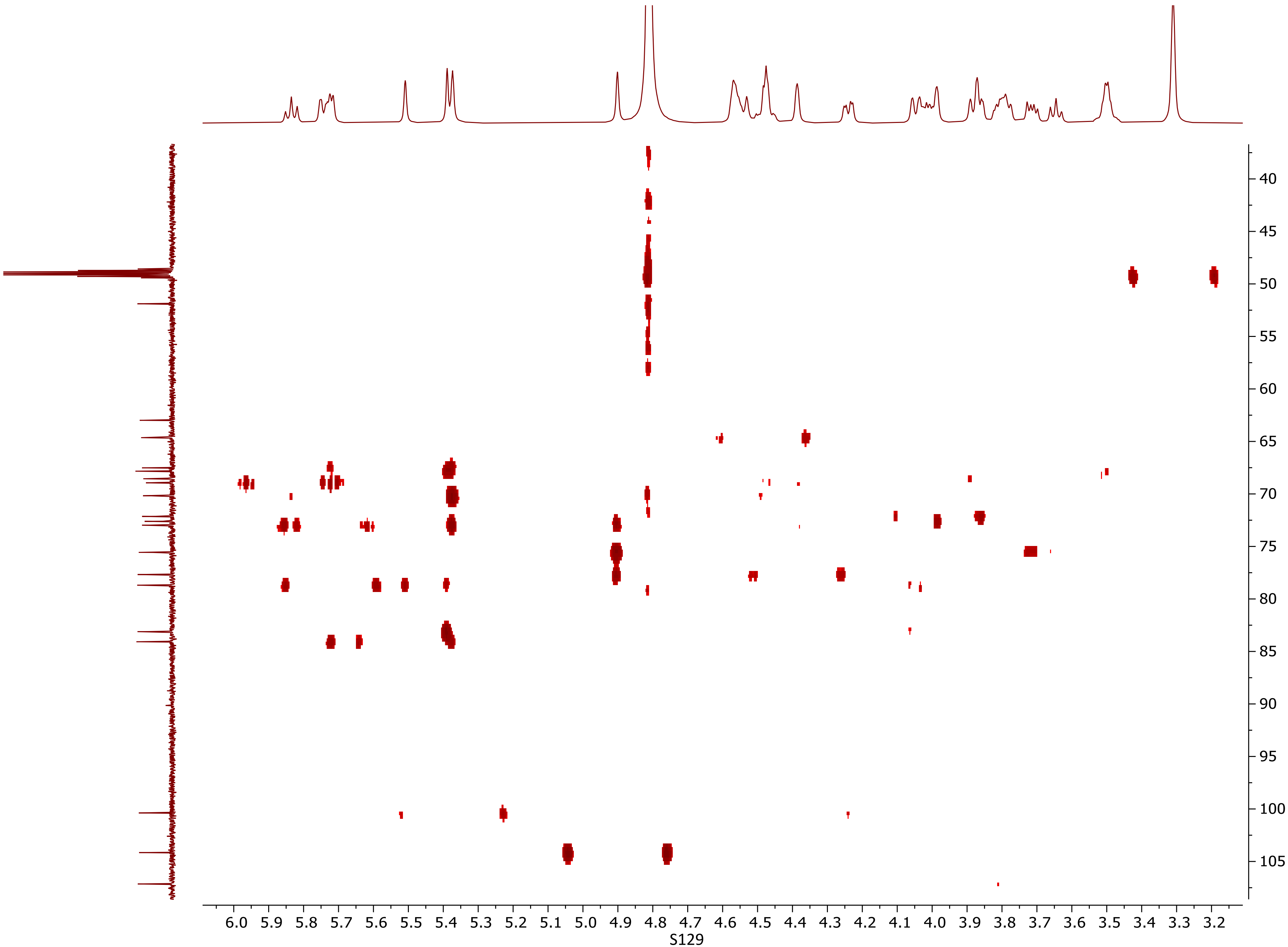

^1H NMR (600 MHz) spectrum of compound 26 in CD_3OD

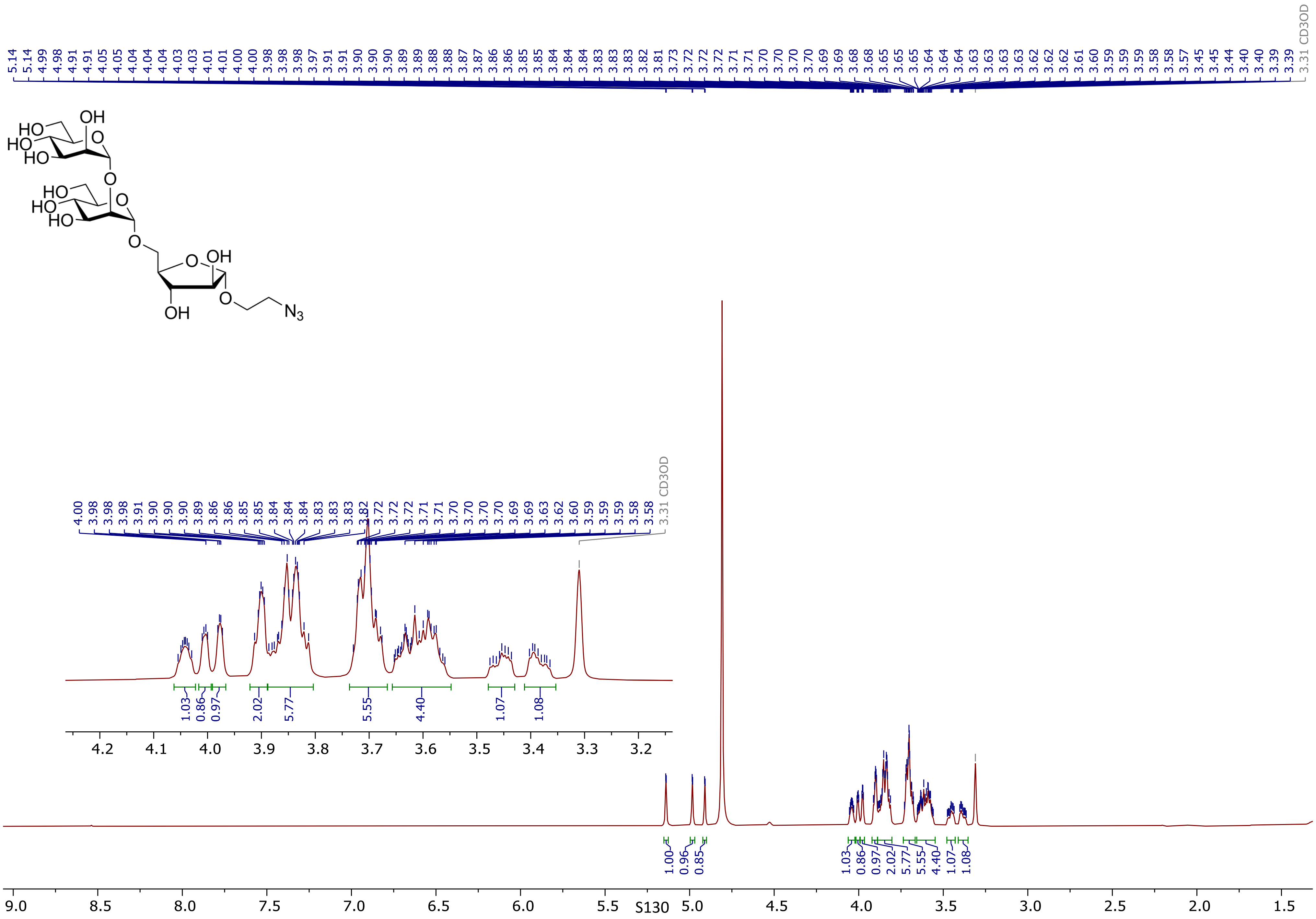

^{13}C NMR (151 MHz) spectrum of compound 26 in CD_3OD

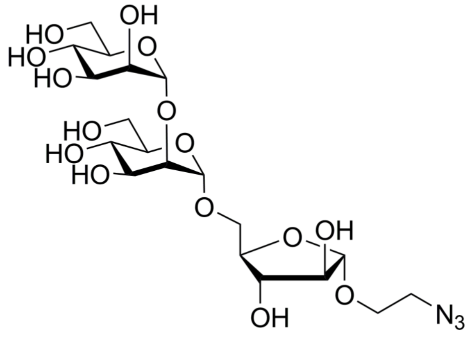

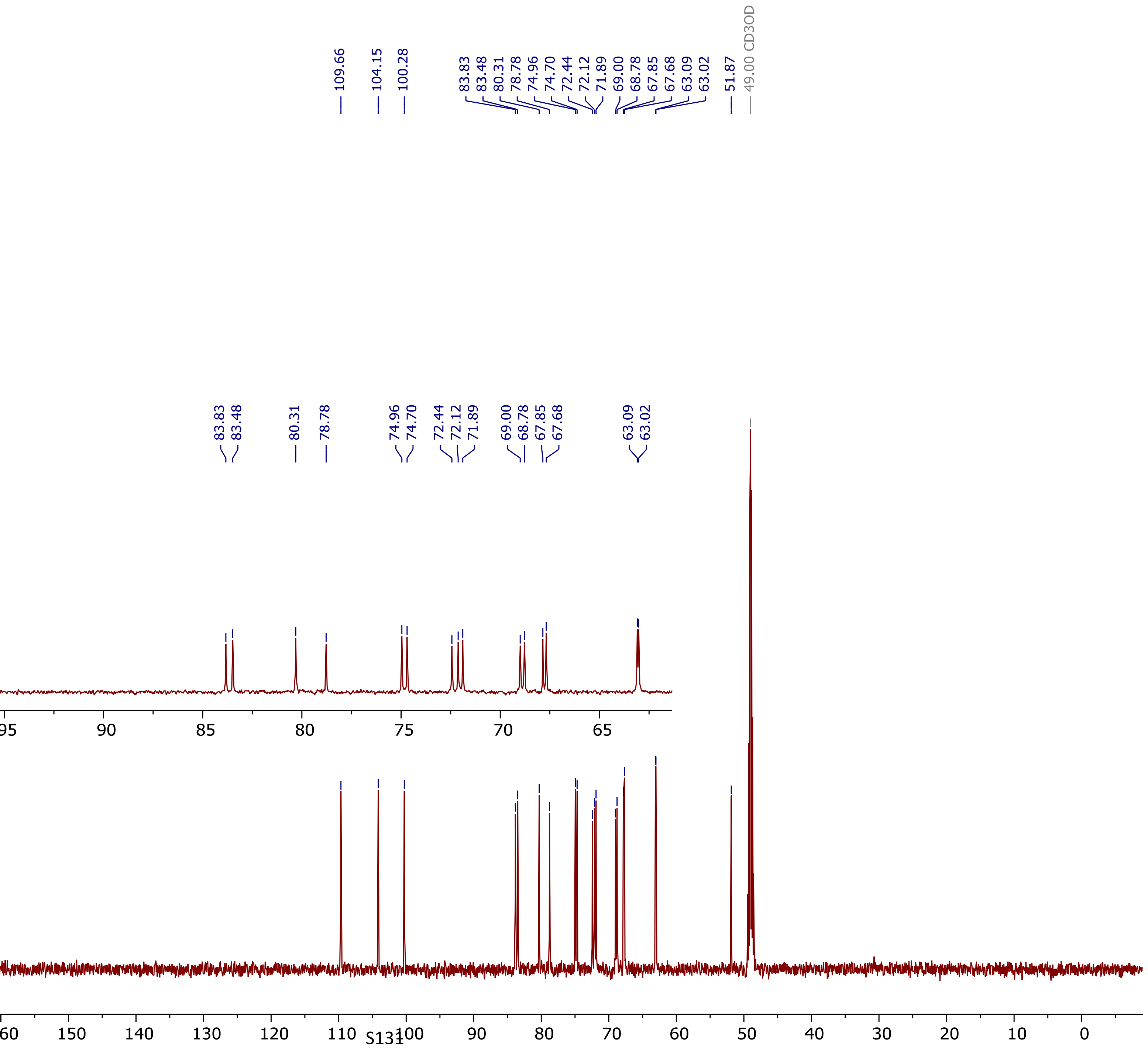

COSY (600 MHz) spectrum of compound 26 in CD_3OD

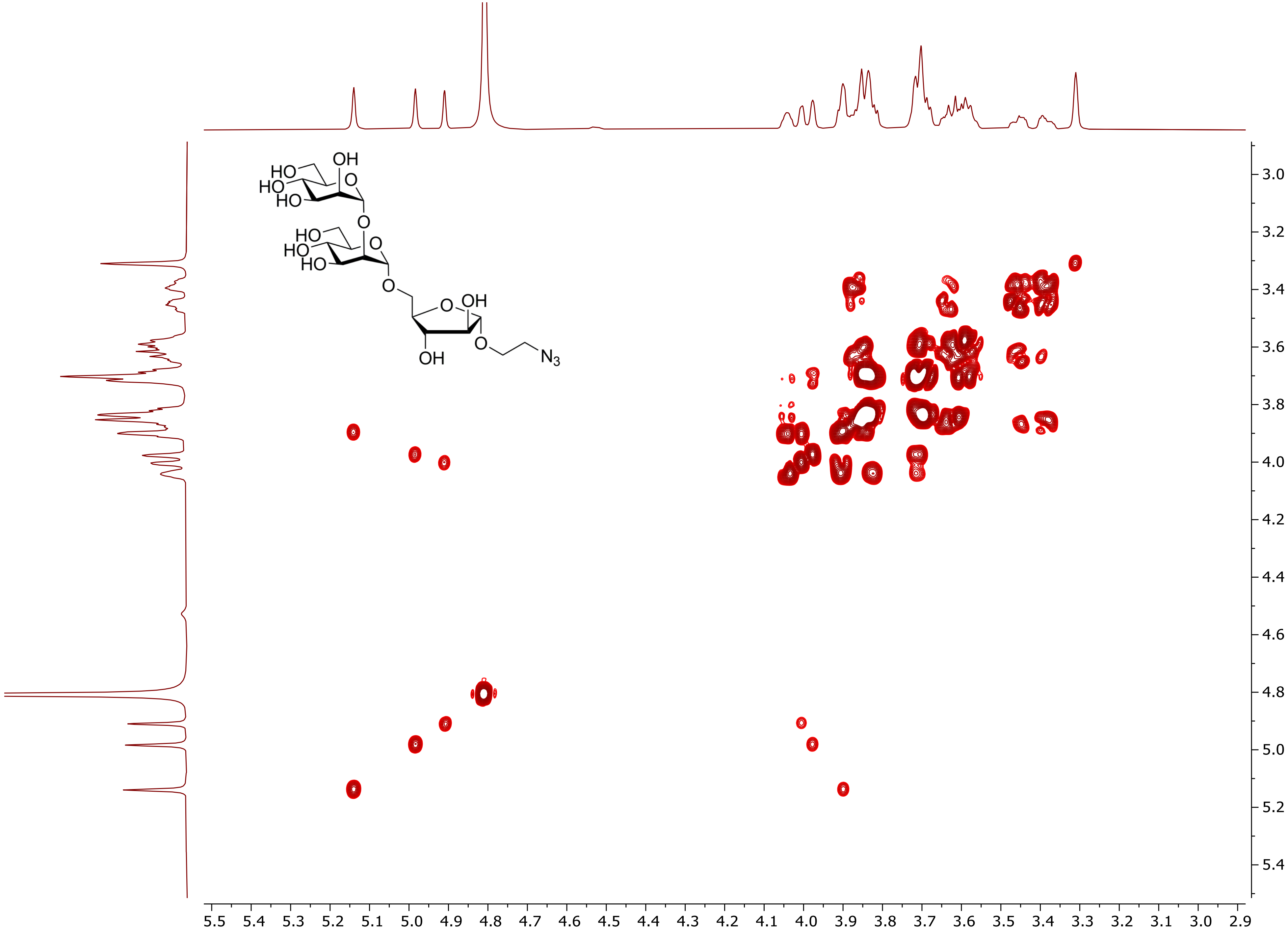

HSQC (600 MHz) spectrum of compound 26 in CD_3OD

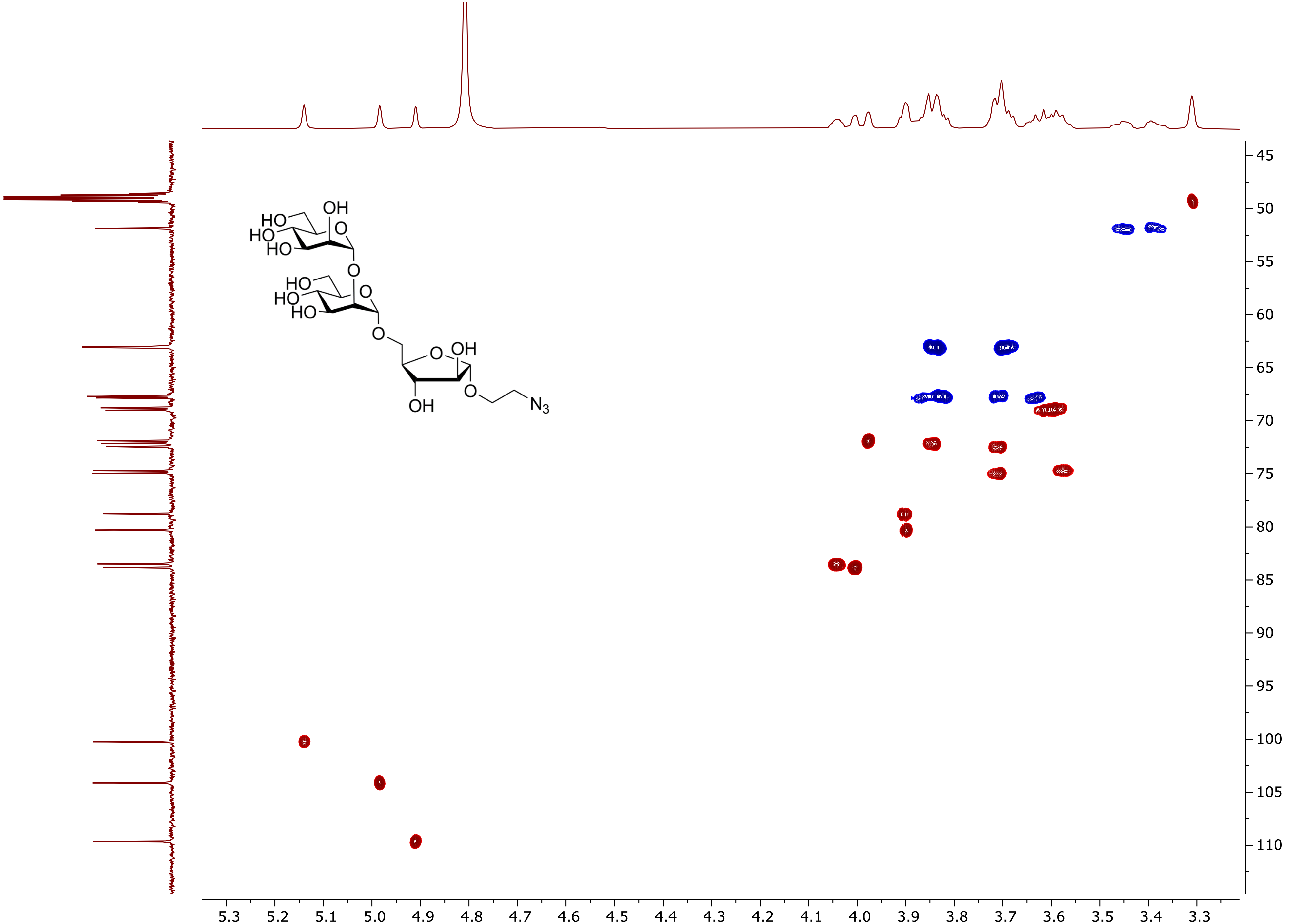

HMBC (600 MHz) spectrum of compound 26 in CD_3OD

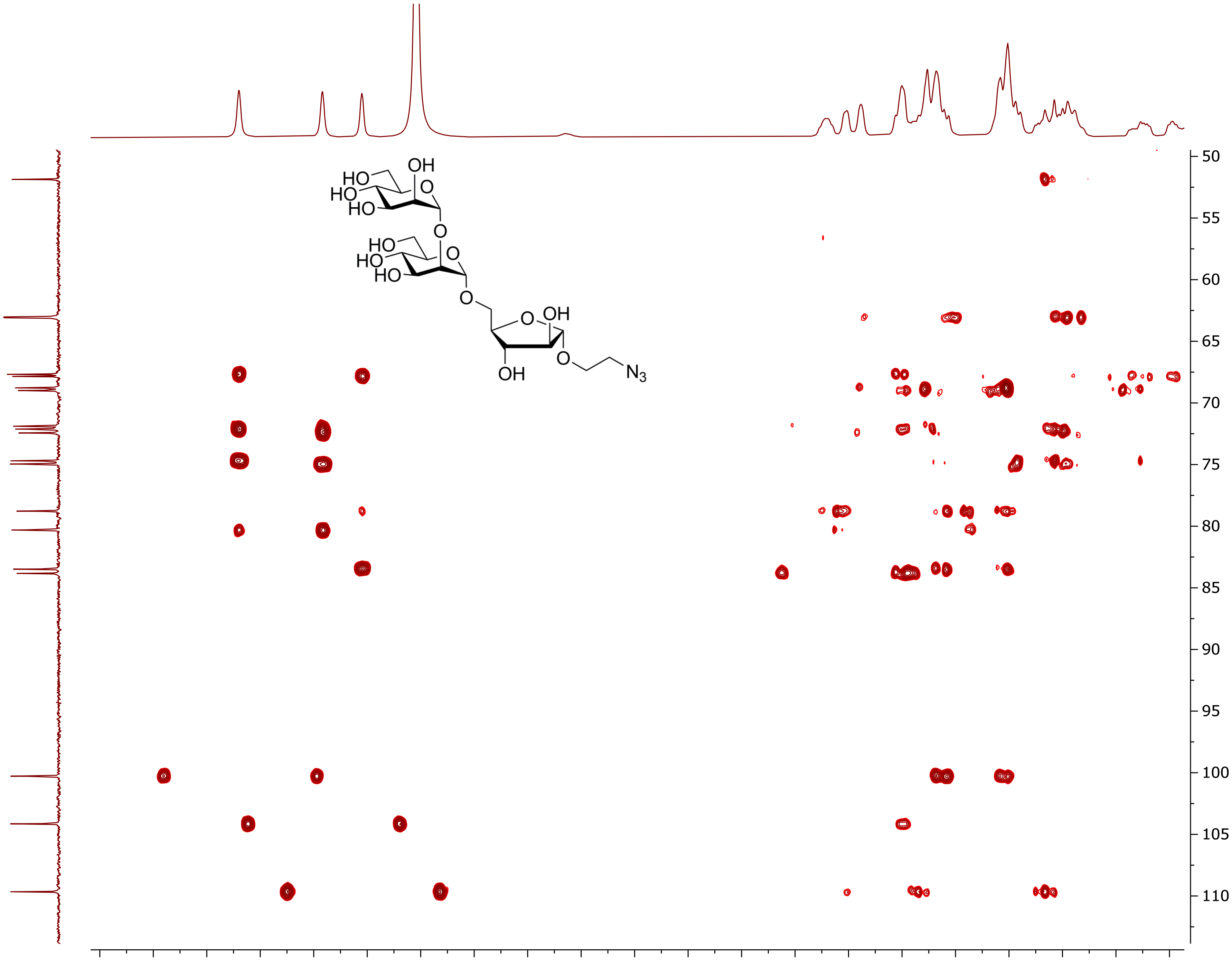

Supplement: Supplementary file 1 [file molecules-31-01598-s001.zip › molecules-4264562-supplementary.pdf]
